# The Burden and Trends of Gynecological Cancers in Asia from 1980 to 2021, with Projections to 2050: A Systematic Analysis for the Global Burden of Disease Study 2021

**DOI:** 10.3390/curroncol32060298

**Published:** 2025-05-23

**Authors:** Yang Yang, Run Miao, Haoyu He, Ning Zhang, Xingyu Wan, Yuzhou Gao, Dongmei Ji

**Affiliations:** 1 Department of Obstetrics and Gynecology, The First Affiliated Hospital of Anhui Medical University, Hefei 230022, China; 2 First School of Clinical Medicine, Anhui Medical University, Hefei 230032, China; 3 Second School of Clinical Medicine, Anhui Medical University, Hefei 230032, China; 4 NHC Key Laboratory of Study on Abnormal Gametes and Reproductive Tract (Anhui Medical University), No. 81 Meishan Road, Hefei 230032, China; 5 Key Laboratory of Population Health Across Life Cycle (Anhui Medical University), Ministry of Education of the People’s Republic of China, No. 81 Meishan Road, Hefei 230032, China

**Keywords:** gynecological cancers, cervical cancer, ovarian cancer, uterine cancer, Asia, disease burden

## Abstract

Gynecological cancers pose a significant threat to women’s health. This study aimed to investigate the disease burden of cervical, uterine, and ovarian cancers in Asia from 1980 to 2021. The Global Burden of Disease 2021 database (GBD 2021) was used to conduct a cross-sectional study. The incidence, mortality rates, and disability-adjusted life years (DALYs) were obtained as indicators to estimate the burden. The effects of age, period, and cohort on the incidence of gynecological cancers were analyzed via the age-period-cohort web tool (APC-Web). The future trends of the gynecological cancer burden in Asia from 2025 to 2050 were predicted via a Bayesian age-period-cohort model. In 2021, cervical cancer exhibited the highest age-standardized mortality burden (3.1 deaths per 100,000; 95% UI: 2.7–3.4), whereas uterine cancer had the lowest (0.7 deaths per 100,000; 95% UI: 0.6–0.9). Geographically, South Asia has experienced the highest cervical cancer burden, with Seychelles, Mongolia, Cambodia, and Nepal ranking among the most affected nations. In contrast, Central Asia had the highest ovarian cancer burden, led by Georgia, followed by the United Arab Emirates, Seychelles, and Brunei Darussalam. Similarly, the uterine cancer burden was most pronounced in Central Asia, with Georgia, Armenia, Mauritius, and the United Arab Emirates exhibiting elevated rates. Finally, increasing trends in the burden of gynecological cancers were predicted across all age groups from 2025 to 2050, with women aged 60 to 64 years being the most affected. In conclusion, gynecological cancers are significant contributors to the disease burden in Asia. Improved early screening methods are essential to mitigate this increasing burden.

## 1. Introduction

Gynecological cancers include a range of malignancies originating from female reproductive organs, with the primary types being cervical, ovarian, and uterine cancers (endometrial cancer and uterine sarcoma). They are characterized by their potential to induce severe physical consequences, including infertility, hormonal imbalances, sexual dysfunction, and long-term disability. Gynecological cancers accounted for 19% of the overall female cancer burden globally [1]. The World Health Organization (WHO) reported approximately 604,127 incident cases and 314,831 deaths from cervical cancer in 2020, making it the fourth most frequently diagnosed cancer and the fourth leading cause of cancer-related mortality among women [2]. In 2019, estimations showed approximately 294,422 (95% UI: 260,649 to 329,727) incident cases of ovarian cancer and 198,412 (95% UI: 175,357 to 217,665) deaths, accounting for 5% of female oncology deaths worldwide, which exceeded any other gynecological cancer [3]. For uterine cancer, 435,041 (95% UI: 397,021.1 to 479,728.7) incident cases and 91,640 (95% UI: 82,389.2 to 101,502.1) deaths were reported globally in 2019, standing out as the most common gynecological cancer of the female genital tract in developed countries [4]. These incident cases and deaths caused by gynecological cancers result in significant health and economic losses, encompassing both reproductive risks and direct or indirect financial costs.

The distribution of gynecological cancers is geographically specific, with particular concern reported in Asia. Studies have revealed that more than half of all cases of cervical cancer occurred in the Asia–Pacific region [5]. Zhou et al. reported that most global ovarian cases were distributed in East, South, and Southeast Asia based on the Global Burden of Diseases Study 2017 (GBD 2017) [6]. Additionally, in Japan, Yamagami et al. reported that the prevalence of endometrial cancer continues to increase, making it the most common native gynecologic malignant tumor [7]. Although previous studies have investigated the gynecological cancer burden in Asia, they relied on relatively older data [6], examined a single cancer [8], focused on specific regions or countries [9], investigated the burden over a short period [10], or lacked advanced statistical analyses [11]. The substantial gynecological cancer burden in Asia underscores the urgent need for more attention to be brought to this severe public issue.

Our study provides the latest comprehensive assessment of the disease burden of gynecological cancers in Asia from 1980 to 2021, projecting future trends from 2025 to 2050. Data for three types of gynecological cancers (cervical cancer, ovarian cancer, and uterine cancer) were collected from GBD 2021. The age-standardized mortality rate (ASMR), age-standardized DALY rate (ASDR), and age-standardized incident rate (ASIR) were reported by year, location, and sociodemographic index (SDI) level. Pearson correlation was conducted to explore the correlation between these indicators and SDI levels. A Bayesian age-period-cohort (BAPC) model was employed to project the trends in disease burden up to 2050.

## 2. Materials and Methods

### 2.1. Data Collection

The study follows the PRISMA and GATHER guidelines. Our study utilized data from the GBD 2021, accessed via the Global Health Data Exchange (GHDx) results tool (https://ghdx.healthdata.org/) (accessed on 22 February 2025). The GBD 2021 is considered the most comprehensive rigorous assessment of the global epidemiological burden, encompassing data on 371 diseases, injuries, and 88 risk factors [12]. The data used in the Global Burden of Disease (GBD) study are derived from a variety of sources, including scientific literature, vital statistics, surveys, censuses, administrative records, registries, individual studies, reports, and satellite imagery. Mortality associated with International Classification of Diseases (ICD) codes for deaths linked to gynecological cancers was modeled by categorizing the cause-of-death data into five GBD risk factor categories. Ambiguous or poorly defined ICD codes were reallocated to more accurate cause categories via regression or proportional redistribution methods. Bayesian models were primarily employed to estimate incidence and prevalence over time, geography, age, and sex, incorporating relevant data and predictive factors. The specific modeling approach varies by condition, frequently utilizing a Bayesian meta-regression tool called Disease Modeling Meta-Regression (DisMod-MR) 2.1.

We employed mortality, DALYs, and incidence as indicators to quantify the disease burden across different years, locations, age groups, and SDI levels. We obtained death data from 1980 to 2021, and the data for DALY and incidence from 1990 to 2021 were collected. The 204 countries and territories were divided into 7 GBD super regions and 21 GBD regions. Regionally, we specifically selected five GBD regions in Asia (Central Asia, East Asia, High-income Asia–Pacific, South Asia, and Southeast Asia). Nationally, we gathered related data from 49 Asian countries. The selected GBD regions and countries are listed in the Supplementary Methods in Supplementary Materials. Furthermore, we divided the study regions into five tiers based on their SDI levels: Low SDI, Low-middle SDI, Middle SDI, High-middle SDI, and High SDI regions. The calculation process of the SDI is described in the Supplementary Methods.

Given the increasing risk of gynecological cancers in specific age groups and the challenges posed by partial data availability, we focused our data collection on individuals aged 15 to 95 plus for cervical and ovarian cancers. For uterine cancer, data were gathered for individuals aged 20 to 95 plus. We divided the population into 17 age groups for detailed analysis. These groups are structured in five-year intervals: 15 to 19, 20 to 24, 25 to 29, 30 to 34, 35 to 39, 40 to 44, 45 to 49, 50 to 54, 55 to 59, 60 to 64, 65 to 69, 70 to 74, 75 to 79, 80 to 84, 85 to 89, 90 to 94, and 95 plus. This stratification enables a precise assessment of age-specific disease burden and trends.

### 2.2. Measures Employed as Indicators of Disease Burden

We obtained incidence, mortality, and DALY as indicators to assess the disease burden of gynecological cancers. DALY is a comprehensive measure of disease burden, representing the total years lost due to disability or premature death. It combines years of lost life (YLLs) due to early mortality with years lived with disability (YLDs) to account for the impact of both mortality and morbidity. This metric allows for a more holistic comparison of the effects of different diseases or conditions, capturing both fatal and nonfatal outcomes.

### 2.3. Statistical Analysis

Age standardization was carried out via the direct method, which is based on the GBD standard global population structure. The reference population is derived from the demographic composition of all countries with populations exceeding five million. This method involves calculating the proportion of the population within each age group for each location and then averaging these age-specific proportions across all locations. The ASMR, ASDR, and ASIR, along with their 95% uncertainty intervals (UI), were calculated across year, location, and SDI levels to isolate the effects of age structures. Each ASR was calculated via the following formula:

$$ASR=\frac{\sum_{i=1}^{n}\;\left(r_{i}\cdot N_{i}\right)}{\sum_{i=1}^{n}\;N_{i}}$$

where $r_{i}$ is the age-specific rate for the *i*-th age group, $N_{i}$ is the number of individuals in the standard population for the *i*-th age group, and n is the total number of age groups.

We conducted a Pearson correlation analysis to calculate the correlation coefficients via a linear regression model, identifying the correlations between SDI levels and disease burden. Pearson correlation analysis is a statistical method used to assess the strength and direction of the linear relationship between two continuous variables. It provides a correlation coefficient ranging from −1 to 1, where values closer to 1 or −1 indicate a strong positive or negative linear relationship, respectively, and values near 0 suggest no linear correlation. The correlation coefficient (r) was calculated via the following formula:

$$r=\frac{\sum (x_{i}-\bar{x})(y_{i}-\bar{y})}{\sqrt{\sum (x_{i}-\bar{x}{)}^{2}\sum (y_{i}-\bar{y}{)}^{2}}}$$

where r represents the Pearson correlation coefficient, which measures the strength and direction of the linear relationship between two variables. $x_{i}$ and $y_{i}$ are the individual values for the SDI levels and death rates, respectively, while $\bar{x}$ and $\bar{y}$ represent the mean values of the SDI levels and death rates, respectively.

Decomposition analysis was performed to assess the contributions of three critical factors: population, aging, and epidemiological changes to the disease burden. Decomposition analysis is a quantitative method used to assess the contributions of three major factors: population, aging, and epidemiological changes to changes in DALYs due to gynecological cancers. This method was thoroughly described by Gupta [13]. This analysis enables researchers to determine whether observed trends are primarily due to changes in disease rates or demographic shifts, offering a clearer understanding of the underlying drivers of disease burden. The change in DALYs attributable to different factors was calculated using the following formula:

$${DALY}_{ay,py,ey}=\sum_{i=1}^{17}\left(a_{i,y}^{⋆}p_{y}^{⋆}e_{i,y}\right)$$

where ${\;DALY}_{ay,py,ey}$ represents DALYs based on the factors of age structure, population, and DALYs rate for a specific year *y*; *a_i,y_* represents the proportion of the population for the age category *i* of the 17 age categories in given year *y*; *p_y_* represents the total population in given year *y*; and *e_i,y_* represents DALYs rate given age category *i* in year *y*. The contribution of each factor to the change in DALYs from 1990 to 2021 was defined by the effect of one factor changing while the other factors remained constant.

In addition, temporal trends in disease burden were modeled via joinpoint analysis and the age-period-cohort web (APC-Web) tool. We calculated the estimated annual percent changes (EAPCs), average annual percent change (AAPC), local drifts (age-specific annual percentage changes), and net drifts (overall annual percentage changes) accompanying their 95% confidence intervals (CI) to show the variation trends of age-standardized and age-specific rates. Among these, the EAPC measures the average change in rates over a defined time period. In this analysis, we assumed that the natural logarithm of the ASR follows a linear regression model, which enables the estimation of long-term trends in disease burden. The detailed methods for the use of APC-Web tool and the calculation of other indicators are described in the Supplementary Methods. The EAPC was calculated via the following formula:

y=α+βx+ε

$$EAPC=100\%\times (e^{\beta }-1)$$

where y represents the natural logarithm of the age-standardized rate and where x represents the calendar year. The coefficient *β* is the value we obtain from the linear regression.

Finally, using our BAPC projection model, we forecasted the disease burden from 2025 to 2050. This model is specifically designed for Bayesian age-period-cohort analysis and is implemented through a dedicated software package for projections. BAPC uses integrated nested Laplace approximations (INLA) to conduct detailed Bayesian inference, providing both age-specific and age-standardized projected rates. Moreover, the BAPC model automatically incorporates Poisson noise into its framework to account for the inherent variability in the predictive distribution.

In our statistical analysis, when the *p* value fell below 0.05, it was deemed statistically significant. The data used in this study were constructed, collated, and analyzed via R software (version 4.3.1). The data were processed and refined via the R packages “tidyverse” and “writexl”. For data visualization, we utilized “ggplot2”, “RColorBrewer”, “patchwork”, and “ggsci” to illustrate the disease burden. Additionally, the “easyGBDR” package was applied for parts of the statistical analysis and corresponding visualizations.

## 3. Results

### 3.1. Disease Burden of Gynecological Cancers in Asia

**Figure 1:**
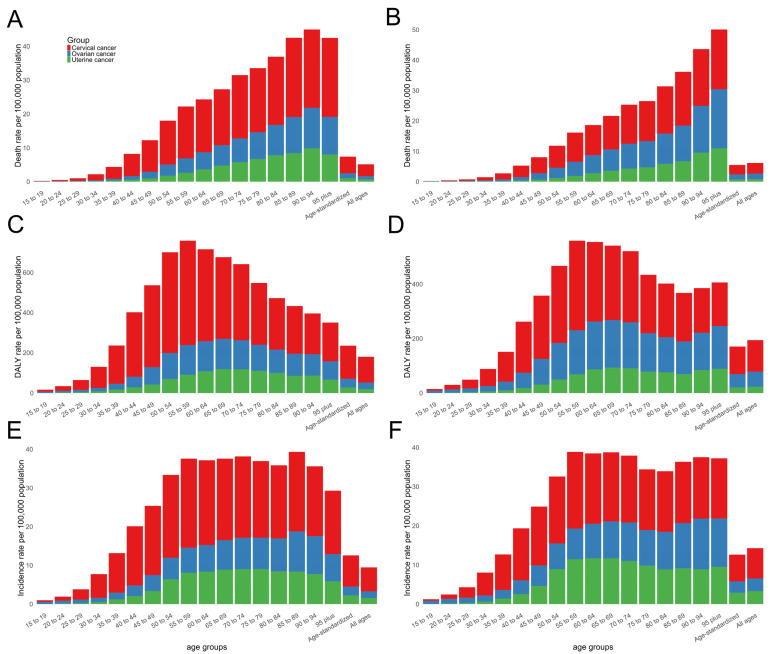
Global age-specific disease burden attributable to three types of gynecological cancers by age group for mortality rate in 1980 (**A**); mortality rate in 2021 (**B**); DALY rate in 1990 (**C**); DALY rate in 2021 (**D**); incidence rate in 1990 (**E**); and incidence rate in 2021 (**F**). DALY = disability-adjusted life year.

In 2021, cervical cancer exhibited the highest disease burden in Asia among the three types of gynecological cancers, whereas uterine cancer had the lowest burden. In Asia, for cervical, ovarian, and uterine cancer, the all-age deaths were 160,723.84 (95% UI: 143,073.14 to 179,396.56), 84,278.89 (95% UI: 73,874.6 to 97,734.25), and 37,508.92 (95% UI: 31,519.05 to 45,758.59) cases, respectively (Table 1). The corresponding ASMRs (per 100,000 population) were 3.10 (95% UI: 2.75 to 3.47), 1.64 (95% UI: 1.44 to 1.91), and 0.74 (95% UI: 0.62 to 0.91) cases, with the EAPCs from 1980 to 2021 of −1.68 (95% CI: −1.77 to −1.59), 0.43 (95% CI: 0.33 to 0.53), and −1.09 (95% CI: −1.17 to −1.02), respectively (Table 1, Figure 1A,B). The all-age DALY cases were 5,318,837.66 (95% UI: 4,733,229.92 to 5,916,376.88), 2,568,854.39 (95% UI: 2,233,137.32 to 2,992,340.5), and 1,076,127.45 (95% UI: 889,738.88 to 1,303,328.41) years, respectively. The corresponding ASDRs (per 100,000 population) were 100.99 (95% UI: 89.92 to 112.21), 48.94 (95% UI: 42.61 to 57.02), and 20.49 (95% UI: 16.95 to 24.9) years, with the EAPCs from 1990 to 2021 of −1.55 (95% CI: −1.68 to −1.42), 0.14 (95% CI: 0.05 to 0.22), and −1.17 (95% CI: −1.29 to −1.04), respectively (Table 1, Figure 1C,D). The all-age incident cases were 357,665.50 (95% UI: 314,247.51 to 403,188.49), 149,451.98 (95% UI: 130,021.59 to 171,706.89), and 152,861.93 (95% UI: 125,810.13 to 185,975.36) cases, respectively. The corresponding ASIRs (per 100,000 population) were 6.84 (95% UI: 6.02 to 7.71), 2.90 (95% UI: 2.52 to 3.33), and 2.88 (95% UI: 2.38 to 3.51) cases, with the EAPCs from 1990 to 2021 of −0.43 (95% CI: −0.55 to −0.31), 0.54 (95% CI: 0.46 to 0.62), and 0.81 (95% CI: 0.67 to 0.95), respectively (Table 1, Figure 1E,F).

### 3.2. Geographical Distribution of Disease Burden

In addition to the overall age distribution, Asia presented significant regional variations in the age-specific gynecological cancer burden. In 2021, for cervical cancer, individuals aged 90 to 94 in Southeast Asia reported the highest age-specific mortality rate (Figure S1). Individuals aged 55 to 59 in South Asia reported the highest age-specific DALY rate and age-specific incidence rate (Figures S2 and S3). For ovarian cancer, individuals aged 95 plus in High-income Asia–Pacific reported the highest age-specific mortality rate (Figure S4), whereas individuals aged 65 to 69 in Central Asia reported the highest age-specific DALY rate, with the highest age-specific incidence rate reported in individuals aged 90 to 94 in high-income Asia–Pacific (Figures S5 and S6). For uterine cancer, individuals aged 95 plus in high-income Asia–Pacific presented the highest age-specific mortality rate (Figure S7). Individuals aged 70 to 74 in Central Asia reported the highest age-specific DALY rate and individuals aged 70 to 74 in Central Asia reported the highest age-specific incidence rate (Figures S8 and S9). Additionally, for the age-specific mortality rate, our results revealed that from 1980 to 2021, the age group most affected shifted from those aged 85 to 89 to those aged 95 and older (Table S1). For the age-specific DALY rate, from 1990 to 2021, the age group most affected was always those aged 55 to 59 (Table S2). For the age-specific incidence rate, the age group most affected shifted from those aged 85 to 89 to those aged 55 to 59 (Table S3).

In 2021, among the four world regions (Africa, America, Asia, and Europe), Asia reported the highest number of deaths, DALYs, and incident cases of 160,723.84 (95% UI: 143,073.14 to 179,396.56), 5,318,837.66 (95% UI: 4,733,229.92 to 5,916,376.88), and 357,665.5 (95% UI: 314,247.51 to 403,188.49), respectively (Table S4). Additionally, Asia had the third highest ASMR per 100,000 population for cervical cancer (3.10; 95% UI: 2.75 to 3.47) and the lowest ASMRs for ovarian cancer (1.64; 95% UI: 1.44 to 1.91) and uterine cancer (0.74; 95% UI: 0.62 to 0.91) (Table S5, Figure S10).

In 2021, for cervical cancer, South Asia had the highest burden, with an ASMR (per 100,000 population) of 4.42 (95% UI: 3.83 to 5.00) cases, followed by Southeast Asia (3.96; 95% UI: 3.42 to 4.56) and Central Asia (3.47; 95% UI: 3.09 to 3.90). High-income Asia–Pacific had the lowest burden, with an ASMR (per 100,000 population) of 1.32 (95% UI: 1.18 to 1.43) cases (Table 1). For ovarian cancer, Central Asia had the highest disease burden, with an ASMR (per 100,000 population) of 2.27 (95% UI: 2.00 to 2.58) cases, followed by Southeast Asia (2.11; 95% UI: 1.64 to 2.73) and South Asia at (2.00; 95% UI: 1.73 to 2.39). East Asia presented the lowest burden, with an ASMR (per 100,000 population) of (1.20; 95% UI: 0.89 to 1.55) cases. For uterine cancer, Central Asia still had the highest burden, with an ASMR (per 100,000 population) of 1.53 (95% UI: 1.37 to 1.71) cases, followed by Southeast Asia (1.09; 95% UI: 0.79 to 1.32) and high-income Asia–Pacific (0.88; 95% UI: 0.75 to 0.96). Conversely, South Asia had the lowest burden, with an ASMR (per 100,000 population) of 0.64 (95% UI: 0.53 to 0.85) cases (Table 1).

**Figure 2:**
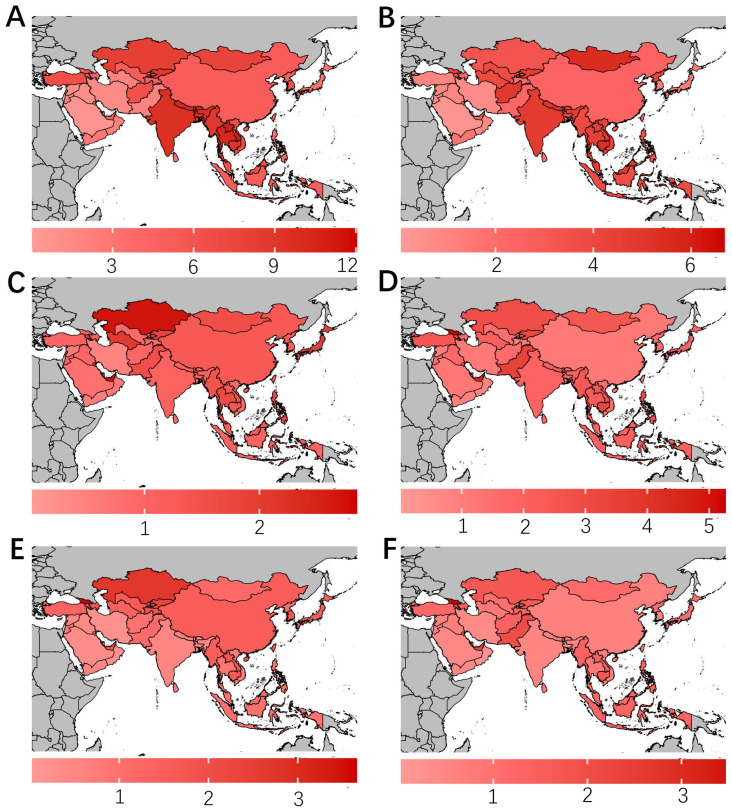
Age-standardized mortality rates attributable to cervical cancer in 1990 (**A**); to cervical cancer in 2021 (**B**); to ovarian cancer in 1990 (**C**); to ovarian cancer in 2021 (**D**); to uterine cancer in 1990 (**E**); and to uterine cancer in 2021 (**F**). Red in the map indicates a high disease burden, whereas blue indicates a low disease burden. DALY = disability-adjusted life year.

According to the EAPCs in the ASMR from 1980 to 2021, the burden of cervical and uterine cancer decreased across most Asian countries, whereas the burden of ovarian cancer increased across most Asian countries (Table S6). Across 49 countries and territories, for cervical cancer, Seychelles reported the highest disease burden in 1980 with an ASMR (per 100,000 population) of 12.08 (95% UI: 10.08 to 14.17) cases, followed by Lao People’s Democratic Republic (10.66; 95% UI: 7.34 to 15.13), Thailand (10.25; 95% UI: 7.65 to 12.56), and Bhutan (10.03; 95% UI: 6.09 to 15.04) (Figure 2A, Table S6). In 2021, Seychelles reported the highest ASMR (per 100,000 population) of cervical cancer of 6.68 (95% UI: 5.56 to 7.86) cases, followed by Mongolia (5.46; 95% UI: 4.03 to 7.18), Cambodia (5.36; 95% UI: 4.04 to 7.44), and Nepal (4.91: 95% UI: 3.44 to 6.56) (Figure 2B, Table S6). For ovarian cancer, Brunei Darussalam reported the highest ASMR (per 100,000 population) in 1980 of 2.86 (95% UI: 1.96 to 4.9) cases, followed by Bahrain (10.66; 95% UI: 7.34 to 15.13), Kazakhstan (10.25: 95% UI: 7.65 to 12.56), and the United Arab Emirates (2.68; 95% UI: 1.54 to 5.74) (Figure 2C, Table S6). In 2021, Georgia reported the highest ASMR (per 100,000 population) of 5.28 (95% UI: 4.47 to 6.11) cases, followed by the United Arab Emirates (4.41; 95% UI: 3.44 to 5.63), Seychelles (3.68; 95% UI: 2.88 to 4.45), and Brunei Darussalam (3.47; 95% UI: 2.7 to 4.24) (Figure 2D, Table S6). For uterine cancer, in 1980, Georgia reported the highest ASMR (per 100,000 population) of 3.66 (95% UI: 3.13 to 4.19) cases, followed by Kazakhstan (2.69; 95% UI: 2.41 to 3.02), Mauritius (2.42; 95% UI: 2.24 to 2.63), and Kyrgyzstan (2.24; 95% UI: 1.79 to 2.78) (Figure 2E, Table S6). In 2021, Georgia still had the highest ASMR (per 100,000 population) of 3.67 (95% UI: 3.11 to 4.32) cases, followed by Armenia (2.33; 95% UI: 1.97 to 2.8), Mauritius (2.27; 95% UI: 2.05 to 2.44), and the United Arab Emirates (2.24; 95% UI: 1.62 to 3.07) (Figure 2F, Table S6).

### 3.3. Correlation Analysis for SDI Levels and Disease Burden

**Figure 3:**
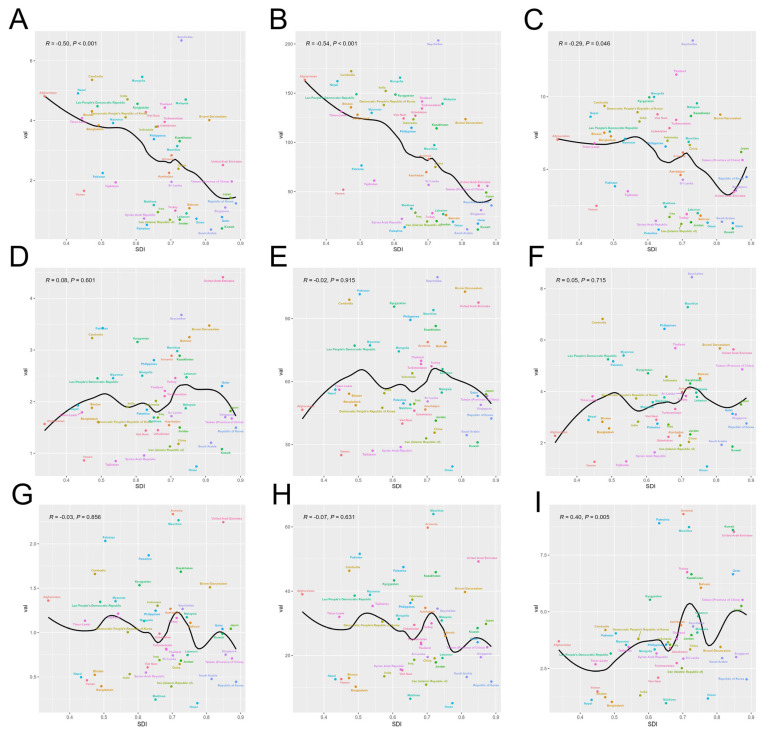
Age-standardized rates for different SDI levels by countries in 2021 of mortality rate of cervical cancer (**A**); of DALY rate of cervical cancer (**B**); of incidence rate of cervical cancer (**C**); of mortality rate of ovarian cancer (**D**); of DALY rate of ovarian cancer (**E**); of incidence rate of ovarian cancer (**F**); of mortality rate of uterine cancer (**G**); of DALY rate of uterine cancer (**H**); and of incidence rate of uterine cancer (**I**); The solid black line is the fit curve. SDI = sociodemographic index. The different colors represent different countries. GBD = Global Burden of Disease Study.

We performed Pearson correlation analyses to examine the relationships between the SDI levels and the ASMR, ASDR, and ASIR across 49 Asian countries. For cervical cancer, our findings revealed negative correlations between disease burden and the SDI, indicating that countries with higher SDIs such as Japan, Qatar, and Singapore generally experienced lower disease burdens. Specifically, the r values for the ASMR, ASDR, and ASIR were −0.49, −0.53, and −0.27, respectively. The *p* values confirmed significant correlations for the ASMR and ASDR with the SDI, whereas the correlation for the ASIR was not significant (Figure 3A–C). For ovarian cancer, the correlation between disease burden and SDI showed an approximate M-shape, with higher burdens observed in countries with high-middle SDI values, such as Georgia, and with low-middle SDI values, such as Pakistan. The *p* values indicated no significant correlations across the measures (Figure 3D–F). For uterine cancer, countries with a high-middle SDI such as Georgia and Mauritius presented greater disease burdens, whereas those with high or low SDI levels were less affected. Additionally, the incidence rate showed a slight positive correlation with the SDI, as evidenced by an r value of 0.38 (Figure 3G–I).

### 3.4. Decomposition Analysis of the Factors Influencing Disease Burden

The impacts of aging, population, and epidemiological changes in the disease burden of gynecological cancers in Asia from 1990 to 2021 were explored via decomposition analysis. Our findings indicated that the population was the leading driver of the cervical cancer burden, increasing the DALYs by 160.34%, and that aging contributed to 75.1% increase. Conversely, epidemiological changes mitigated the ASDR, contributing to a −135.44% reduction across Asia (Table S7, Figure S11A). For ovarian cancer, all three factors contributed to the increase in DALYs, with population, aging, and epidemiological changes leading to 56.76%, 31.88%, and 11.36% increases, respectively. However, in specific locations in Asia, epidemiological changes have demonstrated weakening effects in East Asia and high-income Asia–Pacific, decreasing DALYs by −14.5% and −29.07%, respectively (Table S7, Figure S11B). For uterine cancer, a similar trend to that of cervical cancer was observed, with epidemiological changes reducing the DALYs by −54.58% in Asia. The population was still the leading driver, contributing 95.29% to the increase in DALYs, and aging contributed to 59.29% of the increase (Table S7, Figure S11C).

### 3.5. Temporal Trend of the Global Disease Burden from 1990 to 2021

**Figure 4:**
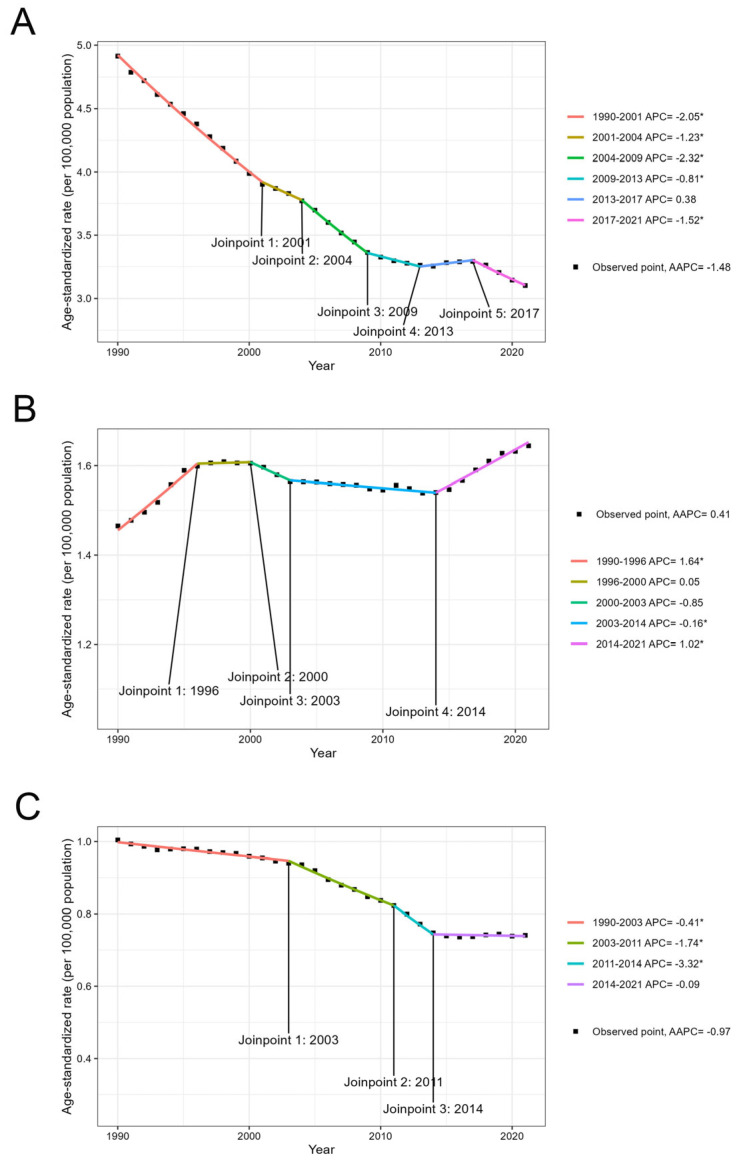
Trends in the age-standardized DALY rates from 1990 to 2021 in Asia attributable to cervical cancer (**A**); ovarian cancer (**B**); and uterine cancer (**C**). DALY = disability-adjusted life year. * indicates that the trend is statistically significant. APC = annual percent change.

We applied joinpoint analysis to determine the temporal trends in the disease burden of gynecological cancers in Asia from 1990 to 2021. For cervical cancer, the ASMR initially decreased significantly from 1990 to 2013 at first, followed by a brief period of increase from 2013 to 2017, and then a significant decline from 2017 to 2021. The AAPC was estimated to be −1.48 from 1990 to 2021, indicating an overall decreasing trend (Figure 4A). For ovarian cancer, the ASMR initially increased significantly from 1990 to 1996, followed by a stable period from 1996 to 2000. A decrease was subsequently observed from 2000 to 2014. The rate subsequently began to increase from 2014 to 2021. The AAPC was estimated to be 0.41 (Figure 4B). For uterine cancer, the ASMR decreased significantly from 1990 to 2014. However, a stable period was observed from 2014 to 2021. The AAPC was estimated to be −0.97, indicating a decreasing trend over the study period (Figure 4C).

The APC-Web tool was employed to explore the age-specific trends in the disease burden of gynecological cancers over the past 30 years. For patients with cervical cancer, the number of deaths tended to decrease across most age groups. The lowest local drift was observed in individuals aged 15 to 19 of −2.17 (95% CI: −2.92 to −1.43) (Figure S12A), and the net drift was estimated to be −1.39 (95% CI: −1.55 to −1.23) (Table S7). For ovarian cancer, the number of deaths increased across most age groups, with the highest local drift observed in individuals aged 95 plus of 1.30 (95% CI: −0.85 to 3.49) (Figure S12B), and the net drift was estimated to be 0.17 (95% CI: 0.08 to 0.26) (Table S7). The number of deaths from uterine cancer also tended to decrease in most age groups, with the lowest local drift observed in individuals aged 30 to 34 of −1.96 (95% CI: −2.16 to −1.76) (Figure S12C), and the net drift was estimated to be −1.26 (95% CI: −1.36 to −1.17) (Table S8). The trends of age patterns varied across different GBD regions in Asia. For example, in East Asia, the number of deaths from all three gynecological cancers has decreased across all age groups in the last 30 years. Additionally, in the High-income Asia–Pacific, the number of deaths from cervical and ovarian cancers decreased across most age groups, whereas the number of deaths from uterine cancer tended to increase among younger individuals and decreased among older individuals (Table S8, Figure S13).

Significant impacts of period effects on disease burden were observed after controlling for age and birth cohort effects. For cervical cancer, the period rate ratio (Period RR) remained below 1, indicating that mortality rates across different GBD regions in Asia consistently decreased over the study period (Table S8, Figure S12D). For ovarian cancer, the Period RR in Asia slightly exceeded 1, suggesting a modest increase in deaths. Notably, South Asia presented the highest increasing trend, whereas East Asia presented the highest decreasing trend (Table S8, Figure S12E). For uterine cancer, the Period RR in Asia indicated a decrease in deaths over time. Specifically, East Asia presented the highest decreasing trend (Table S8, Figure S12F).

After controlling for age and period effects, birth cohort effects had a significant effect on the risk ratio of cancer. For cervical cancer, South Asia had the highest initial risk ratio, but all regions converged to approximately 1.0 in recent cohorts, indicating a significant risk reduction over time (Table S8, Figure S12G). For ovarian cancer, East Asia had the highest decreasing trend in risk, with the risk ratio decreasing from 2.5 in the earliest cohorts to less than 1.0 in recent cohorts. In contrast, South Asia showed the highest increasing trend in risk, with the risk ratios rising above 1.5 in recent cohorts (Table S8, Figure S12H). For uterine cancer, East Asia still showed the highest decreasing trend of risk, with risk ratio decreasing from over 5.0 in the earliest cohorts to 1.0 in recent cohorts. High-income Asia–Pacific, South Asia, and Southeast Asia maintained stable and low risk ratios of approximately 1.0 (Table S8, Figure S12I).

### 3.6. The Predicted Results from 2025 to 2050

**Figure 5:**
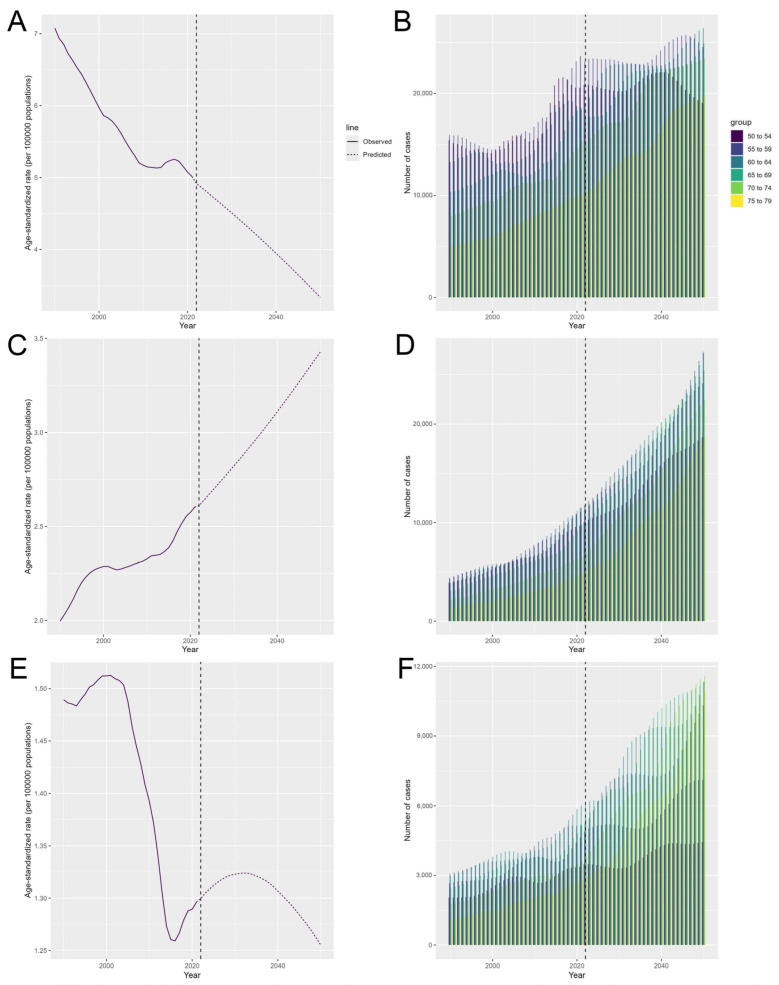
The predicted results from 1990 to 2050 by age group according to the BAPC model for age-standardized mortality rate of cervical cancer (**A**); age-specific death cases of cervical cancer (**B**); age-standardized mortality rate of ovarian cancer (**C**); age-specific death cases of ovarian cancer (**D**); age-standardized mortality rate of uterine cancer (**E**); age-specific death cases of uterine cancer (**F**) in Asia. The part behind the black vertical line is the predicted value. BAPC = Bayesian age-period-cohort.

Based on our BAPC prediction model, for cervical cancer, projections indicated a consistent decline in disease burden from 2025 to 2050. The estimated ASMR (per 100,000 population) was projected to decrease from 4.77 (95% CI: 4.39 to 5.14) cases in 2025 to 3.33 (95% CI: 0.09 to 6.57) cases in 2050 (Figure 5A, Table S9). For ovarian cancer, the ASMR (per 100,000 population) was projected to increase from 2.69 (95% CI: 2.51 to 2.87) cases in 2025 to 3.43 (95% CI: 0.37 to 6.49) cases in 2050 (Figure 5C, Table S9). For uterine cancer, the ASMR (per 100,000 population) was projected to peak in 2032 of 1.32 (95% CI: 0.90 to 1.75) cases, then the rate (per 100,000 population) was projected to decrease to 1.26 (95% CI: −0.32 to 2.83) cases in 2050 (Figure 5E, Table S9).

The projections also indicated variability in age-specific deaths, with an overall increasing trend by 2050. In 2050, for cervical cancer, the greatest number of age-specific deaths were projected to occur in individuals aged 60 to 64, with 26,404.07 (95% CI: 0.00 to 55,125.94) deaths, followed closely by individuals aged 65 to 69 (24,887.51; 95% CI: 0.00 to 51,958.49) (Figure 5B, Table S10). Among those with ovarian cancer, individuals aged 60 to 64 were expected to die the most (27,327.08; 95% CI: 920.43 to 53,733.73), followed by individuals aged 65 to 69 (27,170.54; 95% CI: 917.13 to 53,423.95) (Figure 5D, Table S10). For uterine cancer, the greatest number of deaths were projected to occur in individuals aged 75 to 79 (11,610.70; 95% CI: 0.00 to 28,997.96), followed by individuals aged 70 to 74 (11,356.44; 95% CI: 0.00 to 28,363.14) (Figure 5F, Table S10).

## 4. Discussion

Since the beginning of the 21st century, gynecological cancers have consistently posed a significant threat to public health worldwide. Previous studies have assessed the global disease burden of cervical, ovarian, and uterine cancer; however, these studies primarily relied to outdated data and lacked a focus on Asia [10,14]. To the best of our knowledge, our study provides the latest comprehensive assessment of the disease burden of gynecological cancers in Asia, spanning from 1980 to 2021, and projects future trends up to 2050. These findings offer policymakers timely evidence-based insights through detailed epidemiological assessments and predictive warnings, which will facilitate the development of targeted strategies to mitigate the disease burden and improve women’s health outcomes across Asian populations.

In this study, we found that cervical cancer posed the highest burden across Asia, followed by ovarian cancer and uterine cancer. South Asia has the highest disease burden of cervical cancer, and Central Asia has the highest disease burden of ovarian and uterine cancers. Negative correlations were revealed between the disease burden of cervical cancer and the SDI, whereas the correlations for the other two cancers were not that significant. In addition, population and aging emerged as drivers of the disease burden across all three gynecological cancers, whereas epidemiological changes had a diminishing impact on the cervical and uterine cancer burdens. Over the study period, for cervical cancer, the ASMR, ASDR, and ASIR all showed decreasing trends; for ovarian cancer, these indicators all showed an overall increasing trend; for uterine cancer, the ASMR and ASDR showed decreasing trends, and the ASIR showed an increasing trend. The APC-Web tool further confirmed decreasing trends in cervical and uterine cancer burdens for most age groups over the past three decades. Finally, our projections indicate that the number of deaths from gynecological cancers in Asia will increase over the next 25 years, particularly affecting individuals aged 60 to 64.

### 4.1. Cervical Cancer

From 1980 to 2021, cervical cancer was the predominant contributor to the burden of gynecological cancer in Asia. Our findings showed that cervical cancer was highly prevalent across Asia, which was supported by previous research [15]. Inadequate human papillomavirus (HPV) vaccination coverage in Asia could be a significant contributor to the high incidence of cervical cancer across this region. Research has shown that persistent infection with high-risk, carcinogenic HPV, which is transmitted predominantly through sexual contact, affects nearly all cervical cancer patients [16]. A high prevalence of high-risk HPV has been reported across Central Asia and the Middle East [17]. In less developed areas within Asia, various challenges including high costs associated with vaccines, limited access to healthcare services, and logistical difficulties in implementing vaccination programs, have contributed to this severe disease burden. For example, in China, despite the availability of bivalent, quadrivalent, and nine-valent vaccines, coverage rates remain suboptimal. The reason might be attributed to the exclusion of HPV vaccines from the National Immunization Program [18]. Additionally, high population density among many Asian countries may exacerbate the prevalence of sexually transmitted infections. Du et al. demonstrated that dense populations contributed to the rapid spread of infections, facilitated by closer human contact and frequently overburdened healthcare infrastructure [19].

Furthermore, we revealed a decreasing trend in ASMR, ASDR, and ASIR of cervical cancer over the study period. The establishment of national screening programs has played an essential role in lowering the disease burden, primarily through modalities such as cervical cytology, colposcopy, and cervical conization [20]. Research has indicated that screening programs implemented in Japan have effectively reduced the incidence of invasive cervical cancer [21]. In addition, the WHO has updated its guidelines to advance the screening of cervical cancer, recommending HPV DNA testing as the preferred screening method over visual inspection with acetic acid (VIA) or cytology (Pap smear) [22].

Our Pearson correlation analysis revealed a negative relationship between the SDI and the disease burden of cervical cancer, indicating that the low-SDI regions presented the highest disease burden. Other researchers have noted this phenomenon, where the mortality rate in lower middle-income countries is three times higher than that in high-income countries [23]. The observed negative correlation may be attributable to the prioritized control measures and prevention efforts implemented in regions with high SDIs. For example, Japan implemented a nationwide policy providing free access to cervical cancer screenings through a voucher system [24]. In addition, the National Cancer Screening Program (NCSP) was launched in Korea to support cervical cancer prevention, early detection, diagnosis, treatment, and palliative care [25]. However, countries and regions with low SDI levels tend to neglect the implementation of relevant protection policies, leading to a particularly high disease burden. We advocate for the adoption of targeted policies tailored to regions with varying SDI levels across Asia.

### 4.2. Ovarian Cancer

Our study revealed a high disease burden attributable to ovarian cancer across Asia. This significant burden has been previously reported in other studies [26]. Specific recessive gene mutations, which are particularly common among Asian ethnicities could be a primary risk factor [27]. Previous studies have demonstrated the correlation between *BRCA* mutations and ovarian cancer [28]. One study involving Chinese, Korean, Japanese, and Indian populations revealed that more than half of the *BRCA* mutations were unique to Asian ethnic groups, highlighting significant ethnic-specific variations [29]. A case–control study further confirmed this finding in Asian, revealing that approximately 15.8% of ovarian cancer patients harbored mutations in the *BRCA1* or *BRCA2* genes in Pakistani countries [30]. The low use of oral contraceptive pills (OCPs) has also been identified as a risk factor for ovarian cancer in Asia. The use of OCPs has been proven to decrease the incidence of epithelial ovarian cancer, while studies have shown that the adoption of OCPs in South, Central, and Southeast Asia has remained below the global average [31]. In addition, Tabassum et al. demonstrated that factors such as changes in dietary habits, reduced physical activity, obesity, delayed pregnancies, and decreased breastfeeding contribute to the high ASMR of ovarian cancer [32].

The results revealed increasing trends in ASMR, ASDR, and ASIR for ovarian cancer across Asia, which were previously reported in other studies [33]. Aging is considered a key factor contributing to the increasing disease burden. The incidence of ovarian cancer peaks post-menopause, with the risk increasing significantly as individuals age. As life expectancy increases, the proportion of elderly individuals increases, resulting in more cases of ovarian cancer among elderly individuals [34]. Furthermore, shifts in reproductive lifestyle factors may also contribute to this trend. A recent extensive birth cohort study comparing Chinese women born in the 1930s and 1970s revealed a decrease of 1.8 years in the age at menarche, an increase of 6 years in the age at first birth, a decrease of 4–5 months in breastfeeding duration, and an increase of 1.4 years in the age at menopause [35]. These changes are linked to more ovulatory cycles, resulting in a high incidence of ovarian cancer. Notably, individuals carrying BRCA1/2 mutations face a significantly elevated risk of developing ovarian cancer [36]. Consequently, current research recommends that BRCA1 mutation carriers undergo risk-reducing bilateral salpingo-oophorectomy (RRBSO) between ages 35–40 upon completion of childbearing, while BRCA2 mutation carriers are advised to undergo the same procedure between ages 40–45 [37].

### 4.3. Uterine Cancer

Uterine cancer contributed the least but still significantly to the gynecological cancer burden in Asia, with the ASIR showing an increasing trend. Economic development could contribute to this trend. Economic development can lead to increased obesity rates and different lifestyles, both of which are risk factors for uterine cancer. A previous study reported a strong correlation between elevated body mass index (BMI) and uterine cancer, and obesity was considered the primary contributor to high BMI [38]. Moreover, high BMI can influence age at menarche and menopause to induce excessive estrogen and progesterone levels, with endometrial cells proliferating due to excess estrogen, which is considered a risk factor for endometrial cancer. Additionally, the westernization of lifestyles, including increased consumption of processed foods and sedentary behaviors, has led to an increased prevalence of metabolic conditions such as diabetes. Diabetes can contribute to the onset of endometrial cancer through elevated insulin and insulin-like growth factor (IGF) levels, stimulating endometrial cell growth and hormonal imbalances [39].

Our study revealed a decreasing trend in the disease burden of uterine cancer across Asia. The results were confirmed by a study based on the WHO mortality database and National Cancer Incidence Databases [40]. Innovations in drugs for the treatment of endometrial cancer have contributed to reducing the disease burden. Research has focused on combining drugs such as metformin with progestin to improve treatment efficacy in preventing the progression of endometrial cancer [41]. Additionally, new approaches for the clinical treatment of endometrial cancer have significantly reduced the disease burden. A population-based study in Japan demonstrated a greater therapeutic effect of minimally invasive surgery than traditional open surgery in Japanese patients with early-stage endometrial cancer [42]. Furthermore, the application of molecular markers in risk stratification has provided novel insights into personalized treatment, effectively reducing the disease burden [43]. To further reduce the disease burden of uterine cancer, continuing efforts to enhance early detection, promote innovative treatment approaches, and ensure equitable access to these advancements across all populations in Asia is crucial.

### 4.4. Future Trends

According to our BAPC prediction model, the number of deaths attributable to gynecological cancers in Asia is expected to increase, particularly among individuals aged 60 to 64. This trend has also been predicted by the WHO [15]. In November 2020, the WHO launched a global strategy to eradicate cervical cancer by reducing its incidence to fewer than four cases per 100,000 women per year by 2120. More efforts are being made in Asia to reduce the burden of gynecological cancer. In Asia, the strategic framework of the Western Pacific Region, developed with member states, supported the WHO goal by integrating global and regional health initiatives, focusing on adapting strategies to local needs, and strengthening national cervical cancer programs to meet WHO targets [2]. Recently, the East Asian Gynecologic Oncology Trial Group (EAGOT) was established as a platform to develop standards of care and formulate guidelines for Asian women affected by gynecologic cancers [44]. Although various measures have been implemented to reduce the disease burden, targeted actions by governments are still needed.

### 4.5. Limitations

We recognize the limitations of our study. The reliance on data from the GBD 2021 database restricted our access to more detailed data at the county, provincial, and state levels. For example, in China, the database offered only aggregated annual figures on disease burden, preventing more comprehensive investigations. Additionally, an overemphasis on *p* values in the study might overlook the clinical significance of the findings, suggesting a need for a more diverse analytical approach to fully understand the implications of the results. Furthermore, statistical reliability may have been compromised in economically underdeveloped regions, where robust data collection and implementation might be lacking. Finally, our predictive models might not include all variables influencing changes in disease burden, possibly leading to inaccuracies in our projections.

## 5. Conclusions

In conclusion, from 1980 to 2021, gynecological cancers significantly contributed to the disease burden across Asia. This health issue underscores the need for more advanced screening methods and personalized policies tailored to women in Asia. Our study offers evidence-based recommendations to optimize medical resource allocation, enhance early detection of cervical and uterine cancers, and implement prophylactic surgical interventions for high-risk ovarian cancer patients.

## Figures and Tables

**Table 1:** The all-age deaths, DALYs, incident cases, ASMR, ASDR, and ASIR per 100,000 population attributable to gynecological cancers in 1980/1990 and 2021 and the EAPCs from 1980 to 2021 by GBD region in Asia.

Location	1980/1990		2021		EAPC from 1980 to 2021 ^b^
	Counts ^a^	ASR ^a^	Counts ^a^	ASR ^a^	
**Deaths**					
**Cervical cancer**					
Asia	105,469.25 (91,934.2–124,165.45)	6.10 (5.36–7.16)	160,723.84 (143,073.14–179,396.56)	3.10 (2.75–3.47)	−1.68
					(−1.77–−1.59)
Central Asia	2614.76 (2465.78–2765.99)	6.35 (5.96–6.73)	3093.38 (2734.71–3497.22)	3.47 (3.09–3.90)	−1.43
					(−1.54–−1.31)
East Asia	35,015.35 (27,246.63–43,843.21)	5.00 (3.93–6.20)	52,032.08 (39,399.83–66,509.9)	2.41 (1.83–3.07)	−1.58
					(−1.74–−1.41)
High-income Asia–Pacific	5652.76 (5283.89–6114.84)	3.74 (3.48–4.03)	5074.08 (4303.28–5602.42)	1.32 (1.18–1.43)	−2.37
					(−2.53–−2.22)
South Asia	46,998.03 (37,972.51–58,456.86)	8.79 (7.04–10.98)	70,314.60 (61,026.36–79,858.14)	4.42 (3.83–5.00)	−1.92
					(−2.09–−1.75)
Southeast Asia	13,923.40 (11,203.51–17,143.76)	6.25 (5.09–7.70)	27,513.07 (23,668.86–31,860.21)	3.96 (3.42–4.56)	−1.09
					(−1.17–−1.00)
**Ovarian cancer**					
Asia	21,138.66 (16,819.56–28,334.13)	1.31 (1.05–1.74)	84,278.89 (73,874.6–97,734.25)	1.64 (1.44–1.91)	0.43
					(0.33–0.53)
Central Asia	644.84 (560.24–736.9)	1.57 (1.36–1.80)	1949.03 (1705.34–2219.78)	2.27 (2.00–2.58)	1.15
					(1.06–1.25)
East Asia	8746.25 (6158.32–13,134.29)	1.28 (0.93–1.87)	26,337.19 (19,516.44–34,082.62)	1.20 (0.89–1.55)	−0.46
					(−0.62–−0.30)
High-income Asia–Pacific	2803.27 (2678.11–2974.64)	1.81 (1.72–1.92)	7200.86 (6064.30–7968.63)	1.72 (1.51–1.85)	−0.25
					(−0.38–−0.11)
South Asia	5207.88 (3507.82–7227.2)	1.11 (0.72–1.54)	30,585.13 (26,424.78–36,402.65)	2.00 (1.73–2.39)	1.49
					(1.43–1.55)
Southeast Asia	2564.91 (1851.37–3818)	1.22 (0.89–1.76)	14,523.22 (11,286.29–18,865.47)	2.11 (1.64–2.73)	1.33
					(1.23–1.42)
**Uterine cancer**					
Asia	17,792.51 (12,812.07–22,268.12)	1.17 (0.87–1.43)	37,508.92 (31,519.05–45,758.59)	0.74 (0.62–0.91)	−1.09
					(−1.17–−1.02)
Central Asia	876.77 (817.83–937.34)	2.24 (2.08–2.40)	1243.44 (1105.49–1403.90)	1.53 (1.37–1.71)	−1.14
					(−1.32–−0.96)
East Asia	10,011.71 (6213.31–13,815.56)	1.51 (0.98–2.03)	14,233.24 (10,580.48–19,159.53)	0.65 (0.48–0.88)	−2.07
					(−2.29–−1.85)
High-income Asia–Pacific	2251.53 (2047.66–2450.62)	1.58 (1.44–1.71)	3936.83 (3194.38–4379.49)	0.88 (0.75–0.96)	−1.17
					(−1.43–−0.92)
South Asia	2447.90 (1812.16–3015.68)	0.58 (0.43–0.71)	9236.53 (7688.44–12,339.17)	0.64 (0.53–0.85)	0.18
					(0.10–0.26)
Southeast Asia	1949.66 (1395.71–2557.88)	1.00 (0.73–1.29)	7378.74 (5306.59–9002.82)	1.09 (0.79–1.32)	0.21
					(0.12–0.30)
**DALY**					
**Cervical cancer**					
Asia	4,049,060.64 (3,572,517.1–4,543,446.09)	163.87 (144.52–183.89)	5,318,837.66 (4,733,229.92–5,916,376.88)	100.99 (89.92–112.21)	−1.55
					(−1.68–−1.42)
Central Asia	89,741.98 (86,415.34–94,113.3)	174.67 (167.79–183.27)	108,285.23 (94,717.7–12,2647.14)	113.66 (99.9–128.44)	−1.13
					(−1.28–−0.98)
East Asia	1,178,714.01 (958,919.39–1,452,779.21)	114.35 (93.56–140.57)	1,616,240.23 (1,195,414.47–2,080,056.88)	75.79 (56.14–97.92)	−1.06
					(−1.2–−0.92)
High-income Asia–Pacific	144,581.72 (135,082.87–155,151.29)	70.72 (66.01–75.87)	132,808.21 (119,523.11–144,704)	44.07 (40.95–47.42)	−1.43
					(−1.52–−1.34)
South Asia	1,967,172.06 (1,640,297.67–2,286,747.31)	261.61 (216.39–304.23)	2,432,061.02 (2,097,849.72–2,773,134.49)	143.24 (123.88–162.82)	−2.04
					(−2.33–−1.75)
Southeast Asia	613,110.37 (526,601.48–699,789.93)	188.24 (162.71–214.67)	931,500.99 (800,256.95–1,086,328.05)	125.03 (107.37–145.35)	−1.49
					(−1.60–−1.39)
**Ovarian cancer**					
Asia	1,036,435.48 (844,239.2–1,272,438.2)	43.73 (35.98–53.57)	2,568,854.39 (2,233,137.32–2,992,340.5)	48.94 (42.61–57.02)	0.14
					(0.05–0.22)
Central Asia	25,758.05 (23,408.19–28,171.07)	50.44 (45.77–55.24)	61,881.11 (53,807.39–70,556.41)	67.03 (58.39–76.28)	1
					(0.85–1.14)
East Asia	416,565.35 (301,040.22–542,960.91)	40.88 (30.43–52.84)	787,095.89 (580,908.59–1,024,657.54)	36.19 (26.75–47.23)	−0.86
					(−1.02–−0.70)
High-income Asia–Pacific	128,562.40 (123,646.42–132,898.81)	62.41 (59.95–64.54)	168,116.95 (148,704.1–180,427.51)	50.30 (45.19–53.24)	−0.74
					(−0.81–−0.67)
South Asia	264,386.13 (200,200.75–344,157.49)	37.70 (28.43–48.66)	960,207.82 (822,850.63–1,142,385.68)	58.40 (50.28–69.55)	1.3
					(1.22–1.38)
Southeast Asia	150,719.00 (119,298.73–213,939.65)	47.58 (38.24–65.85)	484,774.21 (368,984.4–633,791.17)	65.65 (50.15–85.54)	0.97
					(0.89–1.05)
**Uterine cancer**					
Asia	626,813.16 (462,084.19–746,542.48)	27.70 (20.87–32.64)	1,076,127.45 (889,738.88–1,303,328.41)	20.49 (16.95–24.90)	−1.17
					(−1.29–−1.04)
Central Asia	29,872.56 (28,015.68–31,657.86)	60.86 (56.99–64.51)	36,940.92 (32,386.42–42,248.7)	41.59 (36.74–47.29)	−1.23
					(−1.49–−0.97)
East Asia	353,039.19 (232,773.4–446,225.4)	35.73 (24.38–44.70)	425,141.95 (320,034.77–571,293.09)	19.36 (14.58–26.02)	−2.33
					(−2.65–−2.02)
High-income Asia–Pacific	53,335.23 (47,425.64–58,592.43)	26.02 (23.17–28.54)	88,362.34 (77,557.35–96,560.38)	24.29 (21.84–26.27)	0.08
					(−0.10–0.26)
South Asia	94,296.04 (75,543.98–112,766.55)	14.96 (11.94–17.89)	256,842.42 (214,336.05–344,464.65)	16.42 (13.72–21.96)	0.12
					(−0.02–0.26)
Southeast Asia	89,095.68 (61,687.2–108,414.93)	30.49 (21.6–37.07)	226,681.94 (156,600.79–279,317.23)	31.22 (21.81–38.21)	−0.06
					(−0.15–0.03)
**Incidence**					
**Cervical cancer**					
Asia	196,998.33 (175,157.37–218,833.15)	8.06 (7.17–8.95)	357,665.5 (314,247.51–403,188.49)	6.84 (6.02–7.71)	−0.43
					(−0.55–−0.31)
Central Asia	5168.85 (4951.55–5420.21)	9.97 (9.54–10.46)	7178.83 (6265.57–8081.45)	7.50 (6.56–8.41)	−0.54
					(−0.71–−0.38)
East Asia	61,909.14 (50,407.48–76,000.79)	6.03 (4.95–7.37)	137,863.79 (101,144.48–177,754.73)	6.69 (4.93–8.67)	0.76
					(0.6–0.93)
High-income Asia–Pacific	12,720.39 (12,005.79–13,515.63)	6.30 (5.94–6.68)	15,577.84 (14,044.14–16,881.51)	5.52 (5.12–5.97)	−0.18
					(−0.29–−0.07)
South Asia	83,506.85 (69,576.79–96,968.91)	11.24 (9.26–13.04)	132,481.99 (114,540.70–151,294.70)	7.79 (6.75–8.87)	−1.28
					(−1.59–−0.97)
Southeast Asia	30,364.63 (26,327.24–34,797.64)	9.39 (8.12–10.74)	58,017.08 (49,190.88–67,747.07)	7.84 (6.68–9.13)	−0.8
					(−0.9–−0.69)
**Ovarian cancer**					
Asia	54,459.70 (44,299.59–670,05.9)	2.30 (1.89–2.81)	149,451.98 (130,021.59–171,706.89)	2.90 (2.52–3.33)	0.54
					(0.46–0.62)
Central Asia	1219.94 (1112.09–1337.15)	2.39 (2.17–2.61)	2998.96 (2602.07–3418.89)	3.30 (2.88–3.76)	1.15
					(1.00–1.30)
East Asia	21,065.43 (15,069.18–27,403.26)	2.06 (1.54–2.65)	44,239.91 (33,123.68–57,358.78)	2.11 (1.59–2.74)	−0.32
					(−0.46–−0.18)
High-income Asia–Pacific	6798.13 (6490.41–7078.81)	3.36 (3.20–3.50)	11,227.65 (9782.40–12,235.43)	3.26 (2.9–3.47)	−0.15
					(−0.31–0.00)
South Asia	12,511.24 (9361.05–16,327.09)	1.79 (1.33–2.31)	49,664.47 (42,260.25–59,355)	3.02 (2.59–3.60)	1.57
					(1.49–1.65)
Southeast Asia	10,046.56 (7838.03–14,358.26)	3.05 (2.43–4.23)	35,031.98 (26,271.21–45,639.37)	4.80 (3.61–6.22)	1.35
					(1.24–1.46)
**Uterine cancer**					
Asia	49,559.48 (37,592.10–58,190.73)	2.22 (1.71–2.58)	152,861.93 (125,810.13–185,975.36)	2.88 (2.38–3.51)	0.81
					(0.67–0.95)
Central Asia	2930.84 (2758.52–3105.93)	5.93 (5.58–6.28)	4813.37 (4249.72–5449.13)	5.33 (4.72–6.00)	−0.3
					(−0.61–0.02)
East Asia	27,189.89 (18,791.97–341,44.00)	2.80 (1.97–3.48)	75,543.72 (56,833.53–103,404.09)	3.39 (2.54–4.64)	0.52
					(0.22–0.83)
High-income Asia–Pacific	4826.00 (4375.92–5235.28)	2.35 (2.13–2.55)	13,824.55 (12,300.83–15,052.24)	4.21 (3.82–4.57)	2.24
					(2.12–2.37)
South Asia	6014.57 (4769.73–7122.42)	0.97 (0.77–1.15)	23,668.16 (19,817.78–31,544.04)	1.52 (1.27–2.02)	1.26
					(1.07–1.45)
Southeast Asia	7221.10 (5143.54–8697.35)	2.47 (1.79–2.96)	25,169.01 (17,367.13–30,769.27)	3.43 (2.39–4.17)	0.91
					(0.85–0.96)

^a^ represents data in parentheses is the 95% uncertainty interval; ^b^ represents data in parentheses is the 95% confidence interval.

## Data Availability

The original data are openly available in Global Health Data Exchange (GHDx): https://ghdx.healthdata.org/ (accessed on 22 February 2025).

## Supplementary Materials

The following supporting information can be downloaded at: https://www.mdpi.com/article/10.3390/curroncol32060298/s1, References [45,46,47,48,49] are cited in the Supplementary Materials. Figure S1: Age-specific mortality rates per 100,000 population for cervical cancer by GBD region and age group in 1990 (A); in 2021 (B); Figure S2: Age-specific DALY rates per 100,000 population for cervical cancer by GBD region and age group in 1990 (A); in 2021 (B); Figure S3: Age-specific incidence rates per 100,000 population for cervical cancer by GBD region and age group in 1990 (A); in 2021 (B); Figure S4: Age-specific mortality rates per 100,000 population for ovarian cancer by GBD region and age group in 1990 (A); in 2021 (B); Figure S5: Age-specific DALY rates per 100,000 population for ovarian cancer by GBD region and age group in 1990 (A); in 2021 (B); Figure S6: Age-specific incidence rates per 100,000 population for ovarian cancer by GBD region and age group in 1990 (A); in 2021 (B); Figure S7: Age-specific mortality rates per 100,000 population for uterine cancer by GBD region and age group in 1990 (A); in 2021 (B); Figure S8: Age-specific DALY rates per 100,000 population for uterine cancer by GBD region and age group in 1990 (A); in 2021 (B); Figure S9: Age-specific incidence rates per 100,000 population for uterine cancer by GBD region and age group in 1990 (A); in 2021 (B); Figure S10: Age-standardized mortality rates in 2021 attributable to cervical cancer (A); to ovarian cancer (B); to uterine cancer (C); Figure S11: Effects of the aging, population, and epidemiological change on the DALYs by GBD region from 1990 to 2021 in Asia attributable to cervical cancer (A); to ovarian cancer (B); to uterine cancer (C). The black dots represent the overall difference in disease burden from 1990 to 2021; Figure S12: Age-period-cohort related trends in DALYs from 1992 to 2021 by GBD region attributable to cervical cancer of age effect (A), period effect (D), and cohort effect (G); attributable to ovarian cancer of age effect (B), period effect (E), and cohort effect (H); attributable to uterine cancer of age effect (C), period effect (F), and cohort effect (I). Each vertical row represents one gynecological cancer. DALY = disability-adjusted life year. GBD = Global Burden of Disease Study; Figure S13: Annual change per year of age-specific DALY rate of gynecological cancers for different age groups from 1992 to 2021 in Asia. Table S1: Age-specific deaths and age-specific mortality rate per 100,000 population in Asia attributable to gynecological cancers in 1980 and 2021 and the EAPCs of mortality rate from 1980 to 2021; Table S2: Age-specific DALYs and age-specific DALY rate per 100,000 population in Asia attributable to gynecological cancers in 1990 and 2021 and the EAPC of DALY rate from 1990 to 2021; Table S3: Age-specific incident cases and age-specific incidence rate per 100,000 population in Asia attributable to gynecological cancers in 1990 and 2021 and the EAPC of incidence rate from 1990 to 2021; Table S4: The global all-age deaths, DALYs, and incident cases attributable to gynecological cancers in 2021 by four world regions; Table S5: The global ASMR, ASDR, and ASIR per 100,000 population attributable to gynecological cancers in 1990 and 2021 by SDI level and country; Table S6: The all-age deaths, DALYs, incident cases, ASMR, ASDR, and ASIR per 100,000 population attributable to gynecological cancers in 1980/1990 and 2021 and the EAPC of ASR from 1980/1990 to 2021 by country in Asia; Table S7: Effects of the aging, population, and epidemiological change on the ASDR by GBD region from 1990 to 2021 attributable to gynecological cancer in Asia; Table S8: Annual percent change in the deaths attributable to gynecological cancers by age groups and GBD region in Asia; Table S9: The predicted deaths and ASMR per 100,000 population attributable to gynecological cancers from 1990 to 2050 in Asia; Table S10: The predicted deaths and age-specific mortality rates per 100,000 population attributable to gynecological cancers from 2022 to 2050 by age group in Asia.

## Abbreviations

The following abbreviations are used in this manuscript:

DALY	Disability-adjusted life years
YLL	Years of life lost
YLD	Years lived with disability
ASMR	Age-standardized mortality rate
ASDR	Age-standardized DALY rate
ASIR	Age-standardized incidence rate
ASR	Age-standardized rate
EAPC	Estimated annual percent change
AAPC	Average annual percent change
SDI	Sociodemographic index
BAPC	Bayesian age-period-cohort model
GBD	Global Burden of Disease
APC-Web	Age-period-cohort web tool
CI	Confidence interval
UI	Uncertainty interval
