# Supplementary material for: The Burden and Trends of Gynecological Cancers in Asia from 1980 to 2021, with Projections to 2050: A Systematic Analysis for the Global Burden of Disease Study 2021
Source: Curr Oncol. 2025 May 23;32(6):298. doi: 10.3390/curroncol32060298 (PMC12191526; doi:10.3390/curroncol32060298)
Supplement: Supplementary file 1 [file curroncol-32-00298-s001.zip › curroncol-3607361-supplementary.pdf]

The burden and trends of gynecological cancers in Asia from 1980 to 2021, with projections to 2050: A systematic analysis for the Global Burden of Disease Study 2021

Supplementary Appendix

Content

Statement of GATHER compliance 4

Supplementary Methods 6

The studied countries contained in Asia 6

The selected GBD regions and countries across Asia 7

Sociodemographic index 8

APC-Web tool 8

Supplementary Results 10

Fig S1. Age-specific mortality rates per 100,000 population for cervical cancer by GBD region and age group in 2021. 10

Fig S2. Age-specific DALY rates per 100,000 population for cervical cancer by GBD region and age group in 2021. 11

Fig S3. Age-specific incidence rates per 100,000 population for cervical cancer by GBD region and age group in 2021. 12

Fig S4. Age-specific mortality rates per 100,000 population for ovarian cancer by GBD region and age group in 2021. 13

Fig S5. Age-specific DALY rates per 100,000 population for ovarian cancer by GBD region and age group in 2021 14

Fig S6. Age-specific incidence rates per 100,000 population for ovarian cancer by GBD region and age group in 2021. 15

Fig S7. Age-specific mortality rates per 100,000 population for uterine cancer by GBD region and age group in 2021. 16

Fig S8. Age-specific DALY rates per 100,000 population for uterine cancer by GBD region and age group in 2021 17

Fig S9. Age-specific incidence rates per 100,000 population for uterine cancer by GBD region and age group in 2021. 18

Fig S10. Age-standardized mortality rates in 2021 attributable to cervical cancer (A); to ovarian cancer (B); to uterine cancer (C). 19

Fig S11. Effects of the aging, population, and epidemiological change on the DALYs by GBD region from 1990 to 2021 in Asia attributable to cervical cancer (A); to ovarian cancer (B); to uterine cancer

(C). The black dots represent the overall difference in disease burden from 1990 to 2021. 20

Fig S12. Age-period-cohort related trends in DALYs from 1992 to 2021 by GBD region attributable to cervical cancer of age effect (A), period effect (D), and cohort effect (G); attributable to ovarian cancer of age effect (B), period effect (E), and cohort effect (H); attributable to uterine cancer of age effect (C), period effect (F), and cohort effect (I). Each vertical row represents one gynecological cancer. DALY=disability adjusted life year. GBD = Global Burden of Disease Study. 21

Fig S13. Annual change per year of age-specific DALY rate of gynecological cancers for different age groups from 1992 to 2021 in Asia. 22

Table S1. Age-specific deaths and age-specific mortality rate per 100,000 population in Asia attributable to gynecological cancers in 1980 and 2021 and the EAPCs of mortality rate from 1980 to 2021. 23

Table S2. Age-specific DALYs and age-specific DALY rate per 100,000 population in Asia attributable to gynecological cancers in 1990 and 2021 and the EAPC of DALY rate from 1990 to 2021. 30

Table S3. Age-specific incident cases and age-specific incidence rate per 100,000 population in Asia attributable to gynecological cancers in 1990 and 2021 and the EAPC of incidence rate from 1990 to 2021. 37

Table S4. The global all-age deaths, DALYs, and incident cases attributable to gynecological cancers in 2021 by four world regions. 44

Table S5. The global ASMR, ASDR, and ASIR per 100,000 population attributable to gynecological cancers in 1990 and 2021 by SDI level and country. 45

Table S6. The all-age deaths, DALYs, incident cases, ASMR, ASDR, and ASIR per 100,000 population attributable to gynecological cancers in 1980/1990 and 2021 and the EAPC of ASR from 1980/1990 to 2021 by country in Asia. 71

Table S7. Effects of the ageing, population, and epidemiological change on the ASDR by GBD region from 1990 to 2021 attributable to gynecological cancer in Asia. 131

Table S8. Annual percent change of the deaths attributable to gynecological cancers by age groups and GBD region in Asia. 134

Table S9. The predicted deaths and ASMR per 100,000 population attributable to gynecological cancers from 1990 to 2050 in Asia. 146

Table S10. The predicted deaths and age-specific mortality rates per 100,000 population attributable to gynecological cancers from 2022 to 2050 by age group in Asia. 153

Statement of GATHER compliance

This study complies with the Guidelines for Accurate and Transparent Health Estimates Reporting (GATHER) recommendations. Below is the GATHER checklist.

Item #	Checklist item	Reference
	Objectives and funding	
1	Define the indicator(s), populations (including age, sex, and geographic entities), and time period(s) for which estimates were made.	Main text
2	List the funding sources for the work.	Abstract
	Data Inputs	
	For all data inputs from multiple sources that are synthesized as part of the study:	
3	Describe how the data were identified and how the data were accessed.	Main text
4	Specify the inclusion and exclusion criteria. Identify all ad-hoc exclusions.	Main text
5	Provide information on all included data sources and their main characteristics. For each data source used, report reference information or contact name/institution, population represented, data collection method, year(s) of data collection, sex and age range, diagnostic criteria or measurement method, and sample size, as relevant.	Main text
6	Identify and describe any categories of input data that have potentially important biases (e.g., based on characteristics listed in item 5).	Main text
	For data inputs that contribute to the analysis but were not synthesized as part of the study:	
7	Describe and give sources for any other data inputs.	Global Health Data Exchange (https://ghdx.healthdata.org/)
	For all data inputs:	
8	Provide all data inputs in a file format from which data can be efficiently extracted (e.g., a spreadsheet rather than a PDF), including all relevant meta-data listed in item 5. For any data inputs that cannot be shared because of ethical or legal reasons, such as third-party ownership, provide a contact name or the name of the institution that retains the right to	Global Health Data Exchange (https://ghdx.healthdata.org/)

	the data.	
Data analysis		
9	Provide a conceptual overview of the data analysis method. A diagram may be helpful.	Main text
10	Provide a detailed description of all steps of the analysis, including mathematical formulae. This description should cover, as relevant, data cleaning, data pre-processing, data adjustments and weighting of data sources, and mathematical or statistical model(s).	Main text
11	Describe how candidate models were evaluated and how the final model(s) were selected.	Main text
12	Provide the results of an evaluation of model performance, if done, as well as the results of any relevant sensitivity analysis.	Main text
13	Describe methods for calculating uncertainty of the estimates. State which sources of uncertainty were, and were not, accounted for in the uncertainty analysis.	Main text
14	State how analytic or statistical source code used to generate estimates can be accessed.	Main text
Results and Discussion		
15	Provide published estimates in a file format from which data can be efficiently extracted.	Main text
16	Report a quantitative measure of the uncertainty of the estimates (e.g. uncertainty intervals).	Main text
17	Interpret results in light of existing evidence. If updating a previous set of estimates, describe the reasons for changes in estimates.	Main text
18	Discuss limitations of the estimates. Include a discussion of any modelling assumptions or data limitations that affect interpretation of the estimates.	Main text

Supplementary Methods

The studied countries contained in Asia

The overall Asia region is consisted of 61 countries and territories according to GBD 2021.

Countries and territories (belong to Asia)	
Afghanistan	Nepal
Australia	New Zealand
Bahrain	Niue
Bangladesh	Oman
Bhutan	Pakistan
Brunei Darussalam	Palau
Cambodia	Palestine
China	Papua New Guinea
Cook Islands	Philippines
Democratic People's Republic of Korea	Qatar
Fiji	Republic of Korea
India	Samoa
Indonesia	Saudi Arabia
Iran (Islamic Republic of)	Singapore
Iraq	Solomon Islands
Israel	Sri Lanka
Japan	Syrian Arab Republic
Jordan	Taiwan (Province of China)
Kazakhstan	Tajikistan
Kiribati	Thailand
Kuwait	Timor-Leste
Kyrgyzstan	Tokelau
Lao People's Democratic Republic	Tonga
Lebanon	Turkmenistan
Malaysia	Tuvalu
Maldives	United Arab Emirates
Marshall Islands	Uzbekistan
Micronesia (Federated States of)	Vanuatu
Mongolia	Viet Nam
Myanmar	Yemen
Nauru	

The selected GBD regions and countries across Asia

We specifically selected 5 GBD regions and 49 countries (including the countries in North Africa and Middle East) across Asia to assess the geographical patterns of disease burden of gynecological cancers.

GBD region	Countries	Counts
Central Asia	Armenia, Azerbaijan, Georgia, Kazakhstan, Kyrgyzstan, Mongolia, Tajikistan, Turkmenistan, Uzbekistan	9
High-income Asia Pacific	Brunei Darussalam, Japan, Singapore, Republic of Korea	4
South Asia	Bangladesh, Bhutan, India, Nepal, Pakistan	5
East Asia	China, Democratic People's Republic of Korea, Taiwan (Province of China)	3
Southeast Asia	Cambodia, Indonesia, Lao People's Democratic Republic, Malaysia, Maldives, Mauritius, Myanmar, Philippines, Seychelles, Sri Lanka, Thailand, Timor-Leste, Vietnam	13
North Africa and Middle East **(part)**	Afghanistan, Bahrain, Iran, Iraq, Jordan, Kuwait, Lebanon, Oman, Palestine, Quatar, Saudi Arabia, Syrian Arab Republic, Turkey, United Arab Emirates, Yemen	15

Sociodemographic index

Regions were classified into five tiers based on their SDI levels: Low SDI, Low-middle SDI, Middle SDI, High-middle SDI, and High SDI regions [1]. It includes indicators related to education, income, and fertility rates, providing a comprehensive view of developmental status. Income per capita, a key component of the SDI, reflects the economic resources available to individuals. Educational attainment, another crucial SDI element, encompasses literacy rates, enrollment rates at primary, secondary, and tertiary levels, and years of schooling. Moreover, the fertility rate, particularly the total fertility rate, represents the average number of children born to women of reproductive age, offering insights into demographic trends and development [2].

Joinpoint analysis

The Joinpoint regression model is used to identify inflection points, or "joinpoints," in time-series data, enabling the detection of significant changes in trends. It calculates the annual percent change (APC) for each segment between these joinpoints, offering insights into how rates of change fluctuate across different time intervals [3, 4].

First, segmented regression is performed using a log-linear model (ln y = β * x), and the grid search method (GSM) is employed to identify all potential joinpoints. For each scenario, the mean squared error (MSE) is calculated, and the joinpoint corresponding to the smallest MSE is selected. Second, the optimal number of joinpoints in the regression model is determined through a Monte Carlo permutation test, with the maximum number of possible joinpoints set to five and the minimum to zero. Finally, the optimal model is used to compute the APC and average annual percent change (AAPC), which quantify trends from 1990 to 2021. The APC is calculated using the following formula:

$$APC = 100\% \times (e^{\beta} - 1)$$

where β is the regression coefficient from the log-linear model (ln y = β * x). The AAPC is derived by weighing the APC of each segment by the width of its respective interval and calculating the weighted average, which reflects the overall trend from 1990 to 2019.

APC-Web tool

The APC-Web tool, as outlined by Rosenberg et al., is an online platform designed to perform APC analysis on cancer incidence and mortality rates (https://analysistools.cancer.gov/apc/) [5]. It dissects cancer trends into three key components: age effects, period effects, and cohort effects. Age effects refer to the variations in cancer rates that occur as an individual ageing, highlighting how susceptibility to certain cancers may increase with age. Period effects examine the impact of specific time periods on cancer rates, capturing the influence of external factors such as changes in medical technology, screening practices, and public health initiatives that can affect cancer diagnosis and treatment. Lastly, cohort effects analyze the differences in cancer incidence and mortality rates among groups of individuals born during the same time frame, reflecting how shared experiences and exposures can shape cancer risk across generations. The tool uses maximum likelihood estimation to fit APC models and visualizes the relative contributions of these factors[5].

This tool is designed to facilitate the analysis of age-specific rates over time and examine the interactions between age, period, and cohort effects in cancer research. Input data is formatted as an age-period matrix containing event counts and person-years, with the requirement that age groups and time intervals are of equal length. Users can easily upload datasets in Excel or CSV format, ensuring accessibility and convenience. The core of the tool involves fitting an APC model using R on the backend. It calculates various estimable parameters, including net drift, local drift, and age-specific rate trends. These parameters help characterize the relationships between age, period, and cohort, providing a detailed view of temporal patterns. The results are presented in the form of graphical and tabular outputs, which can be downloaded in multiple formats, such as text files, Excel workbooks, or R workspace files, enabling further analysis and integration with other software. To support interpretation, the tool integrates Wald tests for evaluating key hypotheses, such as the presence of significant age, period, or cohort effects, and whether temporal trends remain stable over time. These tests offer insights into the underlying dynamics of disease rates, guiding researchers in identifying significant patterns. For instance, the tool highlights local drift, which measures the age-specific annual percentage change and captures variability in trends across different age groups, a critical feature in APC analysis. For analysis, the data must cover full decades, so we selected data spanning thirty years, from 1992 to 2021.

References

1. Rudd KE, Johnson SC, Agesa KM, Shackelford KA, Tsoi D, Kievlan DR, et al. Global, regional, and national sepsis incidence and mortality, 1990-2017: analysis for the Global Burden of Disease Study. Lancet. 2020;395(10219):200-11.
2. Xie J, Wang M, Long Z, Ning H, Li J, Cao Y, et al. Global burden of type 2 diabetes in adolescents and young adults, 1990-2019: systematic analysis of the Global Burden of Disease Study 2019. Bmj. 2022;379:e072385.
3. Global, regional, and national burden of respiratory tract cancers and associated risk factors from 1990 to 2019: a systematic analysis for the Global Burden of Disease Study 2019. Lancet Respir Med. 2021;9(9):1030-49.
4. Ilic M, Ilic I. Cancer mortality in Serbia, 1991-2015: an age-period-cohort and joinpoint regression analysis. Cancer Commun (Lond). 2018;38(1):10.
5. Rosenberg PS, Check DP, Anderson WF. A web tool for age-period-cohort analysis of cancer incidence and mortality rates. Cancer Epidemiol Biomarkers Prev. 2014;23(11):2296-302.

Supplementary Results

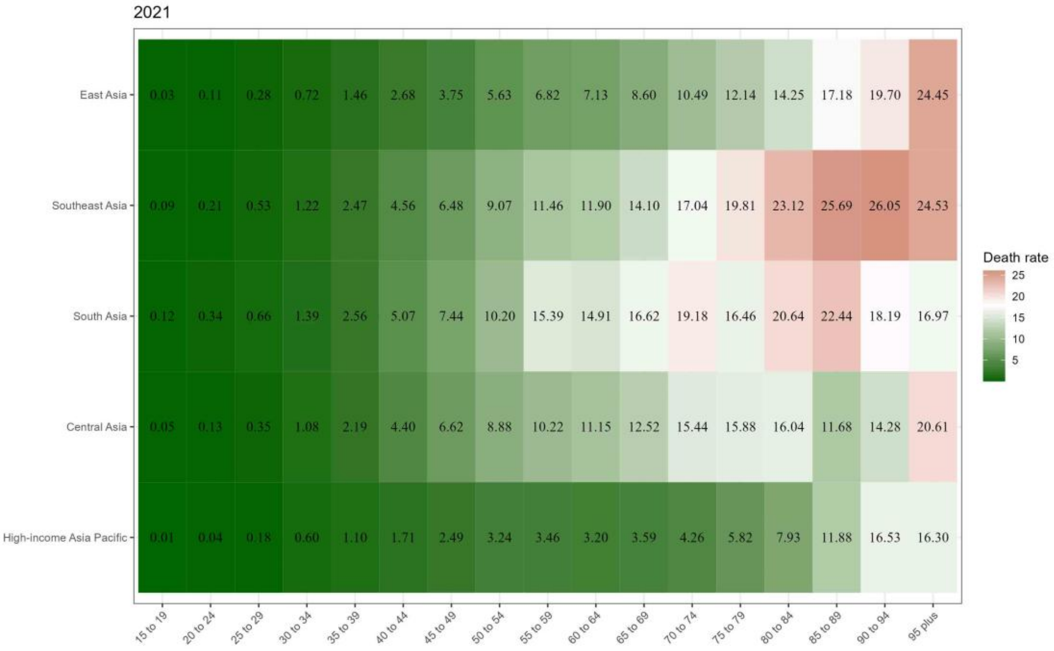

Fig S1. Age-specific mortality rates per 100,000 population for cervical cancer by GBD region and age group in 2021.

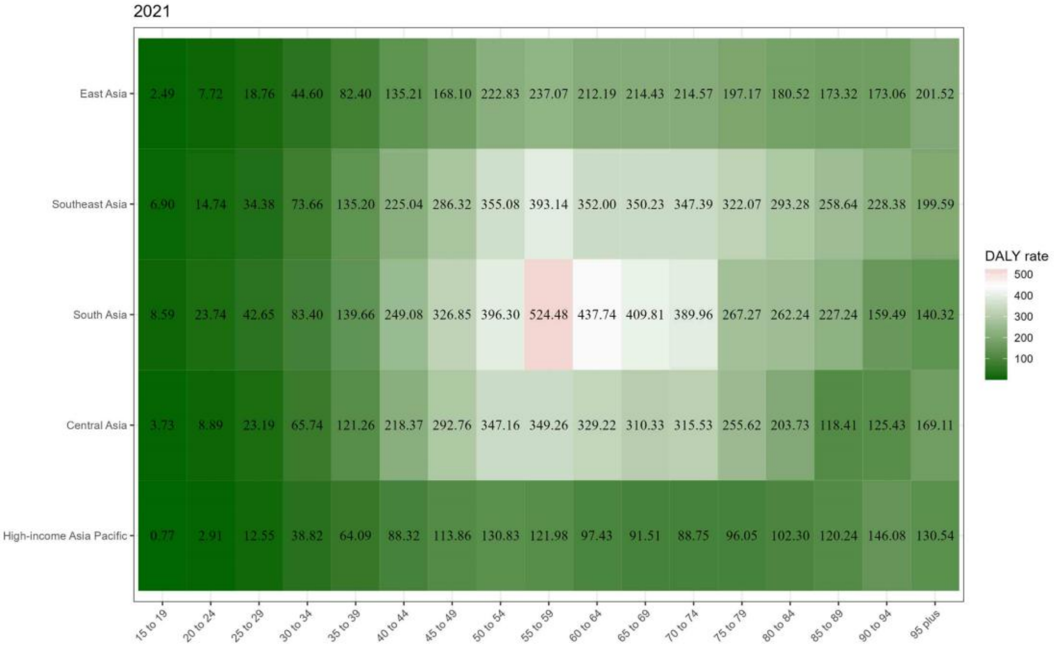

Fig S2. Age-specific DALY rates per 100,000 population for cervical cancer by GBD region and age group in 2021.

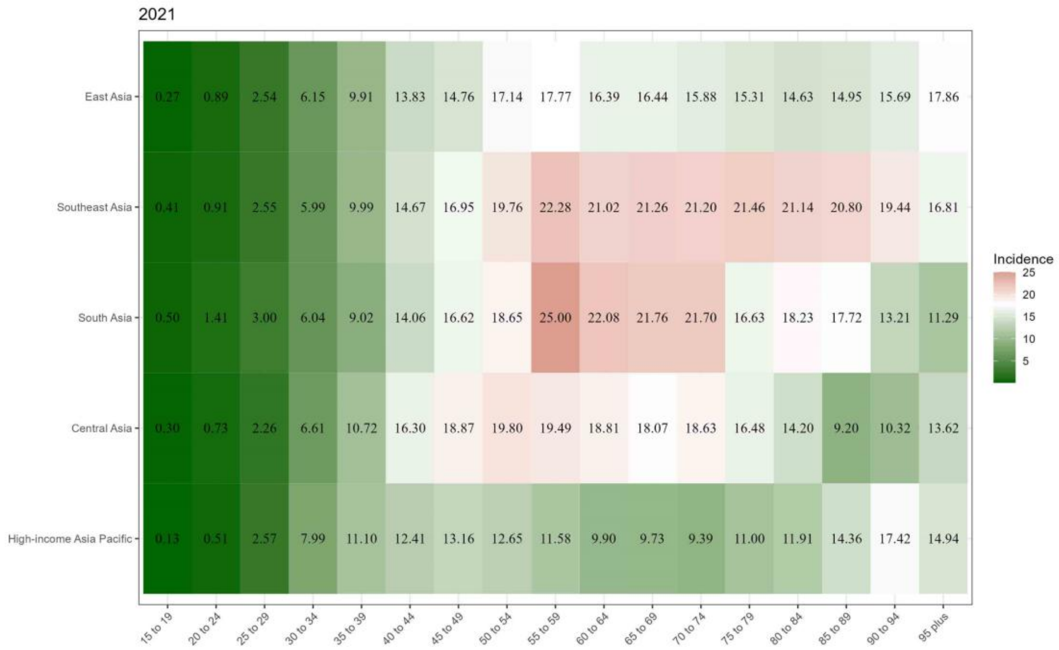

Fig S3. Age-specific incidence rates per 100,000 population for cervical cancer by GBD region and age group in 2021.

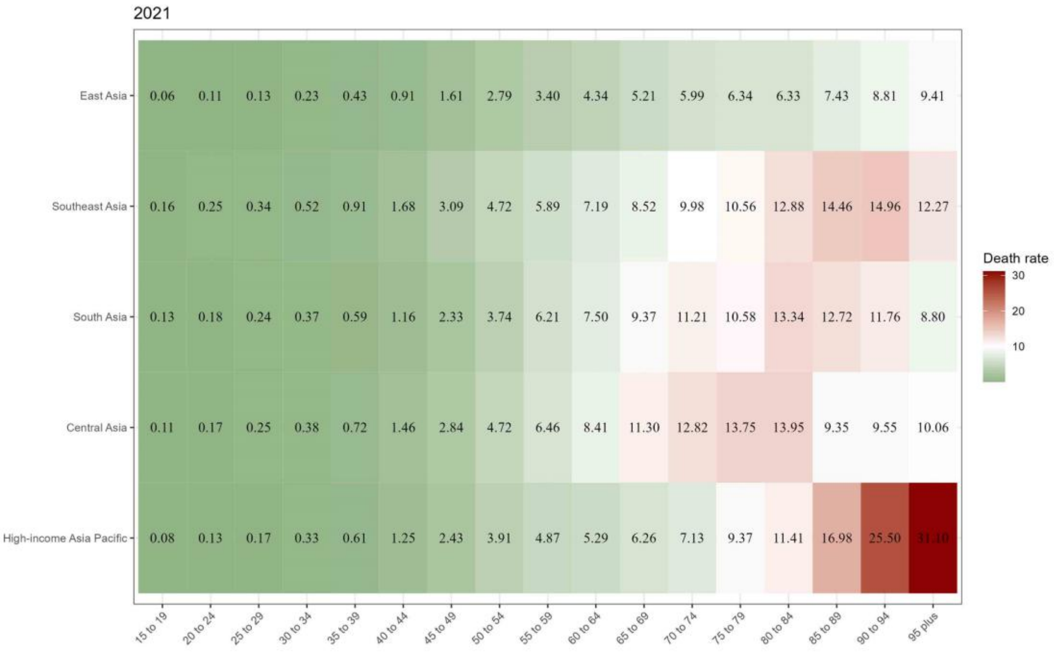

Fig S4. Age-specific mortality rates per 100,000 population for ovarian cancer by GBD region and age group in 2021.

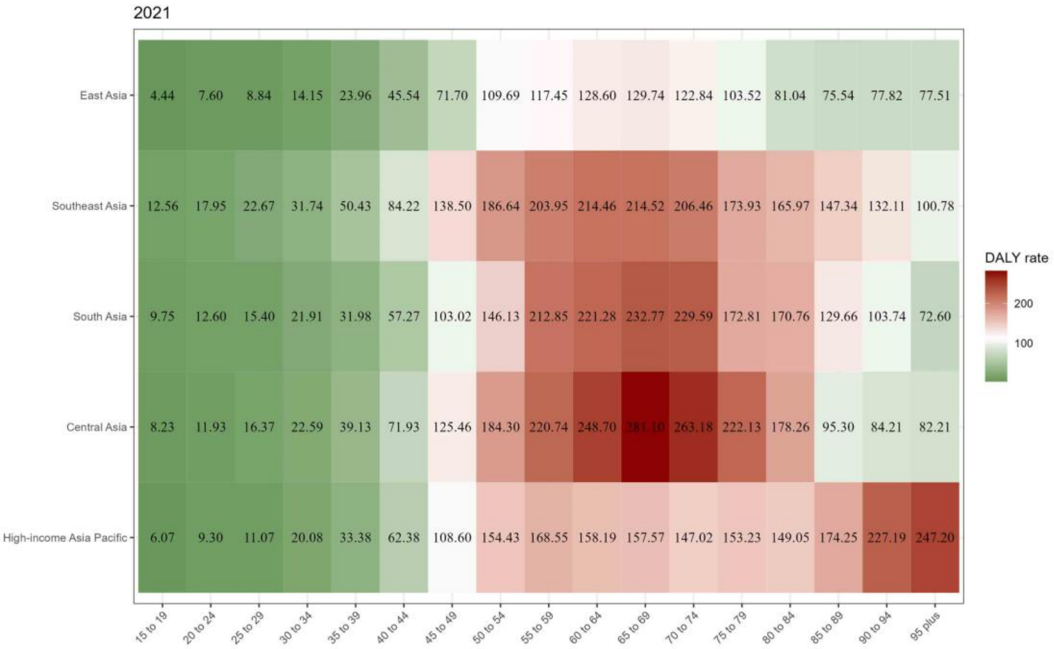

Fig S5. Age-specific DALY rates per 100,000 population for ovarian cancer by GBD region and age group in 2021.

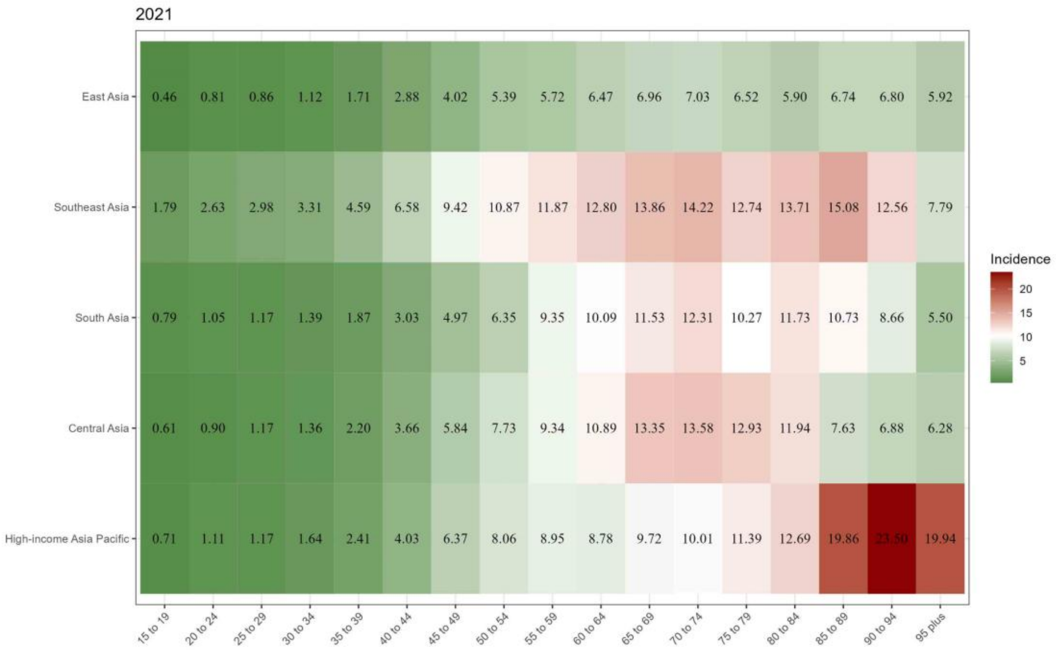

Fig S6. Age-specific incidence rates per 100,000 population for ovarian cancer by GBD region and age group in 2021.

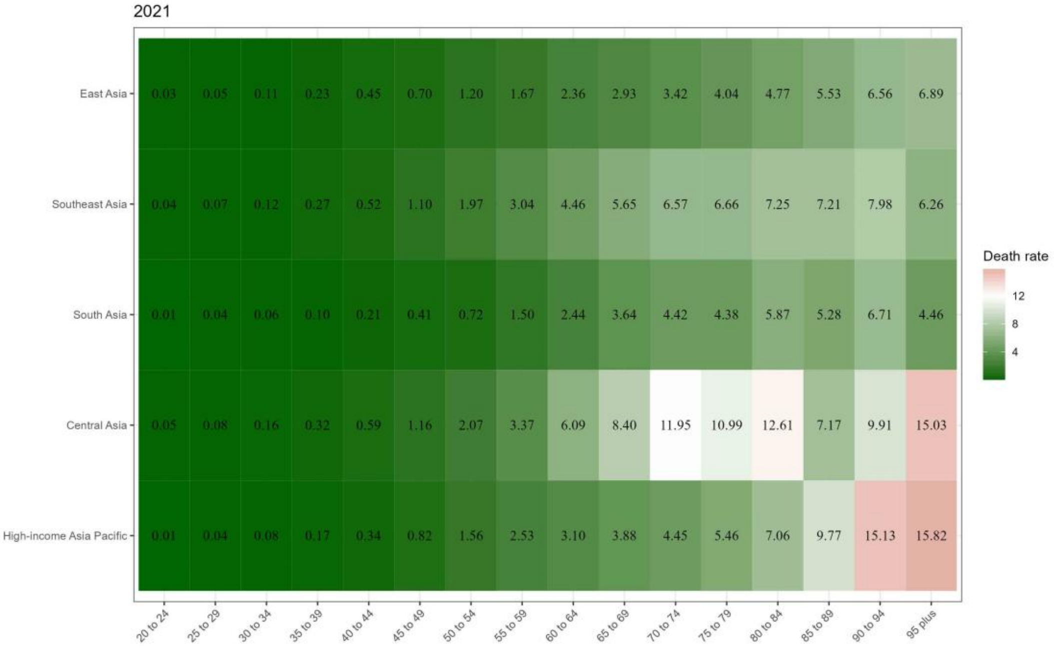

Fig S7. Age-specific mortality rates per 100,000 population for uterine cancer by GBD region and age group in 2021.

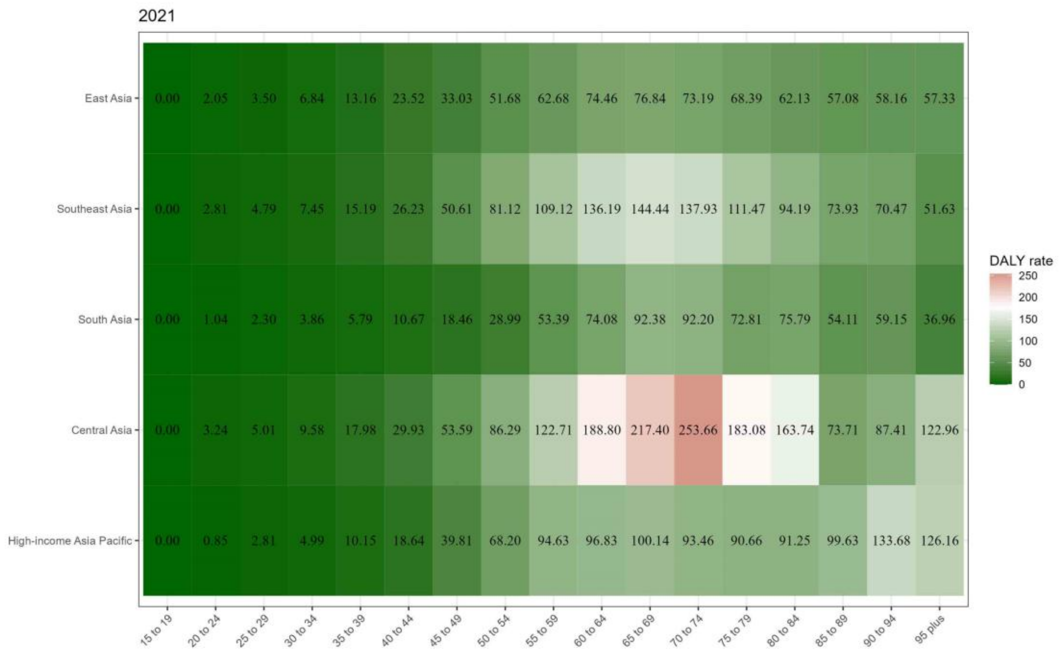

Fig S8. Age-specific DALY rates per 100,000 population for uterine cancer by GBD region and age group in 2021.

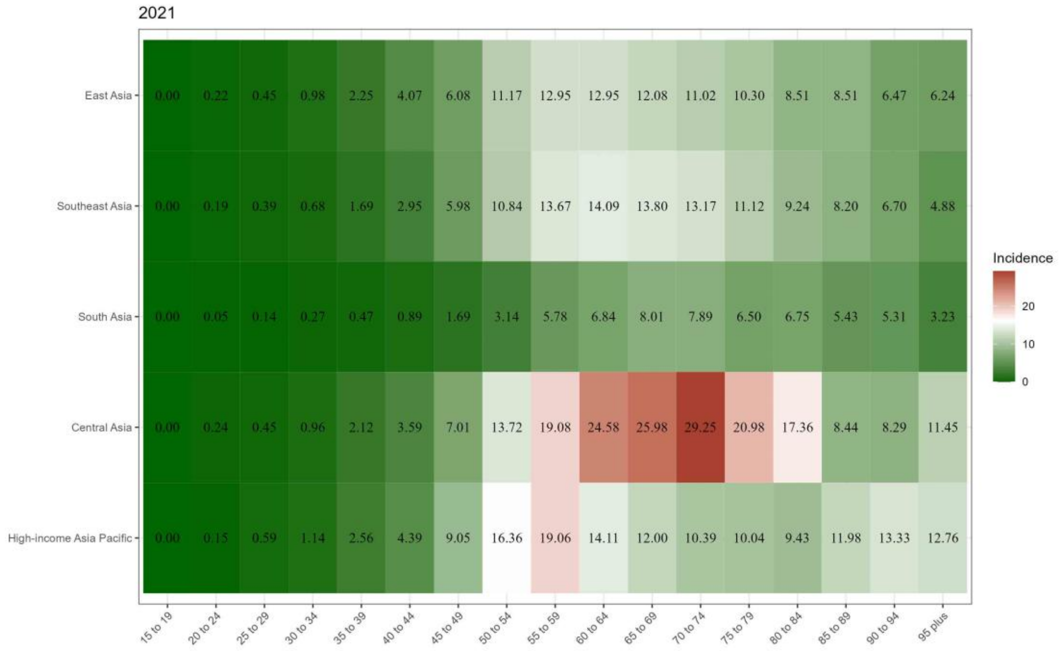

Fig S9. Age-specific incidence rates per 100,000 population for uterine cancer by GBD region and age group in 2021.

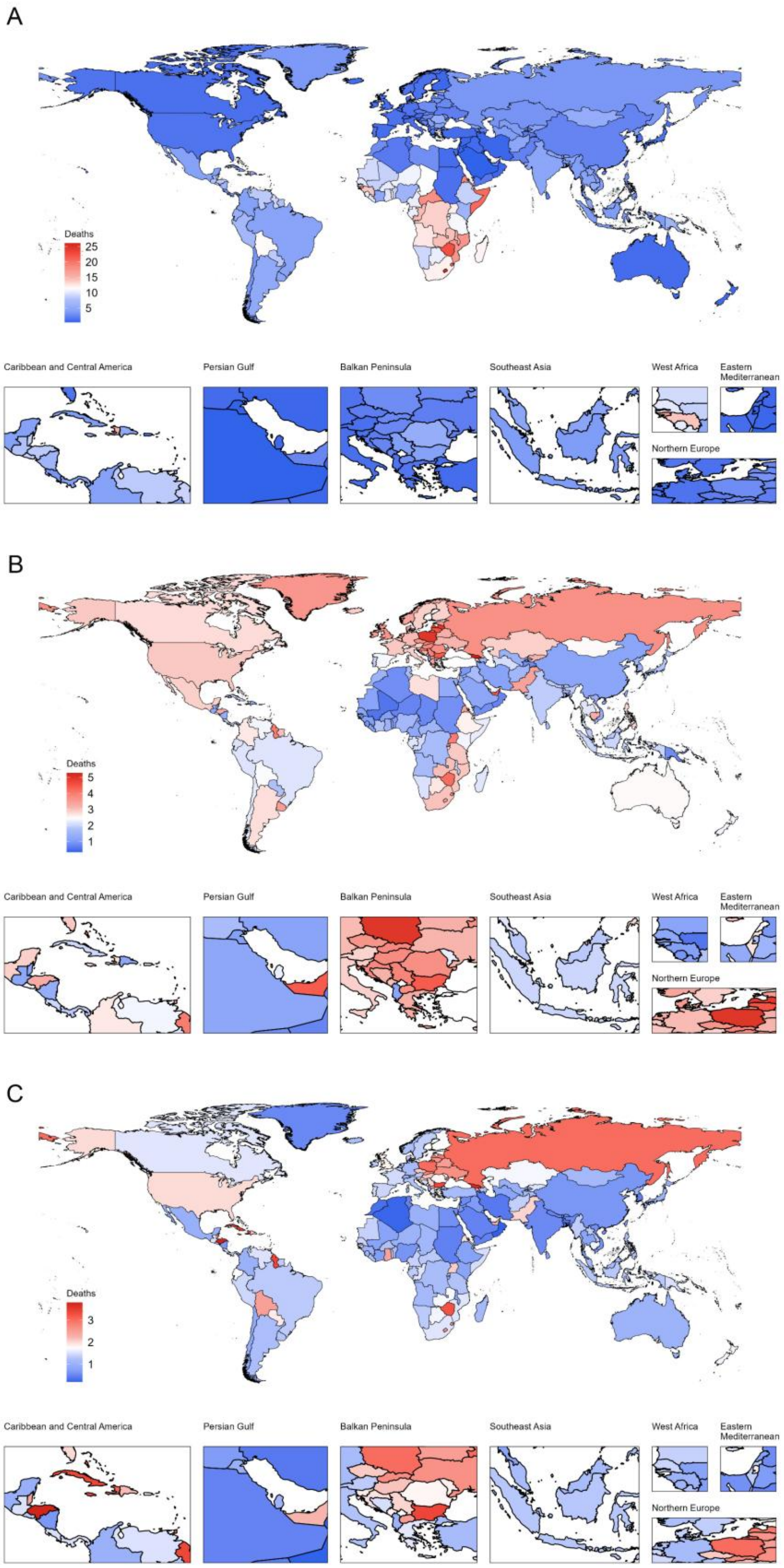

Fig S10. Age-standardized mortality rates in 2021 attributable to cervical cancer (A); to ovarian cancer (B); to uterine cancer (C).

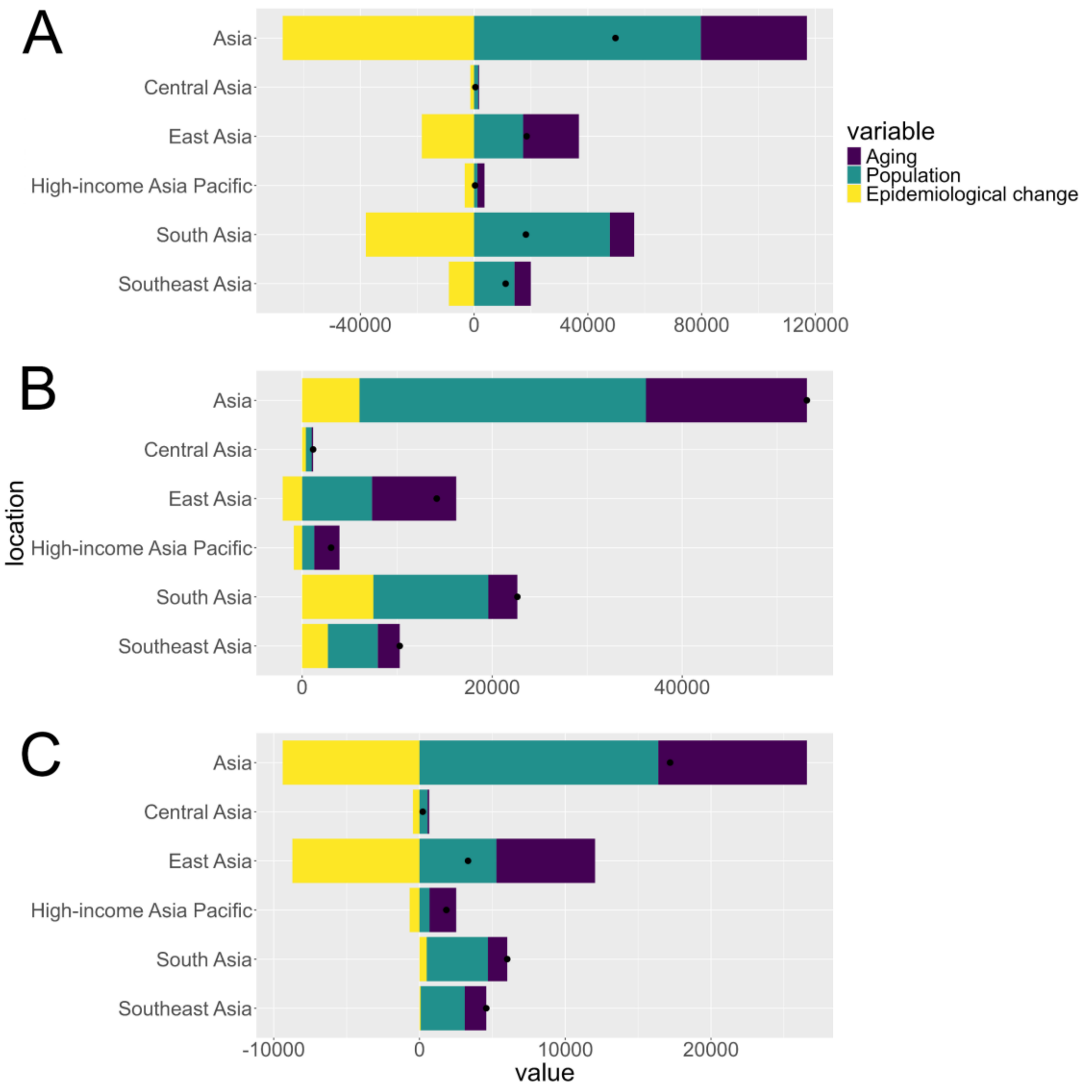

Fig S11. Effects of the aging, population, and epidemiological change on the DALYs by GBD region from 1990 to 2021 in Asia attributable to cervical cancer (A); to ovarian cancer (B); to uterine cancer (C). The black dots represent the overall difference in disease burden from 1990 to 2021.

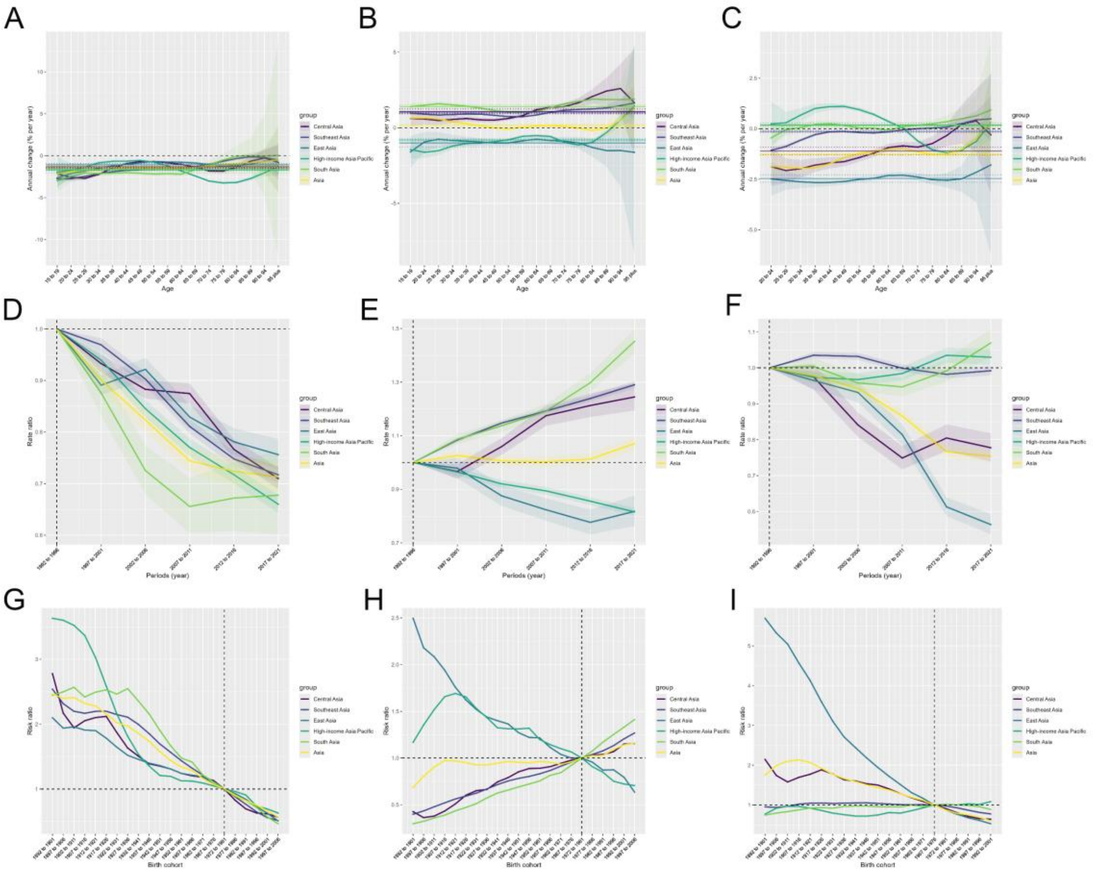

Fig S12. Age-period-cohort related trends in DALYs from 1992 to 2021 by GBD region attributable to cervical cancer of age effect (A), period effect (D), and cohort effect (G); attributable to ovarian cancer of age effect (B), period effect (E), and cohort effect (H); attributable to uterine cancer of age effect (C), period effect (F), and cohort effect (I). Each vertical row represents one gynecological cancer. DALY=disability adjusted life year. GBD = Global Burden of Disease Study.

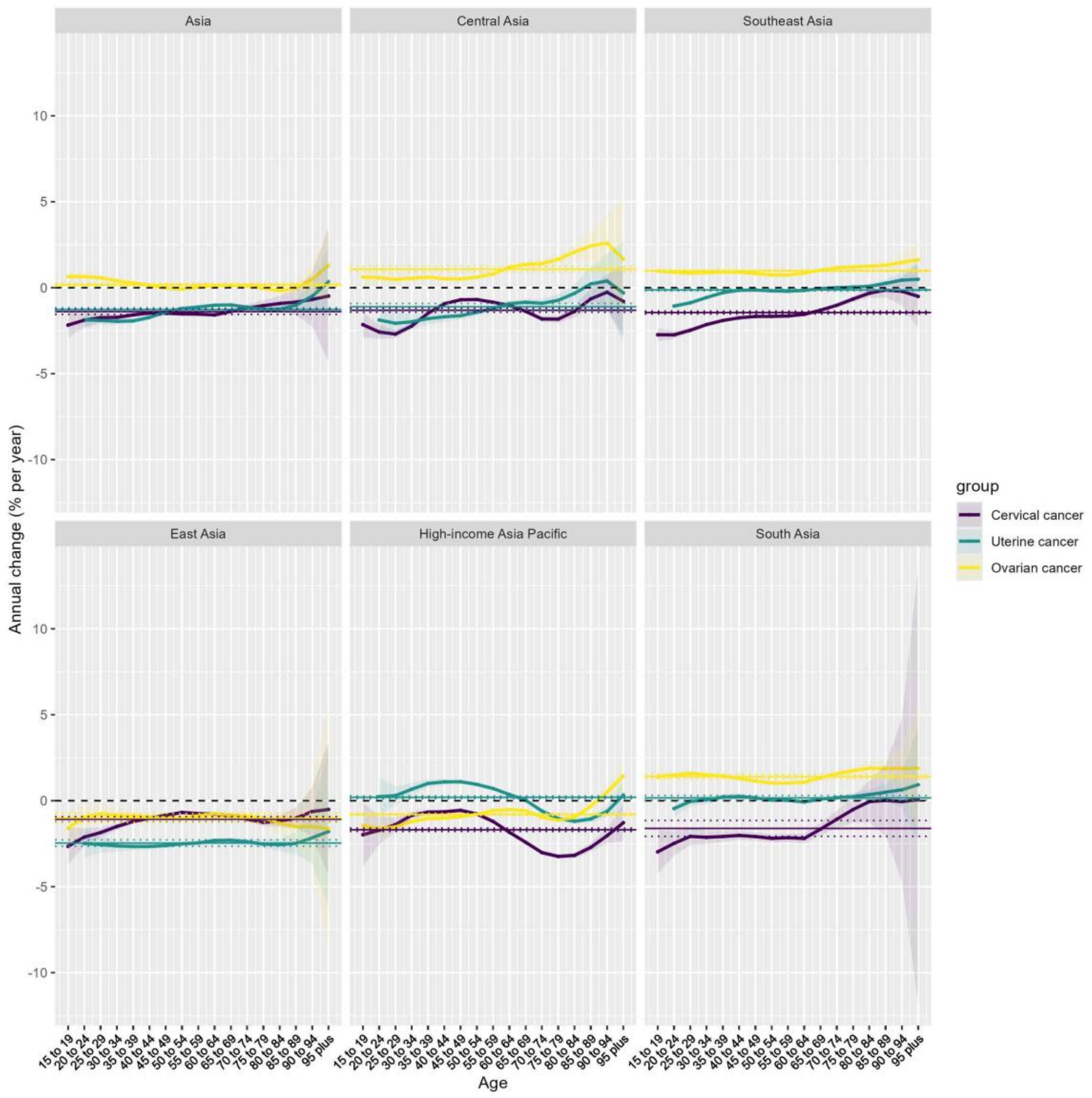

Fig S13. Annual change per year of age-specific DALY rate of gynecological cancers for different age groups from 1992 to 2021 in Asia.

Table S1. Age-specific deaths and age-specific mortality rate per 100,000 population in Asia attributable to gynecological cancers in 1980 and 2021 and the EAPCs of mortality rate from 1980 to 2021.

Age	1980		2021		EAPC for mortality rate from 1980 to 2021[b]
	Counts[a]	Mortality rate[a]	Counts[a]	Mortality rate[a]	
Cervical cancer					
15-19 years	417.79 (343.24-521.43)	0.15 (0.12-0.19)	296.57 (245.92-387.74)	0.08 (0.07-0.11)	-1.38 (-1.52--1.25)
20-24 years	853.38 (692.89-1072.01)	0.39 (0.32-0.49)	823.87 (687.28-992.89)	0.24 (0.2-0.29)	-1.09 (-1.25--0.92)
25-29 years	1971.29 (1645.38-2400.46)	0.92 (0.77-1.12)	1707.19 (1469.99-1947.63)	0.49 (0.42-0.55)	-1.47 (-1.57--1.36)
30-34 years	3608.65 (3031.05-4366.17)	2.15 (1.81-2.6)	3891.05 (3368.37-4415.07)	1.04 (0.9-1.18)	-1.77 (-1.82--1.73)
35-39 years	6277.05 (5273.56-7557.88)	4.46 (3.75-5.37)	6829.26 (5966.09-7842.07)	1.98 (1.73-2.28)	-1.94 (-2--1.87)
40-44 years	10880.99 (9348.8-12999.92)	8.42 (7.23-10.06)	11464.6 (10127.72-12930.67)	3.78 (3.34-4.26)	-2.02 (-2.14--1.9)

45-49 years	13704.18 (11850.28-16268.78)	11.92 (10.31-14.16)	15645.55 (13799.83-17646.44)	5.25 (4.63-5.92)	-2.12 (-2.27--1.98)
50-54 years	15999.24 (13647.57-18921.41)	16.59 (14.15-19.62)	20551.66 (18008.74-23411.63)	7.23 (6.34-8.24)	-2.05 (-2.23--1.87)
55-59 years	15624.28 (13276.84-18834.42)	20.11 (17.09-24.24)	23689.33 (20529.58-27112.14)	9.61 (8.33-11)	-1.89 (-2.06--1.72)
60-64 years	12225.82 (10513.59-14520.47)	19.86 (17.07-23.58)	18603.56 (16483.65-20835.14)	9.91 (8.78-11.1)	-1.74 (-1.84--1.64)
65-69 years	9466.13 (8191.25-11217.29)	20.14 (17.43-23.87)	18436.69 (16067.87-20946.81)	11.03 (9.62-12.54)	-1.5 (-1.58--1.42)
70-74 years	7276.42 (6312.02-8628.64)	22.08 (19.16-26.19)	15403.99 (13490.5-17457.67)	12.82 (11.23-14.53)	-1.34 (-1.41--1.28)
75-79 years	4162.69 (3580.84-4942.55)	21.77 (18.73-25.85)	10013.29 (8508.98-11344.36)	13.2 (11.21-14.95)	-1.24 (-1.3--1.18)
80-84 years	1931.93 (1664.1-2311.89)	22.56 (19.44-27)	7262.42 (6087.21-8285.97)	15.54 (13.02-17.73)	-0.86 (-0.91--0.82)
85-89 years	808.39 (684.14-962.28)	25.48 (21.56-30.33)	4108.2 (3218.98-4778.36)	17.69 (13.86-20.58)	-0.87 (-0.94--0.79)

90-94 years	206.88 (170.22-252.7)	23.75 (19.54-29.01)	1532.17 (1170.2-1784.98)	18.68 (14.26-21.76)	-0.56 (-0.66--0.46)
95+ years	54.16 (41.74-71.12)	24.93 (19.21-32.74)	464.42 (326.38-554.44)	19.67 (13.82-23.48)	-0.54 (-0.63--0.46)
All ages	105469.25 (91934.2-124165.45)	4.04 (3.53-4.76)	160723.84 (143073.14-179396.56)	3.48 (3.09-3.88)	-0.36 (-0.51--0.22)
Ovarian cancer					
15-19 years	226.65 (136.3-339.55)	0.08 (0.05-0.12)	394.16 (288.28-475.87)	0.11 (0.08-0.13)	0.56 (0.45-0.67)
20-24 years	254.03 (155.91-389.99)	0.12 (0.07-0.18)	569.62 (434.63-702.24)	0.16 (0.13-0.2)	0.8 (0.72-0.88)
25-29 years	344.29 (209.66-528.81)	0.16 (0.1-0.25)	762.48 (608.13-900.6)	0.22 (0.17-0.26)	0.7 (0.65-0.75)
30-34 years	444.39 (286.35-670.2)	0.26 (0.17-0.4)	1253.57 (1031.82-1489.13)	0.33 (0.28-0.4)	0.52 (0.49-0.56)
35-39 years	678.48 (471.47-1015.98)	0.48 (0.34-0.72)	1973.89 (1669.29-2301.33)	0.57 (0.48-0.67)	0.26 (0.18-0.35)

40-44 years	1270.15 (907.31-1852.74)	0.98 (0.7-1.43)	3467.43 (2974.4-4079.09)	1.14 (0.98-1.34)	0.18 (0.09-0.27)
45-49 years	2095.11 (1555.58-3023.89)	1.82 (1.35-2.63)	6321.89 (5355.26-7315.01)	2.12 (1.8-2.46)	0.2 (0.13-0.27)
50-54 years	2947.15 (2291.79-4194.53)	3.06 (2.38-4.35)	9691.83 (8168.46-11421.81)	3.41 (2.88-4.02)	0.09 (-0.01-0.2)
55-59 years	3083 (2426.45-4413.44)	3.97 (3.12-5.68)	11524.17 (9952.76-13936.72)	4.67 (4.04-5.65)	0.28 (0.17-0.39)
60-64 years	2864.43 (2290.47-3825.21)	4.65 (3.72-6.21)	11113.35 (9615.15-12934.88)	5.92 (5.12-6.89)	0.45 (0.38-0.53)
65-69 years	2597.13 (2091.72-3323.57)	5.53 (4.45-7.07)	11672.25 (10009.22-13543.59)	6.99 (5.99-8.11)	0.47 (0.38-0.55)
70-74 years	2034.14 (1622.5-2630.55)	6.17 (4.92-7.98)	9881.39 (8423.42-11541.13)	8.22 (7.01-9.6)	0.54 (0.46-0.62)
75-79 years	1278.95 (1028.94-1588.64)	6.69 (5.38-8.31)	6526.21 (5513.91-7773.22)	8.6 (7.27-10.24)	0.46 (0.32-0.6)
80-84 years	655.27 (515.58-807.74)	7.65 (6.02-9.43)	4671.39 (3848.13-5430.62)	9.99 (8.23-11.62)	0.56 (0.35-0.78)

85-89 years	267.93 (213.67-327.46)	8.44 (6.73-10.32)	2730.61 (2096.21-3193.05)	11.76 (9.03-13.75)	0.79 (0.52-1.05)
90-94 years	79.93 (61.88-96.67)	9.18 (7.11-11.1)	1265.25 (918-1499.98)	15.42 (11.19-18.28)	1.37 (1.1-1.65)
95+ years	17.64 (12.28-22.03)	8.12 (5.65-10.14)	459.41 (300.74-554.44)	19.46 (12.74-23.48)	2.35 (2.12-2.58)
All ages	21138.66 (16819.56-28334.13)	0.81 (0.64-1.09)	84278.89 (73874.6-97734.25)	1.82 (1.6-2.11)	1.9 (1.85-1.96)
Uterine cancer					
20-24 years	87.4 (35.3-123.4)	0.04 (0.02-0.06)	78.61 (55.26-97.55)	0.02 (0.02-0.03)	-1.4 (-1.54--1.27)
25-29 years	191.65 (78.78-267.24)	0.09 (0.04-0.12)	161.61 (116.67-200.67)	0.05 (0.03-0.06)	-1.49 (-1.6--1.38)
30-34 years	281.56 (127.87-400.54)	0.17 (0.08-0.24)	335.92 (243.76-423.07)	0.09 (0.07-0.11)	-1.59 (-1.74--1.44)
35-39 years	480.43 (235.64-674.98)	0.34 (0.17-0.48)	603.02 (446.97-742.1)	0.18 (0.13-0.22)	-1.64 (-1.83--1.45)

40-44 years	877.45 (477.07-1191.51)	0.68 (0.37-0.92)	1056.59 (811.82-1274.92)	0.35 (0.27-0.42)	-1.46 (-1.62--1.3)
45-49 years	1376.1 (832.17-1832.33)	1.2 (0.72-1.59)	1963.38 (1511.14-2346.89)	0.66 (0.51-0.79)	-1.32 (-1.44--1.19)
50-54 years	2029.71 (1325.21-2684.01)	2.1 (1.37-2.78)	3344.38 (2562.69-4160.19)	1.18 (0.9-1.46)	-1.4 (-1.52--1.29)
55-59 years	2454.78 (1648.01-3166.75)	3.16 (2.12-4.08)	4605.78 (3753.54-5683.63)	1.87 (1.52-2.31)	-1.26 (-1.34--1.18)
60-64 years	2673.73 (1960.9-3282.02)	4.34 (3.18-5.33)	5267.3 (4346.67-6604.57)	2.81 (2.32-3.52)	-1.03 (-1.09--0.96)
65-69 years	2615.51 (2003.37-3172.77)	5.56 (4.26-6.75)	6043.71 (4983.43-7516.66)	3.62 (2.98-4.5)	-1.06 (-1.14--0.98)
70-74 years	2143.8 (1712.46-2562.09)	6.51 (5.2-7.78)	5149.82 (4342.33-6626.86)	4.29 (3.61-5.51)	-1.09 (-1.14--1.04)
75-79 years	1447.36 (1156.25-1729.31)	7.57 (6.05-9.04)	3563.81 (2960.68-4627.49)	4.7 (3.9-6.1)	-1.22 (-1.29--1.15)
80-84 years	750.66 (610.82-884.16)	8.77 (7.13-10.33)	2735.49 (2175.68-3490.96)	5.85 (4.66-7.47)	-0.98 (-1.1--0.87)

85-89 years	284.69 (230.35-342.04)	8.97 (7.26-10.78)	1557.12 (1152.07-1963.16)	6.71 (4.96-8.45)	-0.63 (-0.76--0.49)
90-94 years	81.48 (65.47-99.49)	9.36 (7.52-11.42)	783.16 (554.87-947.32)	9.55 (6.76-11.55)	0.12 (-0.02-0.27)
95+ years	16.21 (12.11-21.77)	7.46 (5.57-10.02)	259.21 (170.82-311.85)	10.98 (7.23-13.21)	1.1 (1-1.2)
All ages	17792.51 (12812.07-22268.12)	0.68 (0.49-0.85)	37508.92 (31519.05-45758.59)	0.81 (0.68-0.99)	0.46 (0.38-0.54)

[a] represents data in parentheses is the 95% uncertainty interval; [b] represents data in parentheses is the 95% confidence interval; EAPC= estimated annual percent change.

Table S2. Age-specific DALYs and age-specific DALY rate per 100,000 population in Asia attributable to gynecological cancers in 1990 and 2021 and the EAPC of DALY rate from 1990 to 2021.

Age	1990		2021		EAPC for DALY rate from 1990 to 2021[b]
	Counts[a]	DALY rate[a]	Counts[a]	DALY rate[a]	
Cervical cancer					
15-19 years	32275.73 (27335.19-37653.63)	9.88 (8.37-11.52)	22209.03 (18465.45-28991.11)	6.29 (5.23-8.21)	-1.69 (-1.86--1.53)
20-24 years	71288.55 (61214.25-84651.74)	22.81 (19.59-27.09)	57557.75 (47865.12-69816.17)	16.63 (13.83-20.17)	-1.27 (-1.52--1.01)
25-29 years	132466.64 (114781.25-150806.54)	48.94 (42.41-55.72)	111677.42 (96467.96-127471.35)	31.82 (27.48-36.32)	-1.38 (-1.55--1.2)
30-34 years	237983.14 (205875.87-274934.68)	104.62 (90.51-120.87)	235720.7 (203945.26-268836.01)	62.9 (54.42-71.74)	-1.69 (-1.76--1.63)
35-39 years	403062.47 (347708.81-457078.07)	189.73 (163.68-215.16)	376313.57 (328296.8-430497.65)	109.32 (95.38-125.07)	-1.75 (-1.84--1.66)
40-44 years	536989.24 (467439.04-615712.57)	320.38 (278.88-367.35)	567832.7 (504237.11-638044.82)	187.08 (166.13-210.21)	-1.68 (-1.83--1.53)

45-49 years	551257.11 (485047.41-620275.14)	407.73 (358.76-458.78)	692629.55 (611793.86-784370.43)	232.55 (205.41-263.35)	-1.73 (-1.91--1.55)
50-54 years	599016.51 (513957.78-672463.53)	502.05 (430.76-563.6)	805621.48 (705010.18-918846.1)	283.58 (248.16-323.43)	-1.7 (-1.97--1.43)
55-59 years	542567.39 (472260.87-610543.39)	520.85 (453.36-586.11)	814057.36 (705940.7-928315.83)	330.2 (286.35-376.55)	-1.57 (-1.82--1.31)
60-64 years	389596.88 (337917.3-438659.52)	458.72 (397.87-516.49)	549643.29 (488273.74-615883.16)	292.8 (260.11-328.09)	-1.54 (-1.69--1.39)
65-69 years	255820.42 (224261.94-289833.37)	407.63 (357.35-461.83)	457420.33 (398732.05-519235.89)	273.78 (238.65-310.78)	-1.31 (-1.42--1.19)
70-74 years	162337.58 (141444.05-186222.67)	379.56 (330.71-435.41)	314339.15 (275576.79-357486.39)	261.57 (229.31-297.47)	-1.19 (-1.28--1.09)
75-79 years	82404.49 (72035.71-92547.83)	307.55 (268.85-345.4)	162793.3 (138588.97-184606.44)	214.52 (182.63-243.27)	-1.13 (-1.23--1.04)
80-84 years	36011.2 (30797.97-40811.93)	256.74 (219.58-290.97)	92265.59 (77370.72-105005.09)	197.41 (165.54-224.67)	-0.84 (-0.91--0.76)
85-89 years	12735.9 (10748.55-14677.14)	237.76 (200.66-274)	41495.4 (32420.95-48252.02)	178.7 (139.62-207.8)	-0.98 (-1.1--0.86)

90-94 years	2718.08 (2252.82-3145.61)	203.32 (168.51-235.3)	13473.09 (10277.76-15719.86)	164.23 (125.28-191.62)	-0.76 (-0.89--0.63)
95+ years	529.31 (409.75-640.35)	192.49 (149.01-232.87)	3787.95 (2673.77-4521.24)	160.43 (113.24-191.49)	-0.47 (-0.59--0.35)
All ages	4049060.64 (3572517.1-4543446.09)	127.53 (112.52-143.1)	5318837.66 (4733229.92-5916376.88)	115.06 (102.39-127.98)	-0.28 (-0.41--0.15)
Ovarian cancer					
15-19 years	22412.05 (14356.49-29548.72)	6.86 (4.39-9.04)	30001.2 (21892.93-36271.11)	8.49 (6.2-10.27)	0.45 (0.28-0.62)
20-24 years	28125.02 (17608.64-37877.63)	9 (5.63-12.12)	40655.22 (31025.29-49884.49)	11.75 (8.96-14.41)	0.81 (0.67-0.94)
25-29 years	30337.77 (19428.61-42202.91)	11.21 (7.18-15.59)	50225.04 (40043.24-59057.9)	14.31 (11.41-16.83)	0.79 (0.71-0.87)
30-34 years	38734.21 (25585.58-51516.02)	17.03 (11.25-22.65)	75536.18 (61730.37-89474.97)	20.16 (16.47-23.88)	0.53 (0.48-0.59)
35-39 years	62185.85 (44856.58-81238.8)	29.27 (21.12-38.24)	108622.26 (91609.95-126650.7)	31.56 (26.61-36.79)	0.15 (0.03-0.28)

40-44 years	88701.86 (67618.3-112400.1)	52.92 (40.34-67.06)	172538.5 (148156.63-202783.84)	56.84 (48.81-66.81)	0.03 (-0.1-0.16)
45-49 years	116911.76 (93025.48-145196.12)	86.47 (68.81-107.39)	280978.94 (237156.79-325783.04)	94.34 (79.63-109.38)	0.06 (-0.03-0.15)
50-54 years	154452.09 (127267.05-192520.98)	129.45 (106.66-161.35)	380527.56 (320005.66-448244.62)	133.95 (112.64-157.78)	-0.18 (-0.29--0.07)
55-59 years	153258.11 (127505.89-188861.47)	147.12 (122.4-181.3)	396839.17 (342154.3-479515.92)	160.97 (138.79-194.5)	0.04 (-0.11-0.18)
60-64 years	126840.51 (107889.55-153740.15)	149.34 (127.03-181.02)	329313.42 (285191.88-382992.1)	175.43 (151.92-204.02)	0.28 (0.19-0.37)
65-69 years	94256.72 (81178.94-114682.59)	150.19 (129.35-182.74)	291163.6 (249921.97-336605.77)	174.27 (149.59-201.47)	0.26 (0.16-0.35)
70-74 years	61719.21 (52387.41-73312.33)	144.31 (122.49-171.41)	202886.63 (173305.78-236210.9)	168.83 (144.21-196.56)	0.3 (0.23-0.38)
75-79 years	34752.77 (29447.21-40700.12)	129.7 (109.9-151.9)	106727.81 (90205.11-127085.83)	140.64 (118.87-167.47)	0 (-0.12-0.11)
80-84 years	16221.91 (13730.42-18959)	115.66 (97.89-135.17)	60070.39 (49482.66-69971.01)	128.53 (105.87-149.71)	-0.09 (-0.3-0.11)

85-89 years	5854.86 (4835.66-6773.68)	109.3 (90.27-126.45)	27855.76 (21398.4-32511.79)	119.96 (92.15-140.01)	0.02 (-0.25-0.3)
90-94 years	1418.12 (1148.59-1633.09)	106.08 (85.92-122.16)	11214.02 (8148.4-13315.56)	136.69 (99.32-162.31)	0.61 (0.32-0.9)
95+ years	252.64 (190.92-295.39)	91.87 (69.43-107.42)	3698.68 (2434.86-4457.74)	156.65 (103.12-188.8)	1.68 (1.44-1.92)
All ages	1036435.48 (844239.2-1272438.2)	32.64 (26.59-40.08)	2568854.39 (2233137.32-2992340.5)	55.57 (48.31-64.73)	1.57 (1.49-1.65)
Uterine cancer					
20-24 years	8224.73 (3831.06-11020.28)	2.63 (1.23-3.53)	5554.23 (3894.2-6880.74)	1.6 (1.13-1.99)	-1.69 (-1.82--1.56)
25-29 years	12785 (6519.02-16644.18)	4.72 (2.41-6.15)	10676.36 (7690.07-13253.15)	3.04 (2.19-3.78)	-1.57 (-1.71--1.42)
30-34 years	19263.75 (10324.3-25230.65)	8.47 (4.54-11.09)	20604.06 (14859.02-25891.14)	5.5 (3.97-6.91)	-1.67 (-1.9--1.43)
35-39 years	36529.17 (21764.64-47381.08)	17.2 (10.25-22.3)	34373.51 (25316.78-42050.56)	9.99 (7.35-12.22)	-1.94 (-2.19--1.68)

40-44 years	47613.69 (30152.49-59486.48)	28.41 (17.99-35.49)	54563.28 (41583.98-65318.49)	17.98 (13.7-21.52)	-1.64 (-1.87--1.42)
45-49 years	57469.18 (37851.84-70364.17)	42.51 (28-52.04)	91503.54 (70153-109283)	30.72 (23.55-36.69)	-1.21 (-1.4--1.01)
50-54 years	83225.88 (58938.43-102291.91)	69.75 (49.4-85.73)	140839.96 (107750.8-174818.83)	49.58 (37.93-61.54)	-1.33 (-1.52--1.15)
55-59 years	94857.34 (69748.9-114806.46)	91.06 (66.96-110.21)	169033.61 (136947.25-208537.69)	68.56 (55.55-84.59)	-1.15 (-1.28--1.02)
60-64 years	91901.04 (70102.27-108415.75)	108.21 (82.54-127.65)	163101.73 (133638.33-205606.22)	86.89 (71.19-109.53)	-0.85 (-0.94--0.76)
65-69 years	74798.64 (59093.39-88332.27)	119.19 (94.16-140.75)	156247.21 (128563.48-193882.99)	93.52 (76.95-116.04)	-0.96 (-1.08--0.84)
70-74 years	50558.97 (40676.88-59683.14)	118.21 (95.11-139.55)	109076.16 (93420.39-141202.72)	90.76 (77.74-117.5)	-1.04 (-1.13--0.96)
75-79 years	29569.02 (24083.29-34718.36)	110.36 (89.88-129.57)	59948.73 (49674.56-78159.5)	79 (65.46-103)	-1.31 (-1.4--1.21)
80-84 years	14064.53 (11335.87-16795.5)	100.27 (80.82-119.74)	35565.9 (28276.23-45323.18)	76.1 (60.5-96.97)	-1.18 (-1.33--1.03)

85-89 years	4610.55 (3731.73-5567.46)	86.07 (69.67-103.94)	16009.45 (11953.12-20242.15)	68.94 (51.48-87.17)	-0.89 (-1.07--0.7)
90-94 years	1159.47 (920.56-1343.52)	86.73 (68.86-100.5)	6926.22 (4932.28-8387.03)	84.43 (60.12-102.23)	-0.23 (-0.4--0.05)
95+ years	182.22 (132.76-228.04)	66.26 (48.28-82.93)	2103.5 (1387.46-2537.55)	89.09 (58.76-107.47)	0.91 (0.77-1.04)
All ages	626813.16 (462084.19-746542.48)	19.74 (14.55-23.51)	1076127.45 (889738.88-1303328.41)	23.28 (19.25-28.19)	0.37 (0.27-0.47)

[a] represents data in parentheses is the 95% uncertainty interval; [b] represents data in parentheses is the 95% confidence interval; DALY= disability-adjusted life year; EAPC= estimated annual percent change.

Table S3. Age-specific incident cases and age-specific incidence rate per 100,000 population in Asia attributable to gynecological cancers in 1990 and 2021 and the EAPC of incidence rate from 1990 to 2021.

Age	1990		2021		EAPC for incidence rate from 1990 to 2021[b]
	Counts[a]	Incidence rate[a]	Counts[a]	Incidence rate[a]	
Cervical cancer					
15-19 years	1401.09 (1195.94-1619.25)	0.43 (0.37-0.5)	1407.31 (1187.69-1831.22)	0.4 (0.34-0.52)	-0.43 (-0.56--0.3)
20-24 years	3257.55 (2805.15-3860.45)	1.04 (0.9-1.24)	3815.75 (3203.09-4563.43)	1.1 (0.93-1.32)	0.04 (-0.17-0.25)
25-29 years	7269.21 (6306.86-8238.73)	2.69 (2.33-3.04)	9301.67 (8021.31-10591.95)	2.65 (2.29-3.02)	0.11 (-0.04-0.26)
30-34 years	13842.51 (12049.03-15919.16)	6.09 (5.3-7)	21898.76 (18738.32-25337.76)	5.84 (5-6.76)	-0.1 (-0.16--0.03)
35-39 years	21744.51 (18841-24610.29)	10.24 (8.87-11.58)	31212.48 (26799.73-36383.89)	9.07 (7.79-10.57)	-0.28 (-0.35--0.22)
40-44 years	25633.7 (22536.33-28989.02)	15.29 (13.45-17.3)	40298.09 (35405.33-45605.19)	13.28 (11.66-15.03)	-0.26 (-0.4--0.12)

45-49 years	24195.94 (21356-26937.31)	17.9 (15.8-19.92)	44736.97 (39224.74-51406.75)	15.02 (13.17-17.26)	-0.32 (-0.52--0.12)
50-54 years	25523.02 (22149.91-28709.13)	21.39 (18.56-24.06)	48613.34 (41845.39-56224.35)	17.11 (14.73-19.79)	-0.51 (-0.79--0.24)
55-59 years	23959.25 (20973.59-26894.9)	23.00 (20.13-25.82)	48306.12 (41663.58-55325.66)	19.59 (16.9-22.44)	-0.54 (-0.77--0.3)
60-64 years	18592.48 (16256.86-20693.6)	21.89 (19.14-24.37)	33745.29 (29426.39-38290.7)	17.98 (15.68-20.4)	-0.61 (-0.78--0.43)
65-69 years	13219.18 (11708.61-14894.48)	21.06 (18.66-23.73)	29516.6 (25713.06-33981.62)	17.67 (15.39-20.34)	-0.54 (-0.67--0.41)
70-74 years	9012.43 (7905.81-10271.65)	21.07 (18.48-24.02)	20534.42 (17796.02-23473.51)	17.09 (14.81-19.53)	-0.63 (-0.74--0.52)
75-79 years	5311.61 (4678.92-5897.43)	19.82 (17.46-22.01)	11760.33 (9805.63-13399.88)	15.5 (12.92-17.66)	-0.72 (-0.83--0.61)
80-84 years	2648.35 (2274.87-2975.7)	18.88 (16.22-21.22)	7234.94 (5972.16-8253.94)	15.48 (12.78-17.66)	-0.61 (-0.69--0.53)
85-89 years	1101.4 (924.27-1251.85)	20.56 (17.25-23.37)	3633.53 (2807.43-4214.68)	15.65 (12.09-18.15)	-0.94 (-1.07--0.81)

90-94 years	241.11 (198.94-279.35)	18.04 (14.88-20.9)	1286.89 (969.46-1509.39)	15.69 (11.82-18.4)	-0.49 (-0.62--0.37)
95+ years	44.98 (34.66-54.28)	16.36 (12.6-19.74)	363.01 (252.91-435.55)	15.37 (10.71-18.45)	-0.09 (-0.22-0.03)
All ages	196998.33 (175157.37-218833.15)	6.2 (5.52-6.89)	357665.5 (314247.51-403188.49)	7.74 (6.8-8.72)	0.86 (0.75-0.98)
Ovarian cancer					
15-19 years	1827.85 (1181.11-2471.19)	0.56 (0.36-0.76)	2961.55 (2241.54-3512.59)	0.84 (0.63-0.99)	1.04 (0.89-1.2)
20-24 years	2366.37 (1513.06-3195.28)	0.76 (0.48-1.02)	4200.98 (3227.51-5073.18)	1.21 (0.93-1.47)	1.43 (1.3-1.56)
25-29 years	2367.96 (1531.11-3319.13)	0.87 (0.57-1.23)	4767.08 (3850.67-5584.15)	1.36 (1.1-1.59)	1.39 (1.28-1.5)
30-34 years	2519.16 (1663.42-3372.62)	1.11 (0.73-1.48)	5872.25 (4837.17-6852.24)	1.57 (1.29-1.83)	1.12 (1.05-1.2)
35-39 years	3631.8 (2610.87-4788.89)	1.71 (1.23-2.25)	7661.53 (6467.67-8959.77)	2.23 (1.88-2.6)	0.78 (0.68-0.88)

40-44 years	4616.54 (3544.73-5769.57)	2.75 (2.11-3.44)	10776.03 (9247.38-12573.75)	3.55 (3.05-4.14)	0.65 (0.55-0.75)
45-49 years	5558.29 (4403.18-6922.07)	4.11 (3.26-5.12)	15635.24 (13142.43-18055.91)	5.25 (4.41-6.06)	0.61 (0.53-0.69)
50-54 years	6665.49 (5476.72-8274.64)	5.59 (4.59-6.94)	18666.8 (15646.8-21892.18)	6.57 (5.51-7.71)	0.26 (0.17-0.35)
55-59 years	6735.09 (5577.33-8301.79)	6.47 (5.35-7.97)	19314.58 (16810.6-23008.8)	7.83 (6.82-9.33)	0.4 (0.27-0.53)
60-64 years	5862.26 (4962.53-7073.93)	6.9 (5.84-8.33)	16562.78 (14300.96-19175.47)	8.82 (7.62-10.21)	0.56 (0.47-0.64)
65-69 years	4786.33 (4128.8-5810.49)	7.63 (6.58-9.26)	15773.9 (13454.07-18139.6)	9.44 (8.05-10.86)	0.47 (0.38-0.56)
70-74 years	3453.96 (2954.1-4099.44)	8.08 (6.91-9.58)	11843.19 (10113.77-13652.75)	9.86 (8.42-11.36)	0.45 (0.38-0.52)
75-79 years	2169.6 (1851.01-2528.87)	8.1 (6.91-9.44)	6881.67 (5819.71-8105.85)	9.07 (7.67-10.68)	0.11 (0-0.22)
80-84 years	1193.26 (1006.67-1386.25)	8.51 (7.18-9.88)	4490.55 (3605.98-5193.62)	9.61 (7.72-11.11)	-0.04 (-0.25-0.17)

85-89 years	555.29 (466.33-636.73)	10.37 (8.71-11.89)	2687.36 (2028.84-3149.46)	11.57 (8.74-13.56)	0.11 (-0.19-0.41)
90-94 years	131.06 (106.37-151.1)	9.8 (7.96-11.3)	1063.8 (765.5-1257.77)	12.97 (9.33-15.33)	0.7 (0.4-1.01)
95+ years	19.38 (14.69-22.68)	7.05 (5.34-8.25)	292.69 (192.21-353)	12.4 (8.14-14.95)	1.76 (1.51-2.02)
All ages	54459.7 (44299.59-67005.9)	1.72 (1.4-2.11)	149451.98 (130021.59-171706.89)	3.23 (2.81-3.71)	1.92 (1.86-1.98)
Uterine cancer					
20-24 years	369.19 (186.02-484.88)	0.12 (0.06-0.16)	433.92 (298.85-531.42)	0.13 (0.09-0.15)	0.29 (0.09-0.48)
25-29 years	687.96 (379.48-879.84)	0.25 (0.14-0.33)	1010.35 (697.43-1260.12)	0.29 (0.2-0.36)	0.49 (0.3-0.68)
30-34 years	1160.68 (672.36-1482.13)	0.51 (0.3-0.65)	2328.36 (1643.46-2974.77)	0.62 (0.44-0.79)	0.58 (0.35-0.82)
35-39 years	2592.95 (1632.08-3298.89)	1.22 (0.77-1.55)	4763.14 (3484.64-5922.69)	1.38 (1.01-1.72)	0.38 (0.09-0.66)

40-44 years	3445.66 (2303.6-4286.28)	2.06 (1.37-2.56)	7651.92 (5914.19-9280.32)	2.52 (1.95-3.06)	0.71 (0.41-1.02)
45-49 years	4547.9 (3083.22-5473.59)	3.36 (2.28-4.05)	13806.99 (10660.83-16891.04)	4.64 (3.58-5.67)	1.09 (0.84-1.34)
50-54 years	7620.13 (5547.77-9233.75)	6.39 (4.65-7.74)	25307.63 (19697.53-31552.58)	8.91 (6.93-11.11)	1 (0.78-1.22)
55-59 years	8440.77 (6376.52-10135.02)	8.1 (6.12-9.73)	28232.89 (23133.97-35599.61)	11.45 (9.38-14.44)	1.07 (0.92-1.22)
60-64 years	7097.81 (5596.16-8372.51)	8.36 (6.59-9.86)	21948.91 (17906.24-27602.38)	11.69 (9.54-14.7)	1.16 (1.06-1.26)
65-69 years	5573.83 (4436.66-6539.74)	8.88 (7.07-10.42)	19495.22 (16031.6-24481.68)	11.67 (9.6-14.65)	0.8 (0.71-0.89)
70-74 years	3850.35 (3149.05-4520.16)	9.00 (7.36-10.57)	13200.15 (11073.16-16930.09)	10.98 (9.21-14.09)	0.52 (0.44-0.6)
75-79 years	2418.23 (1977.99-2827.48)	9.03 (7.38-10.55)	7472.62 (6105.71-9651.23)	9.85 (8.05-12.72)	0.14 (0.05-0.22)
80-84 years	1184.84 (953.35-1406.63)	8.45 (6.8-10.03)	4140.2 (3232.88-5203.25)	8.86 (6.92-11.13)	-0.07 (-0.23-0.1)

85-89 years	449.44 (360.17-540.1)	8.39 (6.72-10.08)	2118.95 (1557.36-2626.5)	9.13 (6.71-11.31)	0.17 (-0.03-0.37)
90-94 years	103.61 (82.59-119.57)	7.75 (6.18-8.94)	727.18 (514.91-876.21)	8.86 (6.28-10.68)	0.34 (0.16-0.53)
95+ years	16.14 (11.86-19.94)	5.87 (4.31-7.25)	223.5 (147.22-270)	9.47 (6.24-11.44)	1.52 (1.36-1.68)
All ages	49559.48 (37592.1-58190.73)	1.56 (1.18-1.83)	152861.93 (125810.13-185975.36)	3.31 (2.72-4.02)	2.47 (2.34-2.6)

[a] represents data in parentheses is the 95% uncertainty interval; [b] represents data in parentheses is the 95% confidence interval; EAPC= estimated annual percent change.

Table S4. The global all-age deaths, DALYs, and incident cases attributable to gynecological cancers in 2021 by four world regions.

Region	Deaths	DALYs	Incident cases
**Cervical cancer**			
Africa	63059.64 (53317.58-73982.49)	2330278.84 (1945844.85-2742397.48)	114266.34 (96326.25-134844.34)
America	44258.82 (40721.77-47550.97)	1449519.76 (1345187.47-1557646.35)	124453.86 (116232.05-133233.22)
Asia	160723.84 (143073.14-179396.56)	5318837.66 (4733229.92-5916376.88)	357665.5 (314247.51-403188.49)
Europe	28179.29 (26067.95-29773.5)	799221.18 (752374.45-843491.42)	70030.2 (66251.72-73652.13)
Ovarian cancer			
Africa	13061.63 (10065.88-15302.57)	427246.9 (326380.54-503427.79)	21804.8 (16639.2-25513.63)
America	36170.07 (33288.15-38172.57)	944236.63 (889096.85-990123.33)	55483.2 (51990.73-58307.32)
Asia	84278.89 (73874.6-97734.25)	2568854.39 (2233137.32-2992340.5)	149451.98 (130021.59-171706.89)
Europe	51709.54 (47016.38-54992.96)	1213088.84 (1129201.94-1280901.64)	71570.45 (65877.83-75668.51)
Uterine cancer			
Africa	6038.35 (4668.76-7351.42)	170554.37 (130681.17-210527.16)	15415.41 (11913.12-18580.04)
America	22732.85 (20712.16-24047.02)	584073.81 (543539.91-625271.81)	134430.23 (125376.57-140736.01)
Asia	37508.92 (31519.05-45758.59)	1076127.45 (889738.88-1303328.41)	152861.93 (125810.13-185975.36)
Europe	31092.44 (27778.12-33212.08)	724997.61 (663447.24-778705.35)	169725.73 (156809.84-179578.9)

Table S5. The global ASMR, ASDR, and ASIR per 100,000 population attributable to gynecological cancers in 1990 and 2021 by SDI level and country.

Country	ASMR		ASDR		ASIR	
	1990	2021	1990	2021	1990	2021
**Cervical cancer**						
Global	5.07 (4.7-5.5)	3.44 (3.16-3.73)	167.71 (155-182.38)	115.05 (105.07-125.5)	9.25 (8.65-9.91)	7.79 (7.16-8.48)
Low SDI	12.09 (10.3-14.7)	8.35 (7.11-9.88)	410.67 (350.5-500.84)	271.05 (230.07-323.22)	16.95 (14.44-20.54)	12.94 (10.96-15.29)
Low-middle SDI	7.22 (6.2-8.33)	5.01 (4.48-5.52)	247.65 (214.86-285.46)	163.73 (146.33-181.68)	11.02 (9.56-12.65)	9.07 (8.13-10.05)
Middle SDI	5.36 (4.93-5.82)	3.5 (3.15-3.87)	170.67 (156.69-185.42)	111.59 (100.68-123.48)	9.06 (8.35-9.79)	8.12 (7.28-9.04)
High-middle SDI	3.76 (3.5-4.03)	2.43 (2.13-2.76)	116.99 (108.53-126.09)	78.32 (68.57-89.31)	7.06 (6.57-7.53)	6.76 (5.83-7.72)
High SDI	2.78 (2.65-2.86)	1.37 (1.26-1.44)	85.86 (83.3-88.13)	43.64 (41.53-45.61)	8.53 (8.3-8.71)	5.16 (4.94-5.34)
**Four World Regions**						
Africa	9.89 (8.49-11.54)	8.23 (7.02-9.59)	331.33 (285.19-389.22)	263.97 (222.29-309.21)	14.14 (12.18-16.5)	12.99 (11.02-15.24)
America	4.99 (4.8-5.12)	3.49 (3.22-3.75)	167.43 (162.82-171.47)	119.43 (110.91-128.4)	12.8 (12.51-13.05)	10.42 (9.73-11.17)
Asia	4.91 (4.33-5.5)	3.1 (2.75-3.47)	163.87 (144.52-183.89)	100.99 (89.92-112.21)	8.06 (7.17-8.95)	6.84 (6.02-7.71)
Europe	3.67 (3.54-3.78)	1.99 (1.86-2.1)	112.2 (109.33-115)	64.21 (60.77-67.88)	8.21 (7.99-8.42)	5.9 (5.59-6.21)
**Countries**						
American Samoa	5.27 (4.06-6.64)	5.24 (3.98-6.68)	154.75 (118.62-198.82)	163.39 (123.18-208.96)	9.44 (7.23-12.3)	10.82 (8.11-13.77)
Antigua and Barbuda	6.66 (5.99-7.28)	5.36 (5.02-5.71)	211.31 (191.22-233.11)	157.41 (146.64-169.16)	14.15 (12.64-15.61)	12.31 (11.35-13.34)
Arab Republic of Egypt	0.78 (0.65-0.97)	1.01 (0.74-1.28)	24.85 (20.69-30.27)	29.61 (21.33-38.38)	1.11 (0.93-1.35)	1.67 (1.23-2.18)
Argentine Republic	5.79 (5.37-6.23)	5.17 (4.7-5.69)	200.26 (185.47-213.66)	176.51 (162.59-193.06)	11.64 (10.69-12.53)	12.88 (11.73-14.24)
Australia	2.79 (2.56-3.09)	0.99 (0.88-1.1)	88.14 (81.51-97)	32.33 (29.25-35.75)	8.93 (8.24-9.83)	4.67 (4.18-5.21)
Barbados	10.06 (9.34-10.8)	6.95 (5.53-8.64)	306.4 (283.01-329.13)	207.04 (161.98-259.65)	20.75 (19.19-22.4)	16.7 (13.01-21.18)
Belize	9.33 (8.6-10.15)	8.81 (7.75-10)	305.39 (280.27-332.26)	294.35 (257.23-336.94)	17.95 (16.37-19.62)	19.97 (17.28-22.79)
Bermuda	4.47 (3.99-4.96)	1.77 (1.45-2.25)	129.01 (114.04-144.65)	52.47 (42.51-66.13)	9.88 (8.79-11.15)	5.97 (4.82-7.59)
Bolivarian Republic of Venezuela	10.38 (9.81-10.96)	8.4 (6.17-10.92)	337.18 (318.32-356.32)	286.63 (204.81-373.58)	23.21 (21.87-24.77)	26.32 (18.86-34.61)

Bosnia and Herzegovina	3.54 (3.04-4.09)	2.52 (1.87-3.23)	110.2 (93.8-128.33)	78.4 (56.84-101.75)	7.04 (5.87-8.16)	6.95 (4.98-8.96)
Brunei Darussalam	6.66 (5.25-8.1)	4.02 (3.22-4.9)	212.69 (167.62-260.29)	123.66 (96.97-154.07)	12.65 (9.92-15.43)	8.77 (6.92-10.92)
Burkina Faso	12.98 (9.9-17.16)	10.35 (7.8-13.25)	422.62 (323.51-571.74)	318.57 (234.98-410.61)	18.41 (14.09-24.94)	15.22 (11.29-19.74)
Canada	1.75 (1.62-1.87)	1.2 (1.11-1.3)	53.63 (49.9-57.02)	40.82 (37.43-44.48)	6.36 (5.84-6.87)	6.74 (6.01-7.43)
Central African Republic	21.05 (15.26-27.43)	18.05 (12.18-26)	694.28 (507.4-906.64)	579.57 (382.27-840.74)	27.4 (19.97-35.58)	24.01 (16.12-34.04)
Commonwealth of Dominica	11.69 (10.05-13.7)	8.19 (6.37-10.35)	344.44 (290.94-403.05)	240.17 (182.79-310.99)	20.25 (17.41-23.69)	15.83 (11.96-20.28)
Commonwealth of the Bahamas	8.07 (7.33-8.81)	5.87 (4.66-7.41)	270.36 (244.41-295.89)	196.8 (151.75-250.87)	16.82 (15.08-18.36)	14.36 (11.17-18.12)
Cook Islands	2.97 (2.35-3.75)	1.76 (1.31-2.29)	89.39 (68.17-113.18)	52.37 (38.38-70.28)	5.96 (4.54-7.62)	4.67 (3.45-6.3)
Czech Republic	4.77 (4.34-5.21)	1.98 (1.66-2.36)	149.11 (137.66-161.14)	58.26 (49.24-69.04)	9.52 (8.79-10.31)	4.89 (4.13-5.73)
Democratic People's Republic of Korea	4.58 (3.42-6.31)	4.11 (2.91-5.87)	148.76 (109.42-208.86)	137.91 (94.09-197.23)	8.07 (5.82-11.22)	8.93 (6.11-12.61)
Democratic Republic of Sao Tome and Principe	13.21 (10.6-16.03)	11.84 (8.35-15.58)	422.26 (325.14-520.61)	369.04 (258.51-493.74)	19.66 (15.16-23.87)	20.31 (14.07-27.46)
Democratic Republic of the Congo	14.53 (10.61-19.52)	13.67 (9.12-19.1)	480.63 (352.48-648.91)	423.69 (280.03-601.21)	20.04 (14.71-26.98)	19.86 (13.14-28.02)
Democratic Republic of Timor-Leste	5.52 (3.99-8.05)	4.08 (3.05-5.5)	181.02 (129.72-264.05)	131.24 (95-181.59)	7.99 (5.72-11.52)	6.79 (4.93-9.15)
Democratic Socialist Republic of Sri Lanka	2.9 (2.45-3.37)	1.95 (1.28-2.71)	92.14 (78.52-107.81)	56.91 (35.74-80.24)	5.1 (4.3-5.93)	4.29 (2.71-5.97)
Dominican Republic	6.13 (5.18-7.26)	5.29 (3.86-6.88)	201.12 (169.38-240.47)	170.61 (123.55-224.6)	10.94 (9.15-13.03)	11.29 (8.15-14.98)
Eastern Republic of Uruguay	5.67 (5.23-6.16)	4.7 (4.25-5.16)	191.21 (175.51-206.95)	155.14 (140.71-171.59)	11.93 (10.82-13.02)	12.25 (11.03-13.67)
Federal Democratic Republic of Ethiopia	20.7 (15.62-31.21)	8.84 (6.69-12.37)	694.65 (527.65-1034.87)	277.05 (205.87-396.88)	27.14 (20.44-40.09)	13.36 (9.95-19.17)
Federal Democratic Republic of Nepal	8.42 (5.84-11.6)	4.91 (3.44-6.56)	290.29 (203.31-404.69)	162.12 (110.13-216.48)	12.11 (8.48-16.81)	8.63 (5.88-11.43)
Federal Republic of Germany	3.49 (3.24-3.7)	1.37 (1.23-1.49)	100.08 (94.37-106.15)	43.05 (38.97-46.63)	7.8 (7.32-8.36)	4.66 (4.19-5.15)
Federal Republic of Nigeria	6.8 (5.01-9.05)	6.39 (4.36-8.85)	216.57 (155.68-296.9)	201.82 (130.89-288.28)	9.42 (6.81-12.9)	10 (6.47-14.41)
Federal Republic of Somalia	22.2 (15.01-31.23)	20.65 (14.28-29.14)	756.16 (509.32-1079.11)	666.37 (457.4-962.91)	29.98 (20.3-42.47)	27.8 (19.03-40.06)
Federated States of Micronesia	11.1 (8.27-14.55)	8.65 (6.38-11.76)	355.87 (257.54-471.27)	275.82 (196.05-377.85)	17.66 (13.03-23.42)	15.81 (11.29-22.1)
Federative Republic of Brazil	6.98 (6.63-7.31)	4.44 (4.14-4.69)	217.76 (209.12-226.56)	147.66 (139.02-154.83)	11.92 (11.4-12.39)	10.46 (9.86-11)
French Republic	2.7 (2.52-2.89)	1.32 (1.17-1.45)	76.67 (72.25-81.77)	38.75 (35.27-42.4)	5.97 (5.57-6.45)	4.19 (3.78-4.68)
Gabonese Republic	13.62 (9.87-18.1)	10.52 (6.9-15.35)	428.33 (306.5-569.5)	323.75 (207.77-483.93)	19.07 (13.79-25.14)	17.36 (11.07-26.1)
Georgia	5.47 (4.87-6.04)	4.31 (3.74-4.95)	178.23 (158.14-198.13)	132.94 (113.73-153.3)	11.51 (10.16-12.86)	9.11 (7.79-10.51)

Grand Duchy of Luxembourg	2.48 (2.32-2.65)	0.86 (0.77-0.96)	72.85 (68.03-77.98)	24.07 (21.51-26.75)	5.59 (5.18-6.02)	2.51 (2.23-2.79)
Greenland	9.84 (7.73-11.98)	3.62 (2.83-4.75)	318.5 (252.93-387.3)	126.01 (95.82-166.57)	19.32 (15.15-23.76)	10.76 (7.82-14.47)
Grenada	12.57 (10.91-14.02)	9.36 (8.2-10.81)	418.87 (362.89-468.89)	280.53 (238.12-331.24)	24.03 (20.74-27.04)	19.81 (16.9-23.3)
Guam	4.36 (3.68-4.97)	2.25 (1.86-2.61)	113.57 (97.39-129.88)	79.6 (67.88-91.31)	8.17 (6.98-9.32)	6.24 (5.22-7.18)
Hashemite Kingdom of Jordan	1.81 (1.39-2.4)	0.69 (0.52-0.89)	58.13 (44-77.89)	20.14 (14.77-26.81)	2.92 (2.23-3.87)	1.36 (0.99-1.81)
Hellenic Republic	2.57 (2.4-2.7)	1.47 (1.34-1.59)	73.46 (69.49-77.16)	43.48 (40.34-46.41)	6.41 (6.01-6.79)	4.28 (3.92-4.61)
Hungary	4.79 (4.18-5.45)	2.61 (2.18-3.1)	155.45 (133.64-178.65)	83.07 (68.13-100.34)	11.23 (9.42-13.05)	8.2 (6.56-10.16)
Independent State of Papua New Guinea	8.59 (5.99-13.78)	6.62 (4.67-11.33)	289.23 (197.34-466.42)	220.83 (156.11-381.71)	14.64 (10.29-23.19)	11.72 (8.42-20.01)
Independent State of Samoa	5.92 (4.42-7.61)	5.83 (4.07-7.75)	197.69 (145.17-256.9)	202.28 (136.47-277)	11.16 (8.21-14.62)	13.14 (8.86-17.84)
Ireland	2.52 (2.32-2.73)	1.12 (0.99-1.26)	76.16 (70.06-82.57)	35.64 (31.61-39.57)	6.04 (5.49-6.63)	4.12 (3.61-4.61)
Islamic Republic of Afghanistan	5.9 (3.21-9.49)	4.81 (2.59-7.05)	214.75 (107.93-353.2)	163.49 (84.69-245.32)	8.09 (4.16-13.3)	7.04 (3.72-10.6)
Islamic Republic of Iran	1.28 (1.11-1.56)	0.68 (0.59-0.77)	39.16 (33.64-47.93)	19.81 (17.2-22.4)	1.87 (1.6-2.3)	1.24 (1.07-1.42)
Islamic Republic of Mauritania	12.18 (9.25-15.2)	9.34 (6.58-12.42)	379.54 (283.13-472.7)	286.02 (198.53-388.14)	16.63 (12.47-20.67)	15.48 (10.87-21.17)
Islamic Republic of Pakistan	2.25 (1.84-2.66)	2.25 (1.61-3.01)	77.2 (62.09-91.7)	76.56 (54.25-103.71)	3.37 (2.72-3.98)	3.83 (2.73-5.21)
Jamaica	9.71 (8.95-10.58)	7.23 (5.45-9.29)	304.16 (282.14-330.03)	233.45 (173.61-306.06)	19.9 (18.26-21.75)	17.6 (12.9-23.48)
Japan	2.03 (1.88-2.11)	1.42 (1.29-1.49)	57.05 (54.32-58.98)	49.09 (46.44-51.09)	5.73 (5.43-5.97)	6.2 (5.8-6.57)
Kingdom of Bahrain	2.37 (1.84-3.01)	1.07 (0.82-1.38)	63.99 (49.02-81.78)	26.24 (20.07-34.1)	3.24 (2.51-4.14)	1.81 (1.39-2.34)
Kingdom of Belgium	2.53 (2.32-2.73)	1.27 (1.13-1.41)	73.4 (67.6-79.52)	38.03 (34.59-42.2)	6.04 (5.5-6.6)	4.01 (3.56-4.51)
Kingdom of Bhutan	8.63 (5.57-11.99)	4.31 (2.92-5.84)	288.08 (180.17-395.73)	135.63 (88.94-185.51)	12.24 (7.69-17.06)	7.52 (4.93-10.43)
Kingdom of Cambodia	8.61 (6.73-11.32)	5.36 (4.04-7.44)	295.07 (230.76-384.5)	172.3 (126.73-240.68)	12.71 (9.94-16.66)	9.36 (6.91-13.13)
Kingdom of Denmark	4.83 (4.5-5.18)	1.52 (1.36-1.67)	144.29 (134.7-154.91)	42.93 (38.78-46.63)	10.38 (9.66-11.18)	4.28 (3.85-4.74)
Kingdom of Eswatini	14.04 (9.85-20.13)	20.58 (11.6-31.69)	422.07 (294.5-611.61)	613.79 (332.03-965.34)	19.53 (13.49-28)	30.3 (16.51-47.68)
Kingdom of Lesotho	12.14 (8.46-17.51)	25.75 (15.9-37.21)	355.67 (243.16-513.21)	767.4 (455.2-1135.28)	16.08 (11.06-23.03)	35.27 (20.96-51.22)
Kingdom of Morocco	3.51 (2.69-4.55)	2.91 (2.02-3.85)	119.94 (90.38-156.3)	96.32 (63.41-131.6)	5.2 (3.92-6.74)	5.15 (3.48-6.99)
Kingdom of Norway	2.76 (2.61-2.88)	1.49 (1.34-1.61)	86.38 (82.46-90.3)	42.15 (39.26-44.92)	6.83 (6.45-7.19)	4.48 (4.14-4.82)
Kingdom of Saudi Arabia	0.65 (0.49-0.86)	0.35 (0.26-0.46)	21.06 (15.45-27.9)	11.75 (8.6-15.88)	1.5 (1.1-1.98)	1.39 (1.02-1.91)

Kingdom of Spain	2.2 (2.03-2.36)	1.02 (0.91-1.13)	66.78 (62.35-72.01)	32.3 (29.15-35.68)	6.8 (6.26-7.36)	4.44 (3.98-4.93)
Kingdom of Sweden	2.05 (1.89-2.19)	1.25 (1.07-1.43)	59.84 (55.74-63.89)	33.66 (29.04-38.37)	4.87 (4.5-5.26)	3.32 (2.83-3.85)
Kingdom of Thailand	8.05 (6.49-9.67)	4.43 (3.34-5.7)	255.19 (208.25-305.78)	141.54 (106.9-184.48)	14.63 (11.8-17.43)	11.53 (8.71-15.04)
Kingdom of the Netherlands	1.91 (1.76-2.05)	0.98 (0.88-1.09)	56.41 (52.17-60.79)	29.48 (26.17-33.05)	5.28 (4.82-5.75)	3.68 (3.25-4.16)
Kingdom of Tonga	12.71 (9.91-16.32)	10.08 (7.33-13.14)	393.38 (309.56-512.36)	292.68 (210.22-391.82)	23.29 (18.2-30.04)	19.5 (13.99-26.24)
Kyrgyz Republic	7.53 (6.69-8.44)	4.56 (3.73-5.48)	241.39 (211.13-276.82)	148.63 (119.44-181.49)	13.36 (11.7-15.45)	9.96 (7.87-12.31)
Lao People's Democratic Republic	9.06 (6.57-12.29)	4.48 (3.29-5.89)	315.83 (227.83-428.65)	148.92 (106.85-198.55)	12.98 (9.29-17.7)	7.6 (5.49-9.8)
Lebanese Republic	2.19 (1.7-2.79)	0.89 (0.7-1.1)	72.74 (55.32-93.39)	27.17 (20.86-34.45)	3.83 (2.91-4.91)	1.98 (1.52-2.52)
Malaysia	6.64 (5.49-7.79)	4.7 (3.62-5.48)	200.74 (166.31-229.87)	139.26 (106.68-162.97)	10.75 (8.9-12.38)	9.54 (7.4-11.2)
Mongolia	7.22 (5.22-9.93)	5.46 (4.03-7.18)	235.88 (170.45-324.2)	165.74 (122.27-218.58)	11.01 (7.95-15.11)	9.98 (7.4-13.39)
Montenegro	2.88 (2.34-3.76)	2.45 (1.92-3.04)	97.71 (78.84-128.31)	74.37 (58.57-93.09)	8.44 (6.62-11.08)	7.49 (5.85-9.48)
New Zealand	3.26 (3.02-3.51)	0.84 (0.76-0.93)	109.99 (101.07-119.85)	28.4 (25.56-31.55)	8.4 (7.62-9.26)	2.75 (2.45-3.09)
North Macedonia	3.95 (3.27-4.71)	2.95 (2.2-3.83)	131.79 (109.21-156.33)	86.47 (64.28-113.02)	8.52 (7.06-10.12)	7.43 (5.49-9.73)
Northern Mariana Islands	9.06 (6.82-11.79)	7.39 (5.94-8.58)	269.37 (197.03-355.14)	218.76 (173.32-261.5)	19.11 (13.73-25.29)	16.75 (13.36-20.2)
Palestine	0.94 (0.68-1.26)	0.51 (0.41-0.61)	27.84 (19.85-38.41)	13.92 (11.01-17.17)	1.36 (0.98-1.87)	0.85 (0.67-1.05)
People's Democratic Republic of Algeria	3.46 (2.66-4.36)	1.76 (1.41-2.2)	116.72 (90.13-146.66)	56.65 (44.33-71.64)	5.6 (4.34-7.17)	3.64 (2.82-4.67)
People's Republic of Bangladesh	7.43 (5.24-9.84)	3.84 (2.7-5.31)	264.51 (184.26-353.85)	128.14 (89.33-182.26)	11.08 (7.75-14.86)	7.28 (4.98-10.46)
People's Republic of China	3.56 (2.91-4.35)	2.39 (1.77-3.08)	112.27 (90.48-138.52)	75.05 (54.64-97.84)	5.84 (4.71-7.19)	6.67 (4.8-8.72)
Plurinational State of Bolivia	19.21 (14.04-24.43)	11.59 (7.91-15.97)	598.56 (434.15-773.76)	337.4 (231.27-476.3)	27.19 (19.87-34.95)	19.06 (13.08-26.63)
Portuguese Republic	3.57 (3.3-3.83)	1.62 (1.45-1.79)	109.74 (101.09-118.14)	50.66 (45.88-55.62)	8.8 (8.03-9.5)	6.22 (5.59-6.89)
Principality of Andorra	1.07 (0.72-1.58)	0.62 (0.43-0.9)	33.49 (21.79-50.06)	19.2 (12.77-28.35)	2.98 (1.91-4.46)	2.2 (1.42-3.27)
Principality of Monaco	2.19 (1.55-2.87)	1.53 (1.11-2.04)	67.34 (47.83-90.67)	48.35 (34.37-65.11)	6.21 (4.43-8.41)	5.65 (3.95-7.76)
Puerto Rico	2.91 (2.67-3.18)	1.87 (1.53-2.25)	89.77 (82.41-97.33)	61.26 (49.21-73.95)	6.81 (6.23-7.46)	6.29 (4.99-7.61)
Republic of Albania	2.03 (1.53-2.65)	1.36 (0.93-1.91)	65.19 (47.3-87.48)	42.73 (28.39-62.39)	4.05 (2.95-5.38)	3.93 (2.61-5.8)
Republic of Angola	14.47 (10.35-19.98)	12.52 (8.38-18.12)	483.91 (341.64-674.71)	401.28 (267-584.95)	19.66 (13.7-27.26)	19.19 (12.74-28.01)
Republic of Armenia	5.48 (5.1-5.84)	2.84 (2.51-3.21)	181.74 (170.71-192.59)	83.01 (73.28-95.08)	11.23 (10.49-12)	6.16 (5.39-7.08)

Republic of Austria	3.53 (3.28-3.75)	1.2 (1.06-1.32)	102.56 (95.65-108.98)	34.6 (31.22-37.61)	8.06 (7.46-8.6)	3.46 (3.11-3.82)
Republic of Azerbaijan	3.98 (3.36-4.6)	2.25 (1.74-2.8)	130.35 (109.59-150.51)	69.87 (53.12-87.43)	7.13 (6.05-8.21)	4.61 (3.5-5.81)
Republic of Belarus	4.88 (4.55-5.19)	2.67 (2.15-3.28)	149.49 (138.94-159.97)	91.05 (71.91-113.38)	9.17 (8.49-9.92)	7.27 (5.7-9.13)
Republic of Benin	10.4 (8.3-12.79)	8.9 (6.56-11.27)	338.59 (264.84-423.45)	270.52 (196.23-351.09)	14.54 (11.35-17.96)	13.27 (9.64-17.38)
Republic of Botswana	13.65 (8.74-19.65)	10.41 (7.36-15.9)	412.69 (259.83-605.66)	295.68 (202.34-463.74)	18.98 (12.15-27.44)	15.47 (10.56-24.14)
Republic of Bulgaria	4.55 (4.09-5.1)	4.16 (3.43-4.9)	157.61 (141.87-176.59)	138.74 (113.84-163.39)	11.1 (10-12.41)	11.8 (9.63-13.98)
Republic of Burundi	21.4 (14.85-28.15)	11.37 (7.95-15.89)	730.78 (501.11-976.14)	372.96 (254.51-530.46)	29.34 (20.07-38.61)	16.65 (11.55-23.39)
Republic of Cabo Verde	10.75 (8.73-13.04)	7.27 (5.6-9.28)	318.54 (253.01-385.5)	181.8 (140.52-234.65)	16.44 (13.15-20.12)	11.24 (8.7-14.55)
Republic of Cameroon	12.01 (9.54-14.84)	10.36 (6.98-14.71)	393.56 (308.74-486.38)	321.86 (208.17-459.4)	17.46 (13.62-21.45)	16.13 (10.72-22.73)
Republic of Chad	10.67 (8.06-13.61)	11.37 (8.4-15.01)	348.85 (267.54-442.29)	365.8 (264.71-487.7)	14.67 (11.16-18.57)	16.39 (11.88-21.65)
Republic of Chile	9.33 (8.68-9.97)	3.39 (3.1-3.69)	290.83 (269.7-312.6)	107.91 (98.51-118.41)	17 (15.63-18.4)	9.58 (8.56-10.63)
Republic of Colombia	7.74 (7.23-8.22)	4.25 (3.53-5.08)	245.67 (229.88-261.69)	142.31 (117.43-172.03)	16.75 (15.44-18.01)	15.01 (12.23-18.17)
Republic of Costa Rica	6.34 (5.88-6.81)	3.38 (2.95-3.86)	198.55 (183.56-213.04)	112.92 (97.41-128.48)	17.18 (15.81-18.55)	12.87 (10.99-14.8)
Republic of Croatia	4.07 (3.49-4.64)	1.93 (1.51-2.38)	117.85 (101.18-133.71)	57.11 (44.01-71.33)	10.67 (9.14-12.11)	6.89 (5.29-8.72)
Republic of Cuba	5.49 (5.06-5.92)	3.93 (3.31-4.6)	184.82 (169.3-198.64)	124.67 (104.51-147.61)	13.75 (12.52-14.91)	11.44 (9.44-13.55)
Republic of Cyprus	2.58 (2.1-3.05)	1.28 (1.02-1.55)	67.07 (55.37-80.85)	33.22 (26.57-40.28)	4.71 (3.86-5.65)	3.39 (2.71-4.15)
Republic of C 么 te d'Ivoire	5.62 (4.23-7.2)	4.87 (3.34-6.72)	176.77 (128.03-227.23)	150.73 (99.11-212.87)	7.77 (5.72-9.93)	7.51 (5.02-10.52)
Republic of Djibouti	12.86 (8.87-17.87)	10.28 (6.54-15.37)	417.95 (278.48-593.01)	326.52 (199.14-499.66)	18.19 (12.31-25.72)	16 (9.92-24.19)
Republic of Ecuador	8.9 (8.37-9.45)	5.93 (4.62-7.39)	280.66 (262.74-298.78)	179.54 (137.94-228.24)	14.45 (13.45-15.43)	12.31 (9.42-15.6)
Republic of El Salvador	9.74 (8.77-10.81)	8.89 (6.89-11.15)	320.57 (289.86-354.45)	285.51 (217.52-358.24)	19.91 (17.88-21.92)	26.11 (19.65-33.42)
Republic of Equatorial Guinea	17.8 (12.03-25.32)	11.45 (6.83-18.09)	592.12 (390.31-857.8)	359.67 (201.8-578.16)	23.78 (15.9-33.84)	19.66 (11.1-31.41)
Republic of Estonia	5.74 (5.27-6.26)	2.63 (2.24-3.05)	176.36 (162.5-191.16)	77.68 (65.53-90.13)	13.42 (12.23-14.64)	8.23 (6.97-9.57)
Republic of Fiji	14.32 (10.92-17.83)	12.1 (8.55-15.78)	462.49 (342.54-585.81)	360.6 (251.17-482.76)	26.15 (19.55-32.92)	21.98 (15.33-29.37)
Republic of Finland	1.65 (1.51-1.8)	0.72 (0.63-0.8)	45.16 (41.37-49.38)	22.03 (19.65-24.42)	3.8 (3.46-4.17)	2.73 (2.41-3.06)
Republic of Ghana	10.4 (7.9-13.11)	10.12 (7.03-13.25)	352.71 (263.67-458.1)	309.21 (210.63-406.6)	15.97 (11.86-20.43)	16.08 (11.06-21.01)
Republic of Guatemala	8.09 (7.7-8.48)	7.93 (6.85-9.22)	251.44 (238.55-264.46)	252.76 (215.68-295.75)	14.09 (13.26-14.96)	18.58 (15.93-21.8)

Republic of Guinea	16.74 (12.36-20.85)	14.15 (10.26-18.9)	580.19 (431.65-728.68)	484.22 (342.29-666.12)	24.16 (18.03-30.2)	22.28 (16-30.14)
Republic of Guinea-Bissau	16.75 (12.21-21.87)	17.44 (12.25-22.2)	587.08 (427.58-774.8)	565.28 (395.41-734)	23.48 (16.96-30.33)	25.41 (17.85-32.63)
Republic of Guyana	12.64 (10.95-14.66)	8.94 (6.77-11.63)	413.39 (353.33-487.36)	292.3 (216.46-390.34)	20.53 (17.61-24.03)	16.92 (12.33-23.01)
Republic of Haiti	22.72 (16.44-30.09)	14.82 (10.16-20.45)	782.67 (565.42-1037.86)	497.8 (338.92-694.09)	33.67 (24.15-44.26)	24.1 (16.32-33.68)
Republic of Honduras	7.32 (5.73-9.33)	8.38 (5.38-11.5)	258.55 (199.2-339.17)	261.64 (158.96-367.03)	14.86 (11.24-19.55)	17.96 (11.11-25.73)
Republic of Iceland	2.22 (2.01-2.43)	0.95 (0.82-1.07)	68.49 (62.05-75.22)	28.18 (24.78-31.75)	6.19 (5.57-6.9)	3.39 (2.9-3.94)
Republic of India	8.19 (6.74-9.62)	4.71 (4.06-5.41)	281.39 (233.34-328.69)	152.25 (130.46-175.71)	12.13 (10.02-14.16)	8.29 (7.1-9.53)
Republic of Indonesia	4.9 (3.76-6.18)	3.79 (2.77-4.89)	169.54 (128.78-213.3)	123.46 (89.37-162.9)	7.95 (6.06-10.01)	6.97 (5.09-9.16)
Republic of Iraq	1.2 (0.91-1.58)	0.93 (0.67-1.25)	40.26 (30.19-53.62)	28.6 (20.48-38.53)	1.87 (1.41-2.47)	1.72 (1.23-2.34)
Republic of Italy	0.99 (0.93-1.03)	1.14 (1.02-1.24)	29.69 (28.39-30.89)	33.98 (31.51-36.08)	2.65 (2.5-2.8)	4.33 (4.01-4.64)
Republic of Kazakhstan	6.91 (6.29-7.59)	3.31 (2.82-3.86)	210.12 (193.44-228.12)	114.31 (97.3-132.81)	12.13 (11.15-13.14)	8.67 (7.34-10.05)
Republic of Kenya	6.35 (4.59-9.59)	6.43 (4.56-8.65)	207.74 (150.54-319.79)	200.43 (141.78-275.41)	9.33 (6.83-14.03)	10.02 (7.04-13.62)
Republic of Kiribati	27.65 (20.35-35.13)	26.1 (18.61-33.82)	860.21 (628.42-1103.77)	774.47 (553.11-1035.34)	39.8 (29.01-50.53)	39.05 (27.97-51.86)
Republic of Korea	3.77 (3.18-4.51)	1.23 (0.96-1.57)	121.9 (102.12-146.83)	36.02 (28.81-46.49)	8.16 (6.93-9.78)	4.48 (3.61-5.78)
Republic of Latvia	4.8 (4.46-5.2)	2.74 (2.35-3.17)	147.84 (136.25-160.62)	80.57 (68.15-93.27)	8.21 (7.52-8.98)	5.37 (4.49-6.26)
Republic of Liberia	9.61 (7.53-12.29)	10.23 (7.1-14.07)	311.99 (237.96-408)	323.37 (222.35-449.37)	13.28 (10.15-17.11)	16.31 (11.28-22.38)
Republic of Lithuania	5.33 (4.98-5.74)	3.35 (2.87-3.83)	165.04 (153.43-177.6)	100.25 (84.48-115.27)	12.59 (11.58-13.73)	9.12 (7.82-10.61)
Republic of Madagascar	15.09 (10.44-18.92)	11.9 (7.35-16.39)	532.64 (367.99-670.52)	404.76 (250.8-562.57)	22.28 (15.48-27.96)	18.74 (11.46-26.63)
Republic of Malawi	15.17 (11.7-19.46)	14.87 (9.98-20.42)	514.77 (393.7-653.89)	493.65 (324.11-687.48)	21.62 (16.56-27.68)	23.66 (15.61-32.84)
Republic of Maldives	5 (3.12-6.93)	1.17 (0.9-1.5)	177.43 (102.72-246.92)	32.73 (24.77-41.85)	8.59 (5.02-11.85)	2.42 (1.85-3.06)
Republic of Mali	12.61 (9.93-15.4)	8.76 (6.55-11.46)	438.84 (343.08-530.9)	296.45 (222.07-391.13)	18.47 (14.5-22.16)	14.12 (10.57-18.7)
Republic of Malta	1.97 (1.79-2.16)	0.91 (0.79-1.03)	57.83 (52.16-63.32)	27.06 (23.54-30.94)	4.4 (3.96-4.82)	2.81 (2.43-3.2)
Republic of Mauritius	5.24 (4.89-5.6)	3.15 (2.84-3.34)	158.27 (147.88-169.55)	97.24 (86.87-103.55)	9.3 (8.64-10.05)	6.89 (6.16-7.38)
Republic of Moldova	6.62 (6.19-7.05)	3.62 (3.24-4.07)	211.42 (196.42-226.09)	119.98 (106.33-135.71)	11.78 (10.92-12.66)	8.14 (7.13-9.34)
Republic of Mozambique	16.56 (12.65-22.01)	17.41 (10.79-27.82)	565.11 (421.14-764.8)	563.63 (342.7-912.82)	23.28 (17.56-31.15)	25.54 (15.72-40.51)
Republic of Namibia	7.19 (5.19-9.78)	9.13 (6.16-12.34)	223.89 (160.89-306)	278.27 (178.69-386.57)	10.19 (7.4-13.99)	14.83 (9.6-20.87)

Republic of Nauru	10.96 (8.18-14.63)	11.5 (7.41-15.58)	381.62 (278.09-520.22)	382.55 (240.28-536.74)	19.93 (14.3-26.78)	22.16 (13.9-30.81)
Republic of Nicaragua	11.09 (9.46-12.86)	6.51 (5.23-8.36)	366.9 (309.82-429.05)	205.09 (160.89-264.71)	24.18 (20.42-28.49)	17.77 (13.86-22.93)
Republic of Niue	7.58 (5.72-10.17)	5.69 (4.38-7.19)	224.76 (165.73-313.12)	175.2 (135.67-221.8)	13.39 (9.9-18.59)	12.52 (9.65-15.7)
Republic of Palau	20.08 (15.07-26.47)	13.85 (10.9-17.35)	587.93 (438.52-777.25)	371.57 (288.82-476.47)	35.77 (26.82-46.83)	25.75 (19.95-33.18)
Republic of Panama	9.26 (8.59-9.96)	4.32 (3.42-5.24)	313.09 (287.79-339.94)	145.79 (114.84-176.54)	24.53 (22.48-26.89)	15.75 (12.52-19.21)
Republic of Paraguay	9.34 (7.57-11.42)	8.34 (6.22-11.11)	307.85 (250.22-379.35)	263.03 (194.9-351.04)	17.46 (14.24-21.15)	18.02 (13.26-24.64)
Republic of Peru	9.21 (7.69-10.9)	6.83 (4.93-9)	295.37 (245.44-348.76)	210.67 (151.41-278.2)	15.26 (12.61-18.19)	15.81 (11.27-20.99)
Republic of Poland	6.3 (6.05-6.54)	2.88 (2.55-3.18)	197.64 (191.14-204.6)	81.07 (71.54-89.95)	10.51 (10.06-10.99)	5.6 (4.93-6.25)
Republic of Rwanda	26.18 (18.05-33.37)	12.21 (8.05-17.99)	916.33 (630.09-1177.87)	386.99 (251.07-576.04)	36.15 (25.05-46.11)	18.77 (12.29-27.38)
Republic of San Marino	0.99 (0.76-1.27)	0.47 (0.29-0.73)	28.23 (21.58-36.52)	14.52 (8.65-23.38)	2.64 (1.97-3.4)	1.63 (0.92-2.62)
Republic of Senegal	10 (7.94-12.69)	10.21 (7.39-13.73)	332.12 (262.92-421.29)	314.86 (225.57-425.08)	14.61 (11.48-18.37)	15.76 (11.35-21.22)
Republic of Serbia	6.41 (5.1-8.18)	4 (3.13-5.07)	202.83 (161.86-253.43)	124.81 (95.15-159.52)	13.52 (10.7-16.87)	10.82 (8.32-13.81)
Republic of Seychelles	11.61 (9.99-13.35)	6.68 (5.56-7.86)	373.02 (320.15-431.57)	203.45 (169.04-239.14)	20.62 (17.42-23.82)	13.87 (11.53-16.42)
Republic of Sierra Leone	8.37 (6.35-10.93)	10.32 (7.73-13.9)	266.87 (194.42-357.53)	326.03 (234.06-445.44)	11.65 (8.58-15.51)	15.78 (11.46-20.97)
Republic of Singapore	3.46 (3.21-3.7)	1.09 (0.97-1.2)	106.93 (98.77-115.93)	31.2 (28.05-34.49)	8.23 (7.52-8.98)	3.54 (3.19-3.91)
Republic of Slovenia	2.74 (2.47-3.02)	1.27 (1.04-1.49)	86.17 (78.12-96.01)	39.65 (31.71-47.74)	14.7 (12.96-16.77)	9.86 (7.84-11.91)
Republic of South Africa	8.52 (7.25-11.35)	12.04 (10.59-13.66)	296.89 (250.4-383.89)	379.77 (333.61-431.56)	15.21 (12.78-19.57)	21.6 (18.89-24.57)
Republic of South Sudan	12.16 (8.18-17.4)	13.71 (9.39-19.79)	413.21 (276.69-606.21)	465.77 (312.09-681.04)	17.45 (11.73-25.25)	21.51 (14.48-31.2)
Republic of Sudan	2.08 (1.51-3.07)	1.28 (0.84-1.79)	72.09 (50.98-107.5)	43.1 (26.24-62.95)	2.99 (2.08-4.43)	2.22 (1.37-3.29)
Republic of Suriname	11.11 (9.16-13.04)	7.92 (5.84-10.05)	361.96 (294.19-432.62)	264.86 (191.72-340.17)	19.68 (15.78-23.62)	16.66 (12.17-21.47)
Republic of Tajikistan	3.65 (2.83-4.45)	1.93 (1.39-2.66)	120.24 (92.75-148.21)	61.29 (41.96-87.46)	6.25 (4.84-7.63)	3.49 (2.39-5.01)
Republic of the Congo	20.87 (14.13-27.85)	14.7 (8.83-21.76)	683.75 (451.04-928.25)	482.48 (278.56-732.09)	28.68 (18.82-38.77)	23.82 (13.95-35.48)
Republic of the Gambia	7.29 (5.58-9.28)	8.35 (5.92-11.15)	239.26 (176.39-315.19)	276.94 (188.58-378.67)	10.92 (8.06-14.22)	13.97 (9.56-18.77)
Republic of the Marshall Islands	10.17 (8.24-12.69)	8.98 (5.9-12.1)	324.93 (257.45-410.49)	307.99 (192.81-423.42)	16.34 (12.98-20.48)	16.69 (10.52-23.04)
Republic of the Niger	10.81 (8.08-14.68)	10.71 (7.75-14.94)	353.58 (263.22-479.64)	332.14 (235.43-469.01)	14.73 (10.96-19.99)	15.24 (10.82-21.42)
Republic of the Philippines	3.39 (2.94-4.2)	3.38 (2.65-4.22)	118.06 (101.94-142.39)	114.86 (87.99-146.1)	6.15 (5.3-7.49)	6.57 (5-8.39)

Republic of the Union of Myanmar	7.91 (5.83-10.52)	3.92 (2.83-5.55)	282.43 (205.96-377.2)	129.96 (92.93-181.09)	12.2 (8.84-16.24)	7.1 (5.06-9.74)
Republic of Trinidad and Tobago	9.43 (8.79-10.08)	5.98 (4.56-7.64)	289 (267.78-310.31)	197.78 (147.35-258.26)	17.11 (15.8-18.56)	14.55 (10.84-19.09)
Republic of Tunisia	2.07 (1.63-2.57)	1.37 (0.95-1.83)	67.9 (53.39-84.81)	43.28 (29.93-58.91)	3.54 (2.78-4.53)	2.97 (2.06-4.03)
Republic of Turkey	2.01 (1.57-2.62)	0.98 (0.76-1.26)	66.05 (52.15-86.26)	28.2 (21.47-36.12)	3.1 (2.44-4.04)	1.91 (1.47-2.45)
Republic of Uganda	13.45 (9.79-17.85)	12.18 (8.64-16.28)	432.18 (306.3-581.3)	385.72 (265.03-523.4)	18.89 (13.74-25.2)	19.02 (13.29-25.55)
Republic of Uzbekistan	4.38 (3.99-4.78)	3.8 (3.15-4.57)	144.9 (133-157.27)	127.08 (104.85-152.99)	8.08 (7.33-8.77)	7.84 (6.36-9.37)
Republic of Vanuatu	7.51 (5.25-11.49)	7.08 (5.31-9.26)	244.65 (162.37-375.73)	231.12 (171.69-302.79)	12.45 (8.4-19.09)	12.35 (9.11-16.38)
Republic of Yemen	2.29 (1.54-3.83)	1.65 (1.14-2.35)	72.86 (47.86-121.95)	52.01 (34.22-74.63)	3.07 (2.04-5.29)	2.49 (1.63-3.63)
Republic of Zambia	18.38 (14.61-23.34)	14.36 (9.42-24.67)	654.76 (519.35-836.54)	471.09 (304.52-855.73)	27.07 (21.14-34.69)	22.81 (14.64-41.24)
Republic of Zimbabwe	14.54 (10.62-18.32)	22.25 (15.48-30.71)	438.29 (323.19-562.8)	713.01 (481.63-1007.84)	21.01 (15.63-26.58)	33.22 (22.61-45.61)
Romania	7.18 (6.62-7.75)	5.59 (4.89-6.35)	251.14 (231.61-270.67)	179.63 (156.37-204.56)	15.27 (13.94-16.61)	14.27 (12.21-16.4)
Russian Federation	4.05 (3.92-4.17)	3.48 (3.1-3.85)	117.09 (113.86-120.19)	124.05 (109.37-137.9)	7.32 (7.11-7.52)	10.39 (9.17-11.54)
Saint Kitts and Nevis	15.94 (14.54-17.4)	7.13 (5.98-8.52)	520.74 (474-572.54)	195.86 (160.2-239.33)	28.45 (25.93-31.37)	13.73 (11.31-16.92)
Saint Lucia	13.21 (12.16-14.34)	6.56 (5.28-7.86)	419.11 (384.89-456.91)	205.28 (162.87-248.89)	24.5 (22.36-26.77)	14.98 (11.99-18.34)
Saint Vincent and the Grenadines	15.7 (14.19-17.17)	9.13 (7.93-10.57)	499.02 (449.48-544.82)	306.19 (263.84-357.47)	29.39 (26.36-32.14)	20.79 (17.77-24.22)
Slovak Republic	4.43 (3.61-5.37)	2.63 (1.96-3.4)	148.76 (122.32-180.49)	82.87 (62.19-105.54)	10.65 (8.75-12.83)	7.44 (5.6-9.54)
Socialist Republic of Viet Nam	5.49 (4.26-7.15)	4.29 (3.33-5.54)	176.49 (134.9-232.31)	124.37 (93.92-163.59)	9.29 (7.1-12.17)	8.79 (6.69-11.54)
Solomon Islands	9.26 (5.8-14)	9.15 (6.8-12.63)	308.66 (169.41-481.01)	309.82 (224.47-442.48)	14.78 (8.2-22.74)	16.28 (11.86-22.91)
State of Eritrea	24.7 (15.37-32.31)	18.7 (11.86-26.47)	828.99 (521.38-1094.5)	595.07 (370.52-857.89)	33.24 (21.26-43.92)	26.71 (16.76-38.74)
State of Israel	1.88 (1.74-2.04)	1.06 (0.92-1.17)	55.84 (51.54-60.6)	29.08 (26.06-31.98)	4.1 (3.73-4.53)	2.78 (2.46-3.11)
State of Kuwait	1.66 (1.51-1.81)	0.39 (0.33-0.46)	50.35 (45.82-54.55)	11.52 (9.73-13.59)	3.14 (2.88-3.44)	0.93 (0.78-1.1)
State of Libya	3.19 (2.34-4.37)	2.99 (2.19-4.03)	104.59 (77.14-148.18)	97.67 (70.17-132.25)	5.28 (3.9-7.49)	5.94 (4.25-8.2)
State of Qatar	1.52 (1.19-1.94)	0.77 (0.59-0.99)	38.52 (30.27-49.3)	17.54 (13.23-23.38)	2.1 (1.65-2.75)	1.3 (0.98-1.72)
Sultanate of Oman	1.74 (1.24-2.54)	0.72 (0.55-0.92)	52.21 (36.79-78.28)	19.49 (14.88-24.71)	2.61 (1.82-3.86)	1.33 (1.01-1.68)
Swiss Confederation	2.46 (2.23-2.67)	0.81 (0.69-0.9)	70.26 (64.73-75.49)	23.4 (21.14-25.87)	6.24 (5.68-6.8)	2.73 (2.44-3.05)
Syrian Arab Republic	1.08 (0.83-1.42)	0.72 (0.5-0.98)	36.07 (27.3-47.88)	22.47 (15.28-30.95)	1.73 (1.31-2.27)	1.46 (1-2.02)

Taiwan (Province of China)	6.23 (5.75-6.71)	1.96 (1.71-2.21)	189.13 (175.38-203.05)	55.84 (49.23-62.2)	13.97 (12.98-15.01)	5.64 (4.94-6.29)
Togolese Republic	11.11 (8.89-13.58)	11.69 (8.07-15.49)	371.33 (293.35-460.6)	357.28 (234.94-478.61)	16.5 (13.08-20.42)	17.91 (11.84-24.19)
Tokelau	10.36 (7.69-14.01)	6.42 (4.86-8.3)	338.07 (242.42-466.64)	215.05 (161.19-277.52)	18.17 (13.25-25)	14.86 (11.17-19.01)
Turkmenistan	4.99 (4.57-5.42)	3.95 (2.96-5.26)	164.03 (150.46-177.65)	134.5 (99.61-179.96)	8.82 (8.07-9.58)	8.41 (6.23-11.07)
Tuvalu	13.78 (10.83-18.14)	8.1 (6.18-10.58)	454.56 (353.26-603.72)	256.35 (190.21-340.56)	22.29 (17.44-29.62)	15.13 (11.27-19.95)
Ukraine	6.18 (5.75-6.64)	2.07 (1.32-3)	186.6 (172.78-200.28)	65.42 (40.01-96.9)	11.61 (10.73-12.56)	4.41 (2.72-6.63)
Union of the Comoros	17.74 (12.08-24.61)	12.7 (8.35-18.09)	610.82 (395.98-852.64)	412.7 (273.32-591)	25.87 (17.02-35.77)	19.81 (13.18-27.79)
United Arab Emirates	4.85 (3.56-6.82)	2.51 (1.82-3.38)	129.94 (96.21-182.56)	56.02 (41.55-73.59)	6.29 (4.61-8.85)	3.43 (2.57-4.44)
United Kingdom of Great Britain and Northern Ireland	3.32 (3.19-3.4)	1.16 (1.08-1.2)	109.24 (106.41-112.66)	38.36 (37-39.83)	15.62 (15.17-16.09)	5.3 (5.07-5.55)
United Mexican States	12.41 (12.03-12.73)	4.19 (3.54-4.85)	365.05 (356.09-373.65)	134.81 (112.78-156.98)	24.24 (23.67-24.77)	12.1 (10.07-14.13)
United Republic of Tanzania	15.15 (11.21-19.56)	11.03 (7.81-14.93)	506.04 (370.99-653.37)	354.49 (243.03-490.09)	22.11 (16.32-28.78)	17.34 (12.01-23.36)
United States of America	2.08 (1.99-2.15)	1.41 (1.33-1.48)	71.72 (69.44-74.01)	48.48 (46.44-50.6)	10.6 (10.32-10.86)	6.47 (6.22-6.73)
United States Virgin Islands	6.75 (5.37-8.22)	2.57 (1.85-3.51)	211.68 (166.61-259.73)	84.73 (59.17-118.15)	14.18 (11.12-17.47)	6.91 (4.77-9.83)
**Ovarian cancer**						
Global	2.58 (2.39-2.78)	2.16 (1.95-2.34)	69.19 (63.48-75.9)	59.51 (54.03-64.66)	3.82 (3.51-4.15)	3.48 (3.15-3.78)
Low SDI	1.31 (0.88-1.86)	1.92 (1.47-2.27)	38.5 (26.01-55.2)	55.18 (41.97-65.68)	1.81 (1.22-2.58)	2.76 (2.1-3.27)
Low-middle SDI	1.2 (0.95-1.6)	1.93 (1.68-2.29)	36.17 (28.54-48.81)	57.09 (49.49-67.21)	1.82 (1.43-2.47)	3.09 (2.68-3.63)
Middle SDI	1.37 (1.21-1.6)	1.66 (1.45-1.86)	41.37 (35.94-48.93)	50.02 (43.87-56.01)	2.18 (1.89-2.58)	2.97 (2.6-3.32)
High-middle SDI	2.76 (2.54-2.94)	2.25 (1.98-2.5)	79.84 (72.3-86.03)	62.48 (55-69.93)	4.23 (3.85-4.55)	3.62 (3.2-4.06)
High SDI	4.27 (4.02-4.43)	2.72 (2.46-2.88)	112.3 (107.73-115.85)	69.15 (64.56-72.47)	6.52 (6.22-6.74)	4.36 (4.05-4.58)
**Four World Regions**						
Africa	1.25 (0.9-1.57)	1.89 (1.47-2.21)	35.44 (26.07-45.99)	53.34 (41.01-62.66)	1.72 (1.26-2.19)	2.71 (2.09-3.16)
America	3.5 (3.28-3.61)	2.72 (2.51-2.87)	91.99 (88.53-94.35)	73.61 (69.3-77.22)	5.35 (5.09-5.49)	4.35 (4.08-4.57)
Asia	1.47 (1.25-1.75)	1.64 (1.44-1.91)	43.73 (35.98-53.57)	48.94 (42.61-57.02)	2.3 (1.89-2.81)	2.9 (2.52-3.33)
Europe	4.34 (4.13-4.5)	3.31 (3.03-3.5)	118.72 (114.22-122.57)	86.08 (80.81-90.91)	6.6 (6.32-6.81)	5.07 (4.75-5.35)

Countries						
American Samoa	0.98 (0.64-1.26)	1.58 (0.82-2.3)	27.61 (17.69-36.25)	45.74 (23.07-67.62)	1.85 (1.16-2.42)	3.27 (1.68-4.87)
Antigua and Barbuda	2.75 (2.43-3.09)	3.67 (3.41-3.95)	76.83 (68.98-85.25)	98.29 (91.2-105.94)	4.13 (3.72-4.56)	5.44 (5.04-5.88)
Arab Republic of Egypt	0.58 (0.4-1.35)	1.3 (1.01-1.63)	16.82 (12.1-36.39)	36.98 (28.3-46.84)	0.84 (0.61-1.83)	1.98 (1.52-2.51)
Argentine Republic	3.62 (3.24-4.04)	2.76 (2.51-3)	99.87 (90.18-110.84)	76.19 (69.55-82.62)	5.18 (4.66-5.77)	4.23 (3.86-4.6)
Australia	5.39 (4.93-5.77)	2.5 (2.18-2.75)	139.76 (128.06-148.48)	60.9 (54.67-66.14)	7.94 (7.26-8.51)	3.76 (3.38-4.11)
Barbados	3.71 (3.38-4.1)	3.6 (2.87-4.45)	103.1 (93.05-114.21)	97.93 (77.42-123.57)	5.57 (5.04-6.17)	5.55 (4.42-6.99)
Belize	0.9 (0.78-1.03)	1.3 (1.15-1.46)	27.28 (23.38-31.72)	40 (35.44-45.33)	1.39 (1.19-1.59)	2.11 (1.86-2.39)
Bermuda	5.24 (4.25-6.32)	3.69 (3.04-4.62)	136.51 (111.56-165.15)	95.1 (77.77-118.48)	7.61 (6.22-9.19)	5.88 (4.79-7.43)
Bolivarian Republic of Venezuela	0.71 (0.66-0.76)	2.36 (1.78-3.06)	20.48 (19.25-21.79)	70.51 (51.57-93.13)	1.09 (1.03-1.17)	3.89 (2.86-5.13)
Bosnia and Herzegovina	2.62 (2.16-3.16)	3.18 (2.22-4.12)	76.29 (62.73-90.15)	86.13 (60.15-112)	3.71 (3.06-4.41)	4.44 (3.09-5.8)
Brunei Darussalam	3.25 (2.5-4.25)	3.47 (2.7-4.24)	98.62 (75.12-133.7)	102.84 (79.23-127.29)	4.76 (3.63-6.36)	5.68 (4.4-6.99)
Burkina Faso	0.77 (0.44-1.03)	1.07 (0.68-1.49)	22.12 (12.38-29.87)	30.22 (18.8-42.37)	1.05 (0.58-1.4)	1.49 (0.94-2.06)
Canada	3.59 (3.33-3.78)	2.78 (2.5-3.03)	95.1 (88.71-100.44)	68.89 (63.3-74.26)	5.49 (5.11-5.79)	4.3 (3.92-4.64)
Central African Republic	1.2 (0.82-1.95)	1.52 (0.99-2.2)	34.04 (23.09-58.55)	42.08 (27.85-63.08)	1.57 (1.06-2.68)	1.99 (1.31-2.97)
Commonwealth of Dominica	1.99 (1.22-2.62)	1.81 (1.33-2.27)	50.34 (33.47-65.88)	48.4 (35.32-61.47)	2.63 (1.7-3.45)	2.63 (1.93-3.33)
Commonwealth of the Bahamas	4 (3.47-4.76)	4.41 (3.56-5.37)	120.93 (103.85-145.69)	132.04 (104.97-163.46)	6.26 (5.43-7.5)	7.23 (5.76-8.93)
Cook Islands	0.52 (0.37-0.7)	0.74 (0.49-1.03)	14.57 (10.08-19.67)	20.94 (13.9-29.05)	1.01 (0.68-1.38)	1.66 (1.09-2.33)
Czech Republic	4.34 (4.03-4.72)	3.64 (3.09-4.22)	122.63 (114.41-132.24)	94.55 (80.99-108.63)	6.82 (6.33-7.36)	5.83 (4.99-6.76)
Democratic People's Republic of Korea	1.33 (0.84-1.91)	1.54 (1.02-2.21)	41.22 (26.2-60.31)	47.54 (31.64-67.76)	2.84 (1.77-4.07)	3.73 (2.44-5.28)
Democratic Republic of Sao Tome and Principe	1.35 (1.01-1.65)	2.08 (1.29-3.14)	39.49 (28.38-49.99)	61.93 (37.62-94.89)	1.88 (1.38-2.35)	3.16 (1.9-4.73)
Democratic Republic of the Congo	1.1 (0.69-1.71)	1.69 (0.85-2.48)	32.24 (20.49-51.37)	46.82 (24.31-68.8)	1.5 (0.94-2.34)	2.29 (1.2-3.32)
Democratic Republic of Timor-Leste	1.03 (0.57-1.75)	1.8 (1.17-2.48)	31.56 (17.31-55.15)	56.17 (36.36-78.26)	1.87 (1.03-3.34)	3.81 (2.4-5.33)
Democratic Socialist Republic of Sri Lanka	1.16 (0.97-1.46)	1.72 (1.04-2.37)	36.45 (29.96-46.25)	50.58 (29.78-71.45)	2.49 (2.05-3.13)	3.93 (2.41-5.5)
Dominican Republic	0.9 (0.71-1.13)	1.2 (0.91-1.53)	27.63 (22.01-35.4)	35.62 (27-45.27)	1.39 (1.11-1.78)	1.9 (1.45-2.4)
Eastern Republic of Uruguay	3.55 (3.19-3.94)	3.5 (3.15-3.84)	97.5 (87.47-108.12)	94.91 (85.56-103.69)	5.17 (4.63-5.73)	5.37 (4.86-5.87)

Federal Democratic Republic of Ethiopia	1.88 (0.71-3.52)	2.58 (1.28-3.68)	54.17 (22.68-103.96)	74.15 (36.52-105.56)	2.49 (1.02-4.79)	3.68 (1.84-5.24)
Federal Democratic Republic of Nepal	1.2 (0.66-1.85)	1.93 (1.32-3.28)	35.22 (20-55.29)	56.22 (37.47-97.06)	1.66 (0.93-2.61)	2.89 (1.95-4.99)
Federal Republic of Germany	5.84 (5.32-6.29)	3.09 (2.71-3.4)	146.86 (134.59-157.01)	75.82 (67.98-82.08)	8.82 (8.08-9.47)	4.76 (4.27-5.17)
Federal Republic of Nigeria	0.9 (0.53-1.25)	1.59 (0.93-2.28)	23.37 (13.74-33.32)	43.88 (25.39-63.59)	1.15 (0.68-1.62)	2.19 (1.26-3.18)
Federal Republic of Somalia	1.74 (0.99-3.09)	2.27 (1.46-3.57)	50.36 (27.76-92.14)	63.75 (39.98-102.85)	2.32 (1.28-4.25)	3 (1.92-4.79)
Federated States of Micronesia	1 (0.62-1.51)	1.66 (0.89-2.45)	28.74 (17.78-44.68)	47.73 (24.62-72.35)	1.77 (1.07-2.77)	3.23 (1.65-4.89)
Federative Republic of Brazil	2 (1.89-2.07)	2.19 (2.01-2.32)	57.82 (55.45-59.94)	62.94 (59.16-66.26)	2.95 (2.83-3.06)	3.46 (3.24-3.64)
French Republic	4.52 (4.16-4.8)	2.97 (2.58-3.33)	115.39 (107.16-122.27)	70.17 (62.7-77.74)	6.97 (6.44-7.4)	4.76 (4.28-5.25)
Gabonese Republic	1.61 (1.17-2.24)	2.2 (1.44-3.11)	45.19 (33.17-63.8)	61.36 (39.65-89.31)	2.17 (1.59-3.05)	3.16 (2.04-4.55)
Georgia	1.14 (0.91-1.42)	5.28 (4.47-6.11)	35.04 (27.72-43.91)	148.69 (124.56-172.53)	1.66 (1.31-2.09)	7.36 (6.25-8.51)
Grand Duchy of Luxembourg	6.4 (5.97-6.85)	3.46 (3.08-3.84)	161.06 (150.98-172.11)	81.47 (73.33-90.38)	9.17 (8.59-9.83)	5.01 (4.49-5.56)
Greenland	6.48 (5.42-8.1)	3.61 (2.72-4.87)	172.43 (144.48-207.23)	109.41 (80.6-151.28)	8.95 (7.5-10.86)	5.91 (4.41-8.16)
Grenada	3.32 (2.55-4.41)	4.5 (3.95-5.04)	99.51 (75.39-133.1)	122.46 (106.21-139.89)	5.11 (3.9-6.78)	6.74 (5.89-7.62)
Guam	1.26 (1.01-2.05)	1.47 (1.13-1.84)	32.85 (26.41-54.37)	50.65 (38.43-60.89)	2.35 (1.88-3.89)	3.75 (2.86-4.5)
Hashemite Kingdom of Jordan	1.26 (0.8-1.73)	1.5 (0.94-2)	36.18 (22.97-49.72)	41.4 (25.96-54.71)	1.86 (1.18-2.53)	2.33 (1.45-3.12)
Hellenic Republic	3.36 (3.15-3.57)	3.27 (2.94-3.53)	89.1 (84.01-93.96)	84.19 (77.87-89.98)	5.22 (4.89-5.51)	5.04 (4.64-5.42)
Hungary	4.67 (4.17-5.06)	3.8 (3.28-4.3)	131.62 (118.74-142.65)	101.8 (87.9-115.35)	6.62 (5.98-7.2)	5.39 (4.64-6.15)
Independent State of Papua New Guinea	0.61 (0.34-0.94)	0.84 (0.45-1.22)	18.44 (9.95-29.1)	25.5 (13.18-38.03)	1.13 (0.62-1.78)	1.67 (0.85-2.49)
Independent State of Samoa	1.41 (1.08-1.8)	1.81 (1.21-2.46)	39.14 (29.74-51.34)	51.3 (34.08-70.34)	2.49 (1.9-3.23)	3.54 (2.36-4.88)
Ireland	5.71 (5.29-6.08)	3.53 (3.09-3.93)	152.73 (141.54-162.33)	86.95 (78.28-96.18)	8.61 (8.01-9.19)	5.33 (4.74-5.88)
Islamic Republic of Afghanistan	0.95 (0.35-2.21)	1.57 (0.69-3.17)	29.32 (11.73-70.77)	46.59 (22.47-100.96)	1.36 (0.53-3.2)	2.28 (1.08-4.9)
Islamic Republic of Iran	0.68 (0.5-0.92)	1.13 (0.78-1.32)	20.45 (15.26-27.93)	32.98 (22.36-38.24)	1.08 (0.79-1.47)	1.9 (1.23-2.21)
Islamic Republic of Mauritania	0.78 (0.59-1.08)	1.14 (0.63-1.66)	22.29 (16.88-30.93)	33.46 (18.44-48.58)	1.05 (0.79-1.45)	1.7 (0.94-2.45)
Islamic Republic of Pakistan	2.04 (1.39-2.55)	3.43 (2.09-4.98)	61.5 (41.72-76.25)	101.62 (61.96-149.9)	2.93 (1.99-3.67)	5.17 (3.13-7.6)
Jamaica	2.39 (2.13-2.65)	2.8 (2.16-3.55)	68.8 (60.35-77.31)	81.5 (61.98-104.68)	3.61 (3.16-4.06)	4.44 (3.38-5.79)
Japan	2.21 (2.09-2.29)	1.82 (1.63-1.94)	67.2 (64.82-69.12)	54.02 (50.59-56.29)	3.64 (3.47-3.77)	3.49 (3.21-3.69)

Kingdom of Bahrain	3.17 (2.38-4.18)	3.25 (2.21-4.23)	81.64 (61.41-106.35)	78.62 (52.57-101.79)	4.28 (3.23-5.59)	4.52 (3.07-5.84)
Kingdom of Belgium	5.68 (5.21-6.05)	2.97 (2.6-3.31)	143.13 (132.83-151.83)	70.52 (63.6-77.71)	8.3 (7.67-8.83)	4.38 (3.94-4.83)
Kingdom of Bhutan	1.28 (0.77-2.01)	1.88 (1.26-3.36)	36.79 (22.77-58.9)	54.25 (35.31-96.88)	1.74 (1.06-2.73)	2.82 (1.82-5.03)
Kingdom of Cambodia	1.53 (0.96-2.66)	3.23 (2.05-4.74)	48.48 (29.42-87.9)	98.99 (60.67-148.75)	2.89 (1.74-5.28)	6.82 (4.18-10.3)
Kingdom of Denmark	4.61 (4.05-5.13)	3.54 (3.14-3.89)	125.44 (111.26-138.69)	81.85 (74.09-89.33)	6.33 (5.6-6.99)	4.86 (4.39-5.35)
Kingdom of Eswatini	2.46 (1.41-3.99)	3.68 (2.14-5.84)	65.84 (38.65-104.57)	98.46 (54.48-158.95)	3.29 (1.93-5.27)	4.99 (2.79-8.08)
Kingdom of Lesotho	1.96 (1.3-2.91)	3.44 (2.09-6.08)	52.38 (34.68-76.38)	90.46 (54.97-163.18)	2.57 (1.72-3.77)	4.48 (2.73-8.21)
Kingdom of Morocco	1 (0.64-1.3)	1.5 (0.96-2.06)	29.11 (19.08-38.1)	43.75 (27.06-61.05)	1.41 (0.93-1.83)	2.28 (1.44-3.16)
Kingdom of Norway	5.1 (4.81-5.33)	3.38 (3.02-3.63)	135.08 (129.12-140.71)	77.98 (71.96-82.92)	7.81 (7.38-8.18)	5 (4.57-5.36)
Kingdom of Saudi Arabia	0.92 (0.61-1.46)	1.2 (0.84-1.9)	25.44 (16.82-40.3)	34.53 (23.97-53.31)	1.28 (0.86-2.02)	1.92 (1.35-2.99)
Kingdom of Spain	2.98 (2.73-3.2)	2.39 (2.06-2.72)	83.73 (77.54-89.49)	62.14 (55.11-69.06)	5.76 (5.28-6.16)	4.32 (3.84-4.81)
Kingdom of Sweden	5.18 (4.86-5.44)	2.89 (2.48-3.31)	138.25 (130.79-145.71)	67.39 (58.18-76.88)	8.01 (7.56-8.41)	4.03 (3.48-4.62)
Kingdom of Thailand	1.73 (1.21-2.2)	2.21 (1.37-3)	52.25 (36.95-65.19)	69.88 (41.57-95.85)	3.52 (2.45-4.35)	5.68 (3.27-7.8)
Kingdom of the Netherlands	5.24 (4.8-5.56)	3.25 (2.87-3.6)	134.76 (125.51-142.09)	76.4 (68.97-83.11)	7.34 (6.8-7.77)	4.43 (4-4.83)
Kingdom of Tonga	0.77 (0.33-1.04)	1.15 (0.59-1.66)	21.97 (10.04-29.23)	32.04 (16.33-46.66)	1.43 (0.66-1.91)	2.29 (1.16-3.33)
Kyrgyz Republic	1.93 (1.57-2.36)	3.15 (2.46-3.91)	53.8 (44.75-65.07)	95.72 (75.62-118.01)	2.55 (2.12-3.08)	4.71 (3.68-5.84)
Lao People's Democratic Republic	1.44 (0.83-2.97)	2.45 (1.58-3.87)	45.25 (25.06-102.02)	77.11 (47.87-123.16)	2.62 (1.46-5.96)	5.25 (3.24-8.61)
Lebanese Republic	2.03 (1.48-3.04)	2.47 (1.64-3.22)	55.92 (40.51-86.06)	65.98 (44.48-86.37)	2.94 (2.12-4.55)	3.79 (2.52-4.94)
Malaysia	1.45 (1.16-2.1)	1.87 (1.48-2.66)	42.84 (34.31-63.91)	54.81 (43.72-78.1)	2.73 (2.14-4.27)	3.96 (3.1-5.83)
Mongolia	1.88 (1.3-2.65)	2.5 (1.75-3.29)	60.1 (41.8-84.49)	74.37 (52.73-97.64)	2.69 (1.85-3.77)	3.58 (2.53-4.71)
Montenegro	2.81 (2.14-3.6)	3.03 (2.19-4.02)	83.72 (62.43-108.27)	79.5 (56.15-106.28)	4.32 (3.23-5.59)	4.29 (3.06-5.73)
New Zealand	4.64 (4.3-4.97)	2.35 (2.08-2.63)	123.44 (115.39-131.89)	58.97 (53.01-64.98)	7.22 (6.72-7.74)	3.69 (3.29-4.09)
North Macedonia	3.08 (2.56-3.86)	3.72 (2.77-4.86)	92.27 (76.21-114.9)	98.57 (71.79-131.11)	4.47 (3.68-5.58)	5.15 (3.71-6.78)
Northern Mariana Islands	0.88 (0.6-1.57)	1.87 (1.45-2.22)	23.91 (16.36-43.47)	52.14 (40.32-61.54)	1.69 (1.15-3.12)	3.89 (3.01-4.64)
Palestine	1.36 (0.87-2.02)	1.84 (1.33-2.31)	38.93 (24.94-58.03)	49.63 (35.53-62.01)	1.98 (1.28-2.95)	2.75 (1.98-3.41)
People's Democratic Republic of Algeria	0.72 (0.5-0.96)	0.85 (0.6-1.14)	19.74 (14.08-25.38)	23.34 (16.5-31.71)	1.02 (0.72-1.31)	1.3 (0.93-1.75)

People's Republic of Bangladesh	1.09 (0.65-1.62)	1.6 (1.03-2.78)	32.97 (20.8-49)	48.74 (29.79-85.57)	1.55 (0.97-2.28)	2.56 (1.57-4.48)
People's Republic of China	1.34 (1.04-1.7)	1.18 (0.87-1.55)	40.95 (30.31-53.22)	35.69 (26-47.31)	2.03 (1.5-2.63)	2.03 (1.49-2.69)
Plurinational State of Bolivia	1.35 (0.96-2.41)	2.42 (1.55-3.57)	38.8 (27.18-70.91)	69.05 (44.22-96.56)	1.87 (1.32-3.43)	3.6 (2.31-5.11)
Portuguese Republic	2.72 (2.54-2.9)	2 (1.77-2.21)	73.62 (68.89-78.37)	51.33 (46.32-55.93)	3.73 (3.49-3.96)	2.65 (2.39-2.88)
Principality of Andorra	1.29 (0.83-1.99)	0.91 (0.62-1.25)	34.27 (21.76-52.29)	23.81 (16.1-32.53)	2 (1.28-3.01)	1.47 (0.98-2)
Principality of Monaco	3.73 (2.38-5.95)	3.1 (2.02-5.17)	102 (64.82-161.95)	80.09 (51.71-134.23)	6 (3.83-9.3)	5.02 (3.26-8.32)
Puerto Rico	1.37 (1.26-1.49)	2.18 (1.79-2.58)	36.47 (33.61-39.58)	62.06 (50.62-74.06)	2 (1.84-2.17)	3.65 (3.01-4.31)
Republic of Albania	1.11 (0.88-1.43)	1.2 (0.84-1.67)	31.2 (24.69-40.16)	33.16 (23.1-45.79)	1.54 (1.22-1.98)	1.75 (1.22-2.41)
Republic of Angola	0.91 (0.42-1.39)	1.66 (0.83-2.5)	26.31 (12.95-40.84)	47.77 (24.09-72.09)	1.22 (0.6-1.89)	2.35 (1.2-3.53)
Republic of Armenia	2.23 (1.85-2.78)	2.89 (2.38-3.44)	65.1 (53.57-83.12)	78.77 (64.54-93.75)	3.16 (2.59-4.02)	3.98 (3.3-4.77)
Republic of Austria	5.95 (5.54-6.3)	2.86 (2.5-3.11)	149.84 (140.91-158.32)	68.27 (61.49-74)	8.87 (8.35-9.37)	4.38 (3.97-4.75)
Republic of Azerbaijan	1.28 (0.85-1.81)	1.55 (1-2.29)	39.33 (26.19-56.37)	46.68 (29.95-69.28)	1.85 (1.21-2.66)	2.28 (1.46-3.44)
Republic of Belarus	3.02 (2.71-3.37)	3.19 (2.53-3.94)	88.19 (79.34-98.4)	91.74 (71.35-115.32)	4.05 (3.64-4.51)	4.5 (3.52-5.56)
Republic of Benin	0.69 (0.49-0.92)	1.03 (0.68-1.4)	20.49 (14.36-27.35)	29.19 (19.28-39.52)	0.95 (0.67-1.27)	1.44 (0.96-1.94)
Republic of Botswana	1.82 (1.15-2.59)	2.67 (1.43-3.77)	50.13 (31.08-71.27)	71.48 (35.97-104.76)	2.48 (1.57-3.5)	3.69 (1.91-5.29)
Republic of Bulgaria	3.07 (2.8-3.38)	4.42 (3.69-5.24)	95.62 (86.93-106.02)	127.59 (104.67-153.29)	4.82 (4.39-5.33)	6.66 (5.48-8.05)
Republic of Burundi	2.29 (1.21-3.57)	2.13 (1.31-2.99)	66.98 (34.51-105.7)	61.73 (36.7-86.1)	3.11 (1.63-4.88)	3.02 (1.84-4.17)
Republic of Cabo Verde	0.31 (0.21-0.47)	1.13 (0.42-1.69)	9.82 (6.69-14.45)	31.49 (12.17-45.82)	0.49 (0.33-0.72)	1.68 (0.67-2.43)
Republic of Cameroon	0.84 (0.65-1.11)	1.24 (0.76-1.73)	24.8 (19.27-32.2)	35.69 (21.45-50.43)	1.18 (0.92-1.53)	1.78 (1.07-2.52)
Republic of Chad	0.57 (0.3-0.82)	0.78 (0.45-1.12)	16.67 (8.52-23.8)	22.89 (13.18-32.93)	0.77 (0.4-1.11)	1.09 (0.64-1.56)
Republic of Chile	2.25 (2.09-2.44)	2.24 (2.03-2.44)	62.01 (57.21-67.22)	62.6 (57.23-68.11)	3.19 (2.95-3.46)	3.62 (3.3-3.93)
Republic of Colombia	2.34 (2.2-2.46)	2.66 (2.23-3.11)	69.33 (65.36-73.08)	78.97 (65.23-93.7)	3.53 (3.32-3.74)	4.43 (3.68-5.29)
Republic of Costa Rica	1.23 (1.14-1.35)	2.02 (1.76-2.29)	35.05 (32.48-38.19)	60.35 (52.89-67.82)	1.87 (1.74-2.04)	3.47 (3.04-3.92)
Republic of Croatia	4.64 (4.12-5.04)	3.82 (3.24-4.41)	125.11 (112.02-135.43)	98.9 (84.75-113.2)	7.75 (6.97-8.36)	6.56 (5.6-7.52)
Republic of Cuba	1.33 (1.23-1.45)	2.04 (1.72-2.35)	39.41 (36.21-42.59)	59.21 (49.33-68.47)	2.08 (1.92-2.25)	3.36 (2.83-3.87)
Republic of Cyprus	3.85 (2.91-5.57)	3.02 (2.18-3.92)	94.36 (72.91-131.63)	72.03 (52.63-94.03)	5.31 (4.11-7.47)	4.39 (3.2-5.73)

Republic of C 么 te d'Ivoire	1.3 (0.94-1.71)	1.81 (1.2-2.58)	36.43 (26.05-47.5)	50.75 (32.85-73.02)	1.75 (1.28-2.29)	2.56 (1.67-3.6)
Republic of Djibouti	2.07 (1.14-3.01)	2.53 (1.36-3.8)	58.49 (32.01-85.03)	71.44 (37-109.4)	2.79 (1.53-4.04)	3.57 (1.9-5.42)
Republic of Ecuador	0.32 (0.29-0.35)	2.17 (1.73-2.69)	9.22 (8.4-10.07)	62.65 (48.95-78.76)	0.47 (0.43-0.51)	3.44 (2.69-4.35)
Republic of El Salvador	1.22 (1.05-1.67)	2.36 (1.66-3.02)	37.86 (32.8-49.38)	72.29 (48.53-93.09)	1.88 (1.63-2.48)	3.96 (2.71-5.09)
Republic of Equatorial Guinea	1.1 (0.67-1.87)	2.16 (1.22-3.4)	31.83 (19.46-55.71)	61.39 (33.38-100.59)	1.47 (0.9-2.5)	3.13 (1.73-5.05)
Republic of Estonia	4.78 (4.25-5.38)	3.52 (2.92-4.2)	133.09 (119.12-148.76)	90.67 (76.01-107.21)	6.67 (5.96-7.39)	4.9 (4.14-5.81)
Republic of Fiji	1.03 (0.78-1.37)	1.26 (0.88-1.68)	30.71 (23.04-41.45)	35.41 (24.63-48.26)	1.93 (1.46-2.52)	2.4 (1.67-3.23)
Republic of Finland	4.83 (4.51-5.12)	2.88 (2.52-3.19)	123.06 (115.58-130.71)	69.75 (62.22-76.07)	7.56 (7.06-8.03)	4.8 (4.27-5.27)
Republic of Ghana	0.77 (0.54-1.01)	1.48 (0.89-2.19)	23.17 (16.16-30.42)	42.46 (25.4-62.73)	1.12 (0.78-1.47)	2.15 (1.27-3.15)
Republic of Guatemala	0.56 (0.53-0.6)	1.21 (1.04-1.41)	17.48 (16.54-18.47)	37.36 (31.71-43.49)	0.87 (0.83-0.92)	1.95 (1.65-2.28)
Republic of Guinea	0.78 (0.59-1.02)	1.07 (0.63-1.51)	23.07 (17.36-30.24)	31.7 (17.79-44.89)	1.07 (0.81-1.39)	1.54 (0.87-2.18)
Republic of Guinea-Bissau	0.79 (0.58-1.16)	1.32 (0.9-1.79)	24.14 (17.27-36.45)	38.85 (26.75-53.44)	1.11 (0.8-1.64)	1.88 (1.31-2.58)
Republic of Guyana	2.7 (2.32-3.19)	3.99 (3.01-5.12)	82.01 (70.4-96.07)	122.32 (90.02-159.59)	4 (3.47-4.72)	6.23 (4.64-8.06)
Republic of Haiti	2.06 (1.21-4.44)	2.72 (1.78-4.77)	60.6 (36.69-140.46)	80.3 (51.34-145.63)	2.83 (1.72-6.53)	3.89 (2.49-7.15)
Republic of Honduras	1.93 (1.53-2.44)	3.25 (2.1-5.34)	62.49 (48.5-78.16)	101.64 (63.13-160.18)	3.05 (2.4-3.83)	5.21 (3.17-8.23)
Republic of Iceland	5.06 (4.6-5.51)	2.91 (2.48-3.26)	131.02 (120.43-143.14)	71.4 (62.6-79.52)	7.65 (6.96-8.33)	4.48 (3.92-5)
Republic of India	1.17 (0.85-1.53)	1.9 (1.61-2.25)	35.42 (25.67-46.87)	54.51 (46.09-64.85)	1.68 (1.21-2.22)	2.82 (2.36-3.35)
Republic of Indonesia	1.46 (1.04-2.66)	2.02 (1.36-3.44)	47.55 (33.73-88.12)	64 (42.23-106.76)	2.98 (2.1-5.57)	4.58 (3.02-7.5)
Republic of Iraq	1.17 (0.83-1.78)	1.69 (1.23-2.24)	36.06 (25.67-54.07)	49.46 (34.6-68.12)	1.79 (1.27-2.71)	2.71 (1.92-3.79)
Republic of Italy	3.43 (3.23-3.59)	2.88 (2.53-3.11)	93.9 (89.6-97.2)	72.92 (66.89-77.29)	5.58 (5.29-5.84)	4.61 (4.22-4.9)
Republic of Kazakhstan	2.68 (2.25-3.2)	2.89 (2.38-3.49)	82.22 (68.89-99.52)	86.48 (70.37-104.47)	3.92 (3.3-4.75)	4.31 (3.51-5.18)
Republic of Kenya	1.39 (0.57-1.94)	2.36 (1.53-3.21)	40.15 (16.3-56.64)	67.01 (42.1-93.07)	1.93 (0.79-2.7)	3.37 (2.11-4.66)
Republic of Kiribati	0.28 (0.18-0.37)	0.46 (0.26-0.66)	8.14 (5.26-10.93)	13.42 (7.22-19.28)	0.49 (0.31-0.65)	0.87 (0.47-1.26)
Republic of Korea	1.24 (1.07-1.81)	1.5 (0.95-1.78)	38.22 (33.96-50.9)	42.45 (28.93-49.61)	1.95 (1.73-2.62)	2.75 (1.84-3.24)
Republic of Latvia	3.9 (3.51-4.36)	4.96 (4.22-5.73)	113.82 (101.91-127.39)	137.71 (115.32-161.79)	4.97 (4.46-5.54)	6.3 (5.34-7.33)
Republic of Liberia	0.65 (0.5-0.88)	1.12 (0.7-1.58)	19.17 (14.33-26.4)	32.37 (20.38-46.49)	0.9 (0.67-1.24)	1.62 (1.03-2.3)

Republic of Lithuania	4.37 (3.94-4.76)	4.52 (3.78-5.2)	124.98 (113.2-135.78)	121.25 (102.06-141.01)	5.9 (5.34-6.45)	6.03 (5.08-6.96)
Republic of Madagascar	1.86 (1.25-2.46)	2.2 (1.38-3.28)	55.8 (36.9-75.54)	65.99 (40.95-98.35)	2.63 (1.74-3.54)	3.25 (2.05-4.74)
Republic of Malawi	1.29 (0.89-1.7)	2.02 (0.94-3.07)	37.5 (25.7-49.93)	57.39 (26.19-87.11)	1.77 (1.22-2.33)	2.85 (1.31-4.35)
Republic of Maldives	1.4 (0.82-3.45)	1.69 (1.29-2.13)	44.61 (25.51-119.62)	49.1 (35.56-64.05)	2.81 (1.61-7.44)	3.77 (2.72-4.93)
Republic of Mali	0.45 (0.33-0.57)	0.56 (0.35-0.76)	13.6 (9.84-16.91)	16.68 (10.07-22.72)	0.63 (0.46-0.78)	0.81 (0.49-1.1)
Republic of Malta	5.01 (4.52-5.41)	3.68 (3.22-4.16)	129.95 (118.59-140.43)	93.51 (82.7-105.03)	7.35 (6.68-7.92)	5.68 (5.02-6.42)
Republic of Mauritius	1.94 (1.8-2.09)	2.98 (2.69-3.19)	60.01 (55.87-65.19)	94.06 (84.48-100.7)	4.15 (3.85-4.47)	7.28 (6.6-7.83)
Republic of Moldova	3.37 (3.11-3.58)	2.28 (1.99-2.58)	104.59 (96.96-111.27)	67.17 (57.9-75.88)	4.65 (4.29-4.95)	3.15 (2.72-3.58)
Republic of Mozambique	2.02 (1.21-2.78)	2.98 (1.84-4.3)	58.96 (33.91-82.03)	84.78 (51.07-124.96)	2.78 (1.64-3.9)	4.17 (2.54-5.97)
Republic of Namibia	1.51 (1.13-1.93)	2.15 (1.38-3.07)	41.65 (30.62-53.43)	58.61 (36.04-87.43)	2.03 (1.5-2.6)	2.99 (1.87-4.43)
Republic of Nauru	0.88 (0.55-1.5)	1.66 (0.84-2.71)	27.43 (16.6-47.71)	48.77 (23.62-83.55)	1.72 (1.04-3.02)	3.31 (1.59-5.81)
Republic of Nicaragua	0.78 (0.61-1.13)	1.35 (1-1.69)	24.21 (19.06-33.76)	41.03 (29.8-52.18)	1.24 (0.98-1.71)	2.21 (1.62-2.78)
Republic of Niue	1.21 (0.83-1.57)	1.77 (0.98-2.49)	32.9 (22.23-43.57)	50.71 (27.93-71.85)	2.19 (1.49-2.9)	3.92 (2.17-5.48)
Republic of Palau	0.31 (0.21-0.42)	0.35 (0.26-0.44)	8.38 (5.76-11.84)	9.13 (6.63-11.83)	0.57 (0.39-0.79)	0.68 (0.49-0.87)
Republic of Panama	0.95 (0.87-1.02)	1.91 (1.54-2.29)	28.82 (26.62-31.05)	58.42 (47.01-69.93)	1.55 (1.43-1.68)	3.31 (2.7-3.94)
Republic of Paraguay	1.11 (0.84-1.63)	1.77 (1.22-2.4)	32.06 (24.47-46.4)	49.49 (33.75-68)	1.67 (1.29-2.37)	2.69 (1.85-3.68)
Republic of Peru	1.68 (1.3-2.1)	2.18 (1.53-2.89)	49.68 (38.92-63.79)	64.59 (44.68-87.24)	2.5 (1.94-3.22)	3.61 (2.52-4.81)
Republic of Poland	5.06 (4.87-5.22)	4.98 (4.43-5.51)	151.05 (145.81-155.09)	128.35 (114.02-142.21)	7.18 (6.9-7.44)	6.61 (5.88-7.33)
Republic of Rwanda	2.77 (1.48-4.47)	3.69 (2.36-5.22)	81.58 (43.39-137.35)	103.97 (64.99-150.97)	3.78 (2.05-6.14)	5.21 (3.29-7.44)
Republic of San Marino	2.53 (1.95-3.37)	1.3 (0.78-1.89)	61.47 (46.67-82.32)	34.02 (20.14-50.53)	3.7 (2.83-4.93)	2.11 (1.25-3.15)
Republic of Senegal	0.68 (0.48-0.89)	1.21 (0.7-1.8)	20.21 (14.34-26.62)	35.17 (20.37-51.62)	0.96 (0.68-1.25)	1.75 (1.03-2.56)
Republic of Serbia	3.54 (2.89-4.39)	3.89 (2.77-4.82)	98.6 (79.72-122.78)	106.17 (75.8-132.67)	4.73 (3.79-5.93)	5.28 (3.73-6.57)
Republic of Seychelles	2.79 (2.27-3.85)	3.68 (2.88-4.45)	88.95 (73.04-117.72)	109.72 (83.41-132.75)	6.05 (4.93-7.99)	8.45 (6.36-10.23)
Republic of Sierra Leone	0.57 (0.36-0.76)	0.94 (0.59-1.33)	16.77 (10.25-22.67)	27.46 (17.07-38.68)	0.79 (0.49-1.06)	1.36 (0.85-1.86)
Republic of Singapore	2.7 (2.52-2.87)	1.7 (1.53-1.87)	80.34 (74.98-85.65)	48.9 (44.7-52.97)	4.13 (3.87-4.39)	3.11 (2.83-3.37)
Republic of Slovenia	4.24 (3.82-4.65)	2.93 (2.46-3.43)	115.99 (104.02-127.55)	73.07 (60.88-86.83)	5.62 (5.05-6.18)	3.81 (3.19-4.52)

Republic of South Africa	1.93 (1.42-2.46)	3.02 (2.3-3.48)	53.83 (42.42-68)	79.54 (62.36-92.25)	2.76 (2.2-3.47)	4.16 (3.25-4.82)
Republic of South Sudan	1.56 (1.04-2.3)	2.12 (1.43-3.06)	45.31 (29.58-68.32)	62.37 (41.26-91.37)	2.16 (1.42-3.24)	3.05 (2.02-4.46)
Republic of Sudan	0.64 (0.15-1.39)	0.88 (0.54-1.49)	19.21 (4.85-42.1)	26.99 (16.33-44.81)	0.93 (0.23-2.02)	1.42 (0.86-2.31)
Republic of Suriname	2.76 (2.27-3.41)	3.04 (2.35-3.96)	81.6 (67.83-100.27)	89.32 (70.16-116.87)	4.09 (3.41-4.97)	4.73 (3.69-6.1)
Republic of Tajikistan	0.91 (0.62-1.24)	0.85 (0.61-1.13)	26.7 (19.12-37.97)	27.06 (19.22-35.86)	1.26 (0.9-1.79)	1.28 (0.9-1.69)
Republic of the Congo	1.51 (1.08-2.33)	2.08 (1.41-2.83)	43.02 (30.69-69.82)	60.13 (41.22-84.16)	2.02 (1.43-3.19)	3 (2.04-4.19)
Republic of the Gambia	0.6 (0.44-0.78)	1.04 (0.67-1.45)	16.62 (11.99-21.77)	29.58 (18.64-42.21)	0.81 (0.58-1.04)	1.48 (0.95-2.11)
Republic of the Marshall Islands	0.94 (0.64-1.41)	1.53 (0.8-2.39)	25.99 (17.74-41.23)	45.3 (23.13-73.32)	1.61 (1.11-2.52)	3 (1.53-4.93)
Republic of the Niger	0.49 (0.33-0.73)	0.74 (0.47-1.08)	14.44 (9.8-21.23)	21.33 (13.51-31.46)	0.67 (0.45-0.99)	1.03 (0.66-1.49)
Republic of the Philippines	1.97 (1.38-2.4)	2.8 (2.17-3.52)	62.56 (45.38-73.89)	89.33 (67.94-113.85)	4.21 (3.11-4.97)	6.44 (4.76-8.19)
Republic of the Union of Myanmar	1.81 (1.23-2.98)	2.46 (1.77-3.48)	58.43 (38.45-101.64)	77.21 (54.23-110.61)	3.54 (2.29-6.21)	5.4 (3.77-7.9)
Republic of Trinidad and Tobago	3.66 (3.24-4.38)	3.44 (2.65-4.36)	106.79 (96.35-126.68)	103.92 (78.77-133.21)	5.46 (4.92-6.45)	5.63 (4.27-7.15)
Republic of Tunisia	1.25 (0.9-1.64)	1.69 (1.12-2.43)	35.28 (25.85-45.87)	47.22 (31.57-67.21)	1.83 (1.34-2.36)	2.65 (1.78-3.79)
Republic of Turkey	2.44 (1.74-3.62)	2.46 (1.74-3.13)	70.64 (48.83-108.33)	67.32 (46.71-86.61)	3.57 (2.46-5.53)	3.83 (2.67-4.91)
Republic of Uganda	2.31 (1.63-3.19)	3.97 (2.54-5.56)	63.52 (44.39-87.36)	109.56 (67.27-155.03)	3.06 (2.16-4.18)	5.55 (3.52-7.84)
Republic of Uzbekistan	0.98 (0.76-1.27)	1.45 (1.12-1.82)	30.53 (23.54-38.2)	46.16 (35.59-58.42)	1.44 (1.12-1.79)	2.23 (1.71-2.81)
Republic of Vanuatu	0.45 (0.19-0.87)	0.83 (0.44-1.4)	13 (5.53-25.46)	24.47 (12.6-42.4)	0.81 (0.34-1.55)	1.61 (0.83-2.77)
Republic of Yemen	0.52 (0.06-1.14)	0.86 (0.39-1.4)	14.57 (2.02-32.15)	25 (11.83-40.89)	0.7 (0.09-1.51)	1.26 (0.58-2.06)
Republic of Zambia	2.12 (1.21-2.86)	3.05 (1.89-4.55)	64.43 (36.36-88)	87.98 (53.15-134.28)	3.03 (1.71-4.09)	4.41 (2.66-6.68)
Republic of Zimbabwe	2.25 (1.69-2.94)	4.19 (2.73-5.66)	59.5 (44.7-77.13)	118.63 (74.28-165.07)	2.99 (2.25-3.91)	5.83 (3.73-7.96)
Romania	2.99 (2.81-3.16)	3.73 (3.24-4.24)	96.82 (90.35-102.06)	103.74 (89.32-117.93)	4.65 (4.33-4.94)	5.34 (4.54-6.09)
Russian Federation	4.18 (4.03-4.28)	3.72 (3.31-4.1)	126.73 (122.79-129.7)	105.18 (93.42-116.91)	6.67 (6.46-6.82)	5.92 (5.27-6.54)
Saint Kitts and Nevis	3.62 (2.84-4.54)	3.52 (2.85-4.11)	102.2 (78.12-131.27)	87.85 (70.29-105.19)	5.17 (3.93-6.61)	4.82 (3.92-5.76)
Saint Lucia	3.47 (2.96-4.26)	3.44 (2.8-4.09)	99.36 (83.66-122.99)	98.13 (79.17-117.67)	5.11 (4.33-6.33)	5.39 (4.37-6.5)
Saint Vincent and the Grenadines	3.09 (2.71-3.78)	3 (2.61-3.46)	88.96 (78.61-107.31)	91.89 (79.9-106.41)	4.63 (4.13-5.58)	4.98 (4.29-5.78)
Slovak Republic	4.33 (3.43-5.02)	3.85 (2.79-4.89)	125.8 (98.16-147.09)	103.68 (75.34-129.87)	6.09 (4.76-7.12)	5.48 (4.01-6.9)

Socialist Republic of Viet Nam	0.95 (0.65-1.23)	1.44 (0.94-1.94)	26.89 (19.01-35.25)	39.98 (25.72-54.37)	1.72 (1.23-2.24)	2.9 (1.89-3.9)
Solomon Islands	0.52 (0.18-0.85)	1.1 (0.61-1.69)	15.45 (5.41-26.13)	33.47 (18.19-53.45)	0.93 (0.32-1.55)	2.18 (1.18-3.44)
State of Eritrea	2.28 (1.51-3.27)	3.41 (2.24-4.91)	65.03 (42.2-94.75)	94.67 (62.22-139.57)	3.02 (1.95-4.36)	4.61 (3.02-6.77)
State of Israel	4.33 (3.99-4.63)	2.71 (2.35-2.98)	116.12 (107.92-123.74)	65.74 (59.38-72.27)	6.41 (5.93-6.82)	3.93 (3.51-4.31)
State of Kuwait	1.49 (1.33-1.63)	1.08 (0.91-1.29)	39.08 (35.49-42.93)	31.05 (26.1-36.99)	2.14 (1.94-2.34)	1.85 (1.56-2.21)
State of Libya	1.71 (1.1-2.38)	2.74 (1.87-3.71)	48.51 (31.27-66.77)	77.59 (53.09-105.84)	2.48 (1.6-3.41)	4.17 (2.87-5.6)
State of Qatar	2.02 (1.41-2.75)	2.3 (1.58-3.03)	49.28 (35.02-66.2)	53.18 (35.25-70.75)	2.69 (1.93-3.64)	3.13 (2.07-4.13)
Sultanate of Oman	0.64 (0.27-1.02)	0.74 (0.53-0.98)	17.08 (7.38-27.52)	19.56 (13.53-26.06)	0.86 (0.38-1.37)	1.08 (0.76-1.42)
Swiss Confederation	2.88 (2.51-3.2)	2.36 (2.01-2.71)	75.55 (67.26-82.98)	53.61 (46.94-60.63)	4.76 (4.22-5.21)	3.48 (3.03-3.93)
Syrian Arab Republic	0.72 (0.49-0.99)	0.96 (0.65-1.34)	21.99 (15.36-30.81)	28.79 (19.35-41.1)	1.12 (0.77-1.57)	1.63 (1.09-2.32)
Taiwan (Province of China)	1.18 (1.09-1.27)	1.68 (1.47-1.87)	37.01 (33.87-39.78)	53.18 (46.39-59.22)	2.88 (2.63-3.11)	4.87 (4.24-5.38)
Togolese Republic	0.75 (0.52-0.96)	1.26 (0.74-1.84)	22.36 (15.46-29.32)	35.87 (20.9-52.97)	1.06 (0.73-1.38)	1.79 (1.05-2.6)
Tokelau	1.07 (0.71-1.46)	1.51 (0.84-2.21)	31.3 (20.43-44.3)	45.8 (25.05-65.74)	1.99 (1.29-2.79)	3.49 (1.9-4.92)
Turkmenistan	1.2 (1.06-1.34)	2.11 (1.62-2.81)	38.55 (34.28-42.75)	68.39 (52.21-92.06)	1.77 (1.57-1.97)	3.32 (2.51-4.47)
Tuvalu	0.94 (0.55-1.51)	1.44 (0.84-2.08)	26.95 (16-45.57)	40.78 (23.06-61.72)	1.64 (0.98-2.76)	2.77 (1.59-4.18)
Ukraine	3 (2.56-3.47)	3.14 (2-4.54)	93.54 (79.6-108.8)	97.43 (60.45-143.16)	4.73 (4.02-5.5)	5.02 (3.16-7.3)
Union of the Comoros	2.17 (1.34-3.07)	3.25 (1.84-4.97)	64.48 (38.72-92.2)	94.98 (51.88-146.89)	3.06 (1.82-4.36)	4.75 (2.63-7.33)
United Arab Emirates	3.49 (2.28-6.01)	4.41 (3.44-5.63)	89.36 (58.4-159.71)	97.66 (75.81-126.56)	4.63 (3.02-8.2)	5.64 (4.45-7.23)
United Kingdom of Great Britain and Northern Ireland	6.02 (5.76-6.16)	3.62 (3.3-3.79)	160.45 (156.04-163.64)	86.9 (82-89.97)	9.16 (8.86-9.34)	5.79 (5.41-6)
United Mexican States	2.12 (2.05-2.18)	2.89 (2.44-3.37)	60.91 (59.35-62.46)	88.57 (74.37-104.35)	3.16 (3.07-3.24)	4.8 (4.04-5.63)
United Republic of Tanzania	2.24 (1.39-2.89)	2.95 (2.03-3.92)	65.36 (39.82-86.16)	85.32 (54.89-114.78)	3.13 (1.93-4.1)	4.27 (2.82-5.75)
United States of America	4.58 (4.29-4.75)	2.96 (2.68-3.12)	119.95 (115.09-123.19)	73.71 (69.52-76.92)	7.34 (6.97-7.55)	4.74 (4.41-4.95)
United States Virgin Islands	3.21 (2.51-4.38)	2.92 (2.06-4.03)	89.51 (69.58-127.54)	86.04 (59.71-119.17)	4.79 (3.73-6.84)	4.92 (3.35-6.92)
**Uterine cancer**						
Global	1.44 (1.28-1.55)	1.14 (1.01-1.26)	36.66 (31.84-39.83)	29.4 (26.24-32.64)	4.72 (4.32-4.97)	5.41 (4.9-5.87)

Low SDI	0.91 (0.7-1.1)	0.94 (0.75-1.21)	23.53 (18.04-28.07)	23.87 (19.07-30.72)	1.45 (1.12-1.73)	1.83 (1.47-2.34)
Low-middle SDI	0.78 (0.66-0.91)	0.86 (0.74-1.09)	20.63 (17.13-23.81)	22.62 (19.36-28.13)	1.45 (1.22-1.67)	2.13 (1.84-2.62)
Middle SDI	1.14 (0.89-1.32)	0.85 (0.71-0.99)	31.58 (23.37-37.06)	23.29 (19.1-27.23)	2.48 (1.9-2.88)	3.14 (2.57-3.71)
High-middle SDI	1.82 (1.67-1.95)	1.32 (1.18-1.46)	48.94 (44.37-52.87)	34.92 (31.33-39.02)	6.44 (6.03-6.81)	7.51 (6.81-8.3)
High SDI	1.64 (1.51-1.71)	1.46 (1.3-1.54)	39.3 (37.18-41.2)	37.53 (34.53-39.92)	7.59 (7.18-7.84)	10.13 (9.43-10.55)
**Four World Regions**						
Africa	0.85 (0.68-1.06)	0.99 (0.76-1.2)	21.53 (17.09-26.41)	24.29 (18.72-29.76)	1.48 (1.19-1.83)	2.2 (1.71-2.63)
America	1.67 (1.55-1.74)	1.67 (1.52-1.77)	41.48 (39.37-43.36)	43.85 (40.84-46.96)	7.69 (7.26-7.93)	10.07 (9.41-10.53)
Asia	1 (0.78-1.17)	0.74 (0.62-0.91)	27.7 (20.87-32.64)	20.49 (16.95-24.9)	2.22 (1.71-2.58)	2.88 (2.38-3.51)
Europe	2.11 (1.99-2.19)	1.89 (1.72-2.02)	53.71 (51.38-55.86)	48.29 (44.6-52)	8.77 (8.45-9.03)	11.58 (10.77-12.24)
**Countries**						
American Samoa	1.93 (1.17-2.62)	2.59 (1.19-4.99)	51.83 (31.16-69.91)	72.87 (33.95-140.91)	5 (3.03-6.81)	7.69 (3.62-14.4)
Antigua and Barbuda	1.73 (1.57-1.9)	2.87 (2.69-3.08)	44.47 (39.76-48.96)	70.67 (66.16-75.72)	4.7 (4.17-5.2)	8.95 (8.31-9.72)
Arab Republic of Egypt	0.59 (0.46-0.77)	0.59 (0.44-0.79)	15.73 (11.66-20.15)	15.88 (11.73-21.4)	1.52 (1.13-1.96)	2.58 (1.88-3.56)
Argentine Republic	1.81 (1.65-1.95)	1.21 (1.08-1.34)	45.68 (41.68-48.98)	29.46 (26.5-32.53)	4.33 (3.94-4.67)	3.91 (3.47-4.4)
Australia	1.01 (0.9-1.12)	1.03 (0.85-1.17)	23.83 (21.55-26.21)	25.43 (21.88-28.98)	4.95 (4.47-5.43)	7.41 (6.4-8.35)
Barbados	2.98 (2.71-3.27)	3.8 (2.97-4.7)	75.63 (69.12-82.48)	93.77 (71.76-118.51)	8.18 (7.49-8.93)	12.02 (9.26-15.15)
Belize	1.8 (1.6-2.27)	2.37 (2.1-2.7)	47.48 (41.93-62.26)	65.74 (57.92-75.43)	4.25 (3.74-5.51)	6.71 (5.88-7.76)
Bermuda	2.57 (2.28-2.86)	1.96 (1.62-2.51)	61.47 (54.49-68.87)	47.93 (39.64-61.1)	7.93 (6.98-8.95)	9.72 (7.99-12.44)
Bolivarian Republic of Venezuela	1.92 (1.79-2.04)	1.53 (1.12-2.01)	49.06 (46.13-52.04)	39.37 (28.64-52.15)	4.25 (3.94-4.53)	4.58 (3.35-6.1)
Bosnia and Herzegovina	1.46 (1.13-1.94)	1.49 (1.02-2.03)	37.07 (28.62-49.36)	37.47 (24.84-50.73)	4.02 (3.07-5.33)	6.62 (4.38-8.95)
Brunei Darussalam	1.53 (1.08-2.28)	1.51 (1.1-2.01)	41.05 (28.02-61.59)	39.74 (29.07-53.03)	2.46 (1.68-3.67)	3.45 (2.52-4.6)
Burkina Faso	1.13 (0.79-1.6)	1.26 (0.84-1.79)	27.07 (18.81-38.17)	29.04 (19.27-41.92)	1.72 (1.19-2.44)	2.11 (1.38-3.05)
Canada	1.66 (1.5-1.82)	1.55 (1.34-1.74)	39.78 (35.61-43.85)	39.06 (34.3-44.15)	8.04 (7.2-8.89)	10.58 (9.39-12.05)
Central African Republic	1.28 (0.94-1.77)	1.32 (0.85-1.98)	33.49 (23.91-46.54)	33.13 (21.28-50.64)	1.85 (1.32-2.58)	1.95 (1.25-2.97)
Commonwealth of Dominica	2.25 (1.78-3.45)	2.49 (1.81-3.28)	54.67 (43.63-80.5)	58.88 (44.33-79.04)	4.94 (3.95-7.24)	5.61 (4.18-7.45)

Commonwealth of the Bahamas	2.32 (2.11-2.53)	3.26 (2.62-4)	64.54 (58.61-70.54)	86.97 (69.32-108.22)	6.18 (5.59-6.73)	9.43 (7.51-11.59)
Cook Islands	0.88 (0.62-1.23)	0.75 (0.54-1.03)	23.81 (16.63-33.66)	20.69 (14.85-28.42)	2.66 (1.84-3.68)	3.35 (2.38-4.72)
Czech Republic	3.31 (2.83-3.83)	2.09 (1.68-2.6)	80.56 (69.04-93.63)	51.03 (40.57-63.48)	10.99 (9.27-12.73)	11.38 (9.03-14.27)
Democratic People's Republic of Korea	1.09 (0.71-1.48)	1 (0.64-1.39)	31.87 (20.15-44.24)	30.51 (19.01-43.82)	3.11 (1.97-4.31)	3.8 (2.31-5.51)
Democratic Republic of Sao Tome and Principe	1.66 (1.19-2.02)	2.03 (1.34-2.71)	36.9 (28.16-46.4)	46.14 (31.07-62.03)	2.6 (1.96-3.25)	4.27 (2.85-5.73)
Democratic Republic of the Congo	1.02 (0.74-1.5)	1.19 (0.74-1.88)	27.39 (19.55-39.59)	29.78 (18.39-46.3)	1.65 (1.17-2.4)	2.15 (1.34-3.4)
Democratic Republic of Timor-Leste	1.09 (0.69-1.56)	1.13 (0.74-1.63)	30.77 (18.71-45.03)	32 (19.86-46.66)	2.09 (1.26-3.03)	2.69 (1.67-3.97)
Democratic Socialist Republic of Sri Lanka	0.81 (0.61-1.04)	0.74 (0.47-1.09)	21.85 (16.32-28.55)	19.51 (11.9-29.06)	2.12 (1.58-2.78)	2.94 (1.72-4.44)
Dominican Republic	1.88 (1.48-2.48)	2.2 (1.58-2.89)	50.18 (39.33-65.27)	59.71 (41.88-79.3)	3.95 (3.07-5.19)	5.72 (3.97-7.61)
Eastern Republic of Uruguay	1.65 (1.5-1.78)	1.28 (1.15-1.41)	40.32 (36.42-43.89)	31.55 (28.56-35.04)	4.31 (3.87-4.71)	4.71 (4.24-5.18)
Federal Democratic Republic of Ethiopia	1.05 (0.63-1.54)	0.76 (0.49-1.24)	27.19 (16.29-39.35)	18.86 (12.13-31.59)	1.49 (0.89-2.17)	1.45 (0.93-2.39)
Federal Democratic Republic of Nepal	0.51 (0.31-0.93)	0.5 (0.3-1.03)	13.24 (7.85-24.28)	12.6 (7.49-26.23)	0.82 (0.47-1.5)	1.12 (0.67-2.34)
Federal Republic of Germany	1.93 (1.74-2.12)	1.16 (1.01-1.29)	44.39 (40.34-48.71)	28.13 (24.78-31.52)	9.77 (8.83-10.63)	7.95 (7.06-8.82)
Federal Republic of Nigeria	0.51 (0.34-0.81)	0.63 (0.44-0.92)	11.53 (7.67-18.4)	15.14 (10.37-22.51)	0.74 (0.5-1.17)	1.21 (0.82-1.79)
Federal Republic of Somalia	1.39 (0.77-2.15)	1.49 (0.87-2.2)	36.44 (19.93-56.73)	36.94 (21.42-55.84)	2.06 (1.14-3.23)	2.25 (1.32-3.44)
Federated States of Micronesia	2.26 (1.35-3.31)	2.66 (1.45-4.16)	62.35 (35.61-93.28)	73.54 (39.51-117.06)	4.45 (2.52-6.66)	6.27 (3.37-9.85)
Federative Republic of Brazil	1.54 (1.45-1.63)	1.33 (1.21-1.42)	36.92 (34.83-38.94)	33.17 (30.64-35.23)	3.11 (2.95-3.27)	3.91 (3.61-4.15)
French Republic	1.87 (1.68-2.07)	1.45 (1.24-1.62)	42.51 (38.78-46.59)	34.4 (30.37-38.69)	7.68 (6.93-8.47)	9.52 (8.49-10.74)
Gabonese Republic	1.26 (0.83-1.84)	1.34 (0.82-1.94)	32.21 (21.08-46.42)	33.3 (20.2-48.14)	2.14 (1.41-3.09)	2.96 (1.81-4.29)
Georgia	3.89 (3.51-4.29)	3.67 (3.11-4.32)	108.65 (98.83-119.44)	96.8 (82.49-113.62)	12.33 (11.11-13.58)	13.07 (11.02-15.32)
Grand Duchy of Luxembourg	2.88 (2.67-3.08)	1.78 (1.55-1.98)	66.27 (61.33-71.4)	41.03 (36.41-45.8)	11.79 (10.82-12.79)	11.19 (9.9-12.57)
Greenland	0.96 (0.71-1.31)	0.52 (0.38-0.72)	22.61 (16.82-30.23)	12.91 (9.34-17.61)	2.14 (1.59-2.89)	1.75 (1.27-2.41)
Grenada	2.48 (2.17-2.83)	2.94 (2.4-3.41)	66.5 (57.99-75.61)	71.97 (58.77-84.51)	5.69 (4.95-6.55)	7.52 (6.15-8.83)
Guam	1.54 (1.22-1.93)	1.14 (0.86-1.4)	39.7 (31.15-49.22)	36.11 (27.43-43.64)	5.02 (3.97-6.25)	5.12 (3.95-6.23)
Hashemite Kingdom of Jordan	0.89 (0.64-1.21)	0.68 (0.46-1.02)	23.35 (16.7-32.38)	17.84 (11.82-26.63)	2.98 (2.13-4.18)	3.98 (2.62-5.93)
Hellenic Republic	1.39 (1.29-1.47)	1.56 (1.4-1.67)	32.66 (30.59-34.87)	39.61 (36.34-42.95)	6.92 (6.42-7.39)	10.28 (9.34-11.18)

Hungary	3.02 (2.64-3.45)	2.14 (1.79-2.58)	73.51 (64.42-83.69)	54.95 (45.62-66)	8.91 (7.79-10.16)	11.17 (9.21-13.49)
Independent State of Papua New Guinea	1.38 (0.76-2.11)	1.51 (0.78-2.51)	39.11 (20.69-60.65)	43 (21.7-73.3)	2.89 (1.5-4.52)	3.29 (1.64-5.53)
Independent State of Samoa	1.96 (1.48-2.63)	2.27 (1.52-3.39)	48.65 (35.09-70.75)	58.53 (38.2-89.44)	4.17 (3.01-6.16)	5.87 (3.78-9.17)
Ireland	1.69 (1.53-1.83)	1.32 (1.12-1.5)	39.43 (35.68-43.19)	32.53 (28.07-37.04)	7.29 (6.59-8.03)	9.56 (8.19-10.89)
Islamic Republic of Afghanistan	0.94 (0.48-1.9)	1.36 (0.69-2.39)	27.6 (12.88-56.8)	39.02 (19.08-67.34)	1.95 (0.92-3.97)	3.7 (1.73-6.42)
Islamic Republic of Iran	0.33 (0.23-0.41)	0.39 (0.21-0.49)	9.04 (5.93-11.3)	10.9 (5.61-13.91)	1.19 (0.75-1.5)	2.52 (1.23-3.26)
Islamic Republic of Mauritania	1.37 (0.95-1.88)	1.39 (0.88-1.98)	33.4 (22.86-46.1)	33.27 (21.74-46.66)	2.16 (1.49-2.99)	3.07 (1.98-4.36)
Islamic Republic of Pakistan	1.39 (1.11-1.9)	2.03 (1.46-2.93)	36.1 (28.77-47.77)	51.62 (36.1-73.77)	2.33 (1.83-3.06)	4.05 (2.88-5.76)
Jamaica	1.7 (1.53-1.91)	3.25 (2.51-4.13)	43.95 (39.67-48.86)	84.63 (64.92-110.08)	4.64 (4.15-5.16)	9.86 (7.47-12.95)
Japan	1 (0.91-1.04)	1.04 (0.91-1.11)	23.8 (22.35-24.83)	29.79 (27.3-31.45)	2.33 (2.19-2.43)	5.26 (4.87-5.57)
Kingdom of Bahrain	1.15 (0.85-1.64)	1.11 (0.77-1.55)	27.81 (20.62-39.07)	26.04 (17.83-37.24)	3.66 (2.68-5.17)	6.06 (4.11-8.79)
Kingdom of Belgium	1.61 (1.46-1.75)	1.44 (1.23-1.59)	38.52 (34.98-42.02)	33.63 (29.84-37.16)	7.51 (6.8-8.19)	8.94 (7.94-9.88)
Kingdom of Bhutan	0.58 (0.33-1.06)	0.52 (0.31-1.18)	14.62 (8.41-26.32)	12.97 (7.35-29.43)	0.93 (0.53-1.68)	1.23 (0.71-2.84)
Kingdom of Cambodia	1.62 (1.01-2.28)	1.66 (1.1-2.3)	46.97 (27.35-67.64)	46.35 (28.88-66.59)	3.06 (1.8-4.44)	4.2 (2.61-5.98)
Kingdom of Denmark	2.19 (1.97-2.39)	1.38 (1.2-1.54)	53.46 (47.88-58.74)	33.1 (29.1-37.2)	9.47 (8.47-10.44)	9 (7.9-10.07)
Kingdom of Eswatini	1.75 (1.17-2.55)	2.53 (1.46-3.81)	40.13 (27.06-57.11)	58.09 (32.68-91.69)	2.79 (1.88-3.99)	4.54 (2.56-7.17)
Kingdom of Lesotho	1.3 (0.9-1.87)	2.6 (1.55-3.79)	29.99 (20.83-43.95)	60.56 (34.57-90.51)	2.01 (1.38-2.97)	4.24 (2.44-6.35)
Kingdom of Morocco	0.26 (0.17-0.36)	0.33 (0.21-0.46)	6.86 (4.7-9.8)	8.75 (5.53-12.65)	0.67 (0.46-0.96)	1.33 (0.83-1.98)
Kingdom of Norway	1.87 (1.71-1.97)	1.24 (1.08-1.34)	44.49 (41.7-47.02)	27.57 (24.71-29.92)	7.58 (7.08-8.03)	7.57 (6.85-8.13)
Kingdom of Saudi Arabia	0.4 (0.28-0.6)	0.48 (0.34-0.65)	10.06 (6.95-15.04)	13.3 (9.41-18.43)	1.1 (0.76-1.64)	2.96 (2.1-4.07)
Kingdom of Spain	1.84 (1.66-2.01)	1.42 (1.2-1.61)	44.99 (40.76-49.35)	34.99 (30.24-39.53)	9.04 (8.18-9.88)	10.6 (9.31-11.81)
Kingdom of Sweden	1.65 (1.48-1.82)	1.36 (1.12-1.59)	38.22 (34.52-41.84)	29.93 (25.22-35.17)	8.71 (7.83-9.53)	9.06 (7.64-10.62)
Kingdom of Thailand	0.83 (0.61-1.06)	0.81 (0.47-1.09)	22.99 (16.78-29.27)	23.45 (13.72-32.18)	2.41 (1.78-3.16)	3.8 (2.24-5.25)
Kingdom of the Netherlands	1.58 (1.42-1.73)	1.28 (1.07-1.44)	35.69 (32.6-38.82)	28.61 (24.87-32.43)	6.26 (5.65-6.86)	6.45 (5.62-7.32)
Kingdom of Tonga	1.54 (0.94-2.07)	1.74 (0.93-2.55)	43.96 (27.12-60.02)	48.18 (25.65-71.26)	4.17 (2.55-5.65)	5.14 (2.74-7.54)
Kyrgyz Republic	2.05 (1.7-2.47)	1.53 (1.19-1.9)	58.3 (48.12-70.39)	43.34 (33-54.4)	5.19 (4.25-6.2)	5.53 (4.2-6.91)

Lao People's Democratic Republic	1.6 (0.87-2.49)	1.35 (0.86-1.86)	46.45 (23.35-73)	38.61 (23.56-55.07)	2.82 (1.45-4.45)	3.16 (1.94-4.53)
Lebanese Republic	0.97 (0.69-1.31)	0.75 (0.55-1.08)	24.64 (16.85-34.69)	19.2 (14.05-27.62)	3.33 (2.28-4.77)	4.8 (3.46-6.92)
Malaysia	1.17 (0.86-1.48)	1.17 (0.88-1.44)	30.95 (23.61-38.45)	30.87 (23.62-37.78)	2.87 (2.23-3.55)	4.09 (3.13-5.03)
Mongolia	1.24 (0.85-1.89)	1.13 (0.79-1.53)	36.07 (24.61-54.41)	31.33 (21.6-43.07)	2.47 (1.7-3.76)	3.34 (2.26-4.65)
Montenegro	1.57 (1.13-2.23)	1.83 (1.32-2.54)	41.73 (29.99-58.58)	44.01 (31.56-62.27)	6.68 (4.78-9.22)	8.88 (6.38-12.6)
New Zealand	1.85 (1.67-2.02)	1.68 (1.47-1.88)	44.68 (40.19-48.78)	41.92 (37.04-46.88)	6.57 (5.91-7.27)	8.57 (7.5-9.67)
North Macedonia	2.25 (1.79-3.02)	2.52 (1.89-3.34)	58.27 (46.24-77.98)	59.29 (43.43-80.46)	6.19 (4.91-8.24)	9.92 (7.17-13.44)
Northern Mariana Islands	2.36 (1.55-3.95)	3.4 (2.62-4.41)	62.67 (40.65-105.46)	93.74 (70.63-122.68)	7.52 (4.83-12.7)	12.49 (9.26-16.66)
Palestine	1.97 (1.37-2.8)	1.87 (1.24-2.45)	52.15 (34.73-75.06)	47.5 (31.1-62.77)	6.33 (4.15-9.06)	8.91 (5.9-11.89)
People's Democratic Republic of Algeria	0.24 (0.16-0.34)	0.24 (0.16-0.35)	6.02 (4.3-8.37)	5.99 (4.09-8.92)	0.67 (0.47-0.93)	1.13 (0.77-1.67)
People's Republic of Bangladesh	0.48 (0.32-0.85)	0.4 (0.23-0.88)	12.77 (8.6-21.67)	10.28 (6.01-23.19)	0.8 (0.54-1.37)	1.03 (0.6-2.34)
People's Republic of China	1.25 (0.91-1.56)	0.64 (0.47-0.88)	36.29 (24.69-45.53)	19.11 (14.2-26.08)	2.81 (1.97-3.53)	3.35 (2.48-4.65)
Plurinational State of Bolivia	2.79 (1.67-3.93)	2.48 (1.46-3.73)	71.64 (42.7-101.79)	61.62 (36.55-93.03)	4.54 (2.68-6.43)	5.48 (3.25-8.37)
Portuguese Republic	2.05 (1.84-2.27)	1.51 (1.27-1.74)	48.98 (43.99-54.56)	36.63 (31.56-42.04)	8.03 (7.16-9.01)	10.48 (9.14-12.05)
Principality of Andorra	0.97 (0.63-1.44)	0.72 (0.46-1.03)	23.72 (15.59-37.1)	17.7 (10.94-26.09)	5.2 (3.29-8.17)	5.18 (3.22-7.77)
Principality of Monaco	0.75 (0.49-1.12)	0.81 (0.53-1.11)	18.09 (11.5-26.95)	19.9 (13.3-27.61)	4.06 (2.6-5.87)	5.52 (3.78-7.72)
Puerto Rico	1.32 (1.21-1.45)	1.73 (1.41-2.05)	32.88 (29.89-36.43)	46.45 (38.03-55.83)	4.13 (3.74-4.59)	8.29 (6.69-10.06)
Republic of Albania	1.29 (0.91-1.8)	1.03 (0.67-1.5)	31.14 (22.48-42.6)	25.13 (16.57-35.95)	3.01 (2.14-4.15)	4.38 (2.9-6.18)
Republic of Angola	0.97 (0.65-1.39)	1.13 (0.71-1.78)	25.45 (17.04-35.63)	28.83 (17.87-44.52)	1.46 (0.98-2.08)	2.14 (1.33-3.28)
Republic of Armenia	2.28 (2.11-2.44)	2.33 (1.97-2.8)	64.17 (59.69-68.54)	59.76 (50.23-71.75)	6.84 (6.34-7.34)	9.31 (7.73-11.25)
Republic of Austria	2.21 (2-2.43)	1.33 (1.13-1.5)	51.34 (46.66-56.37)	31.72 (27.78-35.76)	10.27 (9.17-11.3)	9.3 (8.14-10.49)
Republic of Azerbaijan	1.77 (1.34-2.15)	1.27 (0.92-1.78)	49.54 (37.73-59.76)	34.82 (25.76-48.86)	4.35 (3.3-5.29)	4.4 (3.22-6.31)
Republic of Belarus	1.85 (1.66-2.08)	2.5 (1.99-3.02)	49.98 (44.86-55.75)	67.04 (53.47-82.38)	7.99 (7.14-8.95)	16.6 (13.21-20.47)
Republic of Benin	1.02 (0.75-1.41)	1.21 (0.85-1.63)	25.37 (18.64-35.31)	28.35 (19.55-38.84)	1.6 (1.18-2.23)	2.16 (1.49-2.95)
Republic of Botswana	1.41 (0.94-2.03)	1.67 (1.16-2.44)	33.22 (21.64-49.06)	37.48 (25.14-56)	2.3 (1.49-3.35)	3.16 (2.13-4.72)
Republic of Bulgaria	2.67 (2.18-3.22)	3.38 (2.69-4.11)	74.27 (60.83-89.44)	90.17 (71.43-111.09)	10.89 (8.74-13.13)	16.47 (12.84-20.36)

Republic of Burundi	1.43 (0.76-2.05)	1 (0.61-1.47)	37.31 (19.65-55.44)	24.76 (15.14-36.96)	2.16 (1.15-3.14)	1.67 (1-2.49)
Republic of Cabo Verde	1.57 (1.11-2.1)	1.84 (1.29-2.51)	36.49 (26.48-49)	38.27 (26.87-52.85)	2.9 (2.06-3.88)	4.17 (2.91-5.8)
Republic of Cameroon	1.29 (0.8-1.78)	1.39 (0.9-2.02)	31.6 (19.67-43.51)	32.59 (20.21-47.93)	2.07 (1.29-2.87)	2.57 (1.59-3.73)
Republic of Chad	0.89 (0.61-1.28)	1.1 (0.78-1.57)	21.91 (15.12-31.47)	26.55 (18.68-38.53)	1.34 (0.92-1.94)	1.79 (1.25-2.59)
Republic of Chile	1.18 (1.07-1.28)	0.9 (0.79-0.99)	28.17 (25.38-30.73)	21.57 (19.2-24.02)	2.81 (2.52-3.09)	3.74 (3.31-4.16)
Republic of Colombia	0.99 (0.9-1.08)	1 (0.82-1.19)	25.36 (22.81-27.98)	26.08 (21.15-31.26)	2.2 (1.97-2.44)	3.69 (2.98-4.46)
Republic of Costa Rica	1.08 (0.98-1.18)	1.5 (1.3-1.72)	27.56 (25.17-30.19)	39.52 (34.32-45.45)	3.19 (2.9-3.53)	6.21 (5.35-7.17)
Republic of Croatia	2.24 (1.97-2.55)	1.93 (1.65-2.24)	55.21 (48.86-62.33)	49.71 (42.18-57.86)	11.55 (10.22-12.98)	16.2 (13.76-18.95)
Republic of Cuba	2.45 (2.21-2.67)	3.4 (2.86-3.96)	66.24 (60.11-72.44)	86.65 (72.86-101.67)	8.27 (7.44-9.08)	13.33 (11.16-15.6)
Republic of Cyprus	2.32 (1.6-3.49)	1.53 (1.09-2)	47.94 (34.65-69.92)	33.75 (24.02-44.05)	7.5 (5.47-10.77)	9.23 (6.6-12.31)
Republic of C 么 te d'Ivoire	0.63 (0.48-0.83)	0.72 (0.51-0.99)	15.27 (11.47-19.8)	17.45 (12.09-24.47)	1 (0.76-1.3)	1.39 (0.96-1.94)
Republic of Djibouti	1.23 (0.73-2.03)	1.25 (0.74-2.06)	30.35 (17.55-51.28)	30.19 (17.62-50.77)	1.98 (1.15-3.29)	2.37 (1.4-4.02)
Republic of Ecuador	3 (2.76-3.28)	1.73 (1.35-2.18)	77.46 (71-84.36)	44.23 (34.08-56.61)	5.96 (5.41-6.54)	5.13 (3.9-6.52)
Republic of El Salvador	1.22 (0.95-1.53)	1.33 (1-1.75)	32.36 (25.23-39.71)	35.95 (26.94-47.51)	2.48 (1.95-3.03)	4.46 (3.35-5.94)
Republic of Equatorial Guinea	1.17 (0.79-1.69)	1.33 (0.77-2.08)	30.82 (20.71-44.72)	33.84 (18.88-54.95)	1.74 (1.17-2.5)	3.18 (1.78-5.07)
Republic of Estonia	2.49 (2.21-2.82)	1.78 (1.45-2.1)	63.5 (55.92-73.56)	46.15 (36.96-55.12)	11.03 (9.64-12.8)	13.18 (10.46-15.95)
Republic of Fiji	1.87 (1.27-2.64)	2.46 (1.67-3.34)	53.49 (35.66-76.06)	66.74 (44.49-92.71)	4.52 (3.01-6.38)	6.04 (4.01-8.41)
Republic of Finland	2.17 (1.94-2.38)	1.74 (1.49-1.95)	49.66 (45.26-54.64)	40.03 (35.31-44.97)	8.67 (7.84-9.56)	10.29 (9-11.53)
Republic of Ghana	1.66 (1.13-2.37)	2.43 (1.61-3.39)	39.25 (27.07-56.06)	54.84 (36.71-76.6)	2.65 (1.82-3.79)	4.57 (3.07-6.28)
Republic of Guatemala	1.75 (1.64-1.85)	1.53 (1.32-1.78)	44.42 (41.9-47.15)	40.95 (35.19-47.51)	2.92 (2.73-3.11)	3.86 (3.3-4.54)
Republic of Guinea	0.93 (0.64-1.29)	1.03 (0.71-1.5)	24.1 (16.99-32.67)	26.37 (17.77-37.82)	1.46 (1-1.97)	1.84 (1.25-2.64)
Republic of Guinea-Bissau	1.29 (0.92-1.81)	1.72 (1.21-2.35)	33.29 (23.12-47.13)	41.4 (29-58.1)	1.9 (1.33-2.64)	2.79 (1.95-3.86)
Republic of Guyana	2.38 (2.02-2.76)	3.37 (2.64-4.24)	63.73 (54.19-74.74)	91.98 (70.68-118.84)	4.5 (3.83-5.26)	7.37 (5.66-9.6)
Republic of Haiti	3.08 (2-4.62)	3.08 (1.8-5.13)	83.01 (51.55-125.61)	83.15 (48.44-139.35)	4.73 (2.98-7.21)	5.26 (3.04-8.82)
Republic of Honduras	2.3 (1.73-3.34)	3.79 (2.44-5.42)	62.65 (46.54-88.4)	98.91 (62.46-143.35)	4.28 (3.15-6.11)	8.64 (5.45-12.69)
Republic of Iceland	1.4 (1.25-1.54)	1.23 (1.04-1.41)	34.87 (31.08-38.72)	29.37 (25.43-33.59)	7.99 (7.12-9.01)	8.99 (7.78-10.35)

Republic of India	0.48 (0.34-0.59)	0.53 (0.44-0.69)	12.63 (8.97-15.52)	13.51 (11.19-17.63)	0.83 (0.59-1.01)	1.32 (1.09-1.67)
Republic of Indonesia	1.17 (0.81-1.53)	1.3 (0.81-1.79)	34.44 (22.19-45.15)	37.35 (22.09-51.95)	2.62 (1.71-3.44)	3.65 (2.17-5.13)
Republic of Iraq	0.53 (0.34-0.94)	0.69 (0.48-1)	14.86 (9.16-26.85)	18.64 (12.82-27.08)	1.7 (1.04-3.11)	3.55 (2.39-5.14)
Republic of Italy	0.61 (0.56-0.65)	1.26 (1.09-1.37)	15.57 (14.46-16.6)	34.26 (30.53-37.33)	3.87 (3.57-4.11)	13.04 (11.76-13.98)
Republic of Kazakhstan	2.68 (2.33-3.07)	1.69 (1.42-1.94)	71.81 (62.72-81.66)	45.91 (39.27-52.77)	6.94 (6.05-7.91)	6.66 (5.59-7.7)
Republic of Kenya	0.57 (0.39-0.93)	0.8 (0.57-1.24)	14.2 (9.84-23.95)	19.32 (13.29-30.63)	0.98 (0.68-1.64)	1.56 (1.07-2.43)
Republic of Kiribati	2.27 (0.94-3.19)	2.72 (1.02-4.29)	62.48 (25.22-87.78)	73.37 (26.8-118.13)	3.9 (1.61-5.45)	5.01 (1.88-8)
Republic of Korea	1.35 (0.84-1.83)	0.45 (0.29-0.64)	35.66 (20.63-49.26)	11.87 (7.37-16.86)	2.43 (1.39-3.35)	2.02 (1.23-2.86)
Republic of Latvia	2.4 (2.16-2.66)	2.92 (2.45-3.35)	63.91 (57.26-72.1)	74.22 (61.29-86.54)	10.96 (9.8-12.42)	17.59 (14.46-20.53)
Republic of Liberia	1 (0.74-1.3)	1.26 (0.8-1.84)	24.82 (17.88-32.83)	30.83 (19.41-46.2)	1.54 (1.11-2.04)	2.48 (1.57-3.73)
Republic of Lithuania	2.22 (1.98-2.49)	2.6 (2.17-3.02)	59.19 (52.17-67.09)	67.31 (55.22-79.22)	9.42 (8.28-10.7)	14.17 (11.72-16.58)
Republic of Madagascar	1.16 (0.7-1.53)	1.14 (0.72-1.62)	30.77 (18.58-40.34)	29.27 (18.46-42.04)	1.89 (1.13-2.49)	2.09 (1.31-3.02)
Republic of Malawi	0.76 (0.54-1.01)	0.87 (0.59-1.25)	19.49 (13.96-25.89)	21.39 (14.16-31.33)	1.18 (0.84-1.57)	1.58 (1.06-2.29)
Republic of Maldives	0.46 (0.24-0.7)	0.25 (0.18-0.33)	14.06 (7.01-21.77)	6.53 (4.72-8.75)	1.15 (0.57-1.79)	0.97 (0.7-1.34)
Republic of Mali	0.81 (0.61-1.07)	0.78 (0.54-1.1)	21.27 (15.89-27.54)	20.02 (13.73-28.27)	1.3 (0.98-1.71)	1.47 (1.01-2.05)
Republic of Malta	1.94 (1.73-2.17)	1.74 (1.49-2.03)	45.51 (40.78-51.35)	41.73 (36.19-48.38)	8.17 (7.3-9.2)	10.67 (9.2-12.68)
Republic of Mauritius	2.84 (2.61-3.07)	2.27 (2.05-2.44)	77.59 (71.69-83.93)	63.98 (57.79-69.18)	8.36 (7.66-9.06)	8.74 (7.78-9.55)
Republic of Moldova	2.12 (1.99-2.27)	1.85 (1.67-2.08)	58.44 (54.74-62.92)	52.22 (46.7-59.15)	7.98 (7.37-8.61)	10.6 (9.32-12.07)
Republic of Mozambique	1.29 (0.69-2.1)	1.55 (0.85-2.63)	32.9 (17.33-54.2)	37.75 (20.2-65.12)	1.95 (1.03-3.18)	2.59 (1.38-4.41)
Republic of Namibia	1.11 (0.79-1.58)	1.32 (0.85-1.96)	27.05 (19.82-37.55)	31.56 (19.66-47.13)	1.84 (1.35-2.58)	2.78 (1.74-4.2)
Republic of Nauru	2.21 (1.07-3.52)	3.19 (1.65-4.86)	64 (29.88-103.94)	89.65 (44.18-140.96)	4.95 (2.3-8.06)	7.77 (3.82-12.27)
Republic of Nicaragua	0.58 (0.44-0.78)	0.65 (0.48-0.86)	15.18 (11.8-19.67)	17.22 (12.6-22.79)	1.24 (0.97-1.59)	1.99 (1.46-2.67)
Republic of Niue	2.13 (1.24-2.92)	2.38 (1.18-4.15)	55.29 (32-76.56)	63.66 (31.16-112.77)	5.13 (3-7.26)	7.07 (3.44-12.37)
Republic of Palau	0.33 (0.23-0.47)	0.32 (0.23-0.42)	8.78 (5.83-12.71)	8.09 (5.77-11.02)	0.84 (0.56-1.24)	0.87 (0.61-1.2)
Republic of Panama	1.03 (0.92-1.14)	1.52 (1.2-1.82)	26.04 (23.36-29.12)	40.2 (31.42-48.59)	2.52 (2.24-2.81)	5.6 (4.38-6.84)
Republic of Paraguay	1.83 (1.36-2.32)	1.87 (1.32-2.65)	45.21 (33.01-57.59)	44.92 (31.33-64.35)	3.96 (2.86-5.06)	5.1 (3.56-7.37)

Republic of Peru	1.76 (1.37-2.23)	1.43 (0.96-1.96)	46.07 (35.41-58.4)	37.43 (25.05-52.69)	3.55 (2.7-4.51)	5.13 (3.41-7.33)
Republic of Poland	2.4 (2.27-2.52)	3.02 (2.67-3.34)	60.67 (57.77-63.6)	67.37 (59.28-75.06)	6.97 (6.59-7.4)	12.52 (10.96-13.97)
Republic of Rwanda	1.75 (0.95-2.39)	1.34 (0.89-1.93)	46.37 (24.49-64.15)	32.46 (21.38-47.15)	2.61 (1.39-3.6)	2.5 (1.64-3.61)
Republic of San Marino	0.46 (0.33-0.67)	0.28 (0.17-0.43)	10.54 (7.53-15.2)	7.06 (3.84-11.22)	2.41 (1.73-3.53)	2.01 (1.1-3.19)
Republic of Senegal	1.01 (0.76-1.35)	1.41 (0.99-1.94)	24.99 (18.62-33.27)	33.45 (23.15-46.05)	1.63 (1.22-2.17)	2.66 (1.84-3.69)
Republic of Serbia	2.32 (1.63-3.2)	2.03 (1.4-2.85)	54.69 (38.64-76.73)	50.47 (34.28-71.81)	6.06 (4.28-8.58)	8.72 (5.83-12.41)
Republic of Seychelles	1.53 (1.19-1.97)	1.27 (0.97-1.76)	43.21 (33.22-56.5)	34.56 (26.51-47.2)	4.21 (3.2-5.58)	4.35 (3.29-5.86)
Republic of Sierra Leone	0.77 (0.57-1.04)	1.09 (0.77-1.54)	18.47 (13.39-25.65)	26.06 (18.21-36.38)	1.18 (0.86-1.61)	1.96 (1.36-2.73)
Republic of Singapore	1.16 (1.05-1.29)	0.75 (0.65-0.85)	30.48 (27.62-33.69)	19.46 (17.11-21.97)	2.41 (2.17-2.66)	3.01 (2.66-3.44)
Republic of Slovenia	2.35 (2.09-2.64)	1.86 (1.5-2.25)	58.09 (51.73-65.41)	44.56 (35.41-55)	8.7 (7.64-9.99)	10.96 (8.74-13.6)
Republic of South Africa	0.96 (0.71-1.31)	1.55 (1.08-1.84)	23.2 (17.78-31.51)	35.6 (26.1-41.64)	1.84 (1.41-2.52)	3.47 (2.53-4.11)
Republic of South Sudan	1.02 (0.61-1.47)	1.22 (0.73-1.77)	25.95 (15.11-37.98)	31.6 (18.67-46.58)	1.62 (0.95-2.35)	2.27 (1.34-3.33)
Republic of Sudan	0.4 (0.26-0.73)	0.46 (0.28-0.72)	11.23 (7.16-19.58)	13.21 (8.09-20.62)	0.96 (0.6-1.73)	1.77 (1.05-2.76)
Republic of Suriname	1.18 (0.96-1.55)	1.33 (0.93-1.82)	31.22 (24.97-41.14)	35.93 (25.36-50.11)	2.47 (1.95-3.27)	3.2 (2.24-4.4)
Republic of Tajikistan	1.65 (1.18-2.24)	1.21 (0.73-1.89)	47.65 (33.44-64.55)	35.38 (20.62-59.01)	3.84 (2.7-5.19)	3.45 (2.01-5.7)
Republic of the Congo	1.58 (1.14-2.18)	1.55 (1.07-2.12)	41.7 (28.97-58.03)	40.46 (26.75-57.55)	2.54 (1.77-3.54)	3.18 (2.13-4.55)
Republic of the Gambia	0.73 (0.53-0.99)	1.01 (0.7-1.42)	17.48 (12.89-23.79)	25.06 (17.48-35.58)	1.19 (0.87-1.62)	2.01 (1.4-2.84)
Republic of the Marshall Islands	2.23 (1.35-3.45)	2.87 (1.2-5.96)	59.93 (36.4-92.23)	83.01 (34.82-169.37)	4.32 (2.63-6.77)	6.55 (2.74-13.23)
Republic of the Niger	0.88 (0.58-1.26)	1.04 (0.7-1.46)	21.25 (14.43-30.24)	24.59 (16.4-34.95)	1.28 (0.86-1.82)	1.71 (1.14-2.41)
Republic of the Philippines	1.03 (0.78-1.35)	1.25 (0.96-1.76)	30 (21.57-37.69)	36.32 (27.71-48.83)	2.64 (1.89-3.33)	3.55 (2.71-4.71)
Republic of the Union of Myanmar	1.58 (0.96-2.37)	1.35 (0.92-1.82)	46.7 (26.51-72.64)	38.84 (24.78-53.42)	3.07 (1.77-4.67)	3.54 (2.26-4.96)
Republic of Trinidad and Tobago	2.75 (2.55-2.93)	3.12 (2.4-3.97)	70.5 (65.79-75.18)	83.29 (62.67-107.83)	6.38 (5.91-6.89)	9.21 (6.98-11.83)
Republic of Tunisia	0.46 (0.32-0.58)	0.48 (0.31-0.69)	11.57 (8.66-14.92)	12.31 (8.17-18.12)	1.54 (1.15-2)	2.69 (1.77-4)
Republic of Turkey	1.45 (1-1.9)	1.16 (0.77-1.54)	38.1 (25.92-49.66)	29.97 (19.12-40.21)	4.18 (2.86-5.43)	6.75 (4.32-8.97)
Republic of Uganda	1.45 (1-2.03)	2.07 (1.5-2.83)	35.23 (23.94-49.47)	49.46 (34.81-68.49)	2.28 (1.57-3.2)	3.84 (2.71-5.29)
Republic of Uzbekistan	1.33 (1.13-1.53)	0.99 (0.8-1.18)	37.76 (31.61-43.61)	29.48 (23.58-35.43)	3.39 (2.85-3.94)	3.4 (2.72-4.11)

Republic of Vanuatu	1.4 (0.78-2.1)	1.92 (1.02-2.77)	38.58 (20.99-59.84)	53.78 (28.29-77.89)	2.86 (1.58-4.43)	4.14 (2.23-5.98)
Republic of Yemen	0.41 (0.25-0.74)	0.46 (0.29-0.81)	10.66 (6.39-18.87)	12.66 (7.86-21.45)	0.93 (0.56-1.67)	1.49 (0.91-2.47)
Republic of Zambia	1.33 (0.78-1.89)	1.71 (0.87-3.25)	36.01 (20.97-51.34)	43.7 (21.81-87.36)	2.16 (1.24-3.06)	3.32 (1.67-6.61)
Republic of Zimbabwe	1.76 (1.31-2.26)	3.24 (2.09-4.59)	40.51 (29.18-51.84)	80.59 (50.15-114.43)	2.98 (2.19-3.86)	5.78 (3.62-8.11)
Romania	1.78 (1.59-1.98)	1.8 (1.52-2.12)	48.27 (42.98-53.93)	47.87 (39.81-56.71)	4.93 (4.36-5.6)	8.44 (6.84-10.11)
Russian Federation	3.12 (2.98-3.22)	3.02 (2.69-3.3)	86.7 (83.25-90.15)	81.94 (72.82-90.86)	14.3 (13.78-14.77)	19.96 (17.7-22.05)
Saint Kitts and Nevis	3.46 (3.13-3.78)	3.5 (2.95-4.06)	89.88 (81.5-98.59)	80.05 (66.6-94.35)	7.02 (6.34-7.75)	8.63 (7.16-10.28)
Saint Lucia	2.42 (2.26-2.6)	2.32 (1.92-2.81)	61.8 (57.23-66.42)	59.28 (48.52-72.6)	5.44 (5.04-5.84)	6.52 (5.34-7.98)
Saint Vincent and the Grenadines	2.49 (2.13-2.9)	2.25 (1.84-2.74)	62.69 (53.84-72.73)	60.06 (49.58-74.23)	5.55 (4.78-6.47)	5.98 (4.93-7.34)
Slovak Republic	3.55 (2.68-4.6)	2.71 (1.85-3.72)	90.07 (66.08-117.33)	65.64 (44.97-91.55)	9.79 (7.2-12.69)	11.25 (7.62-15.73)
Socialist Republic of Viet Nam	0.63 (0.45-0.83)	0.61 (0.43-0.8)	15.59 (11.71-21.2)	15.38 (10.91-21.6)	1.38 (1.04-1.88)	2.1 (1.45-2.92)
Solomon Islands	1.44 (0.67-2.15)	2.02 (1.1-3.03)	40.69 (17.47-62.87)	58.42 (30.57-88.53)	2.8 (1.19-4.33)	4.45 (2.34-6.86)
State of Eritrea	1.78 (1.12-2.36)	1.95 (1.27-2.67)	45.39 (28.01-60.9)	47.15 (30.53-65.91)	2.62 (1.61-3.5)	3.23 (2.05-4.51)
State of Israel	1.39 (1.24-1.53)	1.35 (1.14-1.53)	33.04 (29.27-37.1)	30.41 (26.14-34.67)	5.48 (4.82-6.14)	7.32 (6.27-8.39)
State of Kuwait	0.57 (0.5-0.64)	0.99 (0.83-1.17)	14.29 (12.58-16.3)	28.51 (23.71-33.84)	2.76 (2.44-3.16)	8.61 (7.14-10.18)
State of Libya	0.76 (0.53-1.05)	1 (0.66-1.43)	19.94 (14.13-27.31)	27.09 (18-38.92)	2.56 (1.8-3.51)	4.96 (3.25-7.14)
State of Qatar	1.02 (0.67-1.6)	1.04 (0.76-1.48)	23.55 (15.54-36.68)	24.1 (17.28-34.87)	3.44 (2.27-5.46)	6.66 (4.79-9.62)
Sultanate of Oman	0.22 (0.15-0.31)	0.2 (0.15-0.27)	5.53 (3.84-7.89)	5.14 (3.77-6.96)	0.72 (0.48-1.03)	1.18 (0.86-1.61)
Swiss Confederation	1.76 (1.56-1.95)	1.14 (0.96-1.32)	41.33 (36.75-45.4)	27.2 (23.66-31.18)	9.16 (8.18-10.17)	8.18 (7.19-9.32)
Syrian Arab Republic	0.59 (0.44-0.82)	0.55 (0.39-0.81)	16.56 (12.26-22.58)	15.61 (10.97-22.72)	1.88 (1.41-2.54)	3.17 (2.22-4.67)
Taiwan (Province of China)	0.36 (0.32-0.39)	0.71 (0.62-0.81)	10.33 (9.31-11.41)	22.52 (19.66-25.44)	1.66 (1.5-1.83)	5.53 (4.79-6.27)
Togolese Republic	1.02 (0.76-1.39)	1.45 (0.97-2.01)	25.28 (18.91-35.11)	33.7 (22.06-47.25)	1.67 (1.26-2.3)	2.65 (1.71-3.74)
Tokelau	2.18 (1.28-3.07)	2.14 (1.16-3.23)	60.83 (35.09-88)	60.72 (32.49-92.37)	4.96 (2.89-7.09)	6.51 (3.5-9.78)
Turkmenistan	1.26 (1.14-1.38)	0.82 (0.63-1.07)	35.57 (32.28-38.9)	23.97 (18.25-31.89)	3 (2.71-3.3)	2.74 (2.06-3.68)
Tuvalu	2.41 (1.47-3.25)	2.4 (1.24-3.69)	66.26 (40.2-91.05)	65.92 (33.58-103.52)	4.67 (2.8-6.4)	5.89 (3.05-9.16)
Ukraine	2.5 (2.28-2.75)	2.66 (1.8-3.76)	66.45 (60.85-72.98)	73.09 (48.32-105.07)	7.28 (6.61-8)	9.63 (6.36-13.91)

Union of the Comoros	1.42 (0.83-2.05)	1.45 (0.96-2.04)	37.12 (21.15-53.17)	36.42 (23.95-51.92)	2.32 (1.28-3.31)	2.75 (1.82-3.94)
United Arab Emirates	2.28 (1.34-3.41)	2.24 (1.62-3.07)	57.38 (33.23-85.38)	49.23 (36.13-67.21)	6.78 (3.86-10.25)	8.53 (6.21-11.75)
United Kingdom of Great Britain and Northern Ireland	1.54 (1.45-1.59)	1.81 (1.63-1.92)	35.66 (34.03-37.19)	41.65 (38.63-44.03)	6.53 (6.29-6.7)	9.83 (9.16-10.22)
United Mexican States	0.85 (0.82-0.88)	0.97 (0.83-1.13)	20.5 (19.87-21.09)	27.18 (22.87-31.8)	1.71 (1.66-1.76)	3.26 (2.74-3.84)
United Republic of Tanzania	1.29 (0.77-1.81)	1.26 (0.84-1.75)	32.87 (19.37-47.61)	31.51 (20.93-43.76)	2.14 (1.28-3.06)	2.46 (1.62-3.39)
United States of America	1.72 (1.57-1.8)	1.98 (1.78-2.1)	42.89 (40.13-45.48)	53.78 (49.82-57.73)	11.37 (10.74-11.74)	17.44 (16.37-18.25)
United States Virgin Islands	2.21 (1.72-2.88)	1.29 (0.87-2.06)	55.71 (42.82-72.44)	34.21 (22.65-56.61)	5.87 (4.54-7.59)	4.22 (2.77-7)

ASMR= age-standardized mortality rate; DALY= disability-adjusted life year; ASDR= age-standardized DALY rate; ASIR= age-standardized incidence rate; SDI= socio-demographic index; Data in parentheses represent the 95% uncertainty intervals.

Table S6. The all-age deaths, DALYs, incident cases, ASMR, ASDR, and ASIR per 100,000 population attributable to gynecological cancers in 1980/1990 and 2021 and the EAPC of ASR from 1980/1990 to 2021 by country in Asia.

Country	1980/1990		2021		EAPC for ASR[b]
	Counts[a]	ASR[a]	Counts[a]	ASR[a]	
**Deaths**					
Cervical cancer					
Afghanistan	515.21 (247.56-838.49)	6.29 (3.22-10.17)	645.79 (336.29-964.89)	4.81 (2.59-7.05)	-0.6 (-0.71--0.48)
Armenia	127.69 (114.51-140.37)	5.41 (4.84-5.95)	118.65 (105.22-134.45)	2.84 (2.51-3.21)	-1.18 (-1.45--0.91)
Azerbaijan	195.29 (152.25-240.14)	4.58 (3.58-5.64)	247.79 (189.63-310.53)	2.25 (1.74-2.8)	-1.66 (-1.78--1.54)
Bahrain	3.15 (2.42-4.1)	2.47 (1.92-3.2)	9.65 (7.24-12.7)	1.07 (0.82-1.38)	-2.23 (-2.56--1.89)
Bangladesh	4347.58 (2676.88-6234.64)	9.27 (5.76-13.25)	5634.33 (3981.78-7846.27)	3.84 (2.7-5.31)	-2.32 (-2.44--2.2)

Bhutan	21.76 (12.62-32.26)	10.03 (6.09-15.04)	27.88 (18.66-37.75)	4.31 (2.92-5.84)	-2.43 (-2.58--2.27)
Brunei Darussalam	7.89 (6.11-10.32)	8.12 (6.2-10.59)	15.99 (12.52-19.91)	4.02 (3.22-4.9)	-1.7 (-1.87--1.54)
Cambodia	410.04 (298.32-577.45)	9.2 (6.7-12.77)	733.09 (543.22-1022.1)	5.36 (4.04-7.44)	-1.5 (-1.58--1.42)
China	33645.65 (25747.38-42463.67)	5 (3.88-6.25)	49841.19 (36878.07-64386.31)	2.39 (1.77-3.08)	-1.56 (-1.75--1.38)
Democratic People's Republic of Korea	635.59 (453.54-898.37)	4.7 (3.4-6.53)	1396.91 (982.34-2016.48)	4.11 (2.91-5.87)	-0.12 (-0.2--0.04)
Georgia	319.41 (282.08-356.7)	5.9 (5.22-6.58)	238.96 (207.04-275.1)	4.31 (3.74-4.95)	-0.82 (-1.22--0.41)
India	40795.55 (32856.42-50121.8)	9.56 (7.64-11.84)	60040.82 (51584.35-69062.11)	4.71 (4.06-5.41)	-2 (-2.19--1.8)
Indonesia	4426.9 (3108.7-6082.81)	4.93 (3.5-6.88)	10302.39 (7446.36-13508.82)	3.79 (2.77-4.89)	-0.54

					(-0.67--0.41)
Iran (Islamic Republic of)	273.15 (208.69-476.89)	1.3 (1-2.26)	554.62 (478.07-628.6)	0.68 (0.59-0.77)	-1.83 (-1.93--1.72)
Iraq	73.68 (54.52-114.38)	1.06 (0.79-1.65)	253.91 (181.32-340.67)	0.93 (0.67-1.25)	-0.5 (-0.64--0.35)
Japan	4042.57 (3861.89-4151.95)	3.23 (3.06-3.33)	3891.34 (3288.82-4253.26)	1.42 (1.29-1.49)	-1.71 (-1.93--1.48)
Jordan	20.64 (15.26-31.06)	1.95 (1.44-2.99)	57.09 (42.04-75.8)	0.69 (0.52-0.89)	-2.81 (-3.18--2.44)
Kazakhstan	934.8 (838.59-1046.77)	8.17 (7.3-9.16)	632.69 (539.92-738.36)	3.31 (2.82-3.86)	-1.92 (-2.23--1.6)
Kuwait	7.08 (6.43-7.76)	1.37 (1.24-1.49)	14.81 (12.53-17.52)	0.39 (0.33-0.46)	-3.46 (-3.88--3.03)
Kyrgyzstan	232.89 (198.17-270.04)	8.81 (7.5-10.14)	245.94 (199.7-300.14)	4.56 (3.73-5.48)	-1.44 (-1.58--1.3)

Lao People's Democratic Republic	208 (140.41-295.47)	10.66 (7.34-15.13)	243.8 (175.96-323.86)	4.48 (3.29-5.89)	-2.24 (-2.33--2.16)
Lebanon	41 (29.81-55.77)	2.28 (1.68-3.07)	53.7 (41.94-66.4)	0.89 (0.7-1.1)	-2.6 (-2.75--2.45)
Malaysia	515.15 (369.99-682.95)	6.55 (4.69-8.72)	1367.09 (1057.52-1595.83)	4.7 (3.62-5.48)	-1.33 (-1.52--1.15)
Maldives	6.23 (2.43-9.44)	6.69 (2.96-10.08)	4.41 (3.34-5.63)	1.17 (0.9-1.5)	-4.56 (-4.73--4.4)
Mauritius	42.1 (39.25-45.02)	6.91 (6.44-7.37)	56 (50.35-59.36)	3.15 (2.84-3.34)	-2.14 (-2.46--1.83)
Mongolia	78.38 (50.23-121.17)	7.67 (4.92-11.92)	139.67 (103.64-184.29)	5.46 (4.03-7.18)	-0.72 (-0.92--0.52)
Myanmar	1944.96 (1330.42-2786.17)	8.9 (6.15-12.69)	2064.04 (1490.57-2918.85)	3.92 (2.83-5.55)	-2.15 (-2.33--1.96)
Nepal	897.96 (567.42-1282.64)	9.89 (6.41-14.2)	1225.96 (844.76-1640.7)	4.91 (3.44-6.56)	-2.15

					(-2.44--1.86)
Oman	13.07 (8.81-20.2)	2.1 (1.46-3.26)	16.88 (12.67-21.73)	0.72 (0.55-0.92)	-2.49 (-2.58--2.4)
Pakistan	935.18 (703.37-1225.92)	1.89 (1.44-2.44)	3385.61 (2417.84-4577.84)	2.25 (1.61-3.01)	0.45 (0.16-0.74)
Palestine	7.5 (5.17-10.85)	0.98 (0.67-1.42)	13.49 (10.72-16.53)	0.51 (0.41-0.61)	-1.6 (-1.74--1.45)
Philippines	941.58 (815.77-1153.78)	3.46 (2.99-4.32)	3147.37 (2436.43-3967.21)	3.38 (2.65-4.22)	0.05 (-0.01-0.12)
Qatar	1.17 (0.78-1.84)	1.64 (1.11-2.4)	7.04 (5.19-9.94)	0.77 (0.59-0.99)	-1.68 (-2.17--1.18)
Republic of Korea	1515.7 (1190.13-1960.71)	6.09 (4.82-7.79)	1074.61 (831.54-1386.28)	1.23 (0.96-1.57)	-3.88 (-4.02--3.74)
Saudi Arabia	30.25 (20.41-45.16)	0.64 (0.43-0.93)	103.92 (75.28-142.58)	0.35 (0.26-0.46)	-1.25 (-1.55--0.95)

Seychelles	5.86 (4.9-6.87)	12.08 (10.08-14.17)	7.93 (6.6-9.33)	6.68 (5.56-7.86)	-1.42 (-1.53--1.32)
Singapore	86.59 (79.18-94.36)	5.3 (4.86-5.78)	92.14 (82.04-101.32)	1.09 (0.97-1.2)	-4.25 (-4.47--4.04)
Sri Lanka	297.95 (247.97-361.77)	3.27 (2.71-3.96)	525.06 (341.81-734.84)	1.95 (1.28-2.71)	-1.06 (-1.17--0.95)
Syrian Arab Republic	44.88 (33.51-70.93)	0.95 (0.7-1.55)	100.85 (69.06-139.02)	0.72 (0.5-0.98)	-1.16 (-1.39--0.93)
Taiwan (Province of China)	734.1 (675.55-794.97)	5.93 (5.47-6.4)	793.97 (692.86-895.71)	1.96 (1.71-2.21)	-3.62 (-4.01--3.24)
Tajikistan	98.73 (76.13-124.34)	4.16 (3.17-5.2)	130.2 (90.51-185.95)	1.93 (1.39-2.66)	-1.93 (-2.04--1.82)
Thailand	2809.4 (2135.14-3464.32)	10.25 (7.65-12.56)	4617.49 (3484.21-5994.67)	4.43 (3.34-5.7)	-2.08 (-2.25--1.9)
Timor-Leste	17.9 (12.18-27.94)	6.49 (4.51-9.87)	37.03 (27.7-50.17)	4.08 (3.05-5.5)	-1.24

					(-1.42--1.06)
Turkey	569.55 (423.48-827.49)	1.99 (1.48-2.9)	923.43 (712.52-1185.56)	0.98 (0.76-1.26)	-2.09 (-2.28--1.89)
Turkmenistan	113.5 (99.93-128.36)	6.59 (5.8-7.46)	180.72 (134.19-241.38)	3.95 (2.96-5.26)	-0.98 (-1.31--0.64)
United Arab Emirates	13.5 (9.07-21.56)	5.03 (3.42-8.08)	77.87 (58.94-102.92)	2.51 (1.82-3.38)	-0.83 (-1.18--0.49)
Uzbekistan	514.07 (463.2-581.21)	5.17 (4.67-5.84)	1158.76 (958.21-1394.14)	3.8 (3.15-4.57)	-0.95 (-1.15--0.75)
Viet Nam	2276.46 (1672.13-3098.45)	6.56 (4.86-8.94)	4369 (3345.03-5713.47)	4.29 (3.33-5.54)	-0.86 (-0.94--0.79)
Yemen	112.5 (71.36-195.2)	2.68 (1.75-4.57)	277 (182.45-398.5)	1.65 (1.14-2.35)	-1.31 (-1.37--1.25)
Ovarian cancer					
Afghanistan	68.89 (29.54-173.23)	0.94 (0.38-2.23)	174.23 (86.21-388.24)	1.57 (0.69-3.17)	1.28

					(1.11-1.45)
Armenia	46.02 (36.26-58.34)	1.98 (1.53-2.48)	125.79 (103.39-149.95)	2.89 (2.38-3.44)	1.18 (1.06-1.3)
Azerbaijan	47.27 (26.66-76.09)	1.12 (0.63-1.8)	171.64 (110.57-253.78)	1.55 (1-2.29)	1.2 (1-1.4)
Bahrain	3.26 (2.2-4.34)	2.76 (1.86-3.71)	28.63 (18.71-37.51)	3.25 (2.21-4.23)	0.25 (0.09-0.4)
Bangladesh	411.44 (202.04-671.79)	0.95 (0.45-1.5)	2301.35 (1449.99-4002.75)	1.6 (1.03-2.78)	1.23 (1.19-1.28)
Bhutan	1.94 (0.98-3.27)	1.05 (0.5-1.77)	11.72 (7.79-21.03)	1.88 (1.26-3.36)	1.41 (1.34-1.48)
Brunei Darussalam	2.57 (1.72-4.68)	2.86 (1.96-4.9)	13.47 (10.4-16.58)	3.47 (2.7-4.24)	0.69 (0.62-0.77)
Cambodia	55.4 (32.03-105.22)	1.34 (0.79-2.42)	426.36 (265.67-633.22)	3.23 (2.05-4.74)	2.33 (2.28-2.39)

China	8487.12 (5941.49-12837.92)	1.29 (0.93-1.9)	25143.85 (18525.7-32922.74)	1.18 (0.87-1.55)	-0.52 (-0.68--0.35)
Democratic People's Republic of Korea	156.62 (92.88-232.32)	1.21 (0.72-1.79)	517.15 (343.38-743.67)	1.54 (1.02-2.21)	0.64 (0.6-0.69)
Georgia	60.17 (47.34-74.34)	1.1 (0.86-1.35)	305.95 (259.77-354.16)	5.28 (4.47-6.11)	4.73 (4.25-5.2)
India	3992.79 (2591.14-5685.83)	1.07 (0.7-1.49)	23219.14 (19715.06-27527.72)	1.9 (1.61-2.25)	1.47 (1.4-1.54)
Indonesia	862.43 (535.04-1582.67)	1.04 (0.66-1.86)	5296.21 (3519.94-8958.22)	2.02 (1.36-3.44)	1.51 (1.32-1.7)
Iran (Islamic Republic of)	89.75 (25.56-140.19)	0.44 (0.12-0.69)	913.6 (628.39-1059.06)	1.13 (0.78-1.32)	2.46 (2.29-2.64)
Iraq	65.44 (38.39-106.37)	0.99 (0.57-1.6)	440.8 (310.05-601.37)	1.69 (1.23-2.24)	1.24 (1.18-1.31)
Japan	2488.94 (2399.74-2545.6)	1.93 (1.85-1.97)	5686.43 (4746.32-6260.27)	1.82 (1.63-1.94)	-0.24

					(-0.38--0.11)
Jordan	9.11 (3.64-13.91)	1 (0.38-1.51)	118.66 (74.01-157.47)	1.5 (0.94-2)	0.98 (0.73-1.22)
Kazakhstan	307.11 (248.34-373.6)	2.7 (2.18-3.29)	539.53 (441.79-651.43)	2.89 (2.38-3.49)	0.49 (0.27-0.71)
Kuwait	8.46 (7.78-9.15)	1.98 (1.83-2.17)	38.13 (32.16-45.77)	1.08 (0.91-1.29)	-1.32 (-1.6--1.05)
Kyrgyzstan	45.71 (36.38-57.1)	1.76 (1.41-2.2)	164.92 (129.92-203.37)	3.15 (2.46-3.91)	1.81 (1.26-2.37)
Lao People's Democratic Republic	22.66 (11.64-49.36)	1.29 (0.68-2.62)	126.43 (79.52-201.85)	2.45 (1.58-3.87)	1.79 (1.73-1.85)
Lebanon	29.61 (20.77-47.92)	1.77 (1.25-2.8)	149.31 (98.34-195.29)	2.47 (1.64-3.22)	0.99 (0.87-1.11)
Malaysia	92.16 (63.78-149.97)	1.22 (0.85-1.95)	543.94 (432.93-775.3)	1.87 (1.48-2.66)	1.47 (1.24-1.69)

Maldives	0.93 (0.43-3.34)	1.15 (0.57-3.64)	6.36 (4.63-8.27)	1.69 (1.29-2.13)	1.21 (0.9-1.52)
Mauritius	5.62 (5.22-6.08)	0.92 (0.85-0.99)	54.01 (48.77-58.01)	2.98 (2.69-3.19)	2.15 (1.73-2.56)
Mongolia	15.9 (8.14-26.14)	1.55 (0.79-2.53)	63.23 (44.51-82.83)	2.5 (1.75-3.29)	1.22 (1.11-1.34)
Myanmar	287.82 (164.47-509.34)	1.43 (0.84-2.45)	1263.57 (902.03-1782.56)	2.46 (1.77-3.48)	1.19 (1.08-1.3)
Nepal	81.24 (37.15-132.8)	1.05 (0.45-1.71)	463.94 (313.29-795.64)	1.93 (1.32-3.28)	1.42 (1.36-1.47)
Oman	3.16 (1.06-5.33)	0.61 (0.19-1.01)	15.43 (10.75-20.43)	0.74 (0.53-0.98)	0.97 (0.76-1.18)
Pakistan	720.47 (367.35-968.93)	1.56 (0.8-2.1)	4588.99 (2801.44-6734.42)	3.43 (2.09-4.98)	1.99 (1.82-2.16)
Palestine	9.18 (5.35-14.4)	1.22 (0.71-1.89)	48.28 (34.64-60.49)	1.84 (1.33-2.31)	1.11

					(1.05-1.17)
Philippines	435.93 (304.66-539.66)	1.71 (1.18-2.12)	2502.47 (1918.86-3183.27)	2.8 (2.17-3.52)	1.2 (1.16-1.24)
Qatar	1.4 (0.95-2.1)	2.14 (1.43-3.23)	20.09 (12.76-27.73)	2.3 (1.58-3.03)	0.45 (0.24-0.66)
Republic of Korea	270.57 (196.25-432.51)	1.16 (0.83-1.85)	1355.6 (848.32-1625.71)	1.5 (0.95-1.78)	0.74 (0.55-0.93)
Saudi Arabia	32.64 (16.7-61.43)	0.78 (0.38-1.49)	283.99 (195.35-435.48)	1.2 (0.84-1.9)	1.31 (1.08-1.54)
Seychelles	1.18 (0.96-1.71)	2.41 (1.93-3.49)	4.35 (3.38-5.26)	3.68 (2.88-4.45)	1.51 (1.32-1.69)
Singapore	41.19 (38.18-44.19)	2.57 (2.4-2.76)	145.36 (131.41-159.51)	1.7 (1.53-1.87)	-1.01 (-1.18--0.83)
Sri Lanka	90.29 (70.32-120.04)	1.01 (0.8-1.36)	466.21 (279.7-639.87)	1.72 (1.04-2.37)	1.6 (1.48-1.72)

Syrian Arab Republic	25.23 (10.73-36.26)	0.58 (0.23-0.86)	133.74 (90.32-190.72)	0.96 (0.65-1.34)	1.07 (0.96-1.19)
Taiwan (Province of China)	102.52 (94.02-110.42)	0.85 (0.78-0.92)	676.18 (592.04-754.16)	1.68 (1.47-1.87)	1.57 (1.32-1.81)
Tajikistan	19.34 (12.1-29.12)	0.84 (0.52-1.28)	57.9 (41.29-76.53)	0.85 (0.61-1.13)	-0.04 (-0.16-0.09)
Thailand	420.9 (252.52-636.37)	1.64 (1-2.44)	2345.28 (1463.15-3163.37)	2.21 (1.37-3)	0.75 (0.66-0.83)
Timor-Leste	2.32 (1.19-4.44)	0.94 (0.48-1.75)	16.17 (10.56-22.43)	1.8 (1.17-2.48)	1.7 (1.56-1.84)
Turkey	544.34 (339.36-814.74)	2.04 (1.27-3.04)	2312.47 (1631.49-2964.26)	2.46 (1.74-3.13)	0.26 (0.14-0.38)
Turkmenistan	17.5 (14.68-21.86)	1.01 (0.84-1.27)	94.54 (72.17-126.58)	2.11 (1.62-2.81)	2.19 (0.93-3.46)
United Arab Emirates	6.59 (3.79-14.15)	2.68 (1.54-5.74)	126.34 (94.78-178.67)	4.41 (3.44-5.63)	2.15

					(1.78-2.52)
Uzbekistan	85.83 (64.71-118.24)	0.87 (0.64-1.18)	425.52 (328.05-535.56)	1.45 (1.12-1.82)	1.07<br>(0.89-1.25)
Viet Nam	283.44 (182.66-412.78)	0.84 (0.54-1.22)	1451.58 (945.75-1973.38)	1.44 (0.94-1.94)	1.4<br>(1.35-1.45)
Yemen	19.89 (2.51-42.46)	0.53 (0.06-1.16)	132.64 (62.12-216.69)	0.86 (0.39-1.4)	1.27<br>(1.08-1.47)
Uterine cancer					
Afghanistan	69.27 (32.49-138.75)	0.97 (0.49-1.92)	136.07 (66.7-236.7)	1.36 (0.69-2.39)	0.92<br>(0.78-1.06)
Armenia	43.08 (36.27-50.3)	1.93 (1.62-2.26)	102.44 (86.26-123.07)	2.33 (1.97-2.8)	0.09<br>(-0.24-0.42)
Azerbaijan	70.04 (51.94-88.92)	1.77 (1.3-2.26)	132.03 (97.23-185.12)	1.27 (0.92-1.78)	-0.93<br>(-1.03--0.83)
Bahrain	1.04 (0.77-1.55)	1.03 (0.76-1.58)	8.13 (5.54-11.98)	1.11 (0.77-1.55)	0.28

					(-0.02-0.57)
Bangladesh	209.1 (132.73-358.42)	0.51 (0.33-0.88)	537.81 (314-1196.6)	0.4 (0.23-0.88)	-0.78<br>(-0.85--0.71)
Bhutan	0.97 (0.51-1.65)	0.59 (0.32-1.05)	3.09 (1.81-7.05)	0.52 (0.31-1.18)	-0.47<br>(-0.55--0.38)
Brunei Darussalam	1.28 (0.78-2.08)	1.57 (1.01-2.49)	5.3 (3.84-7.13)	1.51 (1.1-2.01)	0.19<br>(0.05-0.32)
Cambodia	61.38 (32.91-97.02)	1.58 (0.9-2.42)	211.62 (134.21-297.5)	1.66 (1.1-2.3)	0.04<br>(0-0.09)
China	9844.02 (6061.65-13643.49)	1.54 (0.99-2.08)	13598.56 (9925.9-18595.65)	0.64 (0.47-0.88)	-2.15<br>(-2.38--1.93)
Democratic People's Republic of Korea	129.51 (84.52-185.52)	1.04 (0.68-1.5)	336.83 (217.05-468.07)	1 (0.64-1.39)	0.06<br>(-0.05-0.17)
Georgia	193.55 (165.77-221.67)	3.66 (3.13-4.19)	220.85 (186.8-260.04)	3.67 (3.11-4.32)	-0.3<br>(-0.59--0.02)

India	1722.3 (1111.5-2207.16)	0.52 (0.34-0.65)	6200.97 (5149.33-8042.5)	0.53 (0.44-0.69)	0.04 (-0.09-0.16)
Indonesia	748 (468.64-1111.01)	0.99 (0.64-1.47)	3287.66 (1955.96-4592.69)	1.3 (0.81-1.79)	0.66 (0.52-0.8)
Iran (Islamic Republic of)	51.92 (30.8-77.14)	0.28 (0.17-0.43)	298.71 (157.55-375.4)	0.39 (0.21-0.49)	0.66 (0.42-0.9)
Iraq	27.18 (16.32-54.19)	0.43 (0.25-0.87)	158.86 (108.43-229.32)	0.69 (0.48-1)	1.03 (0.93-1.13)
Japan	1860.01 (1758.72-1921.79)	1.54 (1.44-1.59)	3453.28 (2836.52-3794.53)	1.04 (0.91-1.11)	-0.55 (-0.84--0.25)
Jordan	6.42 (4.3-10.4)	0.76 (0.5-1.27)	47.84 (32.27-72)	0.68 (0.46-1.02)	-0.44 (-0.73--0.15)
Kazakhstan	292.58 (264.21-326.71)	2.69 (2.41-3.02)	301.91 (255.38-347.28)	1.69 (1.42-1.94)	-1.37 (-1.7--1.04)
Kuwait	2.98 (2.67-3.27)	0.86 (0.76-0.95)	29.92 (25.23-35.02)	0.99 (0.83-1.17)	1.26

					(0.68-1.84)
Kyrgyzstan	57.26 (45.58-71.25)	2.24 (1.79-2.78)	75.25 (57.58-93.85)	1.53 (1.19-1.9)	-0.99 (-1.19--0.79)
Lao People's Democratic Republic	27.98 (13.48-48.44)	1.65 (0.85-2.78)	65.29 (40.35-92.47)	1.35 (0.86-1.86)	-0.51 (-0.57--0.45)
Lebanon	14.47 (9.3-20.5)	0.91 (0.59-1.26)	45.87 (33.58-66.12)	0.75 (0.55-1.08)	-0.58 (-0.66--0.5)
Malaysia	72.81 (50.42-102.11)	1.02 (0.7-1.45)	326.98 (249.68-401.95)	1.17 (0.88-1.44)	0.13 (0.02-0.24)
Maldives	0.38 (0.14-0.66)	0.5 (0.2-0.83)	0.81 (0.58-1.09)	0.25 (0.18-0.33)	-1.92 (-2.09--1.75)
Mauritius	13.77 (12.73-14.95)	2.42 (2.24-2.63)	42.15 (38.07-45.47)	2.27 (2.05-2.44)	-0.9 (-1.31--0.49)
Mongolia	11.46 (7.31-18.55)	1.16 (0.73-1.87)	26.21 (18.08-35.93)	1.13 (0.79-1.53)	-0.16 (-0.33-0)

Myanmar	289.39 (159.79-454.01)	1.5 (0.85-2.31)	680.06 (446.78-925.34)	1.35 (0.92-1.82)	-0.37 (-0.51--0.23)
Nepal	37.49 (21.54-65.8)	0.54 (0.32-0.94)	112.6 (66.72-235.24)	0.5 (0.3-1.03)	-0.51 (-0.75--0.27)
Oman	1.12 (0.72-1.7)	0.23 (0.15-0.36)	3.48 (2.57-4.76)	0.2 (0.15-0.27)	0.08 (-0.03-0.2)
Pakistan	478.04 (351.13-672.17)	1.13 (0.82-1.6)	2382.04 (1681.54-3414.87)	2.03 (1.46-2.93)	1.57 (1.36-1.77)
Palestine	13.67 (9.02-19.41)	1.84 (1.22-2.59)	44.01 (28.84-57.8)	1.87 (1.24-2.45)	0.04 (-0.04-0.11)
Philippines	230.34 (169.39-288.88)	0.98 (0.76-1.27)	1068.38 (822.47-1464.68)	1.25 (0.96-1.76)	0.68 (0.63-0.74)
Qatar	0.52 (0.3-0.91)	1.02 (0.6-1.76)	6.73 (4.74-10.03)	1.04 (0.76-1.48)	0.39 (0.09-0.7)
Republic of Korea	368.46 (187.4-559.77)	1.75 (0.97-2.57)	413.02 (267.26-593.93)	0.45 (0.29-0.64)	-3.9

					(-4.19--3.61)
Saudi Arabia	14.42 (8.95-24.63)	0.37 (0.23-0.64)	93.5 (66.45-128.07)	0.48 (0.34-0.65)	0.92<br>(0.78-1.05)
Seychelles	0.7 (0.53-0.92)	1.44 (1.08-1.9)	1.48 (1.12-2.03)	1.27 (0.97-1.76)	-0.24<br>(-0.33--0.15)
Singapore	21.78 (19.49-24.15)	1.52 (1.36-1.68)	65.23 (56.74-73.52)	0.75 (0.65-0.85)	-1.12<br>(-1.24--0.99)
Sri Lanka	65.21 (48.18-85)	0.82 (0.61-1.07)	202.5 (126.13-299.73)	0.74 (0.47-1.09)	-0.19<br>(-0.3--0.08)
Syrian Arab Republic	18.6 (11.92-32.84)	0.44 (0.28-0.81)	72.83 (51.07-106.71)	0.55 (0.39-0.81)	0<br>(-0.26-0.27)
Taiwan (Province of China)	38.18 (34.55-42.4)	0.38 (0.34-0.42)	297.85 (257.92-339.07)	0.71 (0.62-0.81)	2.12<br>(1.89-2.34)
Tajikistan	38.35 (25.85-56.47)	1.72 (1.16-2.52)	75.78 (44.24-125.79)	1.21 (0.73-1.89)	-0.85<br>(-0.95--0.75)

Thailand	217.85 (147.83-290.87)	0.91 (0.62-1.19)	887.21 (515.47-1207.74)	0.81 (0.47-1.09)	-0.33 (-0.46--0.2)
Timor-Leste	2.53 (1.44-4.02)	1.15 (0.69-1.77)	9.96 (6.44-14.27)	1.13 (0.74-1.63)	-0.05 (-0.17-0.08)
Turkey	337.4 (225.31-499.25)	1.35 (0.9-2)	1070.43 (697.1-1422.33)	1.16 (0.77-1.54)	-0.64 (-0.87--0.41)
Turkmenistan	21.36 (18.56-24.41)	1.32 (1.15-1.51)	34.2 (26.18-44.94)	0.82 (0.63-1.07)	-1.76 (-2.16--1.35)
United Arab Emirates	4.25 (2.58-7.03)	2.03 (1.24-3.41)	49.79 (36.46-69.24)	2.24 (1.62-3.07)	1.33 (0.91-1.75)
Uzbekistan	149.1 (127.71-170.88)	1.56 (1.35-1.79)	274.77 (220.24-328.34)	0.99 (0.8-1.18)	-1.17 (-1.39--0.94)
Viet Nam	216.4 (154.22-292.49)	0.66 (0.47-0.89)	584.34 (414.14-790.71)	0.61 (0.43-0.8)	-0.12 (-0.17--0.08)
Yemen	15.91 (9.29-28.37)	0.46 (0.28-0.85)	65.47 (41.18-111.14)	0.46 (0.29-0.81)	-0.01

					(-0.17-0.14)
**DALYs**					
Cervical cancer					
Afghanistan	15677.26 (7722.92-25460.46)	214.75 (107.93-353.2)	26188.05 (13466.25-39524.89)	163.49 (84.69-245.32)	-1.09 (-1.26--0.92)
Armenia	5521.78 (5187.78-5844.54)	181.74 (170.71-192.59)	3343.27 (2950.4-3829.61)	83.01 (73.28-95.08)	-1.92 (-2.36--1.48)
Azerbaijan	7409.4 (6281.23-8527.81)	130.35 (109.59-150.51)	8350.89 (6275.99-10442.61)	69.87 (53.12-87.43)	-2.2 (-2.38--2.01)
Bahrain	160.71 (124.35-206.18)	63.99 (49.02-81.78)	328.81 (247.11-434.26)	26.24 (20.07-34.1)	-3.52 (-3.86--3.18)
Bangladesh	161978.42 (112553.14-218558.06)	264.51 (184.26-353.85)	197947.59 (137527.34-283274.53)	128.14 (89.33-182.26)	-2.17 (-2.36--1.98)
Bhutan	962.93 (594.03-1363.36)	288.08 (180.17-395.73)	939.9 (611.54-1300.39)	135.63 (88.94-185.51)	-2.73 (-2.99--2.47)

Brunei Darussalam	341.68 (265.62-418.26)	212.69 (167.62-260.29)	579.06 (446.81-734.29)	123.66 (96.97-154.07)	-1.4 (-1.67--1.13)
Cambodia	17560.25 (13739.13-22775.07)	295.07 (230.76-384.5)	25632.73 (18684.33-35827.27)	172.3 (126.73-240.68)	-1.92 (-2.01--1.83)
China	1116204.22 (894262.88-1381045.75)	112.27 (90.48-138.52)	1547786.46 (1119815.01-2009230.03)	75.05 (54.64-97.84)	-1 (-1.15--0.84)
Democratic People's Republic of Korea	28693.46 (20876.41-40391.15)	148.76 (109.42-208.86)	47466.44 (32248.65-68224.6)	137.91 (94.09-197.23)	-0.08 (-0.2-0.05)
Georgia	10844.84 (9618.88-12053.24)	178.23 (158.14-198.13)	6832.52 (5861.6-7859.26)	132.94 (113.73-153.3)	-0.44 (-1-0.12)
India	1714703.96 (1426545.18-1998969.65)	281.39 (233.34-328.69)	2056394.96 (1757966.05-2378274.53)	152.25 (130.46-175.71)	-2.1 (-2.42--1.77)
Indonesia	224296.08 (170255.54-283271.99)	169.54 (128.78-213.3)	368081.65 (265500.15-488921.36)	123.46 (89.37-162.9)	-1.09 (-1.24--0.93)
Iran (Islamic Republic of)	12829.43 (11037.94-15810.2)	39.16 (33.64-47.93)	17794.92 (15483.7-20150.51)	19.81 (17.2-22.4)	-2.14

					(-2.25--2.03)
Iraq	3956.35 (2977.2-5306.63)	40.26 (30.19-53.62)	8919.23 (6304.23-12183.09)	28.6 (20.48-38.53)	-1.23 (-1.33--1.13)
Japan	94569.17 (90104.88-97771.28)	57.05 (54.32-58.98)	100328.1 (91098-106112.2)	49.09 (46.44-51.09)	-0.23 (-0.32--0.13)
Jordan	1104.56 (826.19-1494.34)	58.13 (44-77.89)	1980.09 (1427.24-2704.72)	20.14 (14.77-26.81)	-3.95 (-4.45--3.45)
Kazakhstan	29052.85 (26814.16-31422.46)	210.12 (193.44-228.12)	22997.51 (19551.5-26691.68)	114.31 (97.3-132.81)	-1.32 (-1.83--0.81)
Kuwait	543.12 (491.46-596.42)	50.35 (45.82-54.55)	558.64 (468.98-672.75)	11.52 (9.73-13.59)	-3.84 (-4.43--3.24)
Kyrgyzstan	7714.17 (6720.3-8901.6)	241.39 (211.13-276.82)	8750.45 (6931.23-10774.45)	148.63 (119.44-181.49)	-1.27 (-1.49--1.05)
Lao People's Democratic Republic	8206.28 (5821.88-11167.39)	315.83 (227.83-428.65)	9052.12 (6411.25-12102.61)	148.92 (106.85-198.55)	-2.67 (-2.75--2.58)

Lebanon	1789.95 (1359-2307.97)	72.74 (55.32-93.39)	1611.79 (1237.15-2034.65)	27.17 (20.86-34.45)	-3.41<br>(-3.5--3.32)
Malaysia	22656.39 (18722.04-25927.42)	200.74 (166.31-229.87)	43452.09 (33265.46-50556.02)	139.26 (106.68-162.97)	-1.36<br>(-1.73--0.99)
Maldives	218.99 (123.46-305.01)	177.43 (102.72-246.92)	149.38 (111.63-188.94)	32.73 (24.77-41.85)	-5.73<br>(-5.87--5.59)
Mauritius	1358.48 (1272.1-1456.71)	158.27 (147.88-169.55)	1700.47 (1520.23-1812.57)	97.24 (86.87-103.55)	-2.57<br>(-3.08--2.06)
Mongolia	2907.07 (2086.51-3981.24)	235.88 (170.45-324.2)	4892.64 (3638.61-6474.8)	165.74 (122.27-218.58)	-1.54<br>(-1.77--1.32)
Myanmar	83049.31 (59514.85-110591.1)	282.43 (205.96-377.2)	72566.51 (51864.94-100904.99)	129.96 (92.93-181.09)	-2.93<br>(-3.14--2.72)
Nepal	35888.49 (24699.36-50029.11)	290.29 (203.31-404.69)	43121.58 (29087.96-57864.67)	162.12 (110.13-216.48)	-1.87<br>(-2.34--1.39)
Oman	522.63 (360.82-790.76)	52.21 (36.79-78.28)	618.37 (455.14-814.13)	19.49 (14.88-24.71)	-2.96

					(-3.11--2.81)
Pakistan	53638.26 (42774.64-64282.1)	77.2 (62.09-91.7)	133656.99 (93319.37-182373.4)	76.56 (54.25-103.71)	-0.49<br>(-0.74--0.23)
Palestine	276.2 (194.23-378.49)	27.84 (19.85-38.41)	442.12 (343.58-555.55)	13.92 (11.01-17.17)	-2.23<br>(-2.36--2.09)
Philippines	48942.71 (41955.8-57913.94)	118.06 (101.94-142.39)	116068.74 (88511.23-148404.63)	114.86 (87.99-146.1)	0.11<br>(0.04-0.17)
Qatar	79.47 (60.67-107.13)	38.52 (30.27-49.3)	261.33 (188.45-374.03)	17.54 (13.23-23.38)	-2.77<br>(-3.44--2.1)
Republic of Korea	46711.2 (39191.28-56093.34)	121.9 (102.12-146.83)	29250.02 (23031.67-38351.01)	36.02 (28.81-46.49)	-4.26<br>(-4.42--4.1)
Saudi Arabia	1915.97 (1379.76-2577.19)	21.06 (15.45-27.9)	4417.79 (3162.9-6122.33)	11.75 (8.6-15.88)	-1.93<br>(-2.21--1.64)
Seychelles	213.54 (182.9-246.9)	373.02 (320.15-431.57)	256.37 (212.39-301.75)	203.45 (169.04-239.14)	-1.46<br>(-1.59--1.33)

Singapore	2959.68 (2715.69-3216.25)	106.93 (98.77-115.93)	2651.03 (2383.56-2930.41)	31.2 (28.05-34.49)	-4.1 (-4.41--3.79)
Sri Lanka	12366.91 (10500.41-14355.59)	92.14 (78.52-107.81)	15391.45 (9606.42-21816.92)	56.91 (35.74-80.24)	-1.34 (-1.48--1.19)
Syrian Arab Republic	2471.06 (1852.93-3277.66)	36.07 (27.3-47.88)	3348.59 (2273.08-4645.97)	22.47 (15.28-30.95)	-1.89 (-2.21--1.58)
Taiwan (Province of China)	33816.33 (31419-36239.36)	189.13 (175.38-203.05)	20987.34 (18496.95-23451.7)	55.84 (49.23-62.2)	-4.88 (-5.27--4.48)
Tajikistan	3765.31 (2896.19-4629.23)	120.24 (92.75-148.21)	4714.19 (3135.91-6887.37)	61.29 (41.96-87.46)	-2.31 (-2.46--2.16)
Thailand	113647.29 (92976.17-136349.96)	255.19 (208.25-305.78)	139974.53 (105654.04-182887.21)	141.54 (106.9-184.48)	-2.47 (-2.74--2.21)
Timor-Leste	787.21 (549.42-1164.74)	181.02 (129.72-264.05)	1254.94 (908.73-1740.5)	131.24 (95-181.59)	-0.98 (-1.27--0.68)
Turkey	28032.81 (22072.1-36720.92)	66.05 (52.15-86.26)	27373.82 (20739.93-35243.82)	28.2 (21.47-36.12)	-3.04

					(-3.27--2.8)
Turkmenistan	3727.92 (3404.21-4049.99)	164.03 (150.46-177.65)	6582.36 (4859.18-8832.93)	134.5 (99.61-179.96)	0.21<br>(-0.12-0.54)
United Arab Emirates	884.13 (632.47-1281.63)	129.94 (96.21-182.56)	2825.36 (2115.57-3827.29)	56.02 (41.55-73.59)	-1.41<br>(-1.84--0.99)
Uzbekistan	18798.64 (17260.73-20384.2)	144.9 (133-157.27)	41821.41 (34414.72-50387.69)	127.08 (104.85-152.99)	-0.49<br>(-0.7--0.27)
Viet Nam	78920.02 (60069.63-104251.38)	176.49 (134.9-232.31)	136620.76 (102668.25-181500.95)	124.37 (93.92-163.59)	-1.17<br>(-1.26--1.07)
Yemen	4684.58 (2962.2-8012.03)	72.86 (47.86-121.95)	10191.19 (6487.32-14741)	52.01 (34.22-74.63)	-1.32<br>(-1.42--1.22)
Ovarian cancer					
Afghanistan	2200.92 (899.01-5416.44)	29.32 (11.73-70.77)	6375.92 (3302.58-15041.53)	46.59 (22.47-100.96)	1.68<br>(1.53-1.83)
Armenia	1921.07 (1567.55-2468.95)	65.1 (53.57-83.12)	3329.15 (2728.53-3958.75)	78.77 (64.54-93.75)	0.85

					(0.65-1.06)
Azerbaijan	2197.86 (1450.25-3140.17)	39.33 (26.19-56.37)	5568.92 (3546.75-8281.35)	46.68 (29.95-69.28)	0.96<br>(0.61-1.31)
Bahrain	190.54 (142.95-249.94)	81.64 (61.41-106.35)	916.72 (601.5-1212.25)	78.62 (52.57-101.79)	-0.44<br>(-0.57--0.32)
Bangladesh	18666.13 (12085.01-27993.52)	32.97 (20.8-49)	73861.79 (44583.61-129952.29)	48.74 (29.79-85.57)	1.2<br>(1.13-1.28)
Bhutan	113.01 (70.46-180.99)	36.79 (22.77-58.9)	360.54 (231.58-646.52)	54.25 (35.31-96.88)	1.17<br>(1.12-1.23)
Brunei Darussalam	144.06 (105.85-202.75)	98.62 (75.12-133.7)	461.17 (354.65-583.62)	102.84 (79.23-127.29)	0.42<br>(0.35-0.5)
Cambodia	2765.93 (1638.38-5194.7)	48.48 (29.42-87.9)	14437.07 (8702.29-21982.45)	98.99 (60.67-148.75)	2.33<br>(2.28-2.38)
China	402037.07 (288619.19-527088.85)	40.95 (30.31-53.22)	750548.81 (545756.13-991109.83)	35.69 (26-47.31)	-0.93<br>(-1.1--0.76)

Democratic People's Republic of Korea	7840.48 (4935.26-11709.39)	41.22 (26.2-60.31)	16317.08 (10862.25-23341.61)	47.54 (31.64-67.76)	0.55 (0.5-0.6)
Georgia	2225.92 (1758.38-2791.5)	35.04 (27.72-43.91)	8102.27 (6810.32-9385.94)	148.69 (124.56-172.53)	5.84 (5.44-6.24)
India	201484.47 (145601.23-268250.32)	35.42 (25.67-46.87)	712412.71 (600595.93-847441.67)	54.51 (46.09-64.85)	1.29 (1.19-1.39)
Indonesia	60954.96 (42515.96-114826.65)	47.55 (33.73-88.12)	186917.18 (121865.63-309820.43)	64 (42.23-106.76)	0.81 (0.69-0.92)
Iran (Islamic Republic of)	6688.26 (4897.4-9199.83)	20.45 (15.26-27.93)	28986.3 (19521.58-33613.58)	32.98 (22.36-38.24)	1.93 (1.76-2.11)
Iraq	3400.02 (2431.91-5135.91)	36.06 (25.67-54.07)	14884.62 (10127.62-21189.83)	49.46 (34.6-68.12)	1.11 (1.02-1.2)
Japan	111747.58 (107725.39-114971.21)	67.2 (64.82-69.12)	126933.74 (113293.04-135381.81)	54.02 (50.59-56.29)	-0.7 (-0.79--0.61)
Jordan	624.06 (401.1-858.68)	36.18 (22.97-49.72)	3828.24 (2387.96-5155)	41.4 (25.96-54.71)	0.29

					(-0.02-0.6)
Kazakhstan	11372.12 (9534.56-13779.18)	82.22 (68.89-99.52)	17083.04 (13863.42-20684.47)	86.48 (70.37-104.47)	-0.11 (-0.41-0.18)
Kuwait	315 (285.97-346.53)	39.08 (35.49-42.93)	1374.61 (1149.52-1660.5)	31.05 (26.1-36.99)	-0.78 (-1.11--0.46)
Kyrgyzstan	1682.4 (1397.03-2020.37)	53.8 (44.75-65.07)	5479.41 (4356.45-6804.61)	95.72 (75.62-118.01)	3.22 (2.54-3.89)
Lao People's Democratic Republic	1128.45 (618.28-2644.17)	45.25 (25.06-102.02)	4531.72 (2768.21-7269.28)	77.11 (47.87-123.16)	1.89 (1.8-1.97)
Lebanon	1323.04 (959.25-2049.47)	55.92 (40.51-86.06)	3877.08 (2595.18-5075.12)	65.98 (44.48-86.37)	0.7 (0.53-0.88)
Malaysia	4693.78 (3726.3-7264.48)	42.84 (34.31-63.91)	16978.99 (13530.47-24258.03)	54.81 (43.72-78.1)	1.1 (0.77-1.44)
Maldives	53.47 (29.95-150.61)	44.61 (25.51-119.62)	219.6 (154-308.71)	49.1 (35.56-64.05)	0.07 (-0.3-0.43)

Mauritius	516.57 (480.87-560.85)	60.01 (55.87-65.19)	1670.29 (1502.79-1794.16)	94.06 (84.48-100.7)	1.2 (0.78-1.62)
Mongolia	746.78 (514.95-1051.29)	60.1 (41.8-84.49)	2139.09 (1528.75-2825.24)	74.37 (52.73-97.64)	0.64 (0.51-0.77)
Myanmar	16475.63 (10555.42-29990.87)	58.43 (38.45-101.64)	42588.68 (29762.87-61023.87)	77.21 (54.23-110.61)	0.75 (0.67-0.83)
Nepal	3990.09 (2251.07-6283)	35.22 (20-55.29)	14576.24 (9682.37-25353.99)	56.22 (37.47-97.06)	1.47 (1.38-1.56)
Oman	138.47 (59.09-219.97)	17.08 (7.38-27.52)	503.42 (339.47-670.52)	19.56 (13.53-26.06)	0.51 (0.16-0.86)
Pakistan	40132.42 (27151.75-49740.75)	61.5 (41.72-76.25)	158996.54 (94939.06-236599.19)	101.62 (61.96-149.9)	1.42 (1.26-1.58)
Palestine	383.18 (246.45-571.25)	38.93 (24.94-58.03)	1541.55 (1107.32-1930.39)	49.63 (35.53-62.01)	0.93 (0.86-1.01)
Philippines	24743.75 (18258.84-29205.75)	62.56 (45.38-73.89)	88182.27 (65452.36-112671.49)	89.33 (67.94-113.85)	1.25

					(1.22-1.28)
Qatar	89.07 (63.92-120.71)	49.28 (35.02-66.2)	708.93 (439.08-1015.43)	53.18 (35.25-70.75)	0.27 (0.03-0.51)
Republic of Korea	14519.95 (12784.76-18269.31)	38.22 (33.96-50.9)	36575.05 (24150.12-43029.25)	42.45 (28.93-49.61)	0.15 (0.02-0.27)
Saudi Arabia	1905.89 (1268.86-3131.45)	25.44 (16.82-40.3)	10568.12 (7192.48-16621.48)	34.53 (23.97-53.31)	0.85 (0.58-1.12)
Seychelles	51.65 (42.53-68.28)	88.95 (73.04-117.72)	137.48 (103.43-167)	109.72 (83.41-132.75)	0.97 (0.76-1.18)
Singapore	2150.8 (2012.68-2289.01)	80.34 (74.98-85.65)	4146.99 (3770.68-4506.43)	48.9 (44.7-52.97)	-1.59 (-1.77--1.41)
Sri Lanka	4823.77 (3949.67-6133.93)	36.45 (29.96-46.25)	13732.8 (8005.03-19384.6)	50.58 (29.78-71.45)	1.4 (1.22-1.58)
Syrian Arab Republic	1435.34 (998.99-2009.31)	21.99 (15.36-30.81)	4288.83 (2867.09-6153.88)	28.79 (19.35-41.1)	0.68 (0.54-0.82)

Taiwan (Province of China)	6687.8 (6140.19-7189.06)	37.01 (33.87-39.78)	20230 (17577.52-22574.7)	53.18 (46.39-59.22)	0.95 (0.72-1.18)
Tajikistan	821.22 (587.06-1202.45)	26.7 (19.12-37.97)	2083.02 (1463.81-2785.41)	27.06 (19.22-35.86)	-0.06 (-0.13-0)
Thailand	22656.58 (16080.23-28350.29)	52.25 (36.95-65.19)	70753.74 (43213.62-97573.35)	69.88 (41.57-95.85)	0.76 (0.64-0.88)
Timor-Leste	127.1 (69.16-227.8)	31.56 (17.31-55.15)	544.87 (346.81-758.09)	56.17 (36.36-78.26)	2.08 (1.87-2.29)
Turkey	28405.06 (19379.82-44572.91)	70.64 (48.83-108.33)	65293.32 (45289.75-84022.43)	67.32 (46.71-86.61)	-0.22 (-0.35--0.09)
Turkmenistan	871.94 (776.27-967.02)	38.55 (34.28-42.75)	3300.62 (2500.76-4460.72)	68.39 (52.21-92.06)	3.4 (1.29-5.56)
United Arab Emirates	572 (365.01-1010.54)	89.36 (58.4-159.71)	4458.29 (3310.3-6511.5)	97.66 (75.81-126.56)	1.72 (1.25-2.19)
Uzbekistan	3918.74 (3026.05-4893.11)	30.53 (23.54-38.2)	14795.59 (11367.8-18804.3)	46.16 (35.59-58.42)	1.11

					(0.79-1.42)
Viet Nam	11509.35 (8195.21-14965.6)	26.89 (19.01-35.25)	43403.36 (27759.26-60076.08)	39.98 (25.72-54.37)	1.28 (1.21-1.35)
Yemen	844.77 (123.69-1834.24)	14.57 (2.02-32.15)	4489.83 (2172.35-7424.15)	25 (11.83-40.89)	2 (1.83-2.17)
Uterine cancer					
Afghanistan	2058.59 (937.11-4259.98)	27.6 (12.88-56.8)	4671.19 (2174.91-8159.97)	39.02 (19.08-67.34)	1.29 (1.2-1.39)
Armenia	1873.94 (1748.64-1998.87)	64.17 (59.69-68.54)	2597.57 (2180.31-3118.67)	59.76 (50.23-71.75)	-0.07 (-0.62-0.48)
Azerbaijan	2649.72 (2013.71-3214.29)	49.54 (37.73-59.76)	4003.51 (2945.03-5730.04)	34.82 (25.76-48.86)	-1.24 (-1.36--1.11)
Bahrain	54.42 (40.32-75.21)	27.81 (20.62-39.07)	261.39 (174.85-387.87)	26.04 (17.83-37.24)	-0.72 (-1.01--0.44)
Bangladesh	6563.98 (4374.84-11014.39)	12.77 (8.6-21.67)	14869.31 (8616.92-33837.62)	10.28 (6.01-23.19)	-0.69

					(-0.82--0.56)
Bhutan	39.06 (22.48-68.9)	14.62 (8.41-26.32)	81.07 (45.64-184.02)	12.97 (7.35-29.43)	-0.56 (-0.7--0.42)
Brunei Darussalam	50.06 (34.15-75.27)	41.05 (28.02-61.59)	163.34 (118.43-219.13)	39.74 (29.07-53.03)	0.36 (0.18-0.55)
Cambodia	2455.86 (1347.87-3597.2)	46.97 (27.35-67.64)	6461.25 (3906.95-9442.28)	46.35 (28.88-66.59)	-0.17 (-0.23--0.1)
China	345367.13 (227925.01-438108.4)	36.29 (24.69-45.53)	405490.48 (301127.08-553177.73)	19.11 (14.2-26.08)	-2.44 (-2.77--2.12)
Democratic People's Republic of Korea	5869.38 (3669.85-8269.81)	31.87 (20.15-44.24)	10543.68 (6475.33-15396.03)	30.51 (19.01-43.82)	-0.06 (-0.18-0.07)
Georgia	6907.12 (6284.86-7616.14)	108.65 (98.83-119.44)	5572.57 (4721.48-6589.02)	96.8 (82.49-113.62)	-0.39 (-0.83-0.06)
India	64724.67 (45581.27-79293.4)	12.63 (8.97-15.52)	168761.46 (139685.18-220234.71)	13.51 (11.19-17.63)	0.06 (-0.15-0.28)

Indonesia	40206.67 (24740.33-52838.37)	34.44 (22.19-45.15)	105091.07 (60992.49-148764.57)	37.35 (22.09-51.95)	0.16 (0.04-0.28)
Iran (Islamic Republic of)	2649.55 (1710.13-3316.84)	9.04 (5.93-11.3)	9135.92 (4599.85-11677.21)	10.9 (5.61-13.91)	0.94 (0.54-1.33)
Iraq	1273.63 (780.41-2321.03)	14.86 (9.16-26.85)	5027.53 (3379.79-7300.25)	18.64 (12.82-27.08)	0.87 (0.71-1.02)
Japan	40544.22 (38121.9-42254.88)	23.8 (22.35-24.83)	75779.53 (66604.32-81241.79)	29.79 (27.3-31.45)	1.02 (0.88-1.17)
Jordan	348.06 (245.07-496.49)	23.35 (16.7-32.38)	1473.97 (961.47-2238.57)	17.84 (11.82-26.63)	-1.05 (-1.42--0.67)
Kazakhstan	9478.98 (8283.82-10784.21)	71.81 (62.72-81.66)	8861.22 (7563.85-10195.53)	45.91 (39.27-52.77)	-1.64 (-2.18--1.1)
Kuwait	95.56 (84.89-107.43)	14.29 (12.58-16.3)	1100.53 (922.19-1304.53)	28.51 (23.71-33.84)	3.01 (2.45-3.58)
Kyrgyzstan	1817.59 (1511.05-2187.23)	58.3 (48.12-70.39)	2361.79 (1803.95-2968.54)	43.34 (33-54.4)	-0.97

					(-1.27--0.66)
Lao People's Democratic Republic	1095.73 (527.38-1740.78)	46.45 (23.35-73)	2098.92 (1257.72-3034.14)	38.61 (23.56-55.07)	-0.75 (-0.83--0.67)
Lebanon	557.91 (375.03-796.09)	24.64 (16.85-34.69)	1130.36 (828.41-1625.31)	19.2 (14.05-27.62)	-0.76 (-0.84--0.68)
Malaysia	3061.77 (2384.16-3821.04)	30.95 (23.61-38.45)	9263.24 (7108.58-11404.1)	30.87 (23.62-37.78)	-0.03 (-0.14-0.08)
Maldives	15.69 (7.44-24.76)	14.06 (7.01-21.77)	25.25 (18.04-34.98)	6.53 (4.72-8.75)	-2.82 (-2.97--2.68)
Mauritius	617.22 (571.01-669.27)	77.59 (71.69-83.93)	1194.55 (1080.8-1291.8)	63.98 (57.79-69.18)	-1.37 (-2.05--0.7)
Mongolia	416.05 (280.44-631.11)	36.07 (24.61-54.41)	840.37 (565.84-1179.46)	31.33 (21.6-43.07)	-0.77 (-0.95--0.6)
Myanmar	12447.92 (6841.63-19439.23)	46.7 (26.51-72.64)	20927.56 (13050.64-29120.67)	38.84 (24.78-53.42)	-0.9 (-1.06--0.75)

Nepal	1366.72 (789.61-2503.69)	13.24 (7.85-24.28)	3095.36 (1821.66-6478.5)	12.6 (7.49-26.23)	-0.17 (-0.55-0.21)
Oman	39.43 (26.93-57.8)	5.53 (3.84-7.89)	108.02 (76.57-157.38)	5.14 (3.77-6.96)	0.12 (-0.09-0.34)
Pakistan	21601.62 (17227.73-28105.7)	36.1 (28.77-47.77)	70035.22 (48443.36-101624.21)	51.62 (36.1-73.77)	0.87 (0.66-1.08)
Palestine	478.85 (316.12-688.31)	52.15 (34.73-75.06)	1322.49 (855.61-1791.16)	47.5 (31.1-62.77)	-0.18 (-0.27--0.08)
Philippines	10722.55 (7478.53-13197.66)	30 (21.57-37.69)	34076.81 (25742.64-44735.87)	36.32 (27.71-48.83)	0.69 (0.61-0.77)
Qatar	30.85 (20.18-48.21)	23.55 (15.54-36.68)	236.28 (160.4-362.13)	24.1 (17.28-34.87)	0.27 (-0.15-0.69)
Republic of Korea	11997.2 (6593.76-16643.59)	35.66 (20.63-49.26)	10703.18 (6621.99-15358.77)	11.87 (7.37-16.86)	-3.31 (-3.82--2.8)
Saudi Arabia	668.3 (452.95-991.75)	10.06 (6.95-15.04)	3492.92 (2401.05-4998.92)	13.3 (9.41-18.43)	0.98

					(0.79-1.18)
Seychelles	24.42 (18.82-31.84)	43.21 (33.22-56.5)	43.26 (32.92-58.93)	34.56 (26.51-47.2)	-0.36 (-0.47--0.25)
Singapore	743.75 (676.1-817.36)	30.48 (27.62-33.69)	1716.29 (1502.29-1942.2)	19.46 (17.11-21.97)	-1.09 (-1.24--0.95)
Sri Lanka	2620.39 (1954.37-3482.58)	21.85 (16.32-28.55)	5440.92 (3290.95-8090.27)	19.51 (11.9-29.06)	-0.48 (-0.65--0.3)
Syrian Arab Republic	989.19 (730.59-1346.27)	16.56 (12.26-22.58)	2274.19 (1599.59-3316.76)	15.61 (10.97-22.72)	-0.63 (-0.92--0.34)
Taiwan (Province of China)	1802.68 (1629.45-1986.3)	10.33 (9.31-11.41)	9107.79 (7915.26-10338.07)	22.52 (19.66-25.44)	2.7 (2.38-3.03)
Tajikistan	1416.21 (996.48-1919.48)	47.65 (33.44-64.55)	2516.93 (1402.7-4357.89)	35.38 (20.62-59.01)	-1.05 (-1.17--0.94)
Thailand	9148.62 (6699.5-11709.14)	22.99 (16.78-29.27)	25287.84 (14857.16-34677.9)	23.45 (13.72-32.18)	-0.37 (-0.57--0.18)

Timor-Leste	107.53 (61.97-162.42)	30.77 (18.71-45.03)	294.14 (180.77-428.37)	32 (19.86-46.66)	0.2<br>(0.02-0.39)
Turkey	14294.42 (9662.06-18732.96)	38.1 (25.92-49.66)	28867.41 (18370.83-38772.41)	29.97 (19.12-40.21)	-0.74<br>(-1.09--0.39)
Turkmenistan	747.75 (679.39-815.47)	35.57 (32.28-38.9)	1096.76 (830.88-1470.07)	23.97 (18.25-31.89)	-1.36<br>(-2.02--0.69)
United Arab Emirates	275.26 (160.53-412.25)	57.38 (33.23-85.38)	1657.65 (1197.09-2382.43)	49.23 (36.13-67.21)	1.2<br>(0.66-1.75)
Uzbekistan	4565.19 (3789.83-5311.37)	37.76 (31.61-43.61)	9090.22 (7196.06-11020.82)	29.48 (23.58-35.43)	-0.43<br>(-0.67--0.19)
Viet Nam	6442.41 (4851.98-8727.26)	15.59 (11.71-21.2)	16160.96 (11291.09-22707.79)	15.38 (10.91-21.6)	0.13<br>(0.07-0.19)
Yemen	574.46 (334.79-1014.72)	10.66 (6.39-18.87)	2069.23 (1297.89-3490.83)	12.66 (7.86-21.45)	0.65<br>(0.51-0.79)
**Incidence**					

Cervical cancer					
Afghanistan	580.12 (293.04-963.48)	8.09 (4.16-13.3)	1102.45 (571.73-1653.29)	7.04 (3.72-10.6)	-0.63 (-0.74--0.52)
Armenia	343.61 (320.73-367.61)	11.23 (10.49-12)	243.72 (214.07-279.5)	6.16 (5.39-7.08)	-1.21 (-1.68--0.73)
Azerbaijan	407.01 (345.74-467.19)	7.13 (6.05-8.21)	549.3 (413.7-697.3)	4.61 (3.5-5.81)	-1.49 (-1.65--1.33)
Bahrain	8.09 (6.33-10.36)	3.24 (2.51-4.14)	23.39 (17.87-30.7)	1.81 (1.39-2.34)	-2.44 (-2.78--2.11)
Bangladesh	6746.84 (4696.22-9094.71)	11.08 (7.75-14.86)	11294.43 (7719.63-16347.82)	7.28 (4.98-10.46)	-1.19 (-1.4--0.98)
Bhutan	40.16 (24.97-56.63)	12.24 (7.69-17.06)	52.85 (34.25-75.05)	7.52 (4.93-10.43)	-1.84 (-2.12--1.57)
Brunei Darussalam	20.9 (16.35-25.69)	12.65 (9.92-15.43)	41.2 (31.93-52.09)	8.77 (6.92-10.92)	-0.83 (-1.07--0.6)

Cambodia	742.61 (582.39-971.21)	12.71 (9.94-16.66)	1389.08 (1013.08-1934.72)	9.36 (6.91-13.13)	-1.14 (-1.24--1.04)
China	57843.04 (46321.43-71401.72)	5.84 (4.71-7.19)	132787.82 (95959.18-172599.73)	6.67 (4.8-8.72)	0.92 (0.73-1.11)
Democratic People's Republic of Korea	1536.62 (1101.64-2133.87)	8.07 (5.82-11.22)	3034.83 (2072.17-4285.8)	8.93 (6.11-12.61)	0.53 (0.41-0.65)
Georgia	690.01 (608.26-768.43)	11.51 (10.16-12.86)	454.68 (389.97-522.43)	9.11 (7.79-10.51)	-0.38 (-0.91-0.16)
India	72929.93 (60449.01-84760.01)	12.13 (10.02-14.16)	112102.51 (95757.45-129057.05)	8.29 (7.1-9.53)	-1.34 (-1.69--0.99)
Indonesia	10417.32 (7931.19-13190.92)	7.95 (6.06-10.01)	20549.61 (14948.41-27288.43)	6.97 (5.09-9.16)	-0.54 (-0.67--0.42)
Iran (Islamic Republic of)	595.97 (511.17-741.74)	1.87 (1.6-2.3)	1123.1 (969.37-1284.77)	1.24 (1.07-1.42)	-1.32 (-1.45--1.19)
Iraq	184.71 (139.32-248.36)	1.87 (1.41-2.47)	542.64 (388.33-751.97)	1.72 (1.23-2.34)	-0.3

					(-0.38--0.21)
Japan	9314.69 (8830.76-9704.99)	5.73 (5.43-5.97)	11820.39 (10737.16-12614.19)	6.2 (5.8-6.57)	0.65 (0.51-0.8)
Jordan	55.45 (41.29-74.67)	2.92 (2.23-3.87)	136.52 (98.57-186.36)	1.36 (0.99-1.81)	-2.87 (-3.35--2.39)
Kazakhstan	1688.46 (1550.85-1828.87)	12.13 (11.15-13.14)	1750.56 (1479.12-2025.75)	8.67 (7.34-10.05)	-0.21 (-0.63-0.21)
Kuwait	36.85 (33.14-41.12)	3.14 (2.88-3.44)	48.64 (40.69-58.55)	0.93 (0.78-1.1)	-3.06 (-3.63--2.48)
Kyrgyzstan	431.06 (373.79-500.21)	13.36 (11.7-15.45)	595.39 (463.34-748.17)	9.96 (7.87-12.31)	-0.56 (-0.85--0.28)
Lao People's Democratic Republic	331.33 (234.59-451.66)	12.98 (9.29-17.7)	460 (327.74-599.02)	7.6 (5.49-9.8)	-1.95 (-2.03--1.87)
Lebanon	93.51 (70.89-120.58)	3.83 (2.91-4.91)	119.88 (91.55-152.41)	1.98 (1.52-2.52)	-2.32 (-2.4--2.23)

Malaysia	1216.52 (1003.83-1399.89)	10.75 (8.9-12.38)	2994.93 (2322.06-3526.66)	9.54 (7.4-11.2)	-0.54 (-0.89--0.19)
Maldives	10.51 (5.86-14.54)	8.59 (5.02-11.85)	11.64 (8.85-14.6)	2.42 (1.85-3.06)	-4.33 (-4.44--4.22)
Mauritius	80.5 (74.59-87.08)	9.3 (8.64-10.05)	117.87 (105.61-126.49)	6.89 (6.16-7.38)	-2.06 (-2.55--1.57)
Mongolia	136.08 (98.18-187.56)	11.01 (7.95-15.11)	297.52 (221.91-393.53)	9.98 (7.4-13.39)	-0.56 (-0.73--0.38)
Myanmar	3539.74 (2511.9-4728.52)	12.2 (8.84-16.24)	3934.26 (2799.89-5410.71)	7.1 (5.06-9.74)	-2.16 (-2.33--1.98)
Nepal	1472.81 (1023.71-2062.72)	12.11 (8.48-16.81)	2297.87 (1536.36-3069.51)	8.63 (5.88-11.43)	-1.09 (-1.56--0.63)
Oman	26.71 (18.28-40.7)	2.61 (1.82-3.86)	46.85 (34.31-62.08)	1.33 (1.01-1.68)	-2.01 (-2.14--1.87)
Pakistan	2317.1 (1868.08-2768.69)	3.37 (2.72-3.98)	6734.34 (4725.45-9318.53)	3.83 (2.73-5.21)	-0.02

					(-0.23-0.19)
Palestine	13.44 (9.68-18.45)	1.36 (0.98-1.87)	27.42 (21.47-34.14)	0.85 (0.67-1.05)	-1.51 (-1.62--1.41)
Philippines	2553.39 (2191.76-3050.57)	6.15 (5.3-7.49)	6636.04 (4985.4-8521.51)	6.57 (5-8.39)	0.29 (0.25-0.34)
Qatar	4.45 (3.37-6.27)	2.1 (1.65-2.75)	22.82 (16.47-31.85)	1.3 (0.98-1.72)	-1.72 (-2.35--1.09)
Republic of Korea	3151.37 (2641.02-3764.94)	8.16 (6.93-9.78)	3420.89 (2693.3-4462.01)	4.48 (3.61-5.78)	-2.15 (-2.29--2.01)
Saudi Arabia	150.72 (108.15-203.24)	1.5 (1.1-1.98)	605.3 (430.25-854.8)	1.39 (1.02-1.91)	-0.14 (-0.38-0.1)
Seychelles	11.9 (10.04-13.69)	20.62 (17.42-23.82)	17.27 (14.31-20.48)	13.87 (11.53-16.42)	-0.84 (-0.95--0.73)
Singapore	233.43 (211.7-255.29)	8.23 (7.52-8.98)	295.36 (265.88-326.27)	3.54 (3.19-3.91)	-2.77 (-3.04--2.51)

Sri Lanka	685.9 (581.02-802.4)	5.1 (4.3-5.93)	1142.45 (717-1608.76)	4.29 (2.71-5.97)	-0.33 (-0.47--0.19)
Syrian Arab Republic	118.02 (89.17-155.36)	1.73 (1.31-2.27)	210.19 (143.27-292.72)	1.46 (1-2.02)	-0.9 (-1.17--0.63)
Taiwan (Province of China)	2529.47 (2355.23-2713.26)	13.97 (12.98-15.01)	2041.14 (1795.91-2291.87)	5.64 (4.94-6.29)	-3.96 (-4.37--3.55)
Tajikistan	197.86 (153.78-243.33)	6.25 (4.84-7.63)	272.2 (182.16-403.55)	3.49 (2.39-5.01)	-2.06 (-2.25--1.86)
Thailand	6530.11 (5248.42-7784.17)	14.63 (11.8-17.43)	10962.99 (8328.79-14225.04)	11.53 (8.71-15.04)	-1.35 (-1.62--1.08)
Timor-Leste	33.77 (23.72-50.17)	7.99 (5.72-11.52)	64.82 (46.7-87.86)	6.79 (4.93-9.15)	-0.41 (-0.71--0.1)
Turkey	1304.67 (1024.42-1703.37)	3.1 (2.44-4.04)	1839.97 (1407.71-2365.45)	1.91 (1.47-2.45)	-1.72 (-1.94--1.51)
Turkmenistan	203.91 (185.77-221.86)	8.82 (8.07-9.58)	413.92 (306.11-546.63)	8.41 (6.23-11.07)	0.72

					(0.37-1.07)
United Arab Emirates	42.77 (30.79-61.98)	6.29 (4.61-8.85)	179.77 (133.62-238.82)	3.43 (2.57-4.44)	-0.61<br>(-1.05--0.17)
Uzbekistan	1070.85 (979.32-1159.65)	8.08 (7.33-8.77)	2601.54 (2101.29-3108.86)	7.84 (6.36-9.37)	-0.06<br>(-0.29-0.17)
Viet Nam	4167.11 (3173.09-5426.1)	9.29 (7.1-12.17)	9655.2 (7258.6-12830.44)	8.79 (6.69-11.54)	-0.24<br>(-0.34--0.15)
Yemen	192.53 (121.89-335.21)	3.07 (2.04-5.29)	488.59 (310.04-726.32)	2.49 (1.63-3.63)	-0.85<br>(-0.93--0.76)
Ovarian cancer					
Afghanistan	99.6 (39.29-242.3)	1.36 (0.53-3.2)	318.39 (165.55-749.55)	2.28 (1.08-4.9)	1.86<br>(1.69-2.03)
Armenia	92 (74.54-118.53)	3.16 (2.59-4.02)	166.54 (138.05-199.1)	3.98 (3.3-4.77)	0.98<br>(0.78-1.18)
Azerbaijan	103.1 (67.89-148.64)	1.85 (1.21-2.66)	264.49 (167.17-400.78)	2.28 (1.46-3.44)	1.1

					(0.77-1.44)
Bahrain	10 (7.56-12.96)	4.28 (3.23-5.59)	51.26 (34.08-67.66)	4.52 (3.07-5.84)	-0.11 (-0.23-0.02)
Bangladesh	892.29 (574.69-1335.12)	1.55 (0.97-2.28)	3899.95 (2337.65-6821.25)	2.56 (1.57-4.48)	1.56 (1.48-1.63)
Bhutan	5.34 (3.27-8.41)	1.74 (1.06-2.73)	18.96 (12.15-34.05)	2.82 (1.82-5.03)	1.48 (1.43-1.54)
Brunei Darussalam	6.91 (5.09-9.65)	4.76 (3.63-6.36)	24.67 (19.13-31.19)	5.68 (4.4-6.99)	0.88 (0.75-1.01)
Cambodia	171.37 (100.53-325.56)	2.89 (1.74-5.28)	1010.26 (604.21-1550.69)	6.82 (4.18-10.3)	2.8 (2.75-2.86)
China	19997.6 (14086.43-26191.07)	2.03 (1.5-2.63)	41236.26 (30302.39-54548.52)	2.03 (1.49-2.69)	-0.41 (-0.56--0.27)
Democratic People's Republic of Korea	535.91 (328.36-773.48)	2.84 (1.77-4.07)	1240.68 (823.21-1749.94)	3.73 (2.44-5.28)	0.97 (0.9-1.03)

Georgia	104.22 (82.3-130.68)	1.66 (1.31-2.09)	397.99 (337.14-460.64)	7.36 (6.25-8.51)	5.98 (5.58-6.37)
India	9497.78 (6832.9-12690.63)	1.68 (1.21-2.22)	36777.16 (30565.31-43621.84)	2.82 (2.36-3.35)	1.57 (1.47-1.67)
Indonesia	3972.85 (2716.45-7572.62)	2.98 (2.1-5.57)	13250.48 (8573.93-21564.96)	4.58 (3.02-7.5)	1.19 (1.05-1.34)
Iran (Islamic Republic of)	358.16 (248.74-486.99)	1.08 (0.79-1.47)	1663.46 (1071.09-1932.89)	1.9 (1.23-2.21)	2.17 (2.01-2.33)
Iraq	173.85 (123.47-258.68)	1.79 (1.27-2.71)	823.02 (563.57-1187.69)	2.71 (1.92-3.79)	1.44 (1.35-1.54)
Japan	5955.86 (5674.44-6177.62)	3.64 (3.47-3.77)	8639.01 (7471.89-9348.18)	3.49 (3.21-3.69)	-0.14 (-0.31-0.04)
Jordan	32.52 (21.07-44.54)	1.86 (1.18-2.53)	216.18 (133.63-293.17)	2.33 (1.45-3.12)	0.6 (0.29-0.91)
Kazakhstan	539.87 (454.79-653.8)	3.92 (3.3-4.75)	835.92 (681.03-1005.95)	4.31 (3.51-5.18)	0.08

					(-0.2-0.35)
Kuwait	17.62 (16-19.28)	2.14 (1.94-2.34)	83.47 (69.76-100.34)	1.85 (1.56-2.21)	-0.6<br>(-0.92--0.27)
Kyrgyzstan	79.41 (66.5-95.68)	2.55 (2.12-3.08)	267.61 (211.93-333.05)	4.71 (3.68-5.84)	3.39<br>(2.7-4.1)
Lao People's Democratic Republic	66.71 (35.66-158.4)	2.62 (1.46-5.96)	318.77 (192.37-529.65)	5.25 (3.24-8.61)	2.41<br>(2.31-2.5)
Lebanon	68.3 (49.07-106.71)	2.94 (2.12-4.55)	226.33 (150.53-293.73)	3.79 (2.52-4.94)	1<br>(0.82-1.18)
Malaysia	307.54 (239.89-509.84)	2.73 (2.14-4.27)	1231.74 (962.12-1842.01)	3.96 (3.1-5.83)	1.49<br>(1.11-1.87)
Maldives	3.51 (1.95-9.73)	2.81 (1.61-7.44)	17.66 (12.28-24.97)	3.77 (2.72-4.93)	0.72<br>(0.27-1.17)
Mauritius	37.24 (34.56-40.24)	4.15 (3.85-4.47)	123.32 (112.08-132.94)	7.28 (6.6-7.83)	1.52<br>(1.11-1.93)

Mongolia	33.94 (23.19-47.95)	2.69 (1.85-3.77)	100.82 (71.84-133.09)	3.58 (2.53-4.71)	0.9 (0.78-1.02)
Myanmar	1035.04 (655.28-1891.03)	3.54 (2.29-6.21)	2970.23 (2037.31-4333.62)	5.4 (3.77-7.9)	1.18 (1.09-1.28)
Nepal	186.16 (102.14-296.64)	1.66 (0.93-2.61)	750.75 (502.61-1301.29)	2.89 (1.95-4.99)	1.75 (1.67-1.82)
Oman	7.06 (3.07-11.08)	0.86 (0.38-1.37)	28.45 (19.33-37.51)	1.08 (0.76-1.42)	0.77 (0.41-1.13)
Pakistan	1929.67 (1308.29-2389.19)	2.93 (1.99-3.67)	8217.65 (4850.47-12141.85)	5.17 (3.13-7.6)	1.64 (1.48-1.79)
Palestine	19.82 (12.85-29.87)	1.98 (1.28-2.95)	86.25 (62.14-107.57)	2.75 (1.98-3.41)	1.2 (1.13-1.27)
Philippines	1765.79 (1328.76-2089.68)	4.21 (3.11-4.97)	6490.86 (4658.33-8328.9)	6.44 (4.76-8.19)	1.4 (1.37-1.43)
Qatar	4.93 (3.5-6.64)	2.69 (1.93-3.64)	43.31 (27.33-62.92)	3.13 (2.07-4.13)	0.53

					(0.29-0.77)
Republic of Korea	726.26 (634-905.48)	1.95 (1.73-2.62)	2309.24 (1498.31-2721.15)	2.75 (1.84-3.24)	0.91 (0.71-1.12)
Saudi Arabia	97.03 (66.01-160.91)	1.28 (0.86-2.02)	597.43 (408.89-955.41)	1.92 (1.35-2.99)	1.19 (0.92-1.47)
Seychelles	3.67 (3.01-4.81)	6.05 (4.93-7.99)	10.24 (7.69-12.47)	8.45 (6.36-10.23)	1.31 (1.08-1.54)
Singapore	109.1 (102.2-116.16)	4.13 (3.87-4.39)	254.73 (231.93-277.35)	3.11 (2.83-3.37)	-0.92 (-1.14--0.7)
Sri Lanka	342.08 (281.63-441.33)	2.49 (2.05-3.13)	1035.64 (630.76-1457.03)	3.93 (2.41-5.5)	1.75 (1.58-1.92)
Syrian Arab Republic	74.31 (50.76-105.91)	1.12 (0.77-1.57)	235.17 (156.72-338.45)	1.63 (1.09-2.32)	1.02 (0.89-1.16)
Taiwan (Province of China)	531.92 (486.23-576.75)	2.88 (2.63-3.11)	1762.96 (1543.04-1958.68)	4.87 (4.24-5.38)	1.41 (1.13-1.68)

Tajikistan	38.93 (27.88-57.02)	1.26 (0.9-1.79)	98.09 (68.58-131.42)	1.28 (0.9-1.69)	-0.11 (-0.18--0.04)
Thailand	1565.8 (1090.05-1947.69)	3.52 (2.45-4.35)	5387.5 (3229.66-7362.21)	5.68 (3.27-7.8)	1.37 (1.21-1.54)
Timor-Leste	7.85 (4.28-14.36)	1.87 (1.03-3.34)	38.75 (23.83-53.88)	3.81 (2.4-5.33)	2.54 (2.3-2.77)
Turkey	1429.42 (968.22-2287.13)	3.57 (2.46-5.53)	3654.54 (2550.84-4706.66)	3.83 (2.67-4.91)	0.2 (0.07-0.32)
Turkmenistan	40.55 (35.97-45.29)	1.77 (1.57-1.97)	158.9 (119.45-214.56)	3.32 (2.51-4.47)	3.48 (1.39-5.62)
United Arab Emirates	30.89 (20.08-53.63)	4.63 (3.02-8.2)	249.82 (185.84-357)	5.64 (4.45-7.23)	2.1 (1.61-2.59)
Uzbekistan	187.93 (145.88-234.17)	1.44 (1.12-1.79)	708.61 (540.67-898.29)	2.23 (1.71-2.81)	1.18 (0.85-1.5)
Viet Nam	752.56 (553.07-973.51)	1.72 (1.23-2.24)	3097.66 (2005.51-4218.41)	2.9 (1.89-3.9)	1.64

					(1.54-1.74)
Yemen	39.9 (5.6-86.02)	0.7 (0.09-1.51)	229.21 (108-383.91)		2.17 (2.01-2.34)
Uterine cancer					
Afghanistan	146.14 (67.04-299.21)	1.95 (0.92-3.97)	431.68 (200.61-742.45)	3.7 (1.73-6.42)	2.33 (2.12-2.54)
Armenia	203.28 (189.2-219.33)	6.84 (6.34-7.34)	401.44 (332.93-487.32)	9.31 (7.73-11.25)	1.28 (0.74-1.82)
Azerbaijan	235.29 (178.32-284.74)	4.35 (3.3-5.29)	521.73 (378.51-767.06)	4.4 (3.22-6.31)	0.04 (-0.19-0.27)
Bahrain	7.28 (5.32-10.18)	3.66 (2.68-5.17)	65.82 (43.87-99.11)	6.06 (4.11-8.79)	1.24 (0.96-1.52)
Bangladesh	401.15 (266.48-673)	0.8 (0.54-1.37)	1491.61 (863.67-3413.75)	1.03 (0.6-2.34)	0.85 (0.68-1.02)
Bhutan	2.4 (1.36-4.31)	0.93 (0.53-1.68)	7.7 (4.43-17.8)	1.23 (0.71-2.84)	0.81

					(0.64-0.97)
Brunei Darussalam	3.05 (2.07-4.61)	2.46 (1.68-3.67)	14.71 (10.8-19.62)	3.45 (2.52-4.6)	1.57 (1.43-1.72)
Cambodia	158.1 (87.44-233)	3.06 (1.8-4.44)	590.36 (357.33-852.11)	4.2 (2.61-5.98)	0.99 (0.88-1.1)
China	26311.18 (18116.14-33311.82)	2.81 (1.97-3.53)	72018.5 (53311.86-99999.63)	3.35 (2.48-4.65)	0.44 (0.13-0.76)
Democratic People's Republic of Korea	585.88 (367.81-825.06)	3.11 (1.97-4.31)	1338.18 (808.11-1967.86)	3.8 (2.31-5.51)	0.85 (0.74-0.95)
Georgia	793.27 (715.24-879.23)	12.33 (11.11-13.58)	743.64 (626.54-875.21)	13.07 (11.02-15.32)	-0.04 (-0.55-0.47)
India	4156.6 (2954.21-5058.26)	0.83 (0.59-1.01)	16467.18 (13602.24-20842.73)	1.32 (1.09-1.67)	1.36 (1.09-1.64)
Indonesia	3062.6 (1913.19-4052.61)	2.62 (1.71-3.44)	10460.82 (6091.39-14914.23)	3.65 (2.17-5.13)	0.92 (0.86-0.98)

Iran (Islamic Republic of)	352.17 (219.16-452.15)	1.19 (0.75-1.5)	2142.76 (1020.21-2777.65)	2.52 (1.23-3.26)	2.65 (2.25-3.06)
Iraq	144.17 (87.66-264.95)	1.7 (1.04-3.11)	979.05 (653.38-1441.72)	3.55 (2.39-5.14)	2.74 (2.48-2.99)
Japan	3930.96 (3697.87-4109.14)	2.33 (2.19-2.43)	11770.44 (10574.21-12591.24)	5.26 (4.87-5.57)	3.02 (2.87-3.17)
Jordan	44.94 (31.81-64.11)	2.98 (2.13-4.18)	340.87 (225.24-511.93)	3.98 (2.62-5.93)	1.03 (0.63-1.43)
Kazakhstan	923.67 (803.81-1051.09)	6.94 (6.05-7.91)	1307.61 (1096.91-1518.71)	6.66 (5.59-7.7)	-0.07 (-0.7-0.56)
Kuwait	19.04 (16.84-21.74)	2.76 (2.44-3.16)	346.14 (288.94-408.67)	8.61 (7.14-10.18)	4.63 (4.02-5.26)
Kyrgyzstan	162.06 (132.95-193.43)	5.19 (4.25-6.2)	307.36 (230.89-385.02)	5.53 (4.2-6.91)	0.24 (-0.13-0.6)
Lao People's Democratic Republic	65.68 (32.3-104.46)	2.82 (1.45-4.45)	172.91 (103-249.79)	3.16 (1.94-4.53)	0.28

					(0.23-0.33)
Lebanon	76.75 (51.85-111.41)	3.33 (2.28-4.77)	278.6 (201.57-402.65)	4.8 (3.46-6.92)	1.36<br>(1.22-1.49)
Malaysia	288.46 (226.23-358.43)	2.87 (2.23-3.55)	1242.38 (960.74-1532.45)	4.09 (3.13-5.03)	1.23<br>(1.11-1.35)
Maldives	1.3 (0.61-2.08)	1.15 (0.57-1.79)	3.98 (2.81-5.76)	0.97 (0.7-1.34)	-0.72<br>(-0.89--0.55)
Mauritius	66.79 (61.18-72.43)	8.36 (7.66-9.06)	162.57 (144.84-177.98)	8.74 (7.78-9.55)	-0.8<br>(-1.47--0.13)
Mongolia	28.06 (19.09-42.73)	2.47 (1.7-3.76)	91.82 (60.36-131.1)	3.34 (2.26-4.65)	0.91<br>(0.75-1.07)
Myanmar	805.57 (444.82-1239.4)	3.07 (1.77-4.67)	1924.85 (1211.27-2716.22)	3.54 (2.26-4.96)	0.22<br>(0.13-0.32)
Nepal	82.07 (47.27-149.9)	0.82 (0.47-1.5)	273.88 (162.44-573.3)	1.12 (0.67-2.34)	1<br>(0.63-1.36)

Oman	5.12 (3.42-7.56)	0.72 (0.48-1.03)	25.85 (18.21-38.01)	1.18 (0.86-1.61)	1.87 (1.63-2.12)
Pakistan	1372.36 (1081.12-1794.79)	2.33 (1.83-3.06)	5427.8 (3800.42-7693.49)	4.05 (2.88-5.76)	1.52 (1.37-1.66)
Palestine	58.01 (37.52-83.67)	6.33 (4.15-9.06)	255.38 (167.5-344.4)	8.91 (5.9-11.89)	1.18 (1.09-1.28)
Philippines	945.88 (652.82-1175.97)	2.64 (1.89-3.33)	3370.99 (2539.52-4411.29)	3.55 (2.71-4.71)	0.91 (0.85-0.96)
Qatar	4.62 (3.02-7.28)	3.44 (2.27-5.46)	71.99 (49.34-108.94)	6.66 (4.79-9.62)	2.49 (2.13-2.86)
Republic of Korea	831.27 (453.08-1155.92)	2.43 (1.39-3.35)	1775.08 (1078.25-2537.31)	2.02 (1.23-2.86)	-0.14 (-0.51-0.24)
Saudi Arabia	72.01 (49.31-107.99)	1.1 (0.76-1.64)	804.56 (553.08-1129.98)	2.96 (2.1-4.07)	3.47 (3.3-3.65)
Seychelles	2.35 (1.79-3.11)	4.21 (3.2-5.58)	5.58 (4.17-7.48)	4.35 (3.29-5.86)	0.47

					(0.36-0.58)
Singapore	60.72 (54.76-66.63)	2.41 (2.17-2.66)	264.32 (233.25-303.77)	3.01 (2.66-3.44)	1.21 (0.94-1.48)
Sri Lanka	256.73 (189.86-340.19)	2.12 (1.58-2.78)	816.88 (475.91-1232.83)	2.94 (1.72-4.44)	1.06 (0.89-1.22)
Syrian Arab Republic	111.72 (83.08-149.53)	1.88 (1.41-2.54)	476.89 (333.9-712.1)	3.17 (2.22-4.67)	1.28 (1.08-1.48)
Taiwan (Province of China)	292.83 (264.75-321.74)	1.66 (1.5-1.83)	2187.04 (1882.36-2496.01)	5.53 (4.79-6.27)	4.13 (3.78-4.48)
Tajikistan	113.44 (80.02-154.13)	3.84 (2.7-5.19)	247.79 (139.5-424.97)	3.45 (2.01-5.7)	-0.52 (-0.66--0.37)
Thailand	981.3 (718.83-1275.39)	2.41 (1.78-3.16)	4095.81 (2403.3-5669.78)	3.8 (2.24-5.25)	1.1 (0.94-1.26)
Timor-Leste	7.28 (4.22-10.91)	2.09 (1.26-3.03)	24.49 (15.15-36.23)	2.69 (1.67-3.97)	1.02 (0.79-1.24)

Turkey	1571.72 (1070.95-2067.44)	4.18 (2.86-5.43)	6583.52 (4243.15-8779.61)	6.75 (4.32-8.97)	1.77 (1.46-2.08)
Turkmenistan	62.79 (56.82-68.99)	3 (2.71-3.3)	127.53 (94.97-171.58)	2.74 (2.06-3.68)	-0.37 (-1.06-0.33)
United Arab Emirates	32.08 (18.02-47.58)	6.78 (3.86-10.25)	315.61 (224.6-460.09)	8.53 (6.21-11.75)	2.34 (1.81-2.87)
Uzbekistan	408.98 (342.4-478.71)	3.39 (2.85-3.94)	1064.45 (846.77-1296.58)	3.4 (2.72-4.11)	0.49 (0.2-0.78)
Viet Nam	568.61 (426.77-779.3)	1.38 (1.04-1.88)	2262.3 (1541.96-3181.34)	2.1 (1.45-2.92)	1.56 (1.48-1.65)
Yemen	49.3 (28.81-89.89)	0.93 (0.56-1.67)	241.64 (148.52-412.53)	1.49 (0.91-2.47)	1.77 (1.62-1.92)

[a] represents data in parentheses is the 95% uncertainty interval; [b] represents data in parentheses is the 95% confidence interval; ASMR= age-standardized mortality rate; DALY= disability-adjusted life year; ASDR= age-standardized DALY rate; ASIR= age-standardized incidence rate; EAPC= estimated annual percent change; ASR= age-standardized rate.

Table S7. Effects of the ageing, population, and epidemiological change on the ASDR by GBD region from 1990 to 2021 attributable to gynecological cancer in Asia.

Location	Overall difference	Aging	Population	Epidemiological change
Cervical cancer				
High-income Asia Pacific	335.25	2470.33 (736.85%)	1196.64 (356.94%)	-3331.72 (-993.79%)
Central Asia	447.82	342.61 (76.51%)	1423.86 (317.95%)	-1318.65 (-294.46%)
South Asia	18211.21	8570.74 (47.06%)	47715.43 (262.01%)	-38074.97 (-209.07%)

East Asia	18476.62	19626.56 (106.22%)	17231.76 (93.26%)	-18381.69 (-99.49%)
Southeast Asia	11091.68	5755.99 (51.89%)	14184.62 (127.89%)	-8848.92 (-79.78%)
Asia	49705.51	37330.87 (75.1%)	79697.02 (160.34%)	-67322.39 (-135.44%)
Ovarian cancer				
High-income Asia Pacific	3041.42	2653.46 (87.24%)	1272.21 (41.83%)	-884.25 (-29.07%)
Central Asia	1149.24	137.08 (11.93%)	616.2 (53.62%)	395.96 (34.45%)
South Asia	22648.3	3055.74 (13.49%)	12093.67 (53.4%)	7498.89 (33.11%)
East Asia	14160.04	8871.97 (62.65%)	7341.46 (51.85%)	-2053.39 (-14.5%)
Southeast Asia	10267.19	2313.64 (22.53%)	5255.51 (51.19%)	2698.04 (26.28%)

Asia	53122.29	16936.84<br>(31.88%)	30151.95<br>(56.76%)	6033.5<br>(11.36%)
Uterine cancer				
High-income Asia Pacific	1843.85	1828.07<br>(99.14%)	691.64<br>(37.51%)	-675.86<br>(-36.65%)
Central Asia	224	110.61<br>(49.38%)	559.47<br>(249.76%)	-446.08<br>(-199.14%)
South Asia	6022.24	1323.89<br>(21.98%)	4199.11<br>(69.73%)	499.24<br>(8.29%)
East Asia	3328.95	6768.04<br>(203.31%)	5276.66<br>(158.51%)	-8715.75<br>(-261.82%)
Southeast Asia	4582.98	1480.24<br>(32.3%)	3024.58<br>(66%)	78.16<br>(1.71%)
Asia	17194	10194.68<br>(59.29%)	16383.86<br>(95.29%)	-9384.54<br>(-54.58%)

ASDR= age-standardized disability-adjusted life year rate.

Table S8. Annual percent change of the deaths attributable to gynecological cancers by age groups and GBD region in Asia.

Characteristic	Cervical cancer		Ovarian cancer		Uterine cancer	
	Local drift	Net drift	Local drift	Net drift	Local drift	Net drift
Asia						
15-19 years	-2.17 (-2.92--1.43)		0.65 (0.33-0.96)			
20-24 years	-1.89 (-2.3--1.48)		0.65 (0.42-0.87)		-1.87 (-2.33--1.4)	
25-29 years	-1.74 (-2.01--1.47)		0.58 (0.4-0.76)		-1.91 (-2.19--1.62)	
30-34 years	-1.73 (-1.93--1.53)		0.4 (0.24-0.55)		-1.96 (-2.16--1.76)	
35-39 years	-1.58 (-1.74--1.42)	-1.39 (-1.55--1.23)	0.27 (0.14-0.4)	0.17 (0.08-0.26)	-1.93 (-2.09--1.78)	-1.26 (-1.36--1.17)
40-44 years	-1.47 (-1.6--1.34)		0.13 (0.02-0.24)		-1.73 (-1.85--1.61)	
45-49 years	-1.48 (-1.6--1.36)		-0.02 (-0.11-0.07)		-1.43 (-1.53--1.32)	
50-54 years	-1.52 (-1.64--1.41)		-0.08 (-0.17-0)		-1.21 (-1.3--1.12)	
55-59 years	-1.53 (-1.66--1.41)		0.02 (-0.06-0.11)		-1.12 (-1.21--1.04)	

60-64 years	-1.58 (-1.72--1.44)		0.12 (0.03-0.2)		-1.02 (-1.1--0.93)	
65-69 years	-1.38 (-1.55--1.21)		0.13 (0.02-0.23)		-1 (-1.1--0.91)	
70-74 years	-1.19 (-1.4--0.97)		0.09 (-0.04-0.22)		-1.13 (-1.25--1.02)	
75-79 years	-1.04 (-1.35--0.74)		-0.03 (-0.2-0.15)		-1.26 (-1.41--1.1)	
80-84 years	-0.92 (-1.37--0.47)		-0.18 (-0.43-0.08)		-1.25 (-1.47--1.03)	
85-89 years	-0.84 (-1.59--0.08)		-0.03 (-0.44-0.39)		-1.02 (-1.39--0.65)	
90-94 years	-0.68 (-2.24-0.9)		0.49 (-0.34-1.33)		-0.5 (-1.22-0.24)	
95+ years	-0.49 (-4.32-3.5)		1.3 (-0.85-3.49)		0.34 (-1.61-2.33)	
Central Asia						
15-19 years	-2.15 (-2.89--1.41)		0.62 (0.1-1.14)			
20-24 years	-2.58 (-2.98--2.17)		0.58 (0.22-0.94)		-1.88 (-2.83--0.92)	
25-29 years	-2.7 (-2.93--2.46)		0.48 (0.2-0.76)		-2.07 (-2.67--1.46)	
30-34 years	-2.23 (-2.38--2.08)		0.55 (0.32-0.79)		-1.98 (-2.42--1.55)	
35-39 years	-1.49 (-1.6--1.37)		0.61 (0.42-0.81)		-1.8 (-2.14--1.46)	
40-44 years	-0.94 (-1.03--0.84)		0.52 (0.36-0.69)		-1.69 (-1.97--1.42)	
45-49 years	-0.7 (-0.8--0.61)		0.51 (0.36-0.65)		-1.63 (-1.86--1.39)	
50-54 years	-0.69 (-0.78--0.59)	-1.31 (-1.42--1.2)	0.62 (0.48-0.75)	1.08 (0.92-1.24)	-1.43 (-1.63--1.22)	-1.11 (-1.3--0.92)
55-59 years	-0.85 (-0.95--0.75)		0.8 (0.67-0.93)		-1.21 (-1.39--1.03)	
60-64 years	-1.03 (-1.14--0.92)		1.19 (1.05-1.33)		-0.93 (-1.11--0.75)	
65-69 years	-1.37 (-1.5--1.24)		1.36 (1.2-1.52)		-0.85 (-1.04--0.65)	
70-74 years	-1.82 (-1.99--1.65)		1.4 (1.18-1.62)		-0.91 (-1.16--0.66)	
75-79 years	-1.83 (-2.06--1.6)		1.65 (1.33-1.96)		-0.73 (-1.09--0.38)	
80-84 years	-1.38 (-1.72--1.04)		2.07 (1.59-2.55)		-0.33 (-0.85-0.2)	
85-89 years	-0.65 (-1.26--0.04)		2.43 (1.57-3.29)		0.22 (-0.74-1.19)	
90-94 years	-0.26 (-1.3-0.79)		2.59 (1-4.22)		0.39 (-1.17-1.97)	

95+ years	-0.79 (-2.84-1.29)		1.66 (-1.79-5.23)		-0.3 (-3.23-2.71)	
East Asia						
15-19 years	-2.65 (-3.72--1.57)		-1.59 (-2.64--0.54)			
20-24 years	-2.12 (-2.64--1.59)		-0.94 (-1.59--0.28)		-2.48 (-3.32--1.64)	
25-29 years	-1.85 (-2.14--1.56)		-0.76 (-1.24--0.27)		-2.56 (-3.03--2.09)	
30-34 years	-1.5 (-1.69--1.31)		-0.84 (-1.22--0.47)		-2.63 (-2.93--2.32)	
35-39 years	-1.21 (-1.35--1.06)		-0.89 (-1.2--0.58)		-2.66 (-2.9--2.43)	
40-44 years	-1.02 (-1.14--0.9)		-0.94 (-1.18--0.7)		-2.66 (-2.83--2.48)	
45-49 years	-0.84 (-0.94--0.74)		-1.01 (-1.21--0.81)		-2.61 (-2.75--2.47)	
50-54 years	-0.68 (-0.78--0.58)		-1 (-1.19--0.82)		-2.51 (-2.64--2.38)	
55-59 years	-0.75 (-0.86--0.64)	-1.08 (-1.23--0.93)	-0.87 (-1.06--0.69)	-1 (-1.27--0.74)	-2.46 (-2.58--2.33)	-2.46 (-2.65--2.28)
60-64 years	-0.78 (-0.9--0.66)		-0.75 (-0.95--0.55)		-2.31 (-2.44--2.18)	
65-69 years	-0.86 (-1--0.72)		-0.85 (-1.08--0.62)		-2.3 (-2.45--2.15)	
70-74 years	-1.07 (-1.24--0.9)		-0.89 (-1.18--0.6)		-2.39 (-2.57--2.21)	
75-79 years	-1.26 (-1.48--1.04)		-1 (-1.39--0.61)		-2.52 (-2.76--2.29)	
80-84 years	-1.19 (-1.53--0.85)		-1.31 (-1.9--0.71)		-2.55 (-2.91--2.2)	
85-89 years	-1.01 (-1.62--0.4)		-1.51 (-2.57--0.44)		-2.48 (-3.13--1.83)	
90-94 years	-0.63 (-2.03-0.79)		-1.5 (-3.88-0.93)		-2.15 (-3.63--0.65)	
95+ years	-0.51 (-4.32-3.45)		-1.63 (-8.4-5.65)		-1.79 (-6.24-2.87)	
High-income Asia Pacific						
15-19 years	-1.98 (-3.84--0.08)		-1.44 (-2.05--0.84)			
20-24 years	-1.72 (-2.55--0.9)	-1.69 (-1.78--1.59)	-1.62 (-2.01--1.24)	-0.8 (-0.87--0.74)	0.24 (-0.86-1.35)	0.2 (0.12-0.28)
25-29 years	-1.39 (-1.77--1)		-1.52 (-1.81--1.23)		0.29 (-0.25-0.84)	
30-34 years	-0.85 (-1.07--0.63)		-1.21 (-1.42--1)		0.67 (0.32-1.02)	

35-39 years	-0.67 (-0.82--0.51)		-1.04 (-1.19--0.88)		1.01 (0.77-1.24)	
40-44 years	-0.65 (-0.77--0.53)		-1.01 (-1.13--0.9)		1.1 (0.93-1.26)	
45-49 years	-0.56 (-0.67--0.45)		-0.93 (-1.02--0.84)		1.1 (0.98-1.23)	
50-54 years	-0.77 (-0.87--0.67)		-0.79 (-0.87--0.71)		0.95 (0.85-1.05)	
55-59 years	-1.21 (-1.31--1.1)		-0.57 (-0.65--0.49)		0.71 (0.62-0.8)	
60-64 years	-1.84 (-1.95--1.73)		-0.51 (-0.59--0.44)		0.36 (0.28-0.45)	
65-69 years	-2.42 (-2.53--2.3)		-0.58 (-0.66--0.49)		0.03 (-0.06-0.11)	
70-74 years	-3.02 (-3.15--2.89)		-0.97 (-1.06--0.87)		-0.6 (-0.69--0.51)	
75-79 years	-3.24 (-3.39--3.1)		-1.14 (-1.25--1.02)		-1.04 (-1.15--0.93)	
80-84 years	-3.18 (-3.35--3)		-0.96 (-1.11--0.82)		-1.2 (-1.33--1.06)	
85-89 years	-2.71 (-2.96--2.47)		-0.31 (-0.53--0.1)		-1.06 (-1.25--0.87)	
90-94 years	-2.04 (-2.47--1.6)		0.51 (0.13-0.89)		-0.63 (-0.96--0.3)	
95+ years	-1.28 (-2.35--0.2)		1.43 (0.5-2.38)		0.33 (-0.55-1.21)	
South Asia						
15-19 years	-2.97 (-4.23--1.7)		1.4 (1.17-1.62)			
20-24 years	-2.48 (-3.19--1.77)		1.49 (1.32-1.67)		-0.46 (-0.93-0.02)	
25-29 years	-2.07 (-2.58--1.57)		1.59 (1.44-1.74)		-0.04 (-0.35-0.26)	
30-34 years	-2.13 (-2.51--1.75)		1.51 (1.38-1.64)		0.05 (-0.18-0.29)	
35-39 years	-2.08 (-2.39--1.77)		1.42 (1.31-1.54)		0.21 (0.02-0.41)	
40-44 years	-2.02 (-2.28--1.76)	-1.61 (-2.07--1.15)	1.3 (1.2-1.4)	1.41 (1.28-1.53)	0.25 (0.09-0.41)	0.16 (0.03-0.3)
45-49 years	-2.09 (-2.32--1.85)		1.14 (1.06-1.23)		0.16 (0.03-0.3)	
50-54 years	-2.19 (-2.42--1.95)		1.03 (0.95-1.1)		0.04 (-0.08-0.16)	
55-59 years	-2.15 (-2.4--1.91)		1.03 (0.95-1.1)		0.03 (-0.08-0.13)	
60-64 years	-2.2 (-2.48--1.91)		1.07 (0.99-1.15)		-0.08 (-0.17-0.02)	
65-69 years	-1.65 (-2.02--1.29)		1.34 (1.24-1.43)		0.1 (-0.01-0.21)	

70-74 years	-1.08 (-1.58--0.58)		1.58 (1.45-1.71)		0.17 (0.04-0.31)	
75-79 years	-0.51 (-1.28-0.26)		1.74 (1.55-1.93)		0.24 (0.05-0.43)	
80-84 years	-0.05 (-1.26-1.18)		1.89 (1.6-2.19)		0.36 (0.07-0.65)	
85-89 years	0.02 (-2.04-2.11)		1.87 (1.35-2.39)		0.49 (-0.02-1.01)	
90-94 years	-0.05 (-4.67-4.8)		1.87 (0.7-3.05)		0.63 (-0.42-1.7)	
95+ years	0.06 (-11.81-13.53)		1.89 (-1.51-5.41)		0.93 (-2.31-4.28)	
Southeast Asia						
15-19 years	-2.74 (-3.12--2.35)		0.97 (0.86-1.08)			
20-24 years	-2.74 (-2.97--2.52)		0.9 (0.82-0.98)		-1.07 (-1.24--0.9)	
25-29 years	-2.48 (-2.62--2.33)		0.84 (0.78-0.91)		-0.88 (-0.99--0.76)	
30-34 years	-2.15 (-2.25--2.04)		0.87 (0.82-0.92)		-0.57 (-0.66--0.48)	
35-39 years	-1.91 (-1.99--1.83)		0.91 (0.87-0.96)		-0.29 (-0.36--0.22)	
40-44 years	-1.75 (-1.82--1.68)		0.9 (0.86-0.94)		-0.15 (-0.2--0.1)	
45-49 years	-1.67 (-1.74--1.61)		0.82 (0.78-0.85)		-0.14 (-0.18--0.09)	
50-54 years	-1.67 (-1.73--1.6)		0.74 (0.71-0.78)		-0.19 (-0.23--0.15)	
55-59 years	-1.65 (-1.72--1.58)	-1.45 (-1.53--1.36)	0.73 (0.7-0.76)	0.98 (0.94-1.03)	-0.21 (-0.25--0.17)	-0.13 (-0.18--0.08)
60-64 years	-1.55 (-1.63--1.47)		0.84 (0.8-0.88)		-0.16 (-0.2--0.12)	
65-69 years	-1.31 (-1.4--1.22)		1.02 (0.97-1.07)		-0.05 (-0.1--0.01)	
70-74 years	-1.02 (-1.15--0.9)		1.16 (1.1-1.22)		0 (-0.05-0.06)	
75-79 years	-0.66 (-0.83--0.49)		1.21 (1.13-1.3)		0.03 (-0.05-0.12)	
80-84 years	-0.32 (-0.58--0.06)		1.25 (1.12-1.38)		0.08 (-0.05-0.21)	
85-89 years	-0.14 (-0.57-0.3)		1.31 (1.1-1.52)		0.26 (0.03-0.48)	
90-94 years	-0.21 (-1.03-0.63)		1.46 (1.04-1.88)		0.44 (0-0.88)	
95+ years	-0.51 (-2.37-1.39)		1.64 (0.58-2.7)		0.49 (-0.6-1.59)	

Period RR			
Asia			
1992 to 1996	1 (1-1)	1 (1-1)	1 (1-1)
1997 to 2001	0.9 (0.88-0.92)	1.03 (1.01-1.04)	0.98 (0.96-1)
2002 to 2006	0.82 (0.8-0.85)	1.01 (0.99-1.03)	0.94 (0.93-0.96)
2007 to 2011	0.74 (0.72-0.77)	1 (0.98-1.02)	0.87 (0.85-0.88)
2012 to 2016	0.72 (0.7-0.75)	1.01 (0.99-1.04)	0.77 (0.75-0.78)
2017 to 2021	0.71 (0.68-0.74)	1.07 (1.04-1.1)	0.75 (0.74-0.77)
Central Asia			
1992 to 1996	1 (1-1)	1 (1-1)	1 (1-1)
1997 to 2001	0.93 (0.92-0.95)	0.97 (0.94-0.99)	0.97 (0.94-1.01)
2002 to 2006	0.88 (0.87-0.9)	1.06 (1.03-1.09)	0.84 (0.81-0.88)
2007 to 2011	0.87 (0.86-0.89)	1.17 (1.14-1.21)	0.75 (0.72-0.78)
2012 to 2016	0.77 (0.75-0.79)	1.21 (1.17-1.26)	0.8 (0.77-0.84)
2017 to 2021	0.71 (0.69-0.73)	1.24 (1.19-1.3)	0.78 (0.74-0.82)
East Asia			
1992 to 1996	1 (1-1)	1 (1-1)	1 (1-1)
1997 to 2001	0.89 (0.87-0.91)	0.98 (0.94-1.02)	0.96 (0.94-0.99)
2002 to 2006	0.92 (0.9-0.94)	0.88 (0.84-0.91)	0.93 (0.9-0.96)
2007 to 2011	0.83 (0.81-0.85)	0.82 (0.78-0.87)	0.81 (0.79-0.84)
2012 to 2016	0.78 (0.76-0.81)	0.78 (0.73-0.82)	0.61 (0.59-0.64)
2017 to 2021	0.76 (0.73-0.79)	0.82 (0.76-0.88)	0.56 (0.54-0.59)
High-income Asia Pacific			

1992 to 1996	1 (1-1)	1 (1-1)	1 (1-1)
1997 to 2001	0.94 (0.92-0.96)	0.96 (0.95-0.98)	0.97 (0.96-0.99)
2002 to 2006	0.85 (0.83-0.86)	0.92 (0.91-0.93)	0.97 (0.95-0.99)
2007 to 2011	0.77 (0.75-0.79)	0.89 (0.88-0.91)	0.98 (0.97-1)
2012 to 2016	0.72 (0.7-0.73)	0.86 (0.84-0.87)	1.04 (1.01-1.06)
2017 to 2021	0.66 (0.64-0.68)	0.82 (0.8-0.83)	1.03 (1.01-1.05)
South Asia			
1992 to 1996	1 (1-1)	1 (1-1)	1 (1-1)
1997 to 2001	0.88 (0.83-0.92)	1.09 (1.07-1.11)	1 (0.98-1.03)
2002 to 2006	0.73 (0.68-0.77)	1.14 (1.12-1.16)	0.96 (0.93-0.98)
2007 to 2011	0.66 (0.6-0.71)	1.19 (1.16-1.22)	0.95 (0.92-0.97)
2012 to 2016	0.67 (0.61-0.74)	1.3 (1.26-1.33)	0.99 (0.96-1.02)
2017 to 2021	0.68 (0.6-0.76)	1.45 (1.41-1.5)	1.07 (1.03-1.11)
Southeast Asia			
1992 to 1996	1 (1-1)	1 (1-1)	1 (1-1)
1997 to 2001	0.97 (0.96-0.98)	1.08 (1.08-1.09)	1.04 (1.03-1.04)
2002 to 2006	0.9 (0.89-0.92)	1.15 (1.14-1.16)	1.03 (1.02-1.04)
2007 to 2011	0.81 (0.8-0.82)	1.19 (1.18-1.2)	1 (0.99-1.01)
2012 to 2016	0.75 (0.73-0.76)	1.24 (1.23-1.25)	0.98 (0.97-0.99)
2017 to 2021	0.72 (0.7-0.73)	1.29 (1.28-1.3)	0.99 (0.98-1.01)
**Cohort RR**			
Asia			
1892 to 1901	2.3 (0.61-8.66)	0.72 (0.35-1.48)	1.75 (0.9-3.41)

1897 to 1906	2.24 (1.33-3.78)	0.84 (0.64-1.1)	1.99 (1.56-2.54)
1902 to 1911	2.25 (1.76-2.88)	0.94 (0.82-1.08)	2.1 (1.86-2.37)
1907 to 1916	2.17 (1.87-2.51)	1.01 (0.93-1.1)	2.14 (1.98-2.3)
1912 to 1921	2.13 (1.93-2.35)	1.01 (0.95-1.07)	2.07 (1.96-2.18)
1917 to 1926	2.02 (1.88-2.16)	0.99 (0.95-1.03)	1.92 (1.84-2.01)
1922 to 1931	1.89 (1.78-2)	0.97 (0.93-1)	1.77 (1.71-1.84)
1927 to 1936	1.84 (1.76-1.93)	0.96 (0.93-1)	1.66 (1.61-1.72)
1932 to 1941	1.73 (1.66-1.81)	0.98 (0.96-1.01)	1.59 (1.54-1.64)
1937 to 1946	1.62 (1.56-1.68)	1 (0.98-1.03)	1.51 (1.46-1.55)
1942 to 1951	1.47 (1.42-1.52)	0.99 (0.97-1.02)	1.44 (1.39-1.48)
1947 to 1956	1.34 (1.3-1.39)	0.99 (0.96-1.01)	1.38 (1.34-1.42)
1952 to 1961	1.25 (1.21-1.29)	1 (0.98-1.03)	1.28 (1.24-1.32)
1957 to 1966	1.2 (1.16-1.24)	0.99 (0.97-1.02)	1.19 (1.16-1.22)
1962 to 1971	1.09 (1.05-1.12)	0.97 (0.95-1)	1.12 (1.09-1.15)
1967 to 1976	1 (1-1)	1 (1-1)	1 (1-1)
1972 to 1981	0.93 (0.9-0.97)	1.04 (1.01-1.07)	0.88 (0.85-0.91)
1977 to 1986	0.85 (0.81-0.89)	1.07 (1.03-1.11)	0.79 (0.76-0.83)
1982 to 1991	0.77 (0.72-0.81)	1.07 (1.03-1.12)	0.75 (0.71-0.8)
1987 to 1996	0.7 (0.65-0.76)	1.14 (1.08-1.2)	0.69 (0.63-0.75)
1992 to 2001	0.62 (0.54-0.71)	1.19 (1.11-1.27)	0.6 (0.52-0.7)
1997 to 2006	0.53 (0.41-0.68)	1.21 (1.1-1.33)	
Central Asia			
1892 to 1901	2.46 (1.25-4.84)	0.44 (0.14-1.38)	2.15 (0.82-5.64)
1897 to 1906	1.92 (1.38-2.68)	0.37 (0.22-0.62)	1.74 (1.07-2.85)
1902 to 1911	1.72 (1.41-2.09)	0.39 (0.29-0.51)	1.58 (1.15-2.17)

1907 to 1916	1.82 (1.63-2.02)	0.44 (0.38-0.52)	1.7 (1.42-2.02)
1912 to 1921	1.86 (1.73-2)	0.52 (0.47-0.58)	1.77 (1.57-2.01)
1917 to 1926	1.88 (1.78-1.98)	0.62 (0.57-0.66)	1.88 (1.71-2.07)
1922 to 1931	1.65 (1.59-1.72)	0.67 (0.63-0.7)	1.78 (1.64-1.93)
1927 to 1936	1.44 (1.39-1.5)	0.68 (0.65-0.71)	1.64 (1.52-1.76)
1932 to 1941	1.33 (1.29-1.38)	0.76 (0.73-0.8)	1.6 (1.49-1.72)
1937 to 1946	1.23 (1.19-1.27)	0.81 (0.77-0.84)	1.53 (1.43-1.65)
1942 to 1951	1.2 (1.16-1.23)	0.87 (0.84-0.91)	1.5 (1.4-1.61)
1947 to 1956	1.16 (1.13-1.19)	0.91 (0.87-0.94)	1.4 (1.31-1.5)
1952 to 1961	1.1 (1.07-1.12)	0.91 (0.88-0.95)	1.28 (1.2-1.37)
1957 to 1966	1.06 (1.04-1.09)	0.93 (0.9-0.96)	1.18 (1.11-1.26)
1962 to 1971	1.05 (1.02-1.07)	0.97 (0.93-1)	1.09 (1.02-1.16)
1967 to 1976	1 (1-1)	1 (1-1)	1 (1-1)
1972 to 1981	0.88 (0.86-0.91)	1.02 (0.98-1.07)	0.91 (0.84-0.99)
1977 to 1986	0.73 (0.71-0.76)	1.06 (1-1.12)	0.81 (0.73-0.9)
1982 to 1991	0.61 (0.59-0.64)	1.06 (1-1.13)	0.71 (0.63-0.82)
1987 to 1996	0.56 (0.52-0.6)	1.09 (1.01-1.18)	0.66 (0.54-0.79)
1992 to 2001	0.55 (0.48-0.62)	1.18 (1.06-1.31)	0.64 (0.47-0.87)
1997 to 2006	0.5 (0.39-0.64)	1.18 (1.01-1.39)	
East Asia			
1892 to 1901	1.95 (0.52-7.36)	2.49 (0.22-28.42)	5.69 (1.17-27.72)
1897 to 1906	1.8 (1.12-2.9)	2.18 (0.96-4.93)	5.32 (3.2-8.87)
1902 to 1911	1.82 (1.49-2.23)	2.08 (1.46-2.97)	5.04 (4.06-6.28)
1907 to 1916	1.78 (1.59-1.98)	1.94 (1.59-2.36)	4.57 (4.05-5.15)
1912 to 1921	1.77 (1.64-1.9)	1.76 (1.54-2)	4.13 (3.8-4.48)

1917 to 1926	1.66 (1.57-1.75)	1.62 (1.47-1.79)	3.59 (3.37-3.83)
1922 to 1931	1.53 (1.46-1.6)	1.52 (1.4-1.65)	3.11 (2.94-3.28)
1927 to 1936	1.41 (1.36-1.47)	1.44 (1.34-1.55)	2.71 (2.58-2.85)
1932 to 1941	1.36 (1.31-1.4)	1.4 (1.31-1.49)	2.45 (2.33-2.56)
1937 to 1946	1.3 (1.26-1.34)	1.36 (1.28-1.45)	2.18 (2.09-2.29)
1942 to 1951	1.27 (1.23-1.3)	1.27 (1.2-1.35)	1.94 (1.86-2.03)
1947 to 1956	1.22 (1.18-1.25)	1.22 (1.15-1.29)	1.72 (1.65-1.79)
1952 to 1961	1.15 (1.12-1.19)	1.22 (1.15-1.28)	1.51 (1.45-1.57)
1957 to 1966	1.13 (1.1-1.16)	1.11 (1.05-1.17)	1.31 (1.26-1.36)
1962 to 1971	1.11 (1.08-1.14)	1.04 (0.98-1.1)	1.17 (1.12-1.22)
1967 to 1976	1 (1-1)	1 (1-1)	1 (1-1)
1972 to 1981	0.93 (0.9-0.96)	1 (0.93-1.07)	0.88 (0.83-0.93)
1977 to 1986	0.87 (0.83-0.9)	0.96 (0.87-1.05)	0.77 (0.72-0.83)
1982 to 1991	0.78 (0.74-0.83)	0.87 (0.78-0.97)	0.68 (0.62-0.75)
1987 to 1996	0.68 (0.62-0.74)	0.87 (0.76-1.01)	0.61 (0.53-0.71)
1992 to 2001	0.58 (0.49-0.7)	0.79 (0.65-0.97)	0.53 (0.4-0.7)
1997 to 2006	0.47 (0.33-0.68)	0.64 (0.45-0.9)	
High-income Asia Pacific			
1892 to 1901	3.46 (2.39-5.01)	1.1 (0.8-1.5)	0.77 (0.57-1.04)
1897 to 1906	3.43 (2.96-3.98)	1.27 (1.12-1.44)	0.92 (0.82-1.03)
1902 to 1911	3.36 (3.09-3.65)	1.43 (1.33-1.53)	0.97 (0.9-1.04)
1907 to 1916	3.21 (3.02-3.41)	1.56 (1.48-1.63)	0.96 (0.91-1.01)
1912 to 1921	2.89 (2.75-3.04)	1.59 (1.52-1.65)	0.92 (0.88-0.97)
1917 to 1926	2.47 (2.36-2.58)	1.55 (1.5-1.61)	0.86 (0.83-0.9)
1922 to 1931	2.05 (1.97-2.14)	1.44 (1.39-1.48)	0.8 (0.77-0.83)

1927 to 1936	1.73 (1.67-1.79)	1.34 (1.3-1.39)	0.76 (0.73-0.79)
1932 to 1941	1.46 (1.41-1.51)	1.24 (1.21-1.28)	0.72 (0.7-0.75)
1937 to 1946	1.29 (1.25-1.33)	1.23 (1.2-1.27)	0.72 (0.69-0.75)
1942 to 1951	1.15 (1.12-1.19)	1.23 (1.2-1.26)	0.75 (0.72-0.78)
1947 to 1956	1.14 (1.11-1.17)	1.24 (1.21-1.27)	0.81 (0.78-0.84)
1952 to 1961	1.07 (1.04-1.1)	1.13 (1.1-1.15)	0.8 (0.77-0.82)
1957 to 1966	1.07 (1.04-1.1)	1.07 (1.05-1.1)	0.86 (0.83-0.89)
1962 to 1971	1.04 (1.01-1.07)	1.03 (1.01-1.06)	0.93 (0.89-0.96)
1967 to 1976	1 (1-1)	1 (1-1)	1 (1-1)
1972 to 1981	0.95 (0.92-0.99)	0.94 (0.91-0.97)	1.02 (0.97-1.07)
1977 to 1986	0.92 (0.88-0.96)	0.85 (0.81-0.89)	1 (0.93-1.08)
1982 to 1991	0.87 (0.81-0.93)	0.8 (0.75-0.85)	1.03 (0.92-1.15)
1987 to 1996	0.7 (0.62-0.8)	0.71 (0.65-0.77)	1.01 (0.85-1.2)
1992 to 2001	0.66 (0.5-0.87)	0.68 (0.6-0.76)	1.08 (0.76-1.56)
1997 to 2006	0.6 (0.32-1.14)	0.66 (0.54-0.81)	
South Asia			
1892 to 1901	2.2 (0.03-159.63)	0.32 (0.1-1.03)	0.75 (0.24-2.28)
1897 to 1906	2.25 (0.46-10.99)	0.35 (0.24-0.52)	0.8 (0.56-1.13)
1902 to 1911	2.32 (1.19-4.53)	0.39 (0.33-0.46)	0.83 (0.7-0.99)
1907 to 1916	2.18 (1.47-3.23)	0.42 (0.38-0.46)	0.88 (0.8-0.97)
1912 to 1921	2.25 (1.75-2.89)	0.47 (0.44-0.5)	0.93 (0.86-0.99)
1917 to 1926	2.28 (1.94-2.69)	0.51 (0.49-0.53)	0.93 (0.89-0.99)
1922 to 1931	2.22 (1.97-2.51)	0.56 (0.54-0.58)	0.92 (0.88-0.96)
1927 to 1936	2.3 (2.09-2.53)	0.62 (0.6-0.64)	0.96 (0.92-1)
1932 to 1941	2.13 (1.95-2.31)	0.68 (0.66-0.7)	0.97 (0.94-1.02)

1937 to 1946	1.95 (1.8-2.1)	0.71 (0.7-0.73)	0.97 (0.94-1.01)
1942 to 1951	1.72 (1.6-1.84)	0.75 (0.73-0.77)	0.96 (0.92-0.99)
1947 to 1956	1.5 (1.41-1.61)	0.78 (0.77-0.8)	0.96 (0.92-0.99)
1952 to 1961	1.33 (1.25-1.42)	0.82 (0.8-0.84)	0.95 (0.92-0.99)
1957 to 1966	1.28 (1.2-1.36)	0.88 (0.87-0.9)	1 (0.96-1.04)
1962 to 1971	1.1 (1.03-1.17)	0.91 (0.9-0.93)	0.96 (0.93-1)
1967 to 1976	1 (1-1)	1 (1-1)	1 (1-1)
1972 to 1981	0.9 (0.84-0.97)	1.09 (1.06-1.11)	1.02 (0.98-1.07)
1977 to 1986	0.8 (0.73-0.88)	1.17 (1.13-1.2)	1 (0.95-1.06)
1982 to 1991	0.74 (0.66-0.83)	1.27 (1.23-1.31)	0.99 (0.92-1.06)
1987 to 1996	0.65 (0.55-0.75)	1.36 (1.3-1.41)	0.96 (0.87-1.05)
1992 to 2001	0.52 (0.41-0.64)	1.44 (1.37-1.52)	0.89 (0.76-1.03)
1997 to 2006	0.42 (0.28-0.64)	1.53 (1.43-1.64)	
Southeast Asia			
1892 to 1901	2.28 (1.22-4.28)	0.42 (0.29-0.59)	0.95 (0.66-1.38)
1897 to 1906	2.08 (1.59-2.73)	0.45 (0.4-0.52)	0.94 (0.82-1.09)
1902 to 1911	1.98 (1.72-2.27)	0.5 (0.47-0.53)	0.97 (0.9-1.04)
1907 to 1916	1.95 (1.79-2.12)	0.54 (0.52-0.57)	1.02 (0.98-1.07)
1912 to 1921	1.98 (1.87-2.09)	0.59 (0.57-0.6)	1.04 (1.02-1.07)
1917 to 1926	1.98 (1.9-2.06)	0.62 (0.61-0.63)	1.05 (1.03-1.07)
1922 to 1931	1.93 (1.87-1.99)	0.65 (0.64-0.66)	1.04 (1.02-1.06)
1927 to 1936	1.9 (1.85-1.94)	0.7 (0.69-0.71)	1.05 (1.03-1.06)
1932 to 1941	1.79 (1.75-1.84)	0.75 (0.74-0.76)	1.05 (1.04-1.07)
1937 to 1946	1.67 (1.63-1.7)	0.79 (0.78-0.8)	1.05 (1.04-1.07)
1942 to 1951	1.52 (1.5-1.55)	0.82 (0.81-0.83)	1.04 (1.03-1.05)

1947 to 1956	1.41 (1.38-1.43)	0.84 (0.83-0.85)	1.02 (1.01-1.04)
1952 to 1961	1.29 (1.27-1.31)	0.87 (0.86-0.88)	1.01 (1-1.02)
1957 to 1966	1.19 (1.17-1.21)	0.91 (0.9-0.91)	1.01 (1-1.02)
1962 to 1971	1.09 (1.08-1.11)	0.95 (0.95-0.96)	1.01 (1-1.02)
1967 to 1976	1 (1-1)	1 (1-1)	1 (1-1)
1972 to 1981	0.9 (0.88-0.92)	1.05 (1.04-1.06)	0.98 (0.96-0.99)
1977 to 1986	0.79 (0.77-0.81)	1.09 (1.07-1.1)	0.93 (0.91-0.95)
1982 to 1991	0.69 (0.67-0.71)	1.13 (1.11-1.14)	0.87 (0.85-0.89)
1987 to 1996	0.58 (0.56-0.61)	1.18 (1.16-1.2)	0.81 (0.79-0.84)
1992 to 2001	0.5 (0.47-0.54)	1.26 (1.23-1.29)	0.78 (0.73-0.82)
1997 to 2006	0.46 (0.41-0.52)	1.33 (1.28-1.37)	

Data in parentheses represent the 95% confidence intervals.

Table S9. The predicted deaths and ASMR per 100,000 population attributable to gynecological cancers from 1990 to 2050 in Asia.

	Cervical cancer		Ovarian cancer		Uterine cancer	
**year**	**Counts**	**ASMR**	**Counts**	**ASMR**	**Counts**	**ASMR**
1990	111129.49 (107877.89-114381.08)	7.08 (7.04-7.12)	31012.8 (29481.93-32543.67)	2 (1.98-2.02)	20289.67 (19126.29-21453.06)	1.49 (1.47-1.51)
1991	111151.2 (107898.65-114403.74)	6.93 (6.89-6.97)	32213.68 (30656.48-33770.89)	2.03 (2.01-2.05)	20680.41 (19513.61-21847.21)	1.49 (1.47-1.5)

1992	112233.43 (108961.42-115505.43)	6.85 (6.82-6.89)	33507.05 (31914.64-35099.45)	2.07 (2.05-2.09)	21101.1 (19921.01-22281.2)	1.49 (1.47-1.5)
1993	112389.79 (109111.98-115667.6)	6.72 (6.68-6.76)	34873.71 (33244.04-36503.39)	2.11 (2.09-2.13)	21508.07 (20314.53-22701.62)	1.48 (1.47-1.5)
1994	113162.36 (109867.82-116456.91)	6.63 (6.59-6.67)	36411.98 (34741.79-38082.16)	2.16 (2.14-2.18)	22030.81 (20821-23240.62)	1.49 (1.47-1.5)
1995	113732.27 (110421.97-117042.57)	6.53 (6.49-6.57)	37805.07 (36097-39513.13)	2.2 (2.19-2.22)	22543.7 (21318.06-23769.35)	1.49 (1.48-1.51)
1996	114539.79 (111216.02-117863.57)	6.44 (6.41-6.48)	39099.95 (37357.88-40842.02)	2.23 (2.21-2.25)	23113.92 (21870.53-24357.31)	1.5 (1.49-1.52)
1997	114987.55 (111655.1-118320)	6.33 (6.29-6.36)	40352.58 (38578.51-42126.65)	2.26 (2.24-2.27)	23650.55 (22390.19-24910.91)	1.5 (1.49-1.52)
1998	115447 (112102.07-118791.92)	6.21 (6.18-6.25)	41499.68 (39696.32-43303.04)	2.27 (2.25-2.29)	24229.92 (22951.62-25508.22)	1.51 (1.49-1.52)
1999	115784.66 (112427.26-119142.07)	6.09 (6.06-6.12)	42591.12 (40759.92-44422.32)	2.28 (2.26-2.3)	24819.02 (23522.81-26115.24)	1.51 (1.5-1.53)
2000	116059.54 (112691.57-119427.51)	5.97 (5.94-6)	43674.54 (41816.17-45532.91)	2.29 (2.27-2.3)	25365.81 (24053.43-26678.2)	1.51 (1.5-1.53)

2001	116612.13 (113231.96-119992.3)	5.86 (5.83-5.9)	44710.18 (42826.56-46593.81)	2.29 (2.27-2.31)	25965.2 (24635.12-27295.29)	1.51 (1.5-1.53)
2002	118783 (115368.33-122197.67)	5.83 (5.8-5.86)	45622.6 (43716.11-47529.09)	2.28 (2.26-2.3)	26540.85 (25193.41-27888.3)	1.51 (1.5-1.52)
2003	120597.86 (117153.38-124042.35)	5.78 (5.75-5.81)	46567.67 (44636.98-48498.37)	2.27 (2.25-2.29)	27148.77 (25783.45-28514.1)	1.51 (1.49-1.52)
2004	121905.6 (118437.95-125373.26)	5.71 (5.68-5.74)	47805.26 (45845.19-49765.34)	2.27 (2.26-2.29)	27736.72 (26354.8-29118.64)	1.5 (1.49-1.52)
2005	122834.31 (119352.35-126316.27)	5.61 (5.58-5.64)	49170.1 (47178.76-51161.44)	2.28 (2.26-2.3)	28118.92 (26726.65-29511.19)	1.49 (1.47-1.5)
2006	123389.2 (119901.43-126876.97)	5.5 (5.47-5.53)	50599.57 (48575.8-52623.35)	2.29 (2.27-2.31)	28377.05 (26977.06-29777.03)	1.46 (1.45-1.48)
2007	124236.2 (120737.41-127734.99)	5.4 (5.37-5.43)	52158.96 (50099.94-54217.99)	2.3 (2.28-2.32)	28763.44 (27351.98-30174.9)	1.44 (1.43-1.46)
2008	125331.05 (121814.3-128847.81)	5.31 (5.28-5.34)	53746.91 (51652.22-55841.59)	2.31 (2.29-2.32)	29204.1 (27780.93-30627.27)	1.43 (1.42-1.44)
2009	125973 (122443.15-129502.84)	5.21 (5.18-5.23)	55358.24 (53228.84-57487.63)	2.31 (2.3-2.33)	29569.68 (28136.73-31002.63)	1.41 (1.4-1.42)

2010	128196.91 (124636.75-131757.07)	5.17 (5.14-5.2)	57137.25 (54970.59-59303.9)	2.33 (2.31-2.34)	30026.05 (28580.71-31471.38)	1.39 (1.38-1.41)
2011	130730.35 (127137.99-134322.71)	5.15 (5.12-5.17)	59062.52 (56854.55-61270.48)	2.34 (2.33-2.36)	30390.36 (28935.16-31845.55)	1.37 (1.36-1.38)
2012	133806.39 (130174.61-137438.16)	5.14 (5.11-5.17)	60728.21 (58485.81-62970.62)	2.35 (2.33-2.36)	30542.14 (29084.06-32000.22)	1.34 (1.33-1.35)
2013	136922.94 (133245.59-140600.29)	5.13 (5.11-5.16)	62454.21 (60177.69-64730.72)	2.35 (2.34-2.37)	30542.21 (29085.12-31999.3)	1.3 (1.29-1.32)
2014	140546 (136824.89-144267.11)	5.14 (5.11-5.17)	64529.38 (62211.7-66847.06)	2.37 (2.35-2.38)	30634.24 (29175.1-32093.39)	1.27 (1.26-1.28)
2015	145769.91 (141989.68-149550.14)	5.21 (5.18-5.23)	66780.95 (64419-69142.89)	2.39 (2.37-2.4)	31164.51 (29692.74-32636.28)	1.26 (1.25-1.27)
2016	150055.31 (146219.24-153891.37)	5.24 (5.21-5.26)	69552.85 (67137.21-71968.49)	2.42 (2.41-2.44)	31989.7 (30497.15-33482.25)	1.26 (1.25-1.27)
2017	154144.24 (150254.25-158034.23)	5.26 (5.23-5.28)	72836.08 (70359.74-75312.41)	2.47 (2.46-2.49)	33074.53 (31553.84-34595.23)	1.27 (1.26-1.28)
2018	156972.42 (153052.55-160892.28)	5.23 (5.2-5.25)	75957.76 (73423.21-78492.3)	2.52 (2.5-2.53)	34304.48 (32752.11-35856.86)	1.28 (1.27-1.29)

2019	158403.63 (154466.11-162341.14)	5.16 (5.13-5.18)	79012.1 (76421.13-81603.08)	2.55 (2.54-2.57)	35486.72 (33904.46-37068.99)	1.29 (1.28-1.3)
2020	159350.89 (155397.64-163304.13)	5.07 (5.05-5.1)	81610 (78970.32-84249.68)	2.58 (2.56-2.59)	36445.59 (34839.02-38052.15)	1.29 (1.28-1.3)
2021	160725.41 (156749.07-164701.75)	5.02 (4.99-5.04)	84321.1 (81624.09-87018.11)	2.6 (2.59-2.62)	37507.75 (35864.21-39151.28)	1.3 (1.28-1.31)
2022	162661.88 (149734.35-175589.41)	4.92 (4.75-5.08)	87399.91 (83134.03-91665.79)	2.61 (2.55-2.67)	38933.67 (36751.51-41115.83)	1.3 (1.27-1.33)
2023	164546.84 (150517.23-178576.46)	4.87 (4.65-5.09)	90329.57 (85338.73-95320.42)	2.64 (2.55-2.73)	40090.73 (37438.84-42742.62)	1.3 (1.25-1.36)
2024	166399.67 (150681.14-182118.19)	4.82 (4.52-5.11)	93364.2 (87252.41-99475.99)	2.66 (2.53-2.79)	41270.64 (37896.74-44644.55)	1.31 (1.22-1.39)
2025	168208.95 (150186.7-186231.2)	4.77 (4.39-5.14)	96487.55 (88859.99-104115.11)	2.69 (2.51-2.87)	42477.73 (38146.25-46809.2)	1.31 (1.2-1.43)
2026	169975.04 (149051.97-190898.11)	4.71 (4.25-5.18)	99674.54 (90151.73-109197.35)	2.71 (2.48-2.95)	43714.45 (38202.77-49226.12)	1.32 (1.16-1.47)
2027	171683.06 (147305.87-196060.26)	4.66 (4.1-5.23)	102911.19 (91126.31-114696.07)	2.74 (2.45-3.03)	44959.25 (38051.29-51867.2)	1.32 (1.13-1.51)

2028	173337.48 (145019.01-201655.95)	4.61 (3.94-5.29)	106241.26 (91842.54-120639.97)	2.77 (2.41-3.12)	46216.28 (37714.41-54718.15)	1.32 (1.09-1.55)
2029	174956.44 (142251.17-207661.72)	4.56 (3.78-5.34)	109694.57 (92329.81-127059.32)	2.79 (2.37-3.22)	47498.71 (37208.08-57789.33)	1.32 (1.04-1.6)
2030	176534.34 (139024.6-214044.08)	4.51 (3.61-5.4)	113253.08 (92564.34-133941.81)	2.82 (2.32-3.32)	48812.99 (36533.27-61092.71)	1.32 (1-1.65)
2031	178092.37 (135362.51-220822.24)	4.45 (3.44-5.46)	116898.35 (92546.44-141250.26)	2.85 (2.27-3.43)	50163.58 (35686.92-64640.23)	1.32 (0.95-1.7)
2032	179614.03 (131271.4-227956.66)	4.4 (3.27-5.53)	120614.04 (92185.44-149042.64)	2.88 (2.21-3.54)	51530.62 (34625.78-68435.46)	1.32 (0.9-1.75)
2033	181105.62 (126775.42-235435.83)	4.34 (3.09-5.6)	124448.42 (91537.98-157358.87)	2.9 (2.15-3.66)	52912.23 (33354.23-72470.23)	1.32 (0.85-1.8)
2034	182581.83 (121893.55-243270.1)	4.29 (2.92-5.66)	128432.85 (90609.18-166256.52)	2.93 (2.08-3.78)	54321.07 (31873.36-76768.77)	1.32 (0.79-1.86)
2035	184029.25 (116614.07-251444.43)	4.23 (2.74-5.73)	132543.14 (89355.93-175730.35)	2.96 (2.01-3.91)	55767.14 (30174.82-81359.45)	1.32 (0.73-1.91)
2036	185452.55 (110928.97-259976.12)	4.18 (2.56-5.8)	136752.84 (87729.23-185776.45)	2.99 (1.94-4.04)	57258.91 (28240.54-86277.49)	1.32 (0.67-1.97)

2037	186833.82 (104815.42-268852.23)	4.12 (2.38-5.87)	141051.71 (85689.68-196413.75)	3.02 (1.86-4.18)	58779.41 (26040.19-91526.05)	1.32 (0.61-2.03)
2038	188161.65 (98273.75-278049.55)	4.06 (2.19-5.93)	145480.93 (83240.59-207721.28)	3.05 (1.77-4.33)	60327.25 (23553.75-97115.99)	1.31 (0.54-2.08)
2039	189462.02 (91313.73-287610.31)	4.01 (2.01-6)	150074.17 (80369.82-219778.52)	3.08 (1.68-4.48)	61917.37 (20766.99-103091.46)	1.31 (0.48-2.14)
2040	190740.1 (83925.91-297557.76)	3.95 (1.83-6.06)	154809.89 (77025.43-232594.34)	3.11 (1.59-4.63)	63563.33 (17669.56-109503.93)	1.31 (0.41-2.2)
2041	192011.98 (76100.57-307942.15)	3.89 (1.65-6.13)	159669.12 (73151.63-246186.62)	3.14 (1.49-4.79)	65279.72 (14213.64-116418.76)	1.3 (0.34-2.26)
2042	193265.94 (67793.06-318773.27)	3.83 (1.47-6.19)	164653.56 (68698.79-260608.34)	3.17 (1.39-4.96)	67058.17 (10363.83-123871.41)	1.3 (0.27-2.32)
2043	194478.28 (58992.83-330023.75)	3.77 (1.29-6.25)	169799.95 (63639.55-275960.34)	3.2 (1.28-5.13)	68903.45 (6078.14-131910.46)	1.29 (0.2-2.39)
2044	195675.01 (49709.59-341757.66)	3.71 (1.11-6.3)	175141.13 (57956.24-292349.69)	3.24 (1.16-5.31)	70832.71 (1367.56-140618.86)	1.29 (0.13-2.45)
2045	196876.51 (39885.58-354043.92)	3.65 (0.94-6.36)	180664.05 (51570.06-309819.72)	3.27 (1.04-5.49)	72856.94 (0-150081.79)	1.28 (0.06-2.51)

2046	198107.87 (29552.6-366974.8)	3.58 (0.76-6.41)	186364.83 (44408.81-328438.35)	3.3 (0.92-5.68)	74988.8 (0-160405.25)	1.28 (-0.02-2.58)
2047	199362.88 (18623.32-380594.05)	3.52 (0.59-6.45)	192262.86 (36420.43-348323.93)	3.33 (0.79-5.88)	77220.24 (0-171669.18)	1.27 (-0.09-2.64)
2048	200611.59 (7192.1-394886.4)	3.46 (0.42-6.5)	198391.4 (27521.02-369619.38)	3.36 (0.65-6.08)	79553.28 (0-183972.05)	1.27 (-0.17-2.7)
2049	201882.54 (0-409958.57)	3.4 (0.26-6.54)	204783.36 (17640.75-392483.92)	3.4 (0.51-6.28)	82011.77 (0-197481.37)	1.26 (-0.24-2.77)
2050	203204.26 (0-425936.96)	3.33 (0.09-6.57)	211430.17 (6769.07-417015.81)	3.43 (0.37-6.49)	84614.43 (0-212380.49)	1.26 (-0.32-2.83)

ASMR= age-standardized mortality rate; Data in parentheses represent the 95% confidence intervals.

Table S10. The predicted deaths and age-specific mortality rates per 100,000 population attributable to gynecological cancers from 2022 to 2050 by age group in Asia.

Year	Age	Counts	Mortality rate
Cervical cancer			
2022	15 to 19	282.49 (235.19-329.79)	0.09 (0.08-0.1)
	20 to 24	779.33 (693.7-864.96)	0.26 (0.24-0.28)

25 to 29	1735.62 (1574.63-1896.6)	0.58 (0.54-0.63)
30 to 34	4049.78 (3709.11-4390.45)	1.33 (1.23-1.43)
35 to 39	7615.62 (7000.81-8230.43)	2.63 (2.44-2.84)
40 to 44	12255.79 (11285.68-13225.91)	4.75 (4.39-5.13)
45 to 49	15875.2 (14629.92-17120.48)	6.57 (6.08-7.09)
50 to 54	20840.84 (19215.88-22465.79)	9.08 (8.41-9.8)
55 to 59	23120.83 (21320.23-24921.43)	11.15 (10.32-12.03)
60 to 64	18992.58 (17509.56-20475.59)	11.23 (10.39-12.11)
65 to 69	17626.84 (16249.97-19003.71)	11.95 (11.06-12.9)
70 to 74	15176.02 (13987.05-16365)	13.32 (12.33-14.37)
75 to 79	10245.38 (9433.7-11057.06)	13.42 (12.41-14.48)
80 to 84	7422.87 (6827.49-8018.26)	14.07 (13.02-15.18)
85 to 89	4501.79 (4130.34-4873.25)	15.07 (13.94-16.26)
90 to 94	1677.35 (1524-1830.71)	13.18 (12.18-14.24)
95 plus	463.54 (407.09-519.99)	11.94 (11-12.93)

	15 to 19	276.51 (225.64-327.38)	0.09 (0.08-0.1)
	20 to 24	759.03 (670.45-847.61)	0.25 (0.23-0.28)
	25 to 29	1689.72 (1523.21-1856.24)	0.57 (0.52-0.62)
	30 to 34	3968.09 (3611.16-4325.02)	1.3 (1.2-1.42)
	35 to 39	7640.29 (6979.18-8301.4)	2.6 (2.39-2.83)
	40 to 44	12348.61 (11298.89-13398.32)	4.7 (4.32-5.1)
	45 to 49	15804.19 (14471.49-17136.9)	6.48 (5.96-7.04)
2023	50 to 54	20822.91 (19077.11-22568.7)	9 (8.27-9.76)
	55 to 59	23362.59 (21407.28-25317.91)	11.03 (10.14-11.97)
	60 to 64	19597.09 (17953.78-21240.4)	11.26 (10.35-12.21)
	65 to 69	17726.31 (16238.09-19214.52)	11.79 (10.85-12.8)
	70 to 74	15633.08 (14317.78-16948.38)	13.19 (12.13-14.31)
	75 to 79	10534.26 (9639.89-11428.62)	13.19 (12.13-14.31)
	80 to 84	7560.45 (6911.43-8209.47)	14.03 (12.9-15.23)
	85 to 89	4584.51 (4181.26-4987.76)	14.95 (13.74-16.23)

	90 to 94	1751.57 (1583.42-1919.72)	13.37 (12.29-14.53)
	95 plus	487.64 (427.18-548.1)	12.02 (11.01-13.09)
	15 to 19	270.98 (215.39-326.57)	0.09 (0.07-0.1)
	20 to 24	740.05 (645.98-834.11)	0.25 (0.22-0.27)
	25 to 29	1645.16 (1468.55-1821.78)	0.55 (0.5-0.61)
	30 to 34	3864.03 (3481.32-4246.73)	1.28 (1.16-1.4)
	35 to 39	7642.19 (6911.05-8373.33)	2.57 (2.34-2.82)
	40 to 44	12502.63 (11324.6-13680.66)	4.65 (4.23-5.09)
2024	45 to 49	15725.13 (14253.06-17197.19)	6.4 (5.82-7.01)
	50 to 54	20753.8 (18820.7-22686.9)	8.91 (8.11-9.76)
	55 to 59	23419.51 (21242.24-25596.79)	10.87 (9.9-11.9)
	60 to 64	20405.46 (18506.08-22304.85)	11.31 (10.3-12.38)
	65 to 69	17733.69 (16080.38-19386.99)	11.65 (10.61-12.75)
	70 to 74	16062.6 (14563.04-17562.16)	13.07 (11.9-14.31)
	75 to 79	10917.21 (9890.69-11943.74)	12.94 (11.78-14.17)

	80 to 84	7691.66 (6961.83-8421.48)	14 (12.75-15.34)
	85 to 89	4661.63 (4210.43-5112.83)	14.79 (13.46-16.2)
	90 to 94	1836.59 (1646.04-2027.14)	13.55 (12.32-14.85)
	95 plus	527.34 (459.75-594.94)	12.13 (11.01-13.33)
	15 to 19	265.73 (204.39-327.07)	0.08 (0.07-0.1)
	20 to 24	722.52 (620.1-824.95)	0.24 (0.21-0.27)
	25 to 29	1602.24 (1410.94-1793.54)	0.54 (0.48-0.6)
	30 to 34	3751.1 (3332.82-4169.37)	1.25 (1.12-1.39)
	35 to 39	7621.27 (6796.29-8446.24)	2.54 (2.28-2.82)
2025	40 to 44	12679.76 (11324.46-14035.05)	4.6 (4.13-5.11)
	45 to 49	15661.78 (13996-17327.55)	6.31 (5.67-7)
	50 to 54	20636.61 (18450.99-22822.22)	8.81 (7.91-9.77)
	55 to 59	23381.66 (20908.78-25854.55)	10.7 (9.61-11.86)
	60 to 64	21255.56 (19005.91-23505.2)	11.34 (10.19-12.58)
	65 to 69	17746.64 (15865.72-19627.56)	11.52 (10.35-12.78)

	70 to 74	16446.57 (14702.26-18190.87)	12.94 (11.63-14.35)
	75 to 79	11362.78 (10151.2-12574.36)	12.73 (11.43-14.11)
	80 to 84	7836.94 (6995.06-8678.81)	13.96 (12.54-15.49)
	85 to 89	4746.78 (4228.98-5264.59)	14.63 (13.14-16.23)
	90 to 94	1921.9 (1700.94-2142.86)	13.67 (12.27-15.18)
	95 plus	569.12 (491.85-646.39)	12.27 (10.99-13.65)
	15 to 19	260.63 (192.69-328.57)	0.08 (0.07-0.1)
	20 to 24	706.23 (592.49-819.98)	0.23 (0.2-0.27)
	25 to 29	1560.6 (1350.41-1770.79)	0.53 (0.46-0.59)
	30 to 34	3640.06 (3177.05-4103.07)	1.23 (1.08-1.38)
2026	35 to 39	7570.11 (6630.7-8509.51)	2.5 (2.21-2.82)
	40 to 44	12840.86 (11263.74-14417.97)	4.56 (4.03-5.14)
	45 to 49	15638.68 (13725.03-17552.34)	6.22 (5.5-7.01)
	50 to 54	20512.65 (18011.32-23013.99)	8.71 (7.69-9.81)
	55 to 59	23341.66 (20498.3-26185.02)	10.55 (9.32-11.89)

	60 to 64	22000.95 (19319.73-24682.18)	11.34 (10.01-12.77)
	65 to 69	17867.88 (15687.65-20048.1)	11.44 (10.1-12.89)
	70 to 74	16755.51 (14710.75-18800.28)	12.8 (11.3-14.41)
	75 to 79	11828.56 (10379.71-13277.42)	12.56 (11.09-14.15)
	80 to 84	8002.13 (7015.96-8988.3)	13.87 (12.25-15.63)
	85 to 89	4844.95 (4241-5448.9)	14.51 (12.82-16.35)
	90 to 94	1996.76 (1737.97-2255.55)	13.73 (12.11-15.47)
	95 plus	606.8 (517.48-696.13)	12.43 (10.95-14.04)
	15 to 19	255.53 (180.31-330.76)	0.08 (0.06-0.1)
	20 to 24	690.94 (563.06-818.82)	0.23 (0.19-0.27)
	25 to 29	1520.17 (1287.43-1752.9)	0.51 (0.44-0.59)
2027	30 to 34	3539.74 (3023.58-4055.9)	1.2 (1.03-1.38)
	35 to 39	7478.18 (6409.51-8546.85)	2.47 (2.13-2.83)
	40 to 44	12951.09 (11115.84-14786.34)	4.52 (3.91-5.19)
	45 to 49	15679.96 (13464.3-17895.63)	6.15 (5.32-7.06)

	50 to 54	20421.98 (17544.21-23299.75)	8.6 (7.45-9.87)
	55 to 59	23341.06 (20055.09-26627.03)	10.44 (9.03-11.98)
	60 to 64	22515.95 (19345.42-25686.49)	11.27 (9.76-12.93)
	65 to 69	18197.75 (15632.77-20762.72)	11.42 (9.88-13.1)
	70 to 74	16987.35 (14592.82-19381.88)	12.64 (10.94-14.5)
	75 to 79	12260.55 (10527.96-13993.13)	12.42 (10.75-14.26)
	80 to 84	8195.62 (7032.15-9359.08)	13.7 (11.86-15.72)
	85 to 89	4955.89 (4246.29-5665.49)	14.44 (12.5-16.58)
	90 to 94	2053.62 (1750.95-2356.3)	13.7 (11.85-15.73)
	95 plus	637.69 (534.19-741.2)	12.62 (10.9-14.51)
	15 to 19	250.48 (167.43-333.54)	0.08 (0.06-0.11)
	20 to 24	676.86 (532.41-821.31)	0.22 (0.18-0.27)
2028	25 to 29	1481.69 (1223.3-1740.08)	0.5 (0.42-0.58)
	30 to 34	3448.76 (2873.07-4024.45)	1.17 (0.99-1.37)
	35 to 39	7332.96 (6128.78-8537.13)	2.43 (2.05-2.84)

	40 to 44	13004.03 (10883.41-15124.64)	4.47 (3.78-5.24)
	45 to 49	15812.49 (13239.69-18385.28)	6.08 (5.14-7.13)
	50 to 54	20346.83 (17043.34-23650.32)	8.5 (7.19-9.96)
	55 to 59	23339.52 (19553.48-27125.57)	10.34 (8.75-12.12)
	60 to 64	22773.75 (19079.39-26468.12)	11.15 (9.43-13.07)
	65 to 69	18805.37 (15752.89-21857.86)	11.45 (9.69-13.42)
	70 to 74	17108.76 (14330.83-19886.7)	12.47 (10.55-14.61)
	75 to 79	12653.8 (10595.68-14711.92)	12.31 (10.41-14.42)
	80 to 84	8460.67 (7080.11-9841.24)	13.47 (11.4-15.79)
	85 to 89	5067.37 (4235.1-5899.64)	14.41 (12.19-16.89)
	90 to 94	2100.19 (1747.69-2452.7)	13.6 (11.49-15.94)
	95 plus	673.93 (552.4-795.46)	12.81 (10.82-15.04)
	15 to 19	245.56 (154.19-336.93)	0.08 (0.05-0.11)
2029	20 to 24	663.93 (500.85-827.02)	0.21 (0.17-0.27)
	25 to 29	1445.93 (1158.96-1732.91)	0.48 (0.4-0.58)

	30 to 34	3360.81 (2721.51-4000.11)	1.14 (0.94-1.37)
	35 to 39	7146.94 (5805.47-8488.41)	2.38 (1.96-2.86)
	40 to 44	13019.97 (10590.45-15449.48)	4.42 (3.64-5.3)
	45 to 49	16026.15 (13041.27-19011.03)	6.03 (4.97-7.23)
	50 to 54	20263.89 (16495.85-24031.94)	8.39 (6.92-10.06)
	55 to 59	23283.29 (18957.08-27609.51)	10.25 (8.46-12.29)
	60 to 64	22853.98 (18607.83-27100.14)	11 (9.07-13.18)
	65 to 69	19613.87 (15968.38-23259.37)	11.52 (9.5-13.8)
	70 to 74	17145.63 (13957.62-20333.65)	12.33 (10.17-14.78)
	75 to 79	13030.19 (10604.53-15455.84)	12.21 (10.07-14.63)
	80 to 84	8803.86 (7161.09-10446.64)	13.24 (10.92-15.87)
	85 to 89	5182.75 (4210.87-6154.64)	14.4 (11.87-17.26)
	90 to 94	2149.14 (1739.45-2558.84)	13.46 (11.1-16.14)
	95 plus	720.53 (575.77-865.29)	12.99 (10.7-15.6)
2030	15 to 19	240.7 (140.66-340.75)	0.07 (0.05-0.11)

20 to 24	651.77 (468.38-835.15)	0.21 (0.16-0.27)
25 to 29	1413.15 (1094.62-1731.69)	0.47 (0.37-0.58)
30 to 34	3276.46 (2570.27-3982.66)	1.11 (0.89-1.37)
35 to 39	6945.02 (5464.6-8425.43)	2.33 (1.87-2.87)
40 to 44	12998.58 (10241.69-15755.47)	4.37 (3.5-5.36)
45 to 49	16272.07 (12826.43-19717.72)	5.98 (4.79-7.34)
50 to 54	20203.93 (15930.97-24476.88)	8.28 (6.65-10.17)
55 to 59	23175.9 (18277.69-28074.11)	10.15 (8.14-12.47)
60 to 64	22844.21 (18016.23-27672.19)	10.84 (8.7-13.31)
65 to 69	20466.27 (16139.92-24792.62)	11.57 (9.28-14.21)
70 to 74	17191.77 (13556.27-20827.27)	12.21 (9.8-15)
75 to 79	13373.16 (10542.94-16203.37)	12.1 (9.71-14.86)
80 to 84	9196.85 (7247.09-11146.6)	13.03 (10.46-16)
85 to 89	5313.31 (4182.49-6444.12)	14.37 (11.53-17.65)
90 to 94	2203.44 (1728.56-2678.31)	13.33 (10.69-16.38)

	95 plus	767.76 (595.76-939.75)	13.13 (10.52-16.14)
2031	15 to 19	235.9 (126.86-344.94)	0.07 (0.05-0.11)
	20 to 24	640.09 (435.02-845.16)	0.2 (0.15-0.27)
	25 to 29	1382.99 (1029.77-1736.2)	0.46 (0.35-0.58)
	30 to 34	3195.11 (2419.27-3970.94)	1.08 (0.84-1.36)
	35 to 39	6747.28 (5124.37-8370.19)	2.29 (1.78-2.88)
	40 to 44	12927.38 (9831.46-16023.3)	4.31 (3.36-5.44)
	45 to 49	16500.49 (12554.45-20446.52)	5.93 (4.62-7.47)
	50 to 54	20199.56 (15373.5-25025.62)	8.18 (6.37-10.31)
	55 to 59	23064.63 (17557.31-28571.95)	10.04 (7.82-12.66)
	60 to 64	22835.35 (17382.89-28287.82)	10.7 (8.34-13.49)
	65 to 69	21221.26 (16153.45-26289.07)	11.57 (9.02-14.59)
	70 to 74	17347.13 (13203.11-21491.15)	12.14 (9.46-15.3)
	75 to 79	13657.63 (10393.31-16921.95)	11.98 (9.33-15.1)
	80 to 84	9604.31 (7305.91-11902.71)	12.87 (10.03-16.22)

	85 to 89	5460.66 (4149.79-6771.54)	14.29 (11.13-18.01)
	90 to 94	2263.63 (1714.96-2812.31)	13.24 (10.31-16.7)
	95 plus	808.98 (607.08-1010.87)	13.19 (10.26-16.65)
2032	15 to 19	231.29 (112.95-349.63)	0.07 (0.04-0.11)
	20 to 24	628.41 (400.67-856.14)	0.2 (0.14-0.28)
	25 to 29	1354.82 (963.93-1745.7)	0.44 (0.33-0.58)
	30 to 34	3116.35 (2268.74-3963.95)	1.05 (0.79-1.36)
	35 to 39	6569.7 (4798.21-8341.18)	2.24 (1.69-2.9)
	40 to 44	12787.28 (9352.07-16222.49)	4.25 (3.21-5.51)
	45 to 49	16665.44 (12194.12-21136.75)	5.88 (4.44-7.62)
	50 to 54	20281.14 (14843.89-25718.39)	8.09 (6.11-10.48)
	55 to 59	22993.33 (16832.1-29154.57)	9.94 (7.5-12.87)
	60 to 64	22866.61 (16739.76-28993.45)	10.6 (8-13.73)
	65 to 69	21755.09 (15925.51-27584.67)	11.52 (8.7-14.92)
	70 to 74	17708.26 (12961.78-22454.74)	12.13 (9.16-15.71)

	75 to 79	13879.31 (10157.62-17600.99)	11.84 (8.94-15.34)
	80 to 84	9981.02 (7302.23-12659.81)	12.75 (9.63-16.52)
	85 to 89	5627.24 (4113.26-7141.22)	14.13 (10.67-18.31)
	90 to 94	2328.52 (1697.3-2959.73)	13.19 (9.96-17.09)
	95 plus	840.26 (607.27-1073.25)	13.18 (9.94-17.09)
2033	15 to 19	226.03 (98.55-353.51)	0.07 (0.04-0.11)
	20 to 24	616.88 (365.62-868.14)	0.19 (0.13-0.28)
	25 to 29	1329.12 (897.57-1760.67)	0.43 (0.31-0.59)
	30 to 34	3041.81 (2120.13-3963.48)	1.02 (0.75-1.37)
	35 to 39	6409.91 (4484.52-8335.3)	2.18 (1.59-2.91)
	40 to 44	12556.77 (8796.99-16316.54)	4.19 (3.06-5.58)
	45 to 49	16758.99 (11746.92-21771.07)	5.82 (4.25-7.76)
	50 to 54	20484.51 (14362.2-26606.82)	8.02 (5.86-10.69)
	55 to 59	22942.23 (16088.28-29796.18)	9.83 (7.18-13.1)
	60 to 64	22899.25 (16058.89-29739.61)	10.52 (7.68-14.02)

	65 to 69	22043.09 (15458.25-28627.93)	11.41 (8.34-15.21)
	70 to 74	18345.14 (12863.99-23826.3)	12.18 (8.9-16.24)
	75 to 79	14012.7 (9824.27-18201.13)	11.71 (8.55-15.6)
	80 to 84	10329.06 (7239.62-13418.51)	12.65 (9.24-16.86)
	85 to 89	5845.03 (4093.51-7596.55)	13.92 (10.16-18.55)
	90 to 94	2395.35 (1673.25-3117.44)	13.18 (9.62-17.57)
	95 plus	869.76 (602.86-1136.66)	13.1 (9.56-17.47)
2034	15 to 19	220.83 (84.08-357.58)	0.07 (0.04-0.12)
	20 to 24	605.71 (330.1-881.31)	0.19 (0.12-0.29)
	25 to 29	1305.79 (830.7-1780.88)	0.42 (0.29-0.59)
	30 to 34	2973.03 (1974.29-3971.77)	1 (0.7-1.37)
	35 to 39	6256.11 (4175.02-8337.19)	2.13 (1.5-2.93)
	40 to 44	12256.95 (8190.85-16323.05)	4.11 (2.9-5.65)
	45 to 49	16807.23 (11237.82-22376.64)	5.77 (4.07-7.91)
	50 to 54	20797.01 (13909.43-27684.59)	7.96 (5.61-10.92)

	55 to 59	22884.96 (15308.51-30461.4)	9.72 (6.85-13.33)
	60 to 64	22881.2 (15306.93-30455.46)	10.44 (7.37-14.33)
	65 to 69	22163.04 (14826.51-29499.57)	11.27 (7.95-15.47)
	70 to 74	19184.93 (12833.53-25536.34)	12.27 (8.65-16.84)
	75 to 79	14081.96 (9418.1-18745.81)	11.59 (8.17-15.9)
	80 to 84	10670.79 (7134.94-14206.63)	12.56 (8.86-17.24)
	85 to 89	6119.64 (4088.9-8150.37)	13.69 (9.66-18.79)
	90 to 94	2468.38 (1645.34-3291.41)	13.19 (9.3-18.1)
	95 plus	904.29 (598.5-1210.08)	12.99 (9.15-17.84)
2035	15 to 19	215.83 (69.61-362.05)	0.07 (0.03-0.12)
	20 to 24	594.74 (294.09-895.38)	0.18 (0.11-0.29)
	25 to 29	1284.04 (762.88-1805.21)	0.41 (0.27-0.6)
	30 to 34	2910.55 (1830.86-3990.24)	0.97 (0.66-1.38)
	35 to 39	6109.36 (3870.56-8348.16)	2.08 (1.41-2.95)
	40 to 44	11930.32 (7568.87-16291.77)	4.04 (2.75-5.72)

	45 to 49	16809.32 (10670.69-22947.95)	5.71 (3.88-8.07)
	50 to 54	21155.3 (13433.57-28877.03)	7.9 (5.37-11.18)
	55 to 59	22856.66 (14516.25-31197.07)	9.61 (6.53-13.59)
	60 to 64	22815.83 (14491.43-31140.22)	10.36 (7.04-14.65)
	65 to 69	22198.69 (14099.43-30297.94)	11.13 (7.57-15.74)
	70 to 74	20073.51 (12749.12-27397.91)	12.34 (8.39-17.46)
	75 to 79	14163.99 (8994.03-19333.94)	11.5 (7.82-16.26)
	80 to 84	10989.62 (6976.89-15002.35)	12.48 (8.48-17.65)
	85 to 89	6427.94 (4078.19-8777.69)	13.5 (9.18-19.1)
	90 to 94	2551.79 (1615.34-3488.23)	13.19 (8.96-18.65)
	95 plus	941.76 (592.26-1291.27)	12.89 (8.76-18.24)
	15 to 19	211.13 (55.16-367.09)	0.07 (0.03-0.12)
2036	20 to 24	583.84 (257.6-910.08)	0.18 (0.1-0.3)
	25 to 29	1263.17 (693.76-1832.59)	0.4 (0.25-0.61)
	30 to 34	2853.44 (1688.31-4018.56)	0.95 (0.62-1.39)

	35 to 39	5968.28 (3569.94-8366.62)	2.03 (1.33-2.96)
	40 to 44	11610.99 (6955.34-16266.65)	3.96 (2.59-5.79)
	45 to 49	16748.12 (10039.22-23457.01)	5.64 (3.69-8.24)
	50 to 54	21493.84 (12888.12-30099.56)	7.85 (5.14-11.46)
	55 to 59	22894.06 (13729.81-32058.3)	9.51 (6.22-13.87)
	60 to 64	22748.76 (13643.91-31853.61)	10.26 (6.72-14.97)
	65 to 69	22236.8 (13336.86-31136.74)	11.01 (7.21-16.06)
	70 to 74	20870.32 (12516.85-29223.79)	12.37 (8.1-18.05)
	75 to 79	14340.1 (8598.59-20081.6)	11.45 (7.49-16.7)
	80 to 84	11261.4 (6751.39-15771.4)	12.37 (8.1-18.05)
	85 to 89	6742.74 (4040.02-9445.46)	13.36 (8.75-19.5)
	90 to 94	2644.68 (1581.2-3708.15)	13.14 (8.6-19.17)
	95 plus	980.91 (582.89-1378.94)	12.82 (8.38-18.72)
2037	15 to 19	206.59 (40.66-372.52)	0.06 (0.03-0.13)
	20 to 24	573.55 (220.88-926.22)	0.18 (0.09-0.31)

25 to 29	1242.53 (623.1-1861.96)	0.39 (0.23-0.62)
30 to 34	2800.76 (1545.27-4056.25)	0.92 (0.58-1.4)
35 to 39	5832.47 (3272.56-8392.39)	1.98 (1.24-2.98)
40 to 44	11327.21 (6366.4-16288.03)	3.89 (2.44-5.85)
45 to 49	16599.28 (9335.94-23862.63)	5.58 (3.51-8.4)
50 to 54	21753.51 (12239.29-31267.74)	7.8 (4.91-11.75)
55 to 59	23033.69 (12961.51-33105.88)	9.42 (5.93-14.19)
60 to 64	22724.6 (12788.84-32660.35)	10.17 (6.4-15.32)
65 to 69	22316.2 (12559.25-32073.16)	10.92 (6.87-16.45)
70 to 74	21452.89 (12073.01-30832.76)	12.34 (7.76-18.58)
75 to 79	14690.59 (8265.71-21115.46)	11.46 (7.21-17.26)
80 to 84	11480.56 (6458.52-16502.6)	12.25 (7.71-18.46)
85 to 89	7031.75 (3953.69-10109.81)	13.26 (8.34-19.98)
90 to 94	2746.85 (1541.31-3952.39)	13.02 (8.19-19.61)
95 plus	1020.79 (569.48-1472.1)	12.8 (8.04-19.29)

	15 to 19	202.94 (26.18-379.71)	0.06 (0.03-0.13)
	20 to 24	561.67 (183.15-940.19)	0.17 (0.09-0.31)
	25 to 29	1222.27 (551.11-1893.44)	0.38 (0.22-0.63)
	30 to 34	2753.35 (1401.76-4104.94)	0.9 (0.54-1.42)
	35 to 39	5704.79 (2979.77-8429.82)	1.93 (1.16-3)
	40 to 44	11074.64 (5797.66-16351.63)	3.8 (2.29-5.92)
	45 to 49	16333.8 (8556.87-24110.73)	5.5 (3.32-8.56)
2038	50 to 54	21923.54 (11489.99-32357.1)	7.74 (4.67-12.05)
	55 to 59	23317.04 (12222.24-34411.84)	9.35 (5.65-14.56)
	60 to 64	22723.11 (11912.15-33534.07)	10.08 (6.08-15.69)
	65 to 69	22399.41 (11742.94-33055.88)	10.86 (6.56-16.9)
	70 to 74	21796.15 (11426.49-32165.81)	12.25 (7.39-19.06)
	75 to 79	15276.1 (8006.95-22545.26)	11.54 (6.96-17.95)
	80 to 84	11630.11 (6094.73-17165.5)	12.14 (7.33-18.89)
	85 to 89	7303.14 (3825.29-10780.98)	13.19 (7.96-20.52)

	90 to 94	2875.81 (1503.43-4248.19)	12.84 (7.75-19.99)
	95 plus	1063.75 (553.04-1574.47)	12.81 (7.73-19.95)
	15 to 19	199.47 (11.49-387.44)	0.06 (0.02-0.14)
	20 to 24	549.98 (145.2-954.75)	0.17 (0.08-0.32)
	25 to 29	1202.78 (477.99-1927.57)	0.38 (0.2-0.65)
	30 to 34	2711.02 (1257.19-4164.85)	0.88 (0.5-1.44)
	35 to 39	5588.15 (2692-8484.31)	1.88 (1.09-3.03)
	40 to 44	10832.85 (5236.66-16429.03)	3.72 (2.15-5.99)
2039	45 to 49	15978.63 (7729.72-24227.54)	5.42 (3.13-8.72)
	50 to 54	22037.96 (10666.1-33409.82)	7.68 (4.44-12.36)
	55 to 59	23730.49 (11487.31-35973.67)	9.3 (5.38-14.96)
	60 to 64	22718.46 (10998.55-34438.36)	9.99 (5.78-16.07)
	65 to 69	22436.55 (10862.71-34010.4)	10.81 (6.25-17.38)
	70 to 74	21977.28 (10640.33-33314.22)	12.12 (7.01-19.5)
	75 to 79	16037.94 (7763.56-24312.31)	11.64 (6.73-18.73)

	80 to 84	11735.19 (5679.45-17790.92)	12.04 (6.96-19.37)
	85 to 89	7577.41 (3665.5-11489.32)	13.12 (7.59-21.11)
	90 to 94	3034.67 (1465.33-4604)	12.66 (7.32-20.37)
	95 plus	1113.23 (534.66-1691.8)	12.85 (7.42-20.68)
	15 to 19	196.14 (0-395.75)	0.06 (0.02-0.14)
	20 to 24	538.79 (107.09-970.49)	0.17 (0.07-0.33)
	25 to 29	1183.77 (403.61-1963.93)	0.37 (0.18-0.66)
	30 to 34	2672.14 (1110.31-4233.98)	0.86 (0.46-1.46)
	35 to 39	5483.56 (2407.82-8559.31)	1.84 (1.01-3.06)
2040	40 to 44	10603.61 (4683.34-16523.88)	3.64 (2.01-6.06)
	45 to 49	15588.8 (6890.39-24287.22)	5.33 (2.95-8.88)
	50 to 54	22094.87 (9771.6-34418.14)	7.62 (4.21-12.68)
	55 to 59	24201.81 (10705.66-37697.95)	9.26 (5.12-15.4)
	60 to 64	22746.11 (10062.77-35429.44)	9.9 (5.48-16.47)
	65 to 69	22430.75 (9924.03-34937.47)	10.74 (5.94-17.86)

	70 to 74	22077.67 (9767.81-34387.53)	11.99 (6.63-19.95)
	75 to 79	16845.69 (7451.94-26239.44)	11.74 (6.49-19.53)
	80 to 84	11858.5 (5244.55-18472.46)	11.97 (6.62-19.91)
	85 to 89	7839.97 (3465.76-12214.19)	13.06 (7.22-21.72)
	90 to 94	3209.39 (1416.29-5002.5)	12.52 (6.92-20.82)
	95 plus	1168.51 (512.94-1824.08)	12.87 (7.11-21.43)
	15 to 19	193.02 (0-404.79)	0.06 (0.02-0.15)
	20 to 24	528.34 (68.74-987.94)	0.16 (0.07-0.34)
	25 to 29	1164.96 (327.85-2002.06)	0.36 (0.17-0.68)
	30 to 34	2635.24 (960.16-4310.33)	0.84 (0.43-1.49)
2041	35 to 39	5389.33 (2124.17-8654.49)	1.8 (0.95-3.1)
	40 to 44	10384.53 (4135.1-16633.95)	3.56 (1.88-6.13)
	45 to 49	15208.81 (6061.35-24356.27)	5.25 (2.77-9.03)
	50 to 54	22070.88 (8801.7-35340.06)	7.56 (3.99-13.01)
	55 to 59	24655.72 (9835.07-39476.36)	9.22 (4.87-15.87)

	60 to 64	22843.35 (9113.08-36573.62)	9.82 (5.19-16.9)
	65 to 69	22426.59 (8947.65-35905.52)	10.67 (5.63-18.36)
	70 to 74	22182.8 (8850.45-35515.15)	11.89 (6.28-20.47)
	75 to 79	17579.19 (7012.66-28145.72)	11.79 (6.23-20.3)
	80 to 84	12066.22 (4812.22-19320.22)	11.95 (6.31-20.57)
	85 to 89	8070.88 (3217.43-12924.34)	12.98 (6.86-22.34)
	90 to 94	3384.95 (1347.13-5422.78)	12.41 (6.56-21.37)
	95 plus	1227.17 (485.81-1968.53)	12.86 (6.79-22.14)
	15 to 19	190.12 (0-414.7)	0.06 (0.02-0.15)
	20 to 24	518.33 (29.97-1006.69)	0.16 (0.06-0.35)
	25 to 29	1147.43 (250.93-2043.93)	0.36 (0.16-0.7)
2042	30 to 34	2598.97 (806.08-4391.87)	0.83 (0.4-1.52)
	35 to 39	5303.72 (1838.18-8769.27)	1.76 (0.88-3.15)
	40 to 44	10174.9 (3590.08-16759.72)	3.48 (1.75-6.2)
	45 to 49	14875.84 (5254.75-24496.92)	5.15 (2.59-9.19)

	50 to 54	21932.86 (7752.77-36112.96)	7.49 (3.77-13.34)
	55 to 59	25023.72 (8848.25-41199.2)	9.19 (4.63-16.37)
	60 to 64	23047.87 (8150.5-37945.24)	9.76 (4.91-17.39)
	65 to 69	22467.92 (7946.25-36989.59)	10.6 (5.34-18.89)
	70 to 74	22331.26 (7898.18-36764.34)	11.83 (5.96-21.08)
	75 to 79	18133.16 (6412.37-29853.96)	11.79 (5.94-21.01)
	80 to 84	12423.91 (4392.26-20455.57)	12 (6.04-21.37)
	85 to 89	8263.93 (2920.27-13607.58)	12.89 (6.49-22.97)
	90 to 94	3544.62 (1250.49-5838.76)	12.35 (6.22-22.01)
	95 plus	1287.35 (451.73-2122.97)	12.77 (6.43-22.76)
	15 to 19	187.49 (0-425.65)	0.06 (0.02-0.16)
	20 to 24	510.59 (0-1030.55)	0.16 (0.05-0.37)
2043	25 to 29	1126.75 (171.88-2081.62)	0.35 (0.14-0.72)
	30 to 34	2563.66 (648.02-4479.31)	0.81 (0.37-1.55)
	35 to 39	5228.37 (1548.77-8907.98)	1.72 (0.82-3.2)

	40 to 44	9979.72 (3048.65-16910.8)	3.4 (1.63-6.28)
	45 to 49	14584.33 (4463.75-24704.91)	5.06 (2.43-9.34)
	50 to 54	21641.38 (6628.26-36654.5)	7.41 (3.55-13.66)
	55 to 59	25293.29 (7750.12-42836.46)	9.14 (4.38-16.87)
	60 to 64	23402.73 (7171.78-39633.67)	9.71 (4.66-17.92)
	65 to 69	22534.63 (6906.58-38162.68)	10.54 (5.05-19.44)
	70 to 74	22486.41 (6892.26-38080.55)	11.8 (5.66-21.77)
	75 to 79	18488.6 (5666-31311.2)	11.74 (5.63-21.66)
	80 to 84	12984.72 (3978.23-21991.21)	12.11 (5.8-22.34)
	85 to 89	8411.1 (2575.67-14246.52)	12.8 (6.14-23.62)
	90 to 94	3697.21 (1130.22-6264.21)	12.32 (5.9-22.72)
	95 plus	1357.29 (412.64-2301.94)	12.64 (6.05-23.32)
	15 to 19	185.17 (0-437.87)	0.06 (0.01-0.16)
2044	20 to 24	503.29 (0-1056.29)	0.16 (0.05-0.38)
	25 to 29	1106.47 (91.59-2121.35)	0.34 (0.13-0.74)

	30 to 34	2530.1 (485.93-4574.27)	0.8 (0.34-1.59)
	35 to 39	5162.95 (1253.93-9071.97)	1.69 (0.76-3.26)
	40 to 44	9804.15 (2509.51-17098.8)	3.33 (1.51-6.36)
	45 to 49	14307.36 (3675.96-24938.75)	4.97 (2.26-9.49)
	50 to 54	21231.49 (5458.94-37004.04)	7.32 (3.33-13.98)
	55 to 59	25503.52 (6561.11-44445.92)	9.1 (4.15-17.39)
	60 to 64	23894.99 (6148.4-41641.57)	9.69 (4.41-18.51)
	65 to 69	22601.48 (5816.36-39386.6)	10.47 (4.77-20.01)
	70 to 74	22599.23 (5816.37-39382.09)	11.77 (5.36-22.49)
	75 to 79	18712.92 (4815.4-32610.44)	11.65 (5.31-22.27)
	80 to 84	13700.77 (3524.64-23876.9)	12.25 (5.58-23.42)
	85 to 89	8535.19 (2194.48-14875.9)	12.74 (5.8-24.34)
	90 to 94	3856.38 (989.67-6723.08)	12.29 (5.6-23.49)
	95 plus	1439.55 (367.29-2511.81)	12.49 (5.69-23.89)
2045	15 to 19	183.19 (0-451.61)	0.06 (0.01-0.17)

20 to 24	496.41 (0-1084.05)	0.16 (0.05-0.39)
25 to 29	1087.24 (9.94-2164.55)	0.34 (0.12-0.76)
30 to 34	2497.69 (319.26-4676.12)	0.78 (0.31-1.63)
35 to 39	5104.43 (951.1-9257.77)	1.65 (0.7-3.32)
40 to 44	9650.08 (1968.78-17331.37)	3.26 (1.41-6.46)
45 to 49	14047.31 (2889.47-25205.15)	4.87 (2.11-9.65)
50 to 54	20775.75 (4277.02-37274.49)	7.22 (3.12-14.3)
55 to 59	25651.18 (5284.73-46017.63)	9.05 (3.91-17.92)
60 to 64	24451.98 (5039.01-43864.95)	9.67 (4.18-19.15)
65 to 69	22704.54 (4679.66-40729.42)	10.41 (4.5-20.61)
70 to 74	22672.48 (4673.72-40671.24)	11.73 (5.07-23.23)
75 to 79	18873.12 (3889.84-33856.4)	11.56 (5-22.89)
80 to 84	14459.09 (2979.12-25939.06)	12.39 (5.36-24.53)
85 to 89	8680.1 (1787.24-15572.95)	12.7 (5.49-25.15)
90 to 94	4012.87 (824.53-7201.22)	12.27 (5.31-24.29)

	95 plus	1529.04 (312.15-2745.94)	12.39 (5.35-24.53)
2046	15 to 19	181.58 (0-467.11)	0.06 (0.01-0.18)
	20 to 24	490.05 (0-1114.33)	0.15 (0.04-0.41)
	25 to 29	1069.53 (0-2212.51)	0.33 (0.11-0.79)
	30 to 34	2465.84 (147.55-4784.13)	0.77 (0.29-1.67)
	35 to 39	5050.02 (638.21-9461.84)	1.62 (0.65-3.39)
	40 to 44	9514.63 (1420.7-17608.56)	3.2 (1.31-6.57)
	45 to 49	13801.06 (2100.28-25501.84)	4.78 (1.96-9.8)
	50 to 54	20333.33 (3097.86-37568.8)	7.12 (2.92-14.62)
	55 to 59	25708.06 (3920.67-47495.45)	9 (3.69-18.48)
	60 to 64	24997.42 (3813.97-46180.88)	9.66 (3.96-19.83)
	65 to 69	22882.02 (3491.94-42272.11)	10.36 (4.24-21.25)
	70 to 74	22750.92 (3472.59-42029.24)	11.69 (4.79-23.99)
	75 to 79	19040.18 (2905.65-35174.7)	11.5 (4.71-23.6)
	80 to 84	15155.23 (2311.76-27998.7)	12.49 (5.12-25.62)

	85 to 89	8891.95 (1355.23-16428.66)	12.72 (5.21-26.1)
	90 to 94	4154.93 (631.65-7678.21)	12.23 (5.01-25.1)
	95 plus	1621.13 (244.54-2997.71)	12.32 (5.05-25.29)
2047	15 to 19	180.33 (0-484.59)	0.06 (0.01-0.19)
	20 to 24	484.31 (0-1147.63)	0.15 (0.04-0.43)
	25 to 29	1052.73 (0-2264.49)	0.33 (0.1-0.82)
	30 to 34	2436.84 (0-4903.31)	0.76 (0.26-1.72)
	35 to 39	4997.15 (313.73-9680.56)	1.6 (0.6-3.47)
	40 to 44	9394.87 (859.7-17930.03)	3.14 (1.21-6.69)
	45 to 49	13567.74 (1304.77-25830.72)	4.68 (1.82-9.96)
	50 to 54	19954.24 (1923.21-37985.28)	7.02 (2.72-14.94)
	55 to 59	25634.08 (2474-48794.16)	8.95 (3.47-19.03)
	60 to 64	25461.14 (2459.4-48462.89)	9.66 (3.74-20.54)
	65 to 69	23172.91 (2239.08-44106.74)	10.32 (4-21.96)
	70 to 74	22879.18 (2211.32-43547.05)	11.66 (4.52-24.79)

	75 to 79	19245.3 (1859.75-36630.84)	11.48 (4.45-24.42)
	80 to 84	15697.64 (1515.87-29879.41)	12.53 (4.86-26.64)
	85 to 89	9216.3 (888.89-17543.71)	12.81 (4.97-27.24)
	90 to 94	4277.92 (411.07-8144.77)	12.19 (4.72-25.92)
	95 plus	1710.19 (162.52-3257.86)	12.3 (4.77-26.17)
2048	15 to 19	179.44 (0-504.29)	0.06 (0.01-0.19)
	20 to 24	479.26 (0-1184.51)	0.15 (0.03-0.44)
	25 to 29	1040.59 (0-2329.26)	0.33 (0.09-0.85)
	30 to 34	2401.24 (0-5014.95)	0.75 (0.24-1.78)
	35 to 39	4946.45 (0-9916.26)	1.57 (0.55-3.55)
	40 to 44	9293.82 (281.51-18306.13)	3.08 (1.12-6.83)
	45 to 49	13354.11 (500.15-26208.06)	4.59 (1.68-10.13)
	50 to 54	19631.41 (742.2-38520.63)	6.92 (2.53-15.25)
	55 to 59	25381.44 (962.08-49800.79)	8.88 (3.25-19.58)
	60 to 64	25830.95 (981.68-50680.22)	9.65 (3.53-21.27)

	65 to 69	23622.21 (898.51-46345.9)	10.31 (3.78-22.74)
	70 to 74	23037.12 (876.87-45197.38)	11.63 (4.26-25.63)
	75 to 79	19458.18 (740.49-38175.87)	11.49 (4.21-25.32)
	80 to 84	16075.33 (610.77-31539.89)	12.51 (4.58-27.59)
	85 to 89	9694.57 (367.3-19021.85)	12.97 (4.75-28.6)
	90 to 94	4380.63 (164.5-8596.77)	12.15 (4.45-26.78)
	95 plus	1804.84 (66.05-3543.64)	12.31 (4.51-27.14)
2049	15 to 19	178.93 (0-526.55)	0.06 (0.01-0.2)
	20 to 24	475.04 (0-1225.74)	0.15 (0.03-0.46)
	25 to 29	1029.41 (0-2399.77)	0.32 (0.08-0.88)
	30 to 34	2366.57 (0-5134.25)	0.74 (0.22-1.84)
	35 to 39	4899.46 (0-10173.45)	1.55 (0.51-3.65)
	40 to 44	9211.06 (0-18741.29)	3.03 (1.04-6.97)
	45 to 49	13167.17 (0-26653.32)	4.51 (1.56-10.32)
	50 to 54	19328.75 (0-39112.76)	6.81 (2.35-15.57)

	55 to 59	24989.95 (0-50566.88)	8.81 (3.04-20.13)
	60 to 64	26146.94 (0-52905.1)	9.63 (3.33-22.01)
	65 to 69	24218.06 (0-49001.32)	10.32 (3.56-23.58)
	70 to 74	23199.59 (0-46939.97)	11.59 (4-26.5)
	75 to 79	19639.12 (0-39736.07)	11.5 (3.97-26.28)
	80 to 84	16349.51 (0-33080.99)	12.46 (4.31-28.49)
	85 to 89	10293.76 (0-20829.05)	13.18 (4.55-30.12)
	90 to 94	4477.85 (0-9062.16)	12.13 (4.19-27.72)
	95 plus	1911.39 (0-3869.9)	12.33 (4.26-28.18)
2050	15 to 19	178.81 (0-551.78)	0.06 (0.01-0.21)
	20 to 24	471.74 (0-1272.15)	0.15 (0.03-0.48)
	25 to 29	1019.14 (0-2476.55)	0.32 (0.07-0.91)
	30 to 34	2334.21 (0-5265.08)	0.73 (0.2-1.9)
	35 to 39	4855.06 (0-10451.61)	1.53 (0.47-3.75)
	40 to 44	9141.32 (0-19230.17)	2.98 (0.96-7.13)

	45 to 49	13009.68 (0-27180.02)	4.44 (1.44-10.52)
	50 to 54	19049.55 (0-39774.35)	6.71 (2.18-15.89)
	55 to 59	24544.3 (0-51246.03)	8.72 (2.84-20.67)
	60 to 64	26404.07 (0-55125.94)	9.62 (3.13-22.79)
	65 to 69	24887.51 (0-51958.49)	10.34 (3.36-24.5)
	70 to 74	23404.17 (0-48861.04)	11.57 (3.76-27.4)
	75 to 79	19790.31 (0-41316.34)	11.51 (3.74-27.26)
	80 to 84	16574.25 (0-34602.85)	12.42 (4.04-29.42)
	85 to 89	10927.47 (0-22814.88)	13.37 (4.35-31.69)
	90 to 94	4591.02 (0-9586.64)	12.14 (3.95-28.76)
	95 plus	2021.64 (0-4223.05)	12.35 (4.01-29.27)
Ovarian cancer			
	15 to 19	404.02 (352.31-455.73)	0.13 (0.12-0.14)
2022	20 to 24	582.35 (526.05-638.65)	0.19 (0.18-0.21)
	25 to 29	797.48 (730.32-864.64)	0.27 (0.26-0.28)

	30 to 34	1305.6 (1213.5-1397.69)	0.43 (0.41-0.45)
	35 to 39	2165.66 (2034.6-2296.73)	0.75 (0.72-0.78)
	40 to 44	3776.67 (3576.52-3976.82)	1.46 (1.4-1.53)
	45 to 49	6507.46 (6194.31-6820.62)	2.69 (2.58-2.81)
	50 to 54	10074.4 (9616-10532.79)	4.39 (4.21-4.57)
	55 to 59	11784.78 (11258.07-12311.49)	5.69 (5.46-5.92)
	60 to 64	11299.15 (10793.38-11804.92)	6.68 (6.41-6.96)
	65 to 69	11580.12 (11063.88-12096.37)	7.85 (7.54-8.18)
	70 to 74	10026.04 (9573.28-10478.81)	8.8 (8.45-9.16)
	75 to 79	7013.62 (6683.45-7343.78)	9.18 (8.81-9.56)
	80 to 84	5138.29 (4884.75-5391.83)	9.74 (9.35-10.15)
	85 to 89	3097.92 (2929-3266.83)	10.37 (9.94-10.81)
	90 to 94	1403.55 (1308.34-1498.76)	11.03 (10.56-11.51)
	95 plus	442.81 (396.28-489.34)	11.4 (10.86-11.97)
2023	15 to 19	408.97 (351.75-466.19)	0.13 (0.12-0.15)

20 to 24	587.31 (527.8-646.81)	0.2 (0.18-0.21)
25 to 29	803.55 (732.56-874.54)	0.27 (0.26-0.29)
30 to 34	1321.8 (1222.39-1421.2)	0.43 (0.41-0.46)
35 to 39	2226.94 (2080.76-2373.11)	0.76 (0.72-0.8)
40 to 44	3885.92 (3657.15-4114.69)	1.48 (1.4-1.55)
45 to 49	6606.84 (6245.76-6967.91)	2.71 (2.58-2.85)
50 to 54	10287.68 (9749.44-10825.92)	4.44 (4.23-4.66)
55 to 59	12178.35 (11550.44-12806.27)	5.75 (5.47-6.03)
60 to 64	11742.34 (11136.61-12348.06)	6.74 (6.42-7.07)
65 to 69	11883.69 (11272.05-12495.32)	7.91 (7.53-8.29)
70 to 74	10521.3 (9975.6-11066.99)	8.88 (8.45-9.31)
75 to 79	7366.01 (6972.28-7759.73)	9.22 (8.78-9.67)
80 to 84	5342.88 (5046.7-5639.07)	9.92 (9.44-10.4)
85 to 89	3219.07 (3026.09-3412.04)	10.5 (9.99-11.02)
90 to 94	1476.96 (1371.12-1582.79)	11.28 (10.72-11.85)

	95 plus	470 (420.24-519.76)	11.59 (10.96-12.24)
2024	15 to 19	414.42 (349.81-479.02)	0.13 (0.12-0.15)
	20 to 24	592.75 (528.03-657.47)	0.2 (0.18-0.21)
	25 to 29	809.67 (732.51-886.83)	0.27 (0.26-0.29)
	30 to 34	1331.53 (1220.83-1442.24)	0.44 (0.41-0.47)
	35 to 39	2287.49 (2117.62-2457.37)	0.77 (0.72-0.82)
	40 to 44	4015.54 (3740.98-4290.11)	1.49 (1.4-1.59)
	45 to 49	6708.51 (6273.05-7143.96)	2.73 (2.57-2.9)
	50 to 54	10475.21 (9815.99-11134.43)	4.5 (4.23-4.77)
	55 to 59	12520.34 (11740.87-13299.8)	5.81 (5.46-6.16)
	60 to 64	12318.13 (11551.9-13084.37)	6.83 (6.42-7.24)
	65 to 69	12112.27 (11358.99-12865.56)	7.95 (7.48-8.43)
	70 to 74	11007.52 (10320.29-11694.75)	8.96 (8.43-9.5)
	75 to 79	7806.61 (7309.61-8303.62)	9.25 (8.71-9.81)
	80 to 84	5544.08 (5181.71-5906.46)	10.09 (9.5-10.71)

	85 to 89	3344.88 (3113.78-3575.98)	10.61 (9.98-11.26)
	90 to 94	1562.54 (1439.34-1685.75)	11.53 (10.83-12.25)
	95 plus	512.68 (457.09-568.27)	11.8 (11.04-12.58)
2025	15 to 19	420.16 (346.29-494.02)	0.13 (0.12-0.15)
	20 to 24	598.74 (526.37-671.11)	0.2 (0.18-0.22)
	25 to 29	815.9 (729.93-901.88)	0.28 (0.25-0.3)
	30 to 34	1337.48 (1211.22-1463.73)	0.45 (0.41-0.48)
	35 to 39	2345.35 (2142.82-2547.88)	0.78 (0.72-0.84)
	40 to 44	4156.25 (3818.26-4494.24)	1.51 (1.4-1.63)
	45 to 49	6822.9 (6287.1-7358.71)	2.75 (2.54-2.96)
	50 to 54	10642.14 (9823.97-11460.31)	4.54 (4.21-4.89)
	55 to 59	12830.66 (11851.76-13809.57)	5.87 (5.44-6.32)
	60 to 64	12962.29 (11974.74-13949.85)	6.92 (6.41-7.44)
	65 to 69	12317.09 (11378-13256.19)	8 (7.41-8.6)
	70 to 74	11482.38 (10605.38-12359.39)	9.04 (8.37-9.72)

	75 to 79	8309.25 (7667.03-8951.46)	9.31 (8.62-10.01)
	80 to 84	5754.39 (5301.24-6207.54)	10.25 (9.5-11.03)
	85 to 89	3480.24 (3195.73-3764.75)	10.73 (9.93-11.55)
	90 to 94	1654.02 (1505.76-1802.29)	11.77 (10.89-12.68)
	95 plus	558.29 (494.39-622.19)	12.04 (11.1-13.01)
2026	15 to 19	425.98 (341.07-510.88)	0.13 (0.11-0.16)
	20 to 24	605.25 (522.55-687.95)	0.2 (0.18-0.22)
	25 to 29	822.37 (724.85-919.88)	0.28 (0.25-0.3)
	30 to 34	1342.78 (1196.76-1488.8)	0.45 (0.41-0.5)
	35 to 39	2397.94 (2154.38-2641.51)	0.79 (0.72-0.87)
	40 to 44	4299.04 (3880.83-4717.25)	1.53 (1.39-1.67)
	45 to 49	6960.09 (6298.75-7621.44)	2.77 (2.52-3.03)
	50 to 54	10799.26 (9787.9-11810.63)	4.58 (4.17-5.02)
	55 to 59	13128.48 (11905.59-14351.37)	5.94 (5.4-6.49)
	60 to 64	13607.33 (12341.65-14873)	7.01 (6.38-7.67)

	65 to 69	12560.6 (11391.2-13730)	8.04 (7.31-8.8)
	70 to 74	11930.48 (10818.91-13042.05)	9.11 (8.29-9.97)
	75 to 79	8836.24 (8007.14-9665.35)	9.38 (8.53-10.26)
	80 to 84	5985.3 (5416.31-6554.29)	10.37 (9.43-11.35)
	85 to 89	3629.01 (3275.26-3982.76)	10.87 (9.88-11.9)
	90 to 94	1743.73 (1562.68-1924.77)	11.99 (10.89-13.13)
	95 plus	600.67 (525.93-675.41)	12.31 (11.15-13.51)
2027	15 to 19	431.65 (334.05-529.25)	0.14 (0.11-0.17)
	20 to 24	612.34 (516.54-708.13)	0.2 (0.17-0.23)
	25 to 29	829.31 (717.55-941.07)	0.28 (0.25-0.31)
	30 to 34	1351.07 (1181.09-1521.05)	0.46 (0.41-0.51)
	35 to 39	2442.51 (2150.62-2734.4)	0.81 (0.72-0.9)
	40 to 44	4434.64 (3921.04-4948.25)	1.55 (1.38-1.73)
	45 to 49	7128.92 (6316.5-7941.34)	2.8 (2.49-3.12)
	50 to 54	10965.25 (9727.92-12202.57)	4.62 (4.11-5.15)

	55 to 59	13432.69 (11922.74-14942.63)	6.01 (5.35-6.7)
	60 to 64	14184 (12591.54-15776.45)	7.1 (6.33-7.92)
	65 to 69	12910.13 (11459.57-14360.69)	8.1 (7.22-9.03)
	70 to 74	12344.1 (10956.67-13731.53)	9.18 (8.18-10.24)
	75 to 79	9340.68 (8286.3-10395.05)	9.47 (8.43-10.55)
	80 to 84	6250.65 (5538.83-6962.46)	10.45 (9.31-11.65)
	85 to 89	3791.91 (3352.71-4231.11)	11.05 (9.84-12.33)
	90 to 94	1825 (1604.17-2045.83)	12.17 (10.83-13.58)
	95 plus	636.36 (548.46-724.26)	12.59 (11.18-14.08)
2028	15 to 19	437.22 (325.36-549.09)	0.14 (0.11-0.17)
	20 to 24	620.25 (508.68-731.82)	0.2 (0.17-0.24)
	25 to 29	836.9 (708.3-965.51)	0.28 (0.24-0.32)
	30 to 34	1362.22 (1164.41-1560.03)	0.46 (0.4-0.53)
	35 to 39	2474.41 (2128.73-2820.08)	0.82 (0.71-0.93)
	40 to 44	4563.39 (3940.59-5186.19)	1.57 (1.36-1.79)

	45 to 49	7340.62 (6350.23-8331.01)	2.82 (2.46-3.22)
	50 to 54	11140.17 (9647.37-12632.97)	4.65 (4.05-5.3)
	55 to 59	13726.26 (11892.1-15560.43)	6.08 (5.3-6.93)
	60 to 64	14670.27 (12711.89-16628.66)	7.18 (6.25-8.18)
	65 to 69	13435.13 (11640.83-15229.43)	8.18 (7.12-9.32)
	70 to 74	12685.07 (10990.36-14379.77)	9.25 (8.05-10.53)
	75 to 79	9819.48 (8504.09-11134.88)	9.55 (8.32-10.88)
	80 to 84	6590.35 (5702.32-7478.39)	10.5 (9.14-11.95)
	85 to 89	3957.74 (3418.09-4497.38)	11.26 (9.8-12.82)
	90 to 94	1904.15 (1636.41-2171.89)	12.33 (10.73-14.04)
	95 plus	677.63 (572.81-782.45)	12.88 (11.19-14.7)
2029	15 to 19	442.81 (315.17-570.46)	0.14 (0.1-0.18)
	20 to 24	629 (499.07-758.93)	0.2 (0.17-0.24)
	25 to 29	845.3 (697.26-993.35)	0.28 (0.24-0.33)
	30 to 34	1373.62 (1144.67-1602.57)	0.47 (0.39-0.54)

	35 to 39	2494.47 (2090.74-2898.2)	0.83 (0.7-0.97)
	40 to 44	4691.4 (3945.59-5437.21)	1.59 (1.35-1.85)
	45 to 49	7592.2 (6395.45-8788.94)	2.86 (2.42-3.32)
	50 to 54	11320.53 (9544.69-13096.37)	4.69 (3.98-5.46)
	55 to 59	13987.25 (11797.8-16176.7)	6.16 (5.23-7.17)
	60 to 64	15096.44 (12735.27-17457.61)	7.27 (6.17-8.46)
	65 to 69	14115.53 (11907.45-16323.62)	8.29 (7.04-9.65)
	70 to 74	12949.7 (10923.09-14976.31)	9.31 (7.91-10.84)
	75 to 79	10294.52 (8680.7-11908.34)	9.64 (8.19-11.23)
	80 to 84	7011.94 (5908.33-8115.56)	10.54 (8.95-12.27)
	85 to 89	4128.05 (3472.74-4783.36)	11.47 (9.74-13.35)
	90 to 94	1990.91 (1667.85-2313.97)	12.47 (10.59-14.52)
	95 plus	730.88 (603.94-857.81)	13.18 (11.17-15.37)
2030	15 to 19	448.32 (303.45-593.18)	0.14 (0.1-0.19)
	20 to 24	638.29 (487.56-789.02)	0.2 (0.16-0.25)

25 to 29	854.59 (684.42-1024.75)	0.28 (0.23-0.34)
30 to 34	1385.4 (1122.16-1648.64)	0.47 (0.39-0.56)
35 to 39	2507.72 (2041.93-2973.51)	0.84 (0.7-1.01)
40 to 44	4814.58 (3932.87-5696.29)	1.62 (1.34-1.93)
45 to 49	7866.11 (6434.93-9297.29)	2.89 (2.39-3.44)
50 to 54	11524.11 (9434.83-13613.39)	4.72 (3.91-5.63)
55 to 59	14222.66 (11648.54-16796.78)	6.23 (5.15-7.42)
60 to 64	15486.57 (12685.59-18287.54)	7.35 (6.08-8.76)
65 to 69	14876.96 (12186.31-17567.62)	8.41 (6.96-10.02)
70 to 74	13192.38 (10805.29-15579.48)	9.37 (7.75-11.16)
75 to 79	10762.25 (8812.72-12711.78)	9.74 (8.06-11.6)
80 to 84	7489.64 (6129.25-8850.03)	10.61 (8.78-12.65)
85 to 89	4310.46 (3522.45-5098.47)	11.66 (9.64-13.89)
90 to 94	2085.38 (1698.02-2472.74)	12.62 (10.43-15.04)
95 plus	787.66 (634.03-941.3)	13.47 (11.12-16.07)

	15 to 19	453.45 (290.18-616.73)	0.14 (0.1-0.19)
	20 to 24	647.68 (474.03-821.34)	0.21 (0.16-0.26)
	25 to 29	864.72 (669.84-1059.6)	0.29 (0.23-0.35)
	30 to 34	1397.79 (1097.51-1698.07)	0.47 (0.38-0.58)
	35 to 39	2520.15 (1988.47-3051.83)	0.85 (0.69-1.04)
	40 to 44	4927.8 (3899.93-5955.68)	1.64 (1.32-2.01)
	45 to 49	8145.64 (6455.34-9835.95)	2.93 (2.35-3.57)
2031	50 to 54	11768.61 (9333.14-14204.07)	4.77 (3.83-5.82)
	55 to 59	14447.58 (11461.81-17433.36)	6.29 (5.06-7.68)
	60 to 64	15864.31 (12587.61-19141.02)	7.44 (5.98-9.08)
	65 to 69	15641.93 (12411.49-18872.36)	8.53 (6.86-10.42)
	70 to 74	13480.35 (10695.12-16265.58)	9.43 (7.59-11.52)
	75 to 79	11207.56 (8890.19-13524.93)	9.83 (7.91-12)
	80 to 84	7988.63 (6333.77-9643.5)	10.71 (8.61-13.07)
	85 to 89	4511.83 (3572.57-5451.09)	11.81 (9.5-14.42)

	90 to 94	2188.3 (1727.31-2649.28)	12.8 (10.29-15.64)
	95 plus	842 (658.12-1025.89)	13.73 (11.03-16.79)
	15 to 19	458.93 (275.62-642.23)	0.14 (0.09-0.2)
	20 to 24	657.02 (458.13-855.91)	0.21 (0.15-0.27)
	25 to 29	875.8 (653.05-1098.55)	0.29 (0.22-0.36)
	30 to 34	1411.12 (1070.28-1751.96)	0.48 (0.37-0.6)
	35 to 39	2538.44 (1934.44-3142.44)	0.86 (0.67-1.08)
	40 to 44	5025.01 (3840.26-6209.76)	1.67 (1.3-2.1)
2032	45 to 49	8412.65 (6437.45-10387.85)	2.97 (2.32-3.72)
	50 to 54	12068.45 (9240.87-14896.04)	4.82 (3.76-6.04)
	55 to 59	14686.29 (11248.97-18123.61)	6.35 (4.96-7.95)
	60 to 64	16251.27 (12449.4-20053.13)	7.53 (5.89-9.44)
	65 to 69	16329.38 (12509.71-20149.05)	8.65 (6.75-10.83)
	70 to 74	13884.76 (10635.74-17133.78)	9.51 (7.43-11.92)
	75 to 79	11621.18 (8900.38-14341.98)	9.92 (7.75-12.42)

	80 to 84	8464.98 (6480.55-10449.4)	10.81 (8.45-13.55)
	85 to 89	4740.01 (3624.69-5855.33)	11.91 (9.3-14.92)
	90 to 94	2298.95 (1753.16-2844.73)	13.03 (10.17-16.33)
	95 plus	889.81 (672.73-1106.88)	13.96 (10.89-17.51)
	15 to 19	462.81 (258.73-666.88)	0.14 (0.09-0.21)
	20 to 24	666.29 (440.02-892.55)	0.21 (0.15-0.29)
	25 to 29	888.17 (634.41-1141.93)	0.29 (0.22-0.38)
	30 to 34	1425.76 (1041-1810.52)	0.48 (0.36-0.62)
	35 to 39	2562.46 (1880.04-3244.88)	0.87 (0.66-1.13)
2033	40 to 44	5096.74 (3749.44-6444.04)	1.7 (1.29-2.19)
	45 to 49	8668.15 (6384.67-10951.62)	3.01 (2.28-3.88)
	50 to 54	12443.54 (9170.98-15716.09)	4.87 (3.69-6.27)
	55 to 59	14939.15 (11013.42-18864.88)	6.4 (4.85-8.24)
	60 to 64	16627.58 (12259.84-20995.32)	7.64 (5.78-9.83)
	65 to 69	16915.42 (12472.6-21358.24)	8.76 (6.63-11.27)

	70 to 74	14482.21 (10677.49-18286.94)	9.62 (7.28-12.38)
	75 to 79	11968.78 (8822.99-15114.58)	10 (7.57-12.87)
	80 to 84	8921.04 (6574.13-11267.96)	10.93 (8.27-14.06)
	85 to 89	5027.34 (3701.1-6353.58)	11.97 (9.06-15.41)
	90 to 94	2413.51 (1772.44-3054.59)	13.28 (10.05-17.1)
	95 plus	939.48 (684.68-1194.29)	14.15 (10.7-18.23)
	15 to 19	466.46 (240.42-692.49)	0.14 (0.09-0.22)
	20 to 24	675.68 (419.84-931.52)	0.21 (0.14-0.3)
	25 to 29	901.88 (613.83-1189.92)	0.29 (0.21-0.39)
	30 to 34	1441.96 (1009.62-1874.29)	0.48 (0.35-0.64)
2034	35 to 39	2587.31 (1821.36-3353.26)	0.88 (0.65-1.17)
	40 to 44	5144.71 (3630.89-6658.54)	1.73 (1.26-2.29)
	45 to 49	8923.9 (6305.8-11542)	3.06 (2.24-4.05)
	50 to 54	12889.08 (9112.91-16665.25)	4.93 (3.61-6.52)
	55 to 59	15201.53 (10750.7-19652.36)	6.45 (4.73-8.54)

	60 to 64	16967.21 (12001.1-21933.32)	7.74 (5.68-10.24)
	65 to 69	17436.02 (12333.32-22538.73)	8.87 (6.5-11.73)
	70 to 74	15252.61 (10788.18-19717.03)	9.76 (7.15-12.91)
	75 to 79	12249.58 (8662.68-15836.47)	10.08 (7.39-13.34)
	80 to 84	9380.7 (6632.05-12129.36)	11.05 (8.09-14.61)
	85 to 89	5380.69 (3800.81-6960.56)	12.04 (8.82-15.93)
	90 to 94	2535.76 (1787.2-3284.32)	13.55 (9.93-17.93)
	95 plus	997.79 (698.47-1297.1)	14.33 (10.49-18.98)
	15 to 19	470.14 (220.83-719.46)	0.15 (0.08-0.23)
	20 to 24	685.03 (397.46-972.6)	0.21 (0.14-0.31)
	25 to 29	916.48 (590.87-1242.08)	0.29 (0.2-0.41)
2035	30 to 34	1459.86 (975.91-1943.82)	0.49 (0.34-0.66)
	35 to 39	2613.21 (1758.43-3468)	0.89 (0.63-1.21)
	40 to 44	5179.26 (3493.64-6864.87)	1.75 (1.24-2.39)
	45 to 49	9172.07 (6194.67-12149.48)	3.11 (2.2-4.24)

	50 to 54	13375.45 (9038.74-17712.16)	5 (3.54-6.8)
	55 to 59	15497.69 (10475.59-20519.79)	6.51 (4.61-8.87)
	60 to 64	17278.78 (11681.3-22876.25)	7.84 (5.56-10.67)
	65 to 69	17918.51 (12114.51-23722.51)	8.98 (6.36-12.22)
	70 to 74	16115.22 (10894.89-21335.56)	9.91 (7.02-13.49)
	75 to 79	12514.97 (8459.39-16570.56)	10.16 (7.2-13.82)
	80 to 84	9838.39 (6648.65-13028.13)	11.17 (7.91-15.2)
	85 to 89	5777.37 (3901.36-7653.38)	12.14 (8.6-16.52)
	90 to 94	2669.33 (1798.82-3539.85)	13.79 (9.77-18.78)
	95 plus	1061.36 (710.88-1411.84)	14.52 (10.28-19.79)
	15 to 19	474.08 (200.01-748.16)	0.15 (0.08-0.25)
	20 to 24	694.17 (372.76-1015.58)	0.21 (0.13-0.33)
2036	25 to 29	931.51 (565.11-1297.92)	0.3 (0.2-0.43)
	30 to 34	1479.45 (939.39-2019.51)	0.49 (0.33-0.69)
	35 to 39	2640.54 (1691.24-3589.85)	0.9 (0.61-1.26)

	40 to 44	5212.63 (3346.69-7078.57)	1.78 (1.22-2.5)
	45 to 49	9402.5 (6044.36-12760.64)	3.17 (2.17-4.44)
	50 to 54	13873.73 (8923.88-18823.59)	5.07 (3.46-7.1)
	55 to 59	15851.38 (10198.62-21504.14)	6.58 (4.5-9.22)
	60 to 64	17580.02 (11312.65-23847.39)	7.93 (5.42-11.11)
	65 to 69	18389.06 (11834.04-24944.07)	9.1 (6.22-12.76)
	70 to 74	16985.04 (10930.25-23039.82)	10.07 (6.88-14.11)
	75 to 79	12827.61 (8253.34-17401.89)	10.24 (7-14.35)
	80 to 84	10277.32 (6611.18-13943.47)	11.29 (7.72-15.82)
	85 to 89	6188.08 (3978.08-8398.09)	12.26 (8.38-17.18)
	90 to 94	2816.84 (1807.34-3826.34)	13.99 (9.56-19.61)
	95 plus	1128.87 (720.3-1537.44)	14.75 (10.08-20.69)
	15 to 19	478.03 (177.79-778.27)	0.15 (0.08-0.26)
2037	20 to 24	703.72 (346.02-1061.43)	0.22 (0.13-0.34)
	25 to 29	946.51 (536.16-1356.85)	0.3 (0.19-0.45)

	30 to 34	1500.89 (899.71-2102.06)	0.49 (0.32-0.72)
	35 to 39	2670.14 (1619.97-3720.32)	0.91 (0.6-1.31)
	40 to 44	5259.07 (3198.61-7319.54)	1.8 (1.19-2.61)
	45 to 49	9604.09 (5848.71-13359.48)	3.23 (2.13-4.66)
	50 to 54	14353.9 (8746.44-19961.36)	5.15 (3.39-7.43)
	55 to 59	16283.88 (9925.03-22642.73)	6.66 (4.39-9.62)
	60 to 64	17901.66 (10912.72-24890.59)	8.01 (5.28-11.57)
	65 to 69	18873.55 (11505.92-26241.18)	9.24 (6.09-13.34)
	70 to 74	17774.08 (10835.49-24712.66)	10.22 (6.74-14.76)
	75 to 79	13255.49 (8079.38-18431.61)	10.34 (6.82-14.93)
	80 to 84	10687.39 (6512.95-14861.83)	11.41 (7.52-16.47)
	85 to 89	6578.12 (4006.44-9149.8)	12.41 (8.17-17.91)
	90 to 94	2981.77 (1812.83-4150.7)	14.13 (9.31-20.4)
	95 plus	1199.42 (725.51-1673.34)	15.04 (9.9-21.73)
2038	15 to 19	483.7 (154.68-812.73)	0.15 (0.07-0.27)

20 to 24	710.93 (315.88-1105.99)	0.22 (0.12-0.36)
25 to 29	961.56 (503.99-1419.12)	0.3 (0.18-0.47)
30 to 34	1524.79 (856.83-2192.74)	0.5 (0.31-0.75)
35 to 39	2702.62 (1544.52-3860.71)	0.91 (0.58-1.36)
40 to 44	5318.21 (3047.75-7588.66)	1.83 (1.16-2.72)
45 to 49	9758.33 (5599.41-13917.26)	3.29 (2.08-4.89)
50 to 54	14817.64 (8507.74-21127.55)	5.23 (3.32-7.79)
55 to 59	16822.56 (9661.39-23983.73)	6.75 (4.28-10.05)
60 to 64	18243.6 (10478.97-26008.23)	8.1 (5.13-12.05)
65 to 69	19348.97 (11114.62-27583.32)	9.38 (5.95-13.96)
70 to 74	18456.46 (10601.83-26311.09)	10.37 (6.58-15.44)
75 to 79	13873.52 (7967.95-19779.09)	10.48 (6.65-15.59)
80 to 84	11040.89 (6339.97-15741.81)	11.52 (7.31-17.15)
85 to 89	6955.32 (3991.87-9918.77)	12.56 (7.96-18.69)
90 to 94	3186.64 (1825.95-4547.34)	14.23 (9.02-21.19)

	95 plus	1275.19 (727.22-1823.16)	15.36 (9.73-22.88)
2039	15 to 19	489.48 (129.88-849.08)	0.15 (0.07-0.29)
	20 to 24	717.89 (283.43-1152.35)	0.22 (0.12-0.38)
	25 to 29	976.95 (468.65-1485.24)	0.31 (0.18-0.49)
	30 to 34	1551.25 (810.32-2292.17)	0.5 (0.3-0.78)
	35 to 39	2738.49 (1464.53-4012.44)	0.92 (0.56-1.42)
	40 to 44	5379.9 (2887.05-7872.76)	1.85 (1.13-2.84)
	45 to 49	9868.46 (5302.54-14434.37)	3.35 (2.04-5.14)
	50 to 54	15285.41 (8218.64-22352.19)	5.33 (3.25-8.18)
	55 to 59	17461.66 (9391.34-25531.98)	6.84 (4.17-10.51)
	60 to 64	18600.55 (10005.25-27195.85)	8.18 (4.99-12.56)
	65 to 69	19786.04 (10643.73-28928.34)	9.53 (5.81-14.63)
	70 to 74	19072.49 (10259.86-27885.12)	10.52 (6.41-16.15)
	75 to 79	14663.91 (7887.18-21440.64)	10.65 (6.49-16.34)
	80 to 84	11342.2 (6099.4-16585.01)	11.64 (7.09-17.87)

	85 to 89	7342.94 (3946.91-10738.98)	12.72 (7.75-19.52)
	90 to 94	3436.38 (1844.36-5028.4)	14.34 (8.74-22.02)
	95 plus	1360.17 (726.76-1993.58)	15.7 (9.56-24.11)
2040	15 to 19	495.34 (103.29-887.38)	0.16 (0.07-0.31)
	20 to 24	725.01 (248.77-1201.25)	0.23 (0.11-0.4)
	25 to 29	992.45 (429.87-1555.04)	0.31 (0.17-0.52)
	30 to 34	1579.52 (759.29-2399.75)	0.51 (0.29-0.82)
	35 to 39	2778.05 (1379.25-4176.85)	0.93 (0.54-1.48)
	40 to 44	5444.66 (2716.03-8173.29)	1.87 (1.09-2.96)
	45 to 49	9954.24 (4972.07-14936.42)	3.41 (1.99-5.4)
	50 to 54	15743.63 (7869.56-23617.7)	5.43 (3.18-8.6)
	55 to 59	18161.16 (9080.76-27241.55)	6.95 (4.06-11.01)
	60 to 64	19002.89 (9503.13-28502.64)	8.27 (4.84-13.1)
	65 to 69	20194.81 (10100.17-30289.46)	9.67 (5.66-15.32)
	70 to 74	19651.32 (9828.44-29474.21)	10.68 (6.25-16.91)

	75 to 79	15547.77 (7775.16-23320.38)	10.83 (6.34-17.17)
	80 to 84	11637.84 (5818.67-17457)	11.75 (6.87-18.61)
	85 to 89	7734.2 (3865.29-11603.11)	12.88 (7.54-20.41)
	90 to 94	3713.71 (1853.46-5573.96)	14.48 (8.47-22.95)
	95 plus	1453.28 (722.21-2184.36)	16.01 (9.36-25.38)
2041	15 to 19	501.4 (74.81-927.99)	0.16 (0.06-0.32)
	20 to 24	732.62 (211.87-1253.38)	0.23 (0.11-0.42)
	25 to 29	1007.82 (387.37-1628.26)	0.31 (0.16-0.54)
	30 to 34	1608.83 (702.93-2514.74)	0.51 (0.28-0.86)
	35 to 39	2821.3 (1287.61-4354.99)	0.94 (0.53-1.54)
	40 to 44	5513.27 (2534.1-8492.45)	1.89 (1.06-3.09)
	45 to 49	10039.29 (4620.75-15457.82)	3.46 (1.94-5.67)
	50 to 54	16174.57 (7450.63-24898.51)	5.54 (3.1-9.06)
	55 to 59	18881.78 (8700.71-29062.85)	7.06 (3.96-11.55)
	60 to 64	19480.58 (8978.31-29982.85)	8.38 (4.7-13.7)

	65 to 69	20596.03 (9493.5-31698.56)	9.8 (5.49-16.03)
	70 to 74	20221.2 (9320.9-31121.51)	10.84 (6.08-17.74)
	75 to 79	16441.52 (7577.9-25305.15)	11.03 (6.19-18.04)
	80 to 84	11984.02 (5522.22-18445.82)	11.87 (6.66-19.41)
	85 to 89	8113.49 (3737.18-12489.79)	13.05 (7.32-21.35)
	90 to 94	3997.96 (1839.19-6156.74)	14.66 (8.22-23.99)
	95 plus	1553.43 (711.66-2395.19)	16.27 (9.12-26.63)
2042	15 to 19	507.76 (44.3-971.23)	0.16 (0.06-0.34)
	20 to 24	740.35 (172.42-1308.28)	0.23 (0.1-0.45)
	25 to 29	1023.97 (341.34-1706.59)	0.32 (0.16-0.57)
	30 to 34	1638.38 (640.49-2636.28)	0.52 (0.27-0.9)
	35 to 39	2868.58 (1188.59-4548.56)	0.95 (0.51-1.61)
	40 to 44	5587.55 (2340.83-8834.28)	1.91 (1.02-3.23)
	45 to 49	10151.21 (4259.29-16043.13)	3.52 (1.89-5.95)
	50 to 54	16558.77 (6953.88-26163.67)	5.65 (3.03-9.55)

	55 to 59	19582.52 (8226.92-30938.13)	7.19 (3.86-12.15)
	60 to 64	20061.01 (8429.63-31692.39)	8.49 (4.56-14.35)
	65 to 69	21025.62 (8835.95-33215.29)	9.92 (5.33-16.77)
	70 to 74	20810.39 (8745.64-32875.14)	11.03 (5.92-18.63)
	75 to 79	17258.81 (7252.36-27265.25)	11.22 (6.02-18.97)
	80 to 84	12441.79 (5226.99-19656.59)	12.01 (6.45-20.3)
	85 to 89	8470.79 (3557.32-13384.25)	13.21 (7.09-22.33)
	90 to 94	4265.84 (1789.28-6742.41)	14.87 (7.98-25.12)
	95 plus	1660.22 (693.56-2626.88)	16.47 (8.83-27.84)
2043	15 to 19	514.53 (11.55-1017.52)	0.16 (0.06-0.37)
	20 to 24	750.89 (130.75-1371.03)	0.23 (0.1-0.48)
	25 to 29	1036.88 (290.32-1783.43)	0.32 (0.15-0.6)
	30 to 34	1668.34 (571.7-2764.98)	0.53 (0.26-0.94)
	35 to 39	2921.12 (1081.56-4760.68)	0.96 (0.49-1.69)
	40 to 44	5668.85 (2135.27-9202.43)	1.93 (0.99-3.37)

	45 to 49	10289.48 (3883.31-16695.64)	3.57 (1.83-6.24)
	50 to 54	16863.97 (6370.56-27357.39)	5.77 (2.96-10.08)
	55 to 59	20266.12 (7659.18-32873.05)	7.33 (3.76-12.8)
	60 to 64	20779.43 (7854.9-33703.97)	8.63 (4.42-15.06)
	65 to 69	21483.38 (8121.87-34844.9)	10.05 (5.15-17.54)
	70 to 74	21394.19 (8088.28-34700.1)	11.23 (5.76-19.6)
	75 to 79	17977.5 (6795.87-29159.13)	11.42 (5.86-19.93)
	80 to 84	13082.63 (4944.4-21220.86)	12.2 (6.26-21.3)
	85 to 89	8788.65 (3320.2-14257.1)	13.38 (6.86-23.36)
	90 to 94	4527.94 (1708.56-7347.31)	15.08 (7.74-26.34)
	95 plus	1786.05 (671.29-2900.82)	16.63 (8.52-29.05)
2044	15 to 19	521.87 (0-1067.42)	0.17 (0.06-0.39)
	20 to 24	761.72 (85.86-1437.58)	0.24 (0.09-0.5)
	25 to 29	1049.59 (235.32-1863.85)	0.33 (0.14-0.64)
	30 to 34	1699.23 (496.31-2902.15)	0.53 (0.25-0.99)

	35 to 39	2979.16 (965.23-4993.09)	0.97 (0.47-1.77)
	40 to 44	5758.32 (1915.87-9600.78)	1.95 (0.96-3.53)
	45 to 49	10434.57 (3481.73-17387.4)	3.62 (1.77-6.54)
	50 to 54	17095.74 (5710.19-28481.29)	5.89 (2.88-10.63)
	55 to 59	20961.09 (7005.04-34917.14)	7.48 (3.66-13.5)
	60 to 64	21629.44 (7230.24-36028.64)	8.77 (4.29-15.82)
	65 to 69	21963.65 (7342.86-36584.44)	10.17 (4.98-18.36)
	70 to 74	21941.28 (7335.58-36546.98)	11.43 (5.6-20.62)
	75 to 79	18639.86 (6231.18-31048.55)	11.61 (5.68-20.95)
	80 to 84	13891.39 (4642.83-23139.95)	12.42 (6.08-22.42)
	85 to 89	9075.73 (3031.98-15119.47)	13.54 (6.63-24.45)
	90 to 94	4803.5 (1602.85-8004.15)	15.31 (7.49-27.63)
	95 plus	1934.98 (643.15-3226.82)	16.79 (8.22-30.32)
2045	15 to 19	529.92 (0-1121.52)	0.17 (0.05-0.41)
	20 to 24	772.82 (37.51-1508.13)	0.24 (0.09-0.53)

25 to 29	1062.71 (176.25-1949.17)	0.33 (0.14-0.67)
30 to 34	1730.66 (413.7-3047.62)	0.54 (0.24-1.05)
35 to 39	3041.32 (837.74-5244.91)	0.99 (0.45-1.86)
40 to 44	5856.71 (1680.38-10033.05)	1.98 (0.92-3.69)
45 to 49	10587.55 (3052.4-18122.7)	3.67 (1.71-6.85)
50 to 54	17288.26 (4989.85-29586.66)	6.01 (2.8-11.22)
55 to 59	21648.61 (6252.61-37044.6)	7.64 (3.56-14.26)
60 to 64	22561.63 (6518.4-38604.86)	8.92 (4.16-16.65)
65 to 69	22503.33 (6502.68-38503.99)	10.31 (4.81-19.25)
70 to 74	22462.69 (6491.32-38434.06)	11.62 (5.43-21.69)
75 to 79	19273.07 (5569.02-32977.13)	11.81 (5.51-22.03)
80 to 84	14791.78 (4273.32-25310.25)	12.68 (5.92-23.65)
85 to 89	9367.61 (2704.95-16030.26)	13.71 (6.4-25.58)
90 to 94	5086.11 (1466.89-8705.33)	15.55 (7.26-29.02)
95 plus	2099.26 (603.04-3595.49)	17 (7.93-31.74)

	15 to 19	538.79 (0-1180.45)	0.17 (0.05-0.44)
	20 to 24	784.38 (0-1583.39)	0.25 (0.08-0.57)
	25 to 29	1076.74 (112.83-2040.66)	0.34 (0.13-0.71)
	30 to 34	1762.21 (323.25-3201.16)	0.55 (0.23-1.1)
	35 to 39	3106.17 (697.35-5514.98)	1 (0.44-1.95)
	40 to 44	5964.08 (1425.86-10502.31)	2 (0.89-3.87)
	45 to 49	10750.08 (2592.72-18907.45)	3.72 (1.65-7.18)
2046	50 to 54	17482.6 (4222.1-30743.11)	6.13 (2.72-11.82)
	55 to 59	22303.97 (5391.17-39216.76)	7.81 (3.47-15.08)
	60 to 64	23527.3 (5689.25-41365.34)	9.09 (4.04-17.55)
	65 to 69	23139.21 (5596.82-40681.6)	10.47 (4.65-20.21)
	70 to 74	22981.39 (5559.18-40403.59)	11.81 (5.25-22.79)
	75 to 79	19903.1 (4814.07-34992.12)	12.02 (5.34-23.21)
	80 to 84	15703.49 (3797.58-27609.39)	12.94 (5.75-24.97)
	85 to 89	9706.89 (2346.1-17067.68)	13.88 (6.17-26.8)

	90 to 94	5363.77 (1294.74-9432.8)	15.79 (7.01-30.48)
	95 plus	2270.67 (545.8-3995.55)	17.26 (7.66-33.32)
	15 to 19	548.52 (0-1244.74)	0.18 (0.05-0.47)
	20 to 24	796.57 (0-1664.07)	0.25 (0.08-0.6)
	25 to 29	1091.14 (44.57-2137.71)	0.34 (0.12-0.76)
	30 to 34	1795.51 (224.67-3366.34)	0.56 (0.22-1.17)
	35 to 39	3172.17 (542.49-5801.84)	1.01 (0.42-2.05)
	40 to 44	6081.28 (1149.25-11013.31)	2.03 (0.85-4.06)
2047	45 to 49	10925.88 (2099.77-19751.99)	3.77 (1.59-7.53)
	50 to 54	17727.26 (3412.92-32041.59)	6.24 (2.63-12.46)
	55 to 59	22899.7 (4413.68-41385.72)	7.99 (3.38-15.96)
	60 to 64	24475.14 (4719.9-44230.39)	9.28 (3.92-18.53)
	65 to 69	23905.28 (4611.52-43199.05)	10.65 (4.5-21.26)
	70 to 74	23537.62 (4541.06-42534.19)	11.99 (5.06-23.94)
	75 to 79	20555.59 (3965.24-37145.94)	12.26 (5.18-24.48)

	80 to 84	16544.04 (3190.71-29897.36)	13.2 (5.58-26.36)
	85 to 89	10139.4 (1954.2-18324.61)	14.09 (5.95-28.13)
	90 to 94	5628.2 (1083.16-10173.25)	16.03 (6.77-32.01)
	95 plus	2439.56 (467.28-4411.84)	17.55 (7.41-35.04)
	15 to 19	559.15 (0-1314.96)	0.18 (0.04-0.5)
	20 to 24	809.55 (0-1751)	0.26 (0.08-0.64)
	25 to 29	1109.9 (0-2248.85)	0.35 (0.12-0.8)
	30 to 34	1823.48 (116.62-3530.34)	0.57 (0.21-1.23)
	35 to 39	3239.72 (372.03-6107.4)	1.03 (0.4-2.16)
2048	40 to 44	6211.06 (847.57-11574.54)	2.06 (0.82-4.27)
	45 to 49	11117.76 (1569.57-20665.96)	3.82 (1.53-7.9)
	50 to 54	18021.75 (2551.73-33491.78)	6.35 (2.54-13.12)
	55 to 59	23390.42 (3316.85-43464)	8.19 (3.28-16.91)
	60 to 64	25409.98 (3606.04-47213.92)	9.49 (3.8-19.6)
	65 to 69	24845.46 (3527.51-46163.42)	10.85 (4.35-22.4)

	70 to 74	24131.53 (3426.53-44836.54)	12.18 (4.88-25.15)
	75 to 79	21207.26 (3010.77-39403.75)	12.52 (5.02-25.86)
	80 to 84	17299.1 (2455.22-32142.97)	13.47 (5.4-27.81)
	85 to 89	10724.84 (1520.91-19928.78)	14.35 (5.75-29.64)
	90 to 94	5871.84 (831.16-10912.53)	16.28 (6.52-33.63)
	95 plus	2618.57 (368.52-4868.62)	17.86 (7.15-36.89)
	15 to 19	570.78 (0-1391.92)	0.19 (0.04-0.54)
	20 to 24	823.6 (0-1845.32)	0.26 (0.07-0.69)
	25 to 29	1129.34 (0-2367.66)	0.36 (0.11-0.85)
	30 to 34	1851.48 (0-3703.47)	0.58 (0.2-1.3)
2049	35 to 39	3309.85 (184.66-6435.04)	1.05 (0.38-2.28)
	40 to 44	6354.06 (516.75-12191.37)	2.09 (0.79-4.49)
	45 to 49	11328.25 (996.75-21659.74)	3.88 (1.47-8.3)
	50 to 54	18332.2 (1623.92-35040.48)	6.46 (2.45-13.81)
	55 to 59	23783.53 (2111.67-45455.38)	8.38 (3.18-17.91)

	60 to 64	26368.77 (2344.4-50393.13)	9.71 (3.69-20.76)
	65 to 69	25953.37 (2309.18-49597.55)	11.06 (4.2-23.63)
	70 to 74	24757.61 (2203.27-47311.94)	12.37 (4.7-26.44)
	75 to 79	21829.99 (1942.27-41717.72)	12.78 (4.86-27.32)
	80 to 84	18013.62 (1602-34425.23)	13.73 (5.22-29.35)
	85 to 89	11453.31 (1017.44-21889.19)	14.66 (5.57-31.33)
	90 to 94	6104.67 (540.78-11668.55)	16.53 (6.28-35.34)
	95 plus	2818.93 (247.65-5390.21)	18.18 (6.9-38.86)
	15 to 19	583.52 (0-1476.58)	0.19 (0.04-0.57)
	20 to 24	838.96 (0-1948.29)	0.27 (0.07-0.73)
	25 to 29	1149.42 (0-2494.71)	0.36 (0.11-0.91)
2050	30 to 34	1880.59 (0-3888.62)	0.59 (0.19-1.38)
	35 to 39	3381.88 (0-6785.09)	1.06 (0.37-2.41)
	40 to 44	6507.52 (152.14-12862.9)	2.12 (0.76-4.73)
	45 to 49	11558.98 (374.53-22743.43)	3.94 (1.41-8.72)

	50 to 54	18660.7 (622.32-36699.07)	6.57 (2.36-14.53)
	55 to 59	24126.44 (809.32-47443.57)	8.58 (3.08-18.97)
	60 to 64	27327.08 (920.43-53733.73)	9.95 (3.58-22.01)
	65 to 69	27170.54 (917.13-53423.95)	11.29 (4.06-24.97)
	70 to 74	25458.49 (860.14-50056.84)	12.58 (4.52-27.82)
	75 to 79	22434.83 (757.68-44111.98)	13.04 (4.69-28.84)
	80 to 84	18710.29 (631.25-36789.32)	14.02 (5.04-30.99)
	85 to 89	12260.25 (412.62-24107.88)	15 (5.39-33.18)
	90 to 94	6348.65 (212.17-12485.14)	16.79 (6.03-37.12)
	95 plus	3032.03 (99.33-5964.73)	18.52 (6.66-40.97)
Uterine cancer			
2022	20 to 24	582.35 (526.05-638.65)	0.19 (0.18-0.21)
	25 to 29	797.48 (730.32-864.64)	0.27 (0.26-0.28)
	30 to 34	1305.6 (1213.5-1397.69)	0.43 (0.41-0.45)
	35 to 39	2165.66 (2034.6-2296.73)	0.75 (0.72-0.78)

	40 to 44	3776.67 (3576.52-3976.82)	1.46 (1.4-1.53)
	45 to 49	6507.46 (6194.31-6820.62)	2.69 (2.58-2.81)
	50 to 54	10074.4 (9616-10532.79)	4.39 (4.21-4.57)
	55 to 59	11784.78 (11258.07-12311.49)	5.69 (5.46-5.92)
	60 to 64	11299.15 (10793.38-11804.92)	6.68 (6.41-6.96)
	65 to 69	11580.12 (11063.88-12096.37)	7.85 (7.54-8.18)
	70 to 74	10026.04 (9573.28-10478.81)	8.8 (8.45-9.16)
	75 to 79	7013.62 (6683.45-7343.78)	9.18 (8.81-9.56)
	80 to 84	5138.29 (4884.75-5391.83)	9.74 (9.35-10.15)
	85 to 89	3097.92 (2929-3266.83)	10.37 (9.94-10.81)
	90 to 94	1403.55 (1308.34-1498.76)	11.03 (10.56-11.51)
	95 plus	442.81 (396.28-489.34)	11.4 (10.86-11.97)
	20 to 24	587.31 (527.8-646.81)	0.2 (0.18-0.21)
2023	25 to 29	803.55 (732.56-874.54)	0.27 (0.26-0.29)
	30 to 34	1321.8 (1222.39-1421.2)	0.43 (0.41-0.46)

	35 to 39	2226.94 (2080.76-2373.11)	0.76 (0.72-0.8)
	40 to 44	3885.92 (3657.15-4114.69)	1.48 (1.4-1.55)
	45 to 49	6606.84 (6245.76-6967.91)	2.71 (2.58-2.85)
	50 to 54	10287.68 (9749.44-10825.92)	4.44 (4.23-4.66)
	55 to 59	12178.35 (11550.44-12806.27)	5.75 (5.47-6.03)
	60 to 64	11742.34 (11136.61-12348.06)	6.74 (6.42-7.07)
	65 to 69	11883.69 (11272.05-12495.32)	7.91 (7.53-8.29)
	70 to 74	10521.3 (9975.6-11066.99)	8.88 (8.45-9.31)
	75 to 79	7366.01 (6972.28-7759.73)	9.22 (8.78-9.67)
	80 to 84	5342.88 (5046.7-5639.07)	9.92 (9.44-10.4)
	85 to 89	3219.07 (3026.09-3412.04)	10.5 (9.99-11.02)
	90 to 94	1476.96 (1371.12-1582.79)	11.28 (10.72-11.85)
	95 plus	470 (420.24-519.76)	11.59 (10.96-12.24)
2024	20 to 24	592.75 (528.03-657.47)	0.2 (0.18-0.21)
	25 to 29	809.67 (732.51-886.83)	0.27 (0.26-0.29)

	30 to 34	1331.53 (1220.83-1442.24)	0.44 (0.41-0.47)
	35 to 39	2287.49 (2117.62-2457.37)	0.77 (0.72-0.82)
	40 to 44	4015.54 (3740.98-4290.11)	1.49 (1.4-1.59)
	45 to 49	6708.51 (6273.05-7143.96)	2.73 (2.57-2.9)
	50 to 54	10475.21 (9815.99-11134.43)	4.5 (4.23-4.77)
	55 to 59	12520.34 (11740.87-13299.8)	5.81 (5.46-6.16)
	60 to 64	12318.13 (11551.9-13084.37)	6.83 (6.42-7.24)
	65 to 69	12112.27 (11358.99-12865.56)	7.95 (7.48-8.43)
	70 to 74	11007.52 (10320.29-11694.75)	8.96 (8.43-9.5)
	75 to 79	7806.61 (7309.61-8303.62)	9.25 (8.71-9.81)
	80 to 84	5544.08 (5181.71-5906.46)	10.09 (9.5-10.71)
	85 to 89	3344.88 (3113.78-3575.98)	10.61 (9.98-11.26)
	90 to 94	1562.54 (1439.34-1685.75)	11.53 (10.83-12.25)
	95 plus	512.68 (457.09-568.27)	11.8 (11.04-12.58)
2025	20 to 24	598.74 (526.37-671.11)	0.2 (0.18-0.22)

25 to 29	815.9 (729.93-901.88)	0.28 (0.25-0.3)
30 to 34	1337.48 (1211.22-1463.73)	0.45 (0.41-0.48)
35 to 39	2345.35 (2142.82-2547.88)	0.78 (0.72-0.84)
40 to 44	4156.25 (3818.26-4494.24)	1.51 (1.4-1.63)
45 to 49	6822.9 (6287.1-7358.71)	2.75 (2.54-2.96)
50 to 54	10642.14 (9823.97-11460.31)	4.54 (4.21-4.89)
55 to 59	12830.66 (11851.76-13809.57)	5.87 (5.44-6.32)
60 to 64	12962.29 (11974.74-13949.85)	6.92 (6.41-7.44)
65 to 69	12317.09 (11378-13256.19)	8 (7.41-8.6)
70 to 74	11482.38 (10605.38-12359.39)	9.04 (8.37-9.72)
75 to 79	8309.25 (7667.03-8951.46)	9.31 (8.62-10.01)
80 to 84	5754.39 (5301.24-6207.54)	10.25 (9.5-11.03)
85 to 89	3480.24 (3195.73-3764.75)	10.73 (9.93-11.55)
90 to 94	1654.02 (1505.76-1802.29)	11.77 (10.89-12.68)
95 plus	558.29 (494.39-622.19)	12.04 (11.1-13.01)

	20 to 24	605.25 (522.55-687.95)	0.2 (0.18-0.22)
	25 to 29	822.37 (724.85-919.88)	0.28 (0.25-0.3)
	30 to 34	1342.78 (1196.76-1488.8)	0.45 (0.41-0.5)
	35 to 39	2397.94 (2154.38-2641.51)	0.79 (0.72-0.87)
	40 to 44	4299.04 (3880.83-4717.25)	1.53 (1.39-1.67)
	45 to 49	6960.09 (6298.75-7621.44)	2.77 (2.52-3.03)
	50 to 54	10799.26 (9787.9-11810.63)	4.58 (4.17-5.02)
2026	55 to 59	13128.48 (11905.59-14351.37)	5.94 (5.4-6.49)
	60 to 64	13607.33 (12341.65-14873)	7.01 (6.38-7.67)
	65 to 69	12560.6 (11391.2-13730)	8.04 (7.31-8.8)
	70 to 74	11930.48 (10818.91-13042.05)	9.11 (8.29-9.97)
	75 to 79	8836.24 (8007.14-9665.35)	9.38 (8.53-10.26)
	80 to 84	5985.3 (5416.31-6554.29)	10.37 (9.43-11.35)
	85 to 89	3629.01 (3275.26-3982.76)	10.87 (9.88-11.9)
	90 to 94	1743.73 (1562.68-1924.77)	11.99 (10.89-13.13)

	95 plus	600.67 (525.93-675.41)	12.31 (11.15-13.51)
2027	20 to 24	612.34 (516.54-708.13)	0.2 (0.17-0.23)
	25 to 29	829.31 (717.55-941.07)	0.28 (0.25-0.31)
	30 to 34	1351.07 (1181.09-1521.05)	0.46 (0.41-0.51)
	35 to 39	2442.51 (2150.62-2734.4)	0.81 (0.72-0.9)
	40 to 44	4434.64 (3921.04-4948.25)	1.55 (1.38-1.73)
	45 to 49	7128.92 (6316.5-7941.34)	2.8 (2.49-3.12)
	50 to 54	10965.25 (9727.92-12202.57)	4.62 (4.11-5.15)
	55 to 59	13432.69 (11922.74-14942.63)	6.01 (5.35-6.7)
	60 to 64	14184 (12591.54-15776.45)	7.1 (6.33-7.92)
	65 to 69	12910.13 (11459.57-14360.69)	8.1 (7.22-9.03)
	70 to 74	12344.1 (10956.67-13731.53)	9.18 (8.18-10.24)
	75 to 79	9340.68 (8286.3-10395.05)	9.47 (8.43-10.55)
	80 to 84	6250.65 (5538.83-6962.46)	10.45 (9.31-11.65)
	85 to 89	3791.91 (3352.71-4231.11)	11.05 (9.84-12.33)

	90 to 94	1825 (1604.17-2045.83)	12.17 (10.83-13.58)
	95 plus	636.36 (548.46-724.26)	12.59 (11.18-14.08)
	20 to 24	620.25 (508.68-731.82)	0.2 (0.17-0.24)
	25 to 29	836.9 (708.3-965.51)	0.28 (0.24-0.32)
	30 to 34	1362.22 (1164.41-1560.03)	0.46 (0.4-0.53)
	35 to 39	2474.41 (2128.73-2820.08)	0.82 (0.71-0.93)
	40 to 44	4563.39 (3940.59-5186.19)	1.57 (1.36-1.79)
	45 to 49	7340.62 (6350.23-8331.01)	2.82 (2.46-3.22)
2028	50 to 54	11140.17 (9647.37-12632.97)	4.65 (4.05-5.3)
	55 to 59	13726.26 (11892.1-15560.43)	6.08 (5.3-6.93)
	60 to 64	14670.27 (12711.89-16628.66)	7.18 (6.25-8.18)
	65 to 69	13435.13 (11640.83-15229.43)	8.18 (7.12-9.32)
	70 to 74	12685.07 (10990.36-14379.77)	9.25 (8.05-10.53)
	75 to 79	9819.48 (8504.09-11134.88)	9.55 (8.32-10.88)
	80 to 84	6590.35 (5702.32-7478.39)	10.5 (9.14-11.95)

	85 to 89	3957.74 (3418.09-4497.38)	11.26 (9.8-12.82)
	90 to 94	1904.15 (1636.41-2171.89)	12.33 (10.73-14.04)
	95 plus	677.63 (572.81-782.45)	12.88 (11.19-14.7)
2029	20 to 24	629 (499.07-758.93)	0.2 (0.17-0.24)
	25 to 29	845.3 (697.26-993.35)	0.28 (0.24-0.33)
	30 to 34	1373.62 (1144.67-1602.57)	0.47 (0.39-0.54)
	35 to 39	2494.47 (2090.74-2898.2)	0.83 (0.7-0.97)
	40 to 44	4691.4 (3945.59-5437.21)	1.59 (1.35-1.85)
	45 to 49	7592.2 (6395.45-8788.94)	2.86 (2.42-3.32)
	50 to 54	11320.53 (9544.69-13096.37)	4.69 (3.98-5.46)
	55 to 59	13987.25 (11797.8-16176.7)	6.16 (5.23-7.17)
	60 to 64	15096.44 (12735.27-17457.61)	7.27 (6.17-8.46)
	65 to 69	14115.53 (11907.45-16323.62)	8.29 (7.04-9.65)
	70 to 74	12949.7 (10923.09-14976.31)	9.31 (7.91-10.84)
	75 to 79	10294.52 (8680.7-11908.34)	9.64 (8.19-11.23)

	80 to 84	7011.94 (5908.33-8115.56)	10.54 (8.95-12.27)
	85 to 89	4128.05 (3472.74-4783.36)	11.47 (9.74-13.35)
	90 to 94	1990.91 (1667.85-2313.97)	12.47 (10.59-14.52)
	95 plus	730.88 (603.94-857.81)	13.18 (11.17-15.37)
	20 to 24	638.29 (487.56-789.02)	0.2 (0.16-0.25)
	25 to 29	854.59 (684.42-1024.75)	0.28 (0.23-0.34)
	30 to 34	1385.4 (1122.16-1648.64)	0.47 (0.39-0.56)
	35 to 39	2507.72 (2041.93-2973.51)	0.84 (0.7-1.01)
	40 to 44	4814.58 (3932.87-5696.29)	1.62 (1.34-1.93)
2030	45 to 49	7866.11 (6434.93-9297.29)	2.89 (2.39-3.44)
	50 to 54	11524.11 (9434.83-13613.39)	4.72 (3.91-5.63)
	55 to 59	14222.66 (11648.54-16796.78)	6.23 (5.15-7.42)
	60 to 64	15486.57 (12685.59-18287.54)	7.35 (6.08-8.76)
	65 to 69	14876.96 (12186.31-17567.62)	8.41 (6.96-10.02)
	70 to 74	13192.38 (10805.29-15579.48)	9.37 (7.75-11.16)

	75 to 79	10762.25 (8812.72-12711.78)	9.74 (8.06-11.6)
	80 to 84	7489.64 (6129.25-8850.03)	10.61 (8.78-12.65)
	85 to 89	4310.46 (3522.45-5098.47)	11.66 (9.64-13.89)
	90 to 94	2085.38 (1698.02-2472.74)	12.62 (10.43-15.04)
	95 plus	787.66 (634.03-941.3)	13.47 (11.12-16.07)
2031	20 to 24	78.25 (28.68-127.82)	0.02 (0.01-0.04)
	25 to 29	152.58 (85.94-219.22)	0.05 (0.03-0.07)
	30 to 34	311.07 (208.08-414.07)	0.11 (0.08-0.14)
	35 to 39	685.08 (479.18-890.98)	0.23 (0.17-0.31)
	40 to 44	1377.78 (974.52-1781.05)	0.46 (0.34-0.61)
	45 to 49	2128.21 (1511.67-2744.75)	0.76 (0.57-1.01)
	50 to 54	3313.62 (2359.44-4267.81)	1.34 (0.99-1.77)
	55 to 59	5115.48 (3648.1-6582.85)	2.23 (1.65-2.93)
	60 to 64	7311.26 (5218.49-9404.03)	3.43 (2.54-4.51)
	65 to 69	8110.83 (5790.51-10431.14)	4.42 (3.28-5.82)

	70 to 74	6803.72 (4856.51-8750.94)	4.76 (3.53-6.27)
	75 to 79	6219.93 (4439.25-8000.61)	5.46 (4.04-7.18)
	80 to 84	4530.48 (3231.4-5829.56)	6.07 (4.5-7.99)
	85 to 89	2382.61 (1695.84-3069.38)	6.24 (4.62-8.21)
	90 to 94	1206.77 (855.22-1558.32)	7.06 (5.23-9.3)
	95 plus	435.89 (304.09-567.7)	7.11 (5.25-9.38)
	20 to 24	78.52 (23.72-133.31)	0.02 (0.01-0.04)
	25 to 29	152.38 (77.48-227.29)	0.05 (0.03-0.08)
	30 to 34	308.94 (192.89-424.99)	0.1 (0.07-0.15)
	35 to 39	676.08 (446.41-905.75)	0.23 (0.16-0.32)
2032	40 to 44	1383.9 (924.85-1842.95)	0.46 (0.33-0.63)
	45 to 49	2193.79 (1472.42-2915.17)	0.77 (0.55-1.06)
	50 to 54	3350.8 (2254.13-4447.47)	1.34 (0.95-1.83)
	55 to 59	5072.53 (3417.29-6727.78)	2.19 (1.56-2.99)
	60 to 64	7361.66 (4963.55-9759.77)	3.41 (2.42-4.66)

	65 to 69	8504.53 (5735.49-11273.56)	4.5 (3.2-6.15)
	70 to 74	6983.54 (4708.86-9258.22)	4.78 (3.4-6.53)
	75 to 79	6404.07 (4317.68-8490.46)	5.46 (3.88-7.46)
	80 to 84	4830.73 (3255.28-6406.18)	6.17 (4.38-8.42)
	85 to 89	2507.2 (1686.35-3328.05)	6.3 (4.47-8.6)
	90 to 94	1262.57 (845.91-1679.23)	7.15 (5.08-9.77)
	95 plus	459.38 (303.49-615.28)	7.21 (5.1-9.85)
2033	20 to 24	78.84 (18.38-139.3)	0.02 (0.01-0.05)
	25 to 29	152.51 (68.56-236.46)	0.05 (0.03-0.08)
	30 to 34	307.31 (176.9-437.73)	0.1 (0.07-0.15)
	35 to 39	669.22 (413.9-924.54)	0.23 (0.15-0.33)
	40 to 44	1379.42 (865-1893.84)	0.46 (0.31-0.65)
	45 to 49	2253.61 (1419.54-3087.68)	0.78 (0.53-1.11)
	50 to 54	3420.18 (2159.21-4681.15)	1.34 (0.91-1.9)
	55 to 59	5038.11 (3184.83-6891.39)	2.16 (1.46-3.06)

	60 to 64	7395.2 (4678.59-10111.81)	3.4 (2.31-4.81)
	65 to 69	8775.81 (5553.5-11998.12)	4.54 (3.08-6.44)
	70 to 74	7334.59 (4640.79-10028.39)	4.87 (3.31-6.9)
	75 to 79	6518.96 (4124.07-8913.86)	5.45 (3.7-7.72)
	80 to 84	5116.49 (3235.56-6997.41)	6.27 (4.25-8.88)
	85 to 89	2665.03 (1682.53-3647.53)	6.35 (4.31-8.99)
	90 to 94	1324.1 (832.97-1815.23)	7.29 (4.94-10.33)
	95 plus	482.84 (299.9-665.79)	7.27 (4.93-10.32)
	20 to 24	79.25 (12.65-145.86)	0.02 (0.01-0.05)
	25 to 29	152.97 (59.12-246.82)	0.05 (0.03-0.08)
	30 to 34	306.21 (160.1-452.32)	0.1 (0.06-0.16)
2034	35 to 39	663.3 (380.63-945.97)	0.23 (0.15-0.34)
	40 to 44	1366.21 (797.45-1934.97)	0.46 (0.3-0.68)
	45 to 49	2307.75 (1353.54-3261.96)	0.79 (0.51-1.17)
	50 to 54	3519.74 (2069.18-4970.3)	1.35 (0.87-1.98)

	55 to 59	5014.75 (2951.69-7077.81)	2.13 (1.38-3.13)
	60 to 64	7398.38 (4358.14-10438.62)	3.38 (2.19-4.97)
	65 to 69	8951.17 (5274.5-12627.84)	4.55 (2.95-6.7)
	70 to 74	7833.58 (4615.58-11051.58)	5.01 (3.24-7.37)
	75 to 79	6572.36 (3871.61-9273.12)	5.41 (3.5-7.96)
	80 to 84	5390.09 (3174.2-7605.98)	6.35 (4.11-9.34)
	85 to 89	2862.64 (1683.35-4041.94)	6.41 (4.15-9.43)
	90 to 94	1393.02 (816.41-1969.62)	7.44 (4.82-10.96)
	95 plus	509.64 (295.2-724.08)	7.32 (4.73-10.79)
2035	20 to 24	79.74 (6.47-153.01)	0.02 (0.01-0.05)
	25 to 29	153.69 (49.07-258.31)	0.05 (0.02-0.09)
	30 to 34	305.65 (142.44-468.86)	0.1 (0.06-0.16)
	35 to 39	658.48 (346.43-970.53)	0.22 (0.14-0.35)
	40 to 44	1348.67 (725.73-1971.61)	0.46 (0.28-0.7)
	45 to 49	2352.68 (1272.82-3432.54)	0.8 (0.49-1.22)

	50 to 54	3640.19 (1974.27-5306.1)	1.36 (0.84-2.08)
	55 to 59	5013.82 (2722.55-7305.1)	2.11 (1.3-3.23)
	60 to 64	7374.3 (4007.66-10740.93)	3.35 (2.06-5.12)
	65 to 69	9072.07 (4932.28-13211.86)	4.55 (2.8-6.96)
	70 to 74	8405.31 (4569.69-12240.93)	5.17 (3.19-7.91)
	75 to 79	6614.1 (3594.97-9633.24)	5.37 (3.31-8.21)
	80 to 84	5651.32 (3070.93-8231.71)	6.42 (3.95-9.82)
	85 to 89	3088.5 (1676.13-4500.88)	6.49 (4-9.93)
	90 to 94	1469.79 (795.09-2144.48)	7.6 (4.68-11.63)
	95 plus	538.83 (288.31-789.35)	7.37 (4.54-11.3)
	20 to 24	80.29 (0-160.78)	0.02 (0.01-0.06)
	25 to 29	154.6 (38.3-270.9)	0.05 (0.02-0.09)
2036	30 to 34	305.66 (123.84-487.48)	0.1 (0.05-0.17)
	35 to 39	654.86 (311-998.71)	0.22 (0.13-0.36)
	40 to 44	1331.16 (652.53-2009.8)	0.45 (0.27-0.73)

	45 to 49	2385.6 (1176.75-3594.46)	0.8 (0.47-1.28)
	50 to 54	3771.46 (1865.48-5677.44)	1.38 (0.81-2.19)
	55 to 59	5045.82 (2498.94-7592.7)	2.1 (1.23-3.34)
	60 to 64	7334.29 (3635.61-11032.98)	3.31 (1.94-5.27)
	65 to 69	9171.67 (4548.59-13794.75)	4.54 (2.66-7.23)
	70 to 74	8968.15 (4447.79-13488.5)	5.32 (3.11-8.47)
	75 to 79	6709 (3326.45-10091.54)	5.36 (3.14-8.53)
	80 to 84	5890.31 (2919.96-8860.66)	6.47 (3.79-10.3)
	85 to 89	3330.06 (1648.85-5011.27)	6.6 (3.86-10.51)
	90 to 94	1555.66 (767.86-2343.45)	7.73 (4.52-12.31)
	95 plus	570.33 (278.58-862.07)	7.45 (4.36-11.88)
2037	20 to 24	80.98 (0-169.38)	0.02 (0.01-0.06)
	25 to 29	155.64 (26.72-284.56)	0.05 (0.02-0.1)
	30 to 34	306.26 (104.17-508.34)	0.1 (0.05-0.18)
	35 to 39	652.47 (274.04-1030.91)	0.22 (0.12-0.37)

	40 to 44	1317.93 (579.61-2056.25)	0.45 (0.25-0.75)
	45 to 49	2404.06 (1065.48-3742.65)	0.81 (0.45-1.34)
	50 to 54	3900.79 (1734.17-6067.42)	1.4 (0.78-2.32)
	55 to 59	5119.59 (2279.04-7960.14)	2.09 (1.16-3.47)
	60 to 64	7297.07 (3251.36-11342.78)	3.27 (1.81-5.42)
	65 to 69	9267.33 (4131.31-14403.35)	4.54 (2.52-7.52)
	70 to 74	9441.13 (4208.95-14673.3)	5.43 (3.01-9.01)
	75 to 79	6919.81 (3084.02-10755.61)	5.4 (3-8.95)
	80 to 84	6091.95 (2714.52-9469.38)	6.5 (3.61-10.78)
	85 to 89	3567.87 (1588.12-5547.62)	6.73 (3.73-11.16)
	90 to 94	1652.08 (733.15-2571.01)	7.83 (4.34-12.99)
	95 plus	604.44 (265.53-943.35)	7.58 (4.2-12.58)
	20 to 24	81.5 (0-178.24)	0.03 (0.01-0.06)
2038	25 to 29	156.84 (14.25-299.42)	0.05 (0.02-0.1)
	30 to 34	307.58 (83.32-531.84)	0.1 (0.05-0.19)

	35 to 39	651.31 (235.23-1067.4)	0.22 (0.11-0.39)
	40 to 44	1309.1 (506.24-2111.96)	0.45 (0.23-0.78)
	45 to 49	2404.62 (939.6-3869.64)	0.81 (0.42-1.4)
	50 to 54	4021.6 (1577.02-6466.18)	1.42 (0.74-2.46)
	55 to 59	5244.74 (2059.76-8429.72)	2.1 (1.1-3.64)
	60 to 64	7273.47 (2859.12-11687.81)	3.23 (1.69-5.58)
	65 to 69	9344.12 (3674.9-15013.34)	4.53 (2.38-7.83)
	70 to 74	9782.64 (3847.65-15717.64)	5.5 (2.88-9.5)
	75 to 79	7305.27 (2872.45-11738.08)	5.52 (2.9-9.54)
	80 to 84	6231.01 (2449.39-10012.63)	6.5 (3.41-11.24)
	85 to 89	3797.87 (1491.47-6104.28)	6.86 (3.6-11.85)
	90 to 94	1772.52 (694.08-2850.97)	7.92 (4.15-13.69)
	95 plus	643.06 (249.29-1036.83)	7.75 (4.06-13.4)
2039	20 to 24	82.11 (0-187.93)	0.03 (0.01-0.07)
	25 to 29	158.25 (0.77-315.73)	0.05 (0.02-0.11)

	30 to 34	309.65 (61.05-558.25)	0.1 (0.04-0.2)
	35 to 39	651.39 (194.25-1108.53)	0.22 (0.1-0.41)
	40 to 44	1302.36 (430.64-2174.07)	0.45 (0.22-0.81)
	45 to 49	2390.41 (802.71-3978.1)	0.81 (0.4-1.46)
	50 to 54	4134.06 (1394.21-6873.91)	1.44 (0.71-2.6)
	55 to 59	5418.75 (1830.78-9006.73)	2.12 (1.05-3.83)
	60 to 64	7267.34 (2457.72-12076.96)	3.2 (1.58-5.76)
	65 to 69	9385.2 (3175.66-15594.74)	4.52 (2.24-8.15)
	70 to 74	10021.71 (3391.57-16651.86)	5.53 (2.74-9.96)
	75 to 79	7844.65 (2654.14-13035.16)	5.7 (2.82-10.27)
	80 to 84	6317.17 (2136.6-10497.75)	6.48 (3.21-11.68)
	85 to 89	4024.36 (1359.84-6688.88)	6.97 (3.45-12.56)
	90 to 94	1921.87 (647.58-3196.17)	8.02 (3.97-14.46)
	95 plus	688.08 (229.48-1146.69)	7.94 (3.93-14.32)
2040	20 to 24	82.85 (0-198.68)	0.03 (0.01-0.07)

25 to 29	159.86 (0-333.57)	0.05 (0.02-0.12)
30 to 34	312.34 (37.08-587.61)	0.1 (0.04-0.21)
35 to 39	652.78 (150.75-1154.82)	0.22 (0.1-0.43)
40 to 44	1298.02 (352.13-2243.91)	0.45 (0.21-0.84)
45 to 49	2368.99 (659.04-4078.93)	0.81 (0.38-1.53)
50 to 54	4231.72 (1183.8-7279.64)	1.46 (0.68-2.75)
55 to 59	5627.76 (1578.01-9677.51)	2.15 (1-4.05)
60 to 64	7295.6 (2047.99-12543.21)	3.18 (1.48-5.97)
65 to 69	9394.14 (2638.9-16149.38)	4.5 (2.1-8.46)
70 to 74	10203.5 (2867.17-17539.84)	5.54 (2.58-10.42)
75 to 79	8463.38 (2377.69-14549.06)	5.9 (2.75-11.09)
80 to 84	6397.18 (1796.51-10997.85)	6.46 (3.01-12.14)
85 to 89	4245.78 (1191.19-7300.37)	7.07 (3.3-13.3)
90 to 94	2091.08 (584.99-3597.16)	8.15 (3.8-15.33)
95 plus	738.34 (204.3-1272.37)	8.13 (3.79-15.3)

	20 to 24	83.79 (0-210.75)	0.03 (0.01-0.08)
	25 to 29	161.64 (0-353.07)	0.05 (0.01-0.13)
	30 to 34	315.52 (11.11-619.94)	0.1 (0.04-0.22)
	35 to 39	655.55 (104.28-1206.82)	0.22 (0.09-0.45)
	40 to 44	1296.32 (269.8-2322.83)	0.44 (0.19-0.88)
	45 to 49	2348.01 (511.13-4184.88)	0.81 (0.35-1.59)
	50 to 54	4309.34 (945.5-7673.18)	1.48 (0.65-2.9)
2041	55 to 59	5856.54 (1289.1-10423.98)	2.19 (0.96-4.3)
	60 to 64	7374.13 (1625.57-13122.69)	3.17 (1.39-6.22)
	65 to 69	9385.07 (2070.88-16699.25)	4.46 (1.96-8.76)
	70 to 74	10364.62 (2288.27-18440.98)	5.56 (2.43-10.9)
	75 to 79	9079.07 (2004.07-16154.07)	6.09 (2.67-11.95)
	80 to 84	6532.72 (1441.34-11624.09)	6.47 (2.83-12.69)
	85 to 89	4453.35 (981.52-7925.18)	7.16 (3.14-14.05)
	90 to 94	2270.83 (498.94-4042.72)	8.33 (3.65-16.34)

	95 plus	793.23 (172.13-1414.34)	8.31 (3.64-16.31)
2042	20 to 24	84.87 (0-224.21)	0.03 (0-0.08)
	25 to 29	163.77 (0-374.79)	0.05 (0.01-0.14)
	30 to 34	319.06 (0-655.28)	0.1 (0.03-0.24)
	35 to 39	659.75 (54.3-1265.21)	0.22 (0.08-0.47)
	40 to 44	1297.35 (182.58-2412.13)	0.44 (0.18-0.92)
	45 to 49	2334.98 (359.54-4310.42)	0.81 (0.33-1.66)
	50 to 54	4362.16 (680.46-8043.86)	1.49 (0.61-3.05)
	55 to 59	6085.49 (953.8-11217.17)	2.23 (0.92-4.58)
	60 to 64	7516.85 (1180.69-13853.01)	3.18 (1.31-6.52)
	65 to 69	9381.68 (1475.5-17287.86)	4.43 (1.82-9.07)
	70 to 74	10524.48 (1656.35-19392.6)	5.58 (2.29-11.42)
	75 to 79	9608.87 (1511.86-17705.88)	6.25 (2.57-12.8)
	80 to 84	6784.58 (1066.83-12502.33)	6.55 (2.69-13.42)
	85 to 89	4634.38 (727.71-8541.05)	7.23 (2.97-14.81)

	90 to 94	2447.5 (382.89-4512.11)	8.53 (3.5-17.47)
	95 plus	852.4 (131.31-1573.49)	8.46 (3.47-17.33)
	20 to 24	86.43 (0-240.16)	0.03 (0-0.09)
	25 to 29	165.6 (0-397.53)	0.05 (0.01-0.14)
	30 to 34	323.02 (0-694.12)	0.1 (0.03-0.25)
	35 to 39	665.72 (0.18-1331.25)	0.22 (0.08-0.5)
	40 to 44	1301.14 (89.39-2512.89)	0.44 (0.17-0.96)
	45 to 49	2330.24 (202.15-4458.33)	0.81 (0.31-1.73)
2043	50 to 54	4383.61 (391.4-8375.83)	1.5 (0.58-3.21)
	55 to 59	6304.52 (567.85-12041.18)	2.28 (0.87-4.88)
	60 to 64	7739.07 (699.81-14778.34)	3.21 (1.23-6.87)
	65 to 69	9397.84 (851.5-17944.17)	4.39 (1.69-9.4)
	70 to 74	10666.31 (967.31-20365.31)	5.6 (2.15-11.97)
	75 to 79	10011.08 (907.59-19114.56)	6.36 (2.44-13.59)
	80 to 84	7212.82 (653.27-13772.37)	6.73 (2.58-14.38)

	85 to 89	4771.77 (431.14-9112.39)	7.26 (2.79-15.54)
	90 to 94	2621.52 (235.56-5007.49)	8.73 (3.35-18.68)
	95 plus	922.77 (80.99-1764.54)	8.59 (3.3-18.38)
2044	20 to 24	88.19 (0-258.16)	0.03 (0-0.1)
	25 to 29	167.67 (0-422.64)	0.05 (0.01-0.16)
	30 to 34	327.55 (0-737.14)	0.1 (0.03-0.27)
	35 to 39	673.53 (0-1405.97)	0.22 (0.07-0.52)
	40 to 44	1307.76 (0-2626.49)	0.44 (0.15-1.01)
	45 to 49	2329.71 (36.28-4623.15)	0.81 (0.29-1.81)
	50 to 54	4379.11 (83.28-8674.95)	1.51 (0.54-3.37)
	55 to 59	6514.03 (129.38-12898.69)	2.32 (0.83-5.19)
	60 to 64	8038.19 (162.77-15913.61)	3.26 (1.17-7.28)
	65 to 69	9438.96 (192.75-18685.17)	4.37 (1.57-9.76)
	70 to 74	10771.11 (220.7-21321.52)	5.61 (2.01-12.53)
	75 to 79	10315.65 (211.32-20419.98)	6.42 (2.3-14.34)

	80 to 84	7800.2 (159.19-15441.22)	6.98 (2.5-15.58)
	85 to 89	4875.11 (98.48-9651.74)	7.28 (2.61-16.25)
	90 to 94	2798.27 (55.33-5541.22)	8.92 (3.19-19.92)
	95 plus	1007.66 (18.1-1997.22)	8.75 (3.13-19.54)
	20 to 24	90.17 (0-278.56)	0.03 (0-0.1)
	25 to 29	170.07 (0-450.77)	0.05 (0.01-0.17)
	30 to 34	332.61 (0-784.78)	0.1 (0.03-0.29)
	35 to 39	682.92 (0-1489.79)	0.22 (0.06-0.56)
	40 to 44	1317.4 (0-2754.68)	0.44 (0.14-1.06)
2045	45 to 49	2334.06 (0-4808.36)	0.81 (0.27-1.89)
	50 to 54	4362.3 (0-8965.24)	1.52 (0.51-3.54)
	55 to 59	6703.68 (0-13770.83)	2.37 (0.79-5.52)
	60 to 64	8394.52 (0-17240.55)	3.32 (1.11-7.74)
	65 to 69	9527.78 (0-19566.25)	4.37 (1.46-10.18)
	70 to 74	10842.43 (0-22265.09)	5.61 (1.87-13.08)

	75 to 79	10567.23 (0-21699.61)	6.47 (2.16-15.1)
	80 to 84	8473.57 (0-17400.88)	7.26 (2.42-16.93)
	85 to 89	4979.16 (0-10225.84)	7.29 (2.43-16.99)
	90 to 94	2975.55 (0-6112.09)	9.1 (3.03-21.22)
	95 plus	1103.51 (0-2268.47)	8.94 (2.98-20.85)
2046	20 to 24	92.4 (0-301.85)	0.03 (0-0.11)
	25 to 29	172.93 (0-482.63)	0.05 (0.01-0.18)
	30 to 34	338.15 (0-837.51)	0.11 (0.02-0.31)
	35 to 39	693.65 (0-1583.18)	0.22 (0.06-0.59)
	40 to 44	1330.24 (0-2899.52)	0.45 (0.13-1.12)
	45 to 49	2343.76 (0-5017.64)	0.81 (0.25-1.98)
	50 to 54	4347.16 (0-9275.47)	1.52 (0.47-3.71)
	55 to 59	6864.76 (0-14639.69)	2.4 (0.74-5.86)
	60 to 64	8786.14 (0-18732.89)	3.4 (1.05-8.27)
	65 to 69	9686.25 (0-20649.89)	4.38 (1.36-10.68)

	70 to 74	10896.11 (0-23227.94)	5.6 (1.73-13.64)
	75 to 79	10802.23 (0-23027.08)	6.53 (2.02-15.89)
	80 to 84	9150.8 (0-19507.26)	7.54 (2.34-18.36)
	85 to 89	5130.69 (0-10938.16)	7.34 (2.27-17.87)
	90 to 94	3146.17 (0-6708.43)	9.26 (2.87-22.56)
	95 plus	1207.37 (0-2576.11)	9.18 (2.84-22.36)
	20 to 24	94.94 (0-328.65)	0.03 (0-0.12)
	25 to 29	176.17 (0-518.58)	0.06 (0.01-0.19)
	30 to 34	344.56 (0-896.96)	0.11 (0.02-0.33)
	35 to 39	705.44 (0-1686.71)	0.23 (0.05-0.63)
2047	40 to 44	1346.48 (0-3063.38)	0.45 (0.12-1.18)
	45 to 49	2359.11 (0-5254.75)	0.81 (0.23-2.08)
	50 to 54	4347.77 (0-9639.24)	1.53 (0.44-3.9)
	55 to 59	6989.17 (0-15485.92)	2.44 (0.7-6.21)
	60 to 64	9184.11 (0-20344.43)	3.48 (1-8.86)

	65 to 69	9934.19 (0-22003.54)	4.43 (1.27-11.25)
	70 to 74	10959.56 (0-24273.48)	5.58 (1.6-14.2)
	75 to 79	11039.62 (0-24450.21)	6.59 (1.89-16.74)
	80 to 84	9748.21 (0-21590.63)	7.78 (2.23-19.78)
	85 to 89	5376.69 (0-11909.21)	7.47 (2.14-19)
	90 to 94	3300.11 (0-7310.74)	9.4 (2.7-23.91)
	95 plus	1314.1 (0-2912.76)	9.45 (2.71-24.04)
2048	20 to 24	97.82 (0-359.7)	0.03 (0-0.13)
	25 to 29	180.49 (0-561.36)	0.06 (0.01-0.21)
	30 to 34	350.51 (0-960.49)	0.11 (0.02-0.35)
	35 to 39	718.5 (0-1802.09)	0.23 (0.05-0.67)
	40 to 44	1366.84 (0-3250.27)	0.45 (0.11-1.25)
	45 to 49	2380.26 (0-5523.38)	0.82 (0.21-2.19)
	50 to 54	4364.99 (0-10065.02)	1.54 (0.41-4.09)
	55 to 59	7065.69 (0-16279.64)	2.47 (0.66-6.57)

	60 to 64	9573.98 (0-22053.48)	3.58 (0.95-9.49)
	65 to 69	10293.89 (0-23708.93)	4.49 (1.19-11.93)
	70 to 74	11049.2 (0-25447.5)	5.58 (1.48-14.8)
	75 to 79	11262.37 (0-25938.16)	6.65 (1.76-17.65)
	80 to 84	10226.32 (0-23552.59)	7.96 (2.11-21.13)
	85 to 89	5767.48 (0-13284.05)	7.72 (2.05-20.49)
	90 to 94	3427.24 (0-7894.97)	9.5 (2.52-25.23)
	95 plus	1427.71 (0-3290.43)	9.74 (2.58-25.85)
	20 to 24	101.12 (0-395.97)	0.03 (0-0.14)
	25 to 29	185.32 (0-610.27)	0.06 (0.01-0.23)
	30 to 34	357.1 (0-1031.72)	0.11 (0.02-0.38)
2049	35 to 39	733.15 (0-1931.77)	0.23 (0.05-0.72)
	40 to 44	1391.6 (0-3463.78)	0.46 (0.1-1.32)
	45 to 49	2407.46 (0-5828.1)	0.82 (0.2-2.31)
	50 to 54	4391.45 (0-10542.91)	1.55 (0.38-4.3)

	55 to 59	7102.41 (0-17032.74)	2.5 (0.61-6.94)
	60 to 64	9956.83 (0-23871.99)	3.67 (0.9-10.16)
	65 to 69	10763.95 (0-25803.72)	4.59 (1.12-12.71)
	70 to 74	11172.15 (0-26781.18)	5.58 (1.37-15.47)
	75 to 79	11451.62 (0-27451.03)	6.71 (1.64-18.58)
	80 to 84	10616.64 (0-25449.75)	8.09 (1.98-22.43)
	85 to 89	6293.1 (0-15086.35)	8.06 (1.97-22.32)
	90 to 94	3536.45 (0-8478.93)	9.58 (2.34-26.54)
	95 plus	1551.41 (0-3721.18)	10 (2.45-27.73)
2050	20 to 24	104.9 (0-438.67)	0.03 (0-0.16)
	25 to 29	190.73 (0-666.42)	0.06 (0.01-0.24)
	30 to 34	364.6 (0-1112.64)	0.11 (0.02-0.41)
	35 to 39	749.36 (0-2077.64)	0.24 (0.04-0.77)
	40 to 44	1420.31 (0-3706.24)	0.46 (0.09-1.41)
	45 to 49	2441.19 (0-6174.64)	0.83 (0.18-2.44)

50 to 54	4428.58 (0-11083.35)	1.56 (0.35-4.52)
55 to 59	7121.05 (0-17793.6)	2.53 (0.57-7.32)
60 to 64	10316.39 (0-25770.65)	3.76 (0.84-10.86)
65 to 69	11319.95 (0-28273.46)	4.7 (1.06-13.6)
70 to 74	11356.44 (0-28363.14)	5.61 (1.26-16.22)
75 to 79	11610.7 (0-28997.96)	6.75 (1.52-19.51)
80 to 84	10961.71 (0-27376.91)	8.21 (1.85-23.73)
85 to 89	6895.64 (0-17222.71)	8.44 (1.9-24.39)
90 to 94	3651.45 (0-9120.91)	9.65 (2.17-27.91)
95 plus	1681.44 (0-4201.56)	10.27 (2.31-29.7)

Data in parentheses represent the 95% confidence intervals.
